# Peripheral blood microbial signatures in current and former smokers

**DOI:** 10.1038/s41598-021-99238-4

**Published:** 2021-10-06

**Authors:** Jarrett D. Morrow, Peter J. Castaldi, Robert P. Chase, Jeong H. Yun, Sool Lee, Yang-Yu Liu, Craig P. Hersh

**Affiliations:** 1 Channing Division of Network Medicine, Brigham and Women’s Hospital, 181 Longwood Avenue, Boston, MA 02115 USA; 2 Division of Pulmonary and Critical Care Medicine, Brigham and Women’s Hospital, Boston, MA USA

**Keywords:** Gene expression, Genomics, Microbial genetics, Sequencing

## Abstract

The human microbiome has a role in the development of multiple diseases. Individual microbiome profiles are highly personalized, though many species are shared. Understanding the relationship between the human microbiome and disease may inform future individualized treatments. We hypothesize the blood microbiome signature may be a surrogate for some lung microbial characteristics. We sought associations between the blood microbiome signature and lung-relevant host factors. Based on reads not mapped to the human genome, we detected microbial nucleic acids through secondary use of peripheral blood RNA-sequencing from 2,590 current and former smokers with and without chronic obstructive pulmonary disease (COPD) from the COPDGene study. We used the Genome Analysis Toolkit (GATK) microbial pipeline PathSeq to infer microbial profiles. We tested associations between the inferred profiles and lung disease relevant phenotypes and examined links to host gene expression pathways. We replicated our analyses using a second independent set of blood RNA-seq data from 1,065 COPDGene study subjects and performed a meta-analysis across the two studies. The four phyla with highest abundance across all subjects were Proteobacteria, Actinobacteria, Firmicutes and Bacteroidetes. In our meta-analysis, we observed associations (q-value < 0.05) between *Acinetobacter*, *Serratia*, *Streptococcus* and *Bacillus* inferred abundances and Modified Medical Research Council (mMRC) dyspnea score. Current smoking status was associated (q < 0.05) with *Acinetobacter*, *Serratia* and *Cutibacterium* abundance. All 12 taxa investigated were associated with at least one white blood cell distribution variable. Abundance for nine of the 12 taxa was associated with sex, and seven of the 12 taxa were associated with race. Host-microbiome interaction analysis revealed clustering of genera associated with mMRC dyspnea score and smoking status, through shared links to several host pathways. This study is the first to identify a bacterial microbiome signature in the peripheral blood of current and former smokers. Understanding the relationships between systemic microbial signatures and lung-related phenotypes may inform novel interventions and aid understanding of the systemic effects of smoking.

## Introduction

The human microbiome has a role in human disease and overall health outcomes^1,2^. Individual microbiome profiles are unique, although many species are shared^2,3^. Knowledge of the relationship between the human microbiome and disease may serve as a component of future comprehensive individualized treatment plans^4^. Studies of the microbiome have typically involved 16S rRNA gene sequencing^5^, with metagenomic sequencing emerging more recently^6^.

Relevance of the lung microbiome has been demonstrated in the context of lung diseases^7–9^, including chronic obstructive pulmonary disease (COPD), asthma and idiopathic pulmonary fibrosis (IPF)^10–14^. In addition, the microbiome has been assessed in healthy lung and COPD exacerbations^15,16^. These studies have involved both lung tissue^17,18^ and the airway sampling^19–23^, with some researchers integrating the microbiome data with host gene expression^13,14,17,18,21^. Study of the respiratory microbiome presents many challenges^24^, including the low microbial biomass available in the samples^25^.

It has historically been believed that peripheral blood does not contain bacteria unless an acute infection was present. Through use of culture-independent sequencing methods, evidence has emerged regarding a possible healthy human blood microbiome^26–30^. Culture-independent methods in microbiome studies do not provide evidence of whether a blood microbial signature is from transient nucleic acids or from live bacteria^31^. A blood microbial signature has been found correlated with host disease traits in schizophrenia^30^, type 2 diabetes^28^, chronic kidney disease^32^ and liver fibrosis^33^, and it may provide a link in other tissues and diseases. Use of the microbiome for disease diagnosis and prediction has proven successful in cancer^34^. As with the lung microbiome, low biomass is an issue for peripheral blood microbiome studies^35^. Sequencing of both RNA^27,30^ and the 16S rRNA gene^28,32,33^ has been used to study the peripheral blood microbial signature.

In this study, we detected microbial signatures through secondary use of whole blood RNA-sequencing data from large subsets of the COPDGene (Genetic Epidemiology of COPD) study, repurposing sequencing reads not mapped to the human genome^27,30,36^. An overarching challenge in population-based microbiome studies relates to statistical power, as testing for associations between the detected microbial profiles and variables of interest places demands on sample size. Though samples were not collected as part of a traditional microbiome study, by using a large population and a meta-analysis approach, we had enhanced power to enable findings in the blood, with its typically lower microbial signals. Using statistical tools developed for microbiome analysis, we tested associations between the identified taxa and multiple COPD-related phenotypes available in COPDGene. We used network methods to integrate the microbiome signatures with the human gene expression data to highlight microbial interactions with host pathways. Our goal was to reveal microbial signatures in peripheral blood associated with lung relevant host factors and to observe lung biology relevance. A blood microbiome signature has the potential to serve as a biomarker of disease severity and progression and may inform personalized diagnostic or treatment efforts.

## Methods

### Study subjects

COPDGene is a longitudinal cohort study that includes non-Hispanic White and African American subjects enrolled at 21 centers across the United States^37^. All subjects in this study provided written consent for study procedures, including genetic analysis. COPDGene was approved by the Institutional Review Boards at all participating centers. The subjects include more than 10,000 current and former cigarette smokers with a minimum 10 pack-years smoking history, along with a small number of non-smokers. COPD cases have airflow obstruction (FEV1/FVC < 0.7), Preserved Ratio Impaired Spirometry (PRISm) cases have preserved ratio (FEV1 < 80% predicted with FEV1/FVC ≥ 0.7)^38^ and control subjects have normal spirometry (FEV1% predicted ≥ 80% and FEV1/FVC ≥ 0.7). The five-year follow-up visit included questionnaires, pre- and post-bronchodilator spirometry, volumetric computed tomography (CT) of the chest, and blood drawn for complete blood cell count, RNA-sequencing and biomarker studies. Subjects were at least one month removed from any exacerbation event or acute respiratory infection. Exacerbations were defined by use of antibiotics and/or systemic steroids, and severe exacerbations by emergency department visit or hospital admission^39^. Details of the RNA-sequencing methods are available in the online supplement^40^. We performed meta-analyses using a primary set of data and a second independent set of replication data from the COPDGene study.

### Microbial detection

Starting from the whole blood RNA-seq data, we used reads that were not mapped to the human genome during the gene expression analysis to detect a bacterial signature. Additional filtering of the unmapped reads was performed using the PathSeq microbial detection pipeline from the Genome Analysis Toolkit (GATK4) and the host reference available from the GATK Resource Bundle^41^. This filtering addresses any remaining quality, host contamination or repetitive sequence issues. We subsequently used PathSeq to map these cleaned reads to bacterial genomes. The bacterial reference for mapping was created using representative genomes, chromosomes, contigs and scaffolds (277,422 total genomic entries; September 25, 2019) from the National Center for Biotechnology Information (NCBI), and the PathSeq reference creation tools. Taxonomy information for these bacterial genomic data was also obtained from NCBI (RefSeq-release95.catalog.gz). Using these mapping results and taxonomy data, the inferred bacterial abundance profiles in each sample were assembled using PathSeq. Included in these profiling data were the raw read counts, adjusted scores and normalized scores (compositional data from the adjusted scores that represent inferred relative abundance) for taxa within each taxonomic classification (genera and phyla). We used the TMM (trimmed mean of M values) method in the R/Bioconductor package edgeR^42^ and the RNA-seq gene expression counts from the primary analysis to normalize the PathSeq count data across samples.

### Taxa associations

We tested associations between the TMM-normalized abundance for each taxon and host variables using linear models with the R/Bioconductor package MaAsLin2 (Multivariate Association with Linear Models)^43^. The abundance values were log-transformed prior to testing. With relatively low levels of bacterial genetic content in peripheral blood, the data is inherently sparse and MaAsLin2 is particularly well suited for analysis of such microbial data. The base statistical model included the covariates age, sex, race, pack-years of smoking, smoking status (current vs. former), RNA-seq library preparation batch and study center. Using the results from our primary and replication analyses, we performed a meta-analysis by combining the p-values from these tests using Stouffer’s method via the sumz function from R package metap^44^. The directions of effect in both the primary and replication analyses were required to be the same for the p-values to be combined. For each of the models, adjustment of the combined p-values for multiple testing controlled for false discovery rate (FDR < 5%). The heatmaps of taxa associations were produced using the labeledHeatmap function from the R package WGCNA^45^.

### Contamination assessment

Nucleic acids from sources other than the peripheral blood of the study subjects could impact the analyses and potentially create a false taxonomic signature. Extraction, amplification and library-preparation kits may contain nucleic acids from water and soil bacteria^46^. Removing taxa with inferred abundances below a specified threshold was the first step in the process of addressing contamination^47^. Recent studies have shown that external contaminants more consistently correlate negatively with sample nucleic acid concentration^48,49^. Therefore, we sought to identify additional contamination by testing the Pearson correlation between taxa abundance and RNA concentration, with a correlation coefficient < -0.4 and p-value < 0.05 demonstrating the conditions for possible contamination^47^. We also examined the inferred taxa abundances across the processing batches and study centers to identify patterns suggestive of contaminant introduction through laboratory kit reagents. This study did not focus on diversity measures or detection of novel organisms, as these are areas where microbial contamination may be expected to have a greater impact. In addition, our analyses involved testing associations between host binary and quantitative characteristics and microbial taxa abundance. This helps reduce the impact of batch-specific or study-wide contamination, as correlations with host variables are not expected to be consistent and significant. Our meta-analysis in two independent sets of data mitigates the effects of contamination and enhances the ability to detect biologically relevant signatures.

### Host microbe interactions

We projected the human gene expression data onto the pathways in the Hallmark gene set collection using gene set variation analysis via the R/Bioconductor package GSVA^50^. The genes represented in both the gene expression data and the Hallmark gene sets were included in the GSVA procedure (Methods in the online supplement). The Hallmark canonical pathway set reduces redundancy found in public gene sets to enhance enrichment analyses. GSVA output is a pathway-by-subject matrix of expression data for observation of host-microbiome interactions. We used the pathways in this matrix as variables in MaAsLin2 models. Similar to the taxa-association analysis, we performed a meta-analysis by combining the p-values from these tests using Stouffer’s method^44^. The directions of effect in both the primary and replication analyses were required to be the same. We constructed a bipartite network (edges connecting taxa and pathways) using the results from these models. Communities within this network were identified using the R package CONDOR^51^. Networks and communities were visualized using the R package igraph^52^, with the GEM (graph embedder) force-directed layout algorithm.

### Ethics statement

All subjects in this study provided written consent for study procedures, including genetic analysis. The study was approved at all clinical centers by the following Institutional Review Boards: National Jewish IRB, Partners Human Research Committee, Institutional Review Board for Baylor College of Medicine and Affiliated Hospitals, Columbia University Medical Center IRB, The Duke University Health System Institutional Review Board for Clinical Investigations (DUHS IRB), Johns Hopkins Medicine Institutional Review Boards (JHM IRB), The John F. Wolf MD Human Subjects Committee of Harbor-UCLA Medical Center, Morehouse School of Medicine Institutional Review Board, Temple University Office for Human Subjects Protections Institutional Review Board, The University of Alabama at Birmingham Institutional Review Board for Human Use, University of California San Diego Human Research Protections Program, The University of Iowa Human Subjects Office, VA Ann Arbor Healthcare System IRB, University of Minnesota Research Subjects’ Protection Programs (RSPP), University of Pittsburgh Institutional Review Board, UT Health Science Center San Antonio Institutional Review Board, Health Partners Research Foundation Institutional Review Board, Medical School Institutional Review Board (IRBMED), Minneapolis VAMC IRB, and Institutional Review Board/Research Review Committee Saint Vincent Hospital – Fallon Clinic – Fallon Community Health Plan. The research methods were carried out in accordance with the relevant guidelines.

### Ethics approval and consent to participate

All subjects in this study provided written informed consent. COPDGene was approved by the Institutional Review Boards at all participating centers.

**Table:** 

Clinical center	Institution title	Protocol number
National Jewish Health	National Jewish IRB	HS-1883a
Brigham and Women’s Hospital	Partners Human Research Committee	2007-P-000554/2; BWH
Baylor College of Medicine	Institutional Review Board for Baylor College of Medicine and Affiliated Hospitals	H-22209
Michael E. DeBakey VAMC	Institutional Review Board for Baylor College of Medicine and Affiliated Hospitals	H-22202
Columbia University Medical Center	Columbia University Medical Center IRB	IRB-AAAC9324
Duke University Medical Center	The Duke University Health System Institutional Review Board for Clinical Investigations (DUHS IRB)	Pro00004464
Johns Hopkins University	Johns Hopkins Medicine Institutional Review Boards (JHM IRB)	NA_00011524
Los Angeles Biomedical Research Institute	The John F. Wolf, MD Human Subjects Committee of Harbor-UCLA Medical Center	12756–01
Morehouse School of Medicine	Morehouse School of Medicine Institutional Review Board	07–1029
Temple University	Temple University Office for Human Subjects Protections Institutional Review Board	11369
University of Alabama at Birmingham	The University of Alabama at Birmingham Institutional Review Board for Human Use	FO70712014
University of California, San Diego	University of California, San Diego Human Research Protections Program	070876
University of Iowa	The University of Iowa Human Subjects Office	200710717
Ann Arbor VA	VA Ann Arbor Healthcare System IRB	PCC 2008–110732
University of Minnesota	University of Minnesota Research Subjects’ Protection Programs (RSPP)	0801M24949
University of Pittsburgh	University of Pittsburgh Institutional Review Board	PRO07120059
University of Texas Health Sciences Center at San Antonio	UT Health Science Center San Antonio Institutional Review Board	HSC20070644H
Health Partners Research Foundation	Health Partners Research Foundation Institutional Review Board	07–127
University of Michigan	Medical School Institutional Review Board (IRBMED)	HUM00014973
Minneapolis VA Medical Center	Minneapolis VAMC IRB	4128-A
Fallon Clinic	Institutional Review Board/Research Review Committee Saint Vincent Hospital – Fallon Clinic – Fallon Community Health Plan	1143

### Consent for publication

Not applicable.

## Results

**Figure 1:**
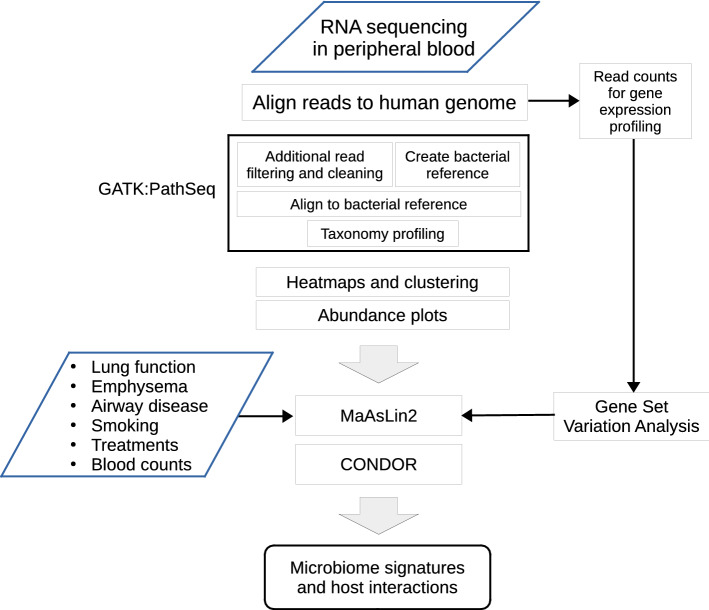
Overview of the study design illustrating the sequencing, statistical and gene enrichment framework. This illustrates the integration with host characteristics and gene expression for observations of host microbiome interaction (GATK = Genome Analysis Toolkit; MaAsLin2 = Multivariate Association with Linear Models).

After quality control procedures, RNA-seq data were available for 2,647 samples from current and former smokers from the COPDGene five-year follow-up visit. Approximately two-thirds of subjects were former smokers and twenty-five percent were African American (Table 1). There were slightly more males than females and the average age of these subjects was 65.5 years. The overall disease burden in the population was summarized in Table 1 by a comorbidity index (range 0 to 14, mean = 2.97 and standard deviation = 1.98)^53^. We performed microbial signature profiling using PathSeq and excluded 57 samples with outlying unmapped read counts (Methods in the online supplement). We then visualized the inferred relative abundance profiles and tested host associations for these 2,590 subjects (Fig. 1). Ordered by mean normalized score from PathSeq, the four taxa observed at the phylum level above an abundance-filtering 1% threshold across all subjects were Proteobacteria, Actinobacteria, Firmicutes, and Bacteroidetes. In the abundance plot of the normalized scores for these four phyla, ordered by RNA-seq library batch and study center, we observed consistent taxon distributions across the batches and study centers (Figures S1-S2 in the online supplement). Twenty genera had mean normalized scores that eclipsed the 1% threshold chosen to remove low-level contamination. We observed batch specific contamination profiles (Figures S3-S10) for eight genera (*Flavobacterium*, *Pseudomonas*, *Methylobacterium*, *Methyloversatilis*, *Streptomyces*, *Methylorubrum*, *Ralstonia* and *Nevskia*). All of these genera are known possible contaminants^24,46^ and were excluded from the analyses. We also sought to identify remaining contamination by observing the relationship between inferred abundance and nucleic acid concentration using the computation approach outlined in Methods. We again identified the aforementioned genus *Methyloversatilis* (correlation coefficient = -0.44 and p < 0.0001) as a possible contaminant.

**Table 1:** COPDGene study subjects.

Demographics	N = 2590 Mean ± SD or distribution
Age, years	65.5 ± 8.6
Sex (Female/Male)	1257/1333
Race (Non-Hispanic White/African American)	1940/650
Smoking status (Current/Former) (n = 2580)	909/1671
Smoking History, pack-years (n = 2579)	44.0 ± 24.0
**GOLD stage (n = 2541)**	
4	101
3	245
2	505
1	258
Control	1101
PRISm *	331
FEV_1_% predicted (n = 2541)	78.6 ± 24.2
FEV_1_/FVC (n = 2540)	0.68 ± 0.15
Percent emphysema at -950HU (n = 2388)	5.5 ± 9.2
Body mass index kg/m^2^ (n = 2581)	29.0 ± 6.3
Airway wall thickness, segmental bronchi (n = 2385)	1.03 ± 0.22
Severe exacerbation in the year prior ** (no/yes) (n = 2581)	2367/214
Treated with chronic oral corticosteroids (no/yes) (n = 2538)	2504/34
Survival (alive/deceased) ***	2431/159
**MMRC dyspnea score (n = 2581)**	
0	1316
1	344
2	315
3	424
4	182
6-min walk distance ft (n = 2539)	1311 ± 442
Comorbidity score **** (range 0 to 14) (n = 2581)	2.97 ± 1.98

FEV1 = forced expiratory volume in 1 s; FVC = forced vital capacity; PRISm = Preserved Ratio Impaired Spirometry; mMRC = Modified Medical Research Council dyspnea score.

*PRISm (FEV1 < 80% predicted with FEV1/FVC ≥ 0.7)^38^.

**Emergency department or hospital admission.

***Survival status as of October 2018.

****Sum of comorbidities reported, considering Coronary Heart disease, Diabetes, Congestive heart failure, Stroke, Osteoarthritis, Osteoporosis, Hypertension, High cholesterol, Gastroesophageal reflux disease, Stomach ulcers, Obesity, Sleep apnea, Hay fever, Peripheral Vascular Disease^53^.

### Genera abundance and host phenotype

We normalized the taxa counts at the genus level from PathSeq using the TMM method. We created a summary of the reads from the gene expression and PathSeq analyses for each of the 12 taxa (Table S1 in the online supplement). Using the TMM-normalized taxa abundances, we created a heatmap with clustering of samples in the columns by Bray–Curtis dissimilarity (Figure S11 in the online supplement). In the color coded tracks for BMI, race, sex, library preparation batch, study center, COPD status and smoking status, we observed visual clustering only by batch (grouping of samples from the same batch). A variable for library batch was included as a covariate in the statistical models to mitigate batch effects and reduce spurious findings. We tested associations between the TMM-normalized abundances for each taxon at the genus level and host phenotype, exposure, treatment and trait variables using linear models with MaAsLin2 (Table S2 in the online supplement). We summarized the findings in a heatmap of the p-values and effect sizes (Figure S12 in the online supplement).

Using an independent replication set of 1,065 samples from the COPDGene five-year follow-up visit (Table S3 in the online supplement), we detected microbial signatures using PathSeq and normalized the taxa counts at the genus level using the TMM method (Table S1 in the online supplement). Contamination was not observed in these data for the 12 taxa using the same methods as in the initial dataset. We performed association tests using the models and methods from the primary analysis and the TMM-normalized taxa abundances for the 12 taxa in the replication set. We summarized the findings in a heatmap of the p-values and effect sizes (Figure S13 in the online supplement).

### Meta-analysis

**Figure 2:**
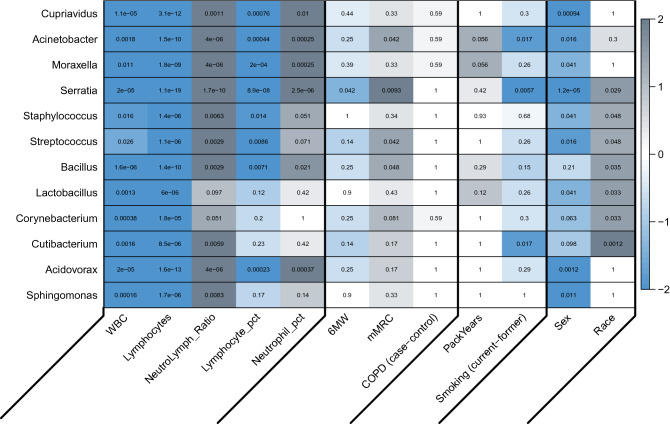
Heatmap of the associations between genera inferred abundance and host-related variables for the meta-analysis. Variables with at least one finding with FDR < 10% were included. The value in each cell is the adjusted q-value. The color scale for the cells represents the sign of the effect multiplied by negative log_10_ of the q-values, with intensity proportional to significance and gray shading representing positively correlated associations and blue shading representing negatively correlated associations. Results with discordant directions of effect in the meta-analysis are set to q = 1 (white) (heatmap produced using the labeledHeatmap function from the R package WGCNA^45^). Variables with at least one significant association are included (WBC = white blood cell count, Lymphocytes = lymphocyte count, NeutroLymph_Ratio = ratio of neutrophil counts to lymphocyte counts, Lymphocyte_pct = percentage of lymphocytes, Neutrophil_pct = percentage of neutrophils, 6 MW = six-minute walk distance, mMRC = Modified Medical Research Council dyspnea score, COPD (case–control) = COPD cases vs. controls, PackYears = pack-years history of smoking, Smoking (current-former) = current vs. former smoking status).

The p-values from the primary and replication analyses were combined for each of the association tests using Stouffer’s method requiring the directions of effect be the same. A heatmap was created to summarize the meta-analysis results (Fig. 2, Figure S14 in the online supplement) with the color intensity indicating significance (negative log transformed q-values) and gray or blue shading indicating the effect direction. Scatter or box plots of the model residuals of the inferred TMM abundance for the significant (FDR < 5%) meta-analysis findings were created in the primary (Figure S15 in the online supplement) and replication (Figure S16 in the online supplement) sets of data to illustrate the relationships between taxa abundance and the variables of interest.

From the meta-analysis (Fig. 2), we observed associations between smoking status (current vs. former) and *Acinetobacter* (q = 0.017), *Serratia* (q = 0.0057) and *Cutibacterium* (q = 0.017) abundance. Two measures of functional capacity (6-min walk distance and mMRC dyspnea scale) were associated with at least one taxon. *Acinetobacter* (q = 0.042), *Serratia* (q = 0.0093), *Streptococcus* (q = 0.042) and *Bacillus* (q = 0.048) abundances were associated with mMRC, with a higher dyspnea score corresponding to higher bacterial abundance. *Serratia* (q = 0.042) abundance was associated with 6-min walk distance (6 MW), with higher bacterial abundance corresponding to lower 6-min walk distances. All 12 taxa were associated (q < 0.05) with at least one white blood cell distribution variable. Neutrophil levels and bacterial abundance were positively correlated. Conversely, lymphocyte levels were negatively correlated with abundance. Abundance for nine of the 12 taxa was associated (q < 0.05) with sex, with lower bacterial abundance in males. Seven of the 12 taxa were associated with race, with bacterial abundance lower in non-Hispanic white participants.

### Host-microbiome interactions

**Figure 3:**
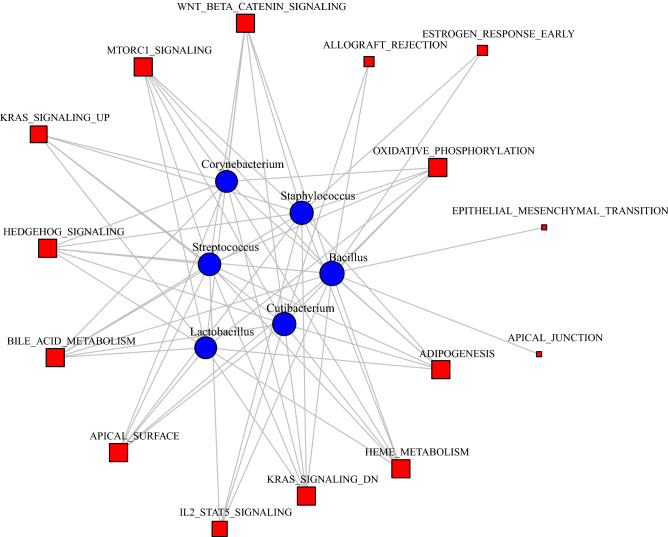
Community from the bipartite network from the host-microbiome interaction analysis with relevance to COPD, dyspnea and smoking associations. Edges represent a significant (FDR < 5%) association between genus abundance (blue circles) and the expression of the human Hallmark pathway (red squares) in the meta-analysis (figure produced using the R package igraph^52^).

We sought to highlight host-microbiome interactions using microbial abundance profiles and host gene expression pathways. We created a matrix of pathway expression for the Hallmark sets from MSigDB using the R/Bioconductor package GSVA and the human blood RNA-seq data in both the primary and replication data. We tested the association between TMM-normalized taxa abundance and host pathways in both sets of data for each of the 12 genera using models, adjusting for age, sex, race, pack-years of smoking, current smoking status (vs, former), library prep batch and study center. The associations across all taxa and pathways were summarized for both sets of data in a heatmaps (Figures S17 and S18 in the online supplement). The p-values from the primary and replication analyses were combined for each of the association tests using Stouffer’s method requiring the directions of effect be the same and a heatmap was created to summarize the results (Figure S19 in the online supplement). We used network methods to visualize the large set of significant findings. We constructed a bipartite network using the significant (FDR < 5%) associations as edges (edge weights = -log10(p-value)) between taxa and pathways (Figure S20 in the online supplement). Using CONDOR (see Methods), we identified three communities within this network (Figures S21 and S22 in the online supplement) with one of particular relevance to our taxa-association findings (Fig. 3). This community has six genera (*Streptococcus*, *Cutibacterium, Corynebacterium*, *Lactobacillus*, *Staphylococcus*, and *Bacillus*) and 15 host pathways, including WNT BETA CATENIN SIGNALING, MTORC1 SIGNALING, and OXIDATIVE PHOSPHORYLATION . Within these communities we observe clustering of genera with shared pathway associations, suggesting joint influence on the host processes.

## Discussion

We re-purposed peripheral blood RNA-sequencing data in a large sample set from the COPDGene Study. Using RNA-sequencing reads that did not map to the human genome, we identified microbial signatures at both the phylum and genus levels. We tested associations between inferred abundance and host-related variables. At the phylum level, we identified Proteobacteria, Actinobacteria, Firmicutes, and Bacteroidetes. Recent studies using both 16S rRNA gene sequencing and unmapped human RNA-seq data have shown that peripheral blood typically includes a nucleic acid signature of these phyla^26,27,35^.

### Taxa associations

Detection at the genus level produced a larger set of taxa, with all 12 taxa significantly associated with at least one host-related variable. Eight of the genera had at least six significant findings. For the associations between taxa abundance and white blood cell composition, we observed positive correlation for neutrophil percentage and neutrophil-to-lymphocyte ratio. Although the role of neutrophils in the establishment of the microbiome can be complex^54^ the positive correlation is plausible given the role of neutrophils in the defense against bacterial infections. We observed a positive association between the genera *Acinetobacter* and *Streptococcus* and mMRC dyspnea score. *Acinetobacter* is a known cause of acute exacerbations and lung infections^55–57^. *Acinetobacter* airway abundance may also be a marker of outcome for critically ill COPD patients^58^. *Streptococcus pneumoniae* is a common cause of respiratory infections and has been observed in the airway of patients with exacerbations^57^ and has been isolated from sputum samples in COPD patients in both a stable and an exacerbation state^59^. The abundance of *Serratia* and *Bacillus* was also associated with mMRC dyspnea score. Although *Serratia* and *Bacillus* species are less frequently associated with lung infections, *Serratia* has been identified in patients with exacerbations of COPD^60,61^. *Bacillus* was isolated from the lung of stable COPD subjects^62^ and subjects with more variable microbiomes during a longitudinal study of sputum in COPD^63^. In the study by Bouquet et al.^63^, microbiota variability corresponded to higher exacerbation frequency and frequent viral infections in stable COPD. The association between *Serratia* abundance and six minute walk distance highlights another association with relevance to pulmonary functional capacity and outcomes in COPD^64^.

*Acinetobacter*, *Serratia* and *Cutibacterium* abundance was associated with current smoking status, compared to former smokers. Species in the *Acinetobacter* and *Serratia* have been identified in cigarettes^65^ providing a possible mechanism for introduction of these taxa, though an explanation for higher abundance in the peripheral blood of former smokers is not apparent at this time. Community acquired *Acinetobacter* infections, including bacteremia, were also found more in patients with a history of heavy smoking^66^. *Cutibacterium* species are members of the upper respiratory tract microbiome^67,68^ and although smoking has an impact on the microbiome of the upper respiratory tract^69,70^ evidence regarding the influence of smoking on *Cutibacterium* is lacking. Irrespective of individual taxa, the impact of smoking on bacterial infections and the microbiome are complex^71,72^, particularly in the context of COPD^73,74^. Together, this information suggests relevance for the identified taxa in the lung microbiome and respiratory infections with possible implications in chronic or persistent dyspnea and inflammation.

Further efforts will be required to determine whether these associations in peripheral blood highlight cross-tissue mechanisms similar to the immunomodulatory effects observed in the gut-lung axis^75,76^, or perhaps similar to interactions or microbial translocations observed between liver and gut in liver disease^77^. Despite any direction of effect ambiguity, together these findings suggest we may be capturing lung disease relevant microbial signatures in peripheral blood.

The associations between nine taxa and sex are supported by previous findings regarding sex-specific microbiome characteristics in the gut^78,79^. Previous studies highlighted sex differences with respect to bacterial infections, including respiratory infections^80^, and relevance in the relationship between airway microbiome and asthma^81^. Likewise, gut microbiota diversity may vary across ethnicity^82,83^, supporting our taxa abundance associations with race. The associations between blood taxa and both sex and race may provide insight into systemic host bacterial responses and inform development of personalized therapeutics.

### Host-microbiome networks

We leveraged the human RNA-seq data from the same samples to explore host-microbiome interactions using network methods for significant taxa and host pathway associations. Within the communities of the bipartite network, genera with common pathway associations were clustered, providing insight into shared influence on the host processes. For one particular community within the bipartite network (Fig. 3), we observed clustering of *Streptococcus* (associated with mMRC dyspnea score) with *Cutibacterium* (associated with current smoking status) through several host pathways, including OXIDATIVE PHOSPHORYLATION, WNT BETA CATENIN SIGNALING, and MTORC1 SIGNALING. Pathways in Fig. 3 are involved in aspects of COPD. In regards to oxidative phosphorylation, mitochondrial reactive oxygen species production and mitochondrial dysfunction are believed to have a role in the development of lung diseases including COPD^84^, with implication in exercise capacity^85^. It has been suggested that cross-talk between the bacterial microbiome and mitochondria is a component of overall microbiome interactions with the host^86^.

The mTORC1 signaling pathway has been implicated in lung cell senescence and emphysema^87^ and is involved in airway inflammation^88^ and development of corticosteroid resistance driven by cigarette smoke^89^. Having a prominent role in regulation of immune responses^90,91^, the mTOR pathway, in particular, responds to environmental changes and regulates intracellular processes^92^. The mTOR pathway may have a role in determining the composition of the gut microbiome^93,94^.

Airway down-regulation of the Wnt/beta-catenin pathway has been observed in smokers^95^, suggesting a role in the development of smoking-related airway disease and airway inflammation in COPD^96^. With a role in cell proliferation and cellular morphology^97^, Wnt/beta-catenin signaling is a process bacterial pathogens may exploit to better establish infection^98^, providing a possible target for future antimicrobial therapeutics^99^. Although the microbial signature observed in our study does not appear to be pathogen-specific, both the establishment and maintenance of the bacterial microbiome and the regulation of a host pathogen defense involve a shared complex relationship with host immune responses^100^.

Together, these findings suggest we have detected a systemic blood signature of host-microbe interactions with pathogenic relevance and perhaps linked to the COPD-relevant associations we identified. This bipartite network approach demonstrates a versatile method for observation of these host-microbiome interactions. The approach is similar to previous airway host-microbiome interaction studies, though focused on a knowledge-based pathway approach instead of unsupervised dimensionality reduction of gene expression data using principal component analysis (PCA)^21^. The edges in this network may highlight taxa with shared interactions or influence on host biological processes. Both the blood microbial signatures and the structure of these host interactions may inform patient stratification or personalized medicine efforts related to COPD and exacerbations. These efforts could involve particular host pathway or gene targets, identified by their relationship to COPD-relevant microbial taxa using these methods.

### Limitations

There are several limitations to the current study. In this secondary analysis of blood RNA-sequencing data, we are capturing RNA from bacterial genes. These mapped reads are serving as a proxy for abundance. Future studies involving 16S rRNA gene or whole-genome shotgun sequencing in parallel with the host transcriptome analysis will provide further insight into the blood microbial signatures. Although the existence of a healthy blood microbiome remains a subject of debate^35^, we have replicated taxa from previous blood microbiome 16S rRNA gene and RNA sequencing studies^26,27,30^, demonstrating the generalizability of this approach. Future metagenomic studies with concurrent blood and lung or airway samples, perhaps in a longitudinal context, will be required to determine to what extent peripheral blood recapitulates the lung microbiome. This may also reveal mechanisms responsible for overlapping microbial signatures, such as bacterial translocation, and further identify any transient behavior of these signatures. Given the relatively small effect sizes, the applicability of these findings in a clinical context will be considered in future studies. The sequencing data from this study was not obtained for use in a microbiome study. Therefore, specific bacterial contamination mitigation procedures were not included in the COPDGene protocol, beyond sterile blood acquisition. We assessed for contamination using visual inspection of our data and statistical testing, and we excluded taxa with any potential evidence of contamination. A replication dataset was included to ensure validity of our results. In future studies, protocols involving the inclusion of negative controls and treatment of kit reagents to reduce contaminating nucleic acid content and other measures will help to address the issue of sample contamination^24,46^.

## Conclusions

In this study of the blood microbiome, we were able to identify COPD-relevant bacterial signatures in a secondary analysis of peripheral blood RNA-seq data from a large cohort of smokers. Analyses at the genus level found associations between blood microbial signals and multiple COPD-relevant traits. Using a network approach on the paired human RNA-seq and microbial datasets, we identified host transcriptomic pathways linking multiple taxa, highlighting a useful method for future studies of the human microbiome and transcriptome. Together these findings demonstrate that the peripheral blood microbial signature and host-microbiome interactions may have the potential to capture relevant lung microbiome features and biology. This study provides an initial step toward discovery of composite blood biomarkers for use in predictive disease models to inform personalized treatments of chronic smoking-related diseases.

## Supplementary Information

Supplementary Information 1.

Supplementary Information 2.

Supplementary Information 3.

## Data Availability

Phenotype and the primary RNA sequencing data are available in dbGaP, accessions phs000179 and phs000765. The replication data will be available in dbGaP when processing is completed.

## Abbreviations

AWT: Airway Wall Thickness
COPD: Chronic Obstructive Pulmonary Disease
CONDOR: COmplex Network Description Of Regulators
COPDGene: Genetic Epidemiology of COPD
CT: Computed Tomography
FEV1: Forced Expiratory Volume in 1 s
FVC: Forced Vital Capacity
FDR: False Discovery Rate
GATK: Genome Analysis Toolkit
GSVA: Gene Set Variation Analysis
IPF: Idiopathic Pulmonary Fibrosis
MaAsLin2: Multivariate Association with Linear Models
mMRC: Modified Medical Research Council dyspnea score
NCBI: National Center for Biotechnology Information
Pi10: SRWA-Pi10; square root wall area of a hypothetical airway with 10 mm internal perimeter
PRISm: Preserved Ratio Impaired Spirometry
6 MW: 6-Minute Walk distance
TMM: Trimmed Mean of M values
WBC: White Blood Cell
