# Supplementary material for: Peripheral blood microbial signatures in current and former smokers
Source: Sci Rep. 2021 Oct 6;11:19875. doi: 10.1038/s41598-021-99238-4 (PMC8494912; doi:10.1038/s41598-021-99238-4)
Supplement: Supplementary file 1 — Supplementary Information 1. [file 41598_2021_99238_MOESM1_ESM.pdf]

Peripheral blood microbial signatures in current and former smokers

Jarrett D. Morrow[1#], Peter J. Castaldi[1], Robert P. Chase[1], Jeong H. Yun[1], Sool Lee[1], Yang-Yu Liu[1], Craig P. Hersh[1,2]

1. Channing Division of Network Medicine, Brigham and Women's Hospital, Boston, MA
2. Division of Pulmonary and Critical Care Medicine, Brigham and Women's Hospital, Boston, MA

Methods

RNA sequencing

Sample processing was previously described for earlier stages of these data [1] and those data are available in GEO (accession number GSE97531) and dbGaP (accessions phs000179 and phs000765). Briefly, total RNA was extracted from PAXgene™ Blood RNA tubes using the Qiagen PreAnalytiX PAXgene Blood miRNA Kit (Qiagen, Valencia, CA). Extracted RNA samples with RNA integrity number greater than seven and a concentration of at least 25 μg/ul were sequenced. Globin reduction and cDNA library preparation for total RNA was performed with the Illumina TruSeq Stranded Total RNA with Ribo-Zero Globin kit (Illumina, Inc., San Diego, CA). Paired end reads with nominal 75 bp length were generated on an Illumina HiSeq 2500 flow cell. Sequencing was performed to an average depth of 20 million reads.

Data Processing

The quality control pipeline for these sequencing reads included FastQC [2] and RNA-SeQC [3]. Adapter trimming was performed using Skewer [4]. STAR aligner version 2.4.0 h [5] was used to map the reads to the GRCH38 genome reference and RSubreads produced gene-level counts [6] with the Ensembl version 81 gene annotation [7]. As part of the cleaning and quality control process, we confirmed expression consistent with reported sex, and concordance between variants called from RNA sequencing reads and corresponding DNA genotyping. Two samples were excluded from the primary set due to kinship issues. Data for genes with variance in the upper 90th percentile and average read counts greater than five were retained and intersected with the Hallmark gene sets from MSigDB [8]. A total of 3,304 genes were included in the host interaction analysis for the primary data and 3,472 for the second independent set.

Microbial detection – quality control

During quality evaluation, we removed one outlying processing batch (57 samples) from the primary data set with a mean total read count four standard deviations from the mean of the total read data. This outlying batch may represent potential contamination, as its mean was significantly higher. Heatmaps of taxa and samples were produced using the R package pheatmap [9] with visual clustering of samples performed using Bray-Curtis dissimilarity from the vegdist function in the R package vegan [10] and clustering of taxa by euclidean distance. Abundance plots were created using the R package ggplot2 [11].

Supplemental Figures

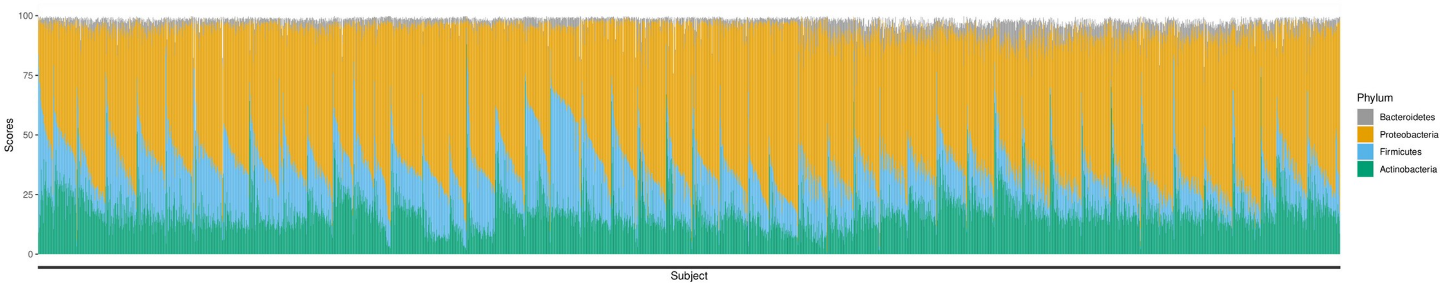

Figure S1. Abundance plots of the normalized scores for the top four phyla ordered by processing batch (created using the R package ggplot2 [11])

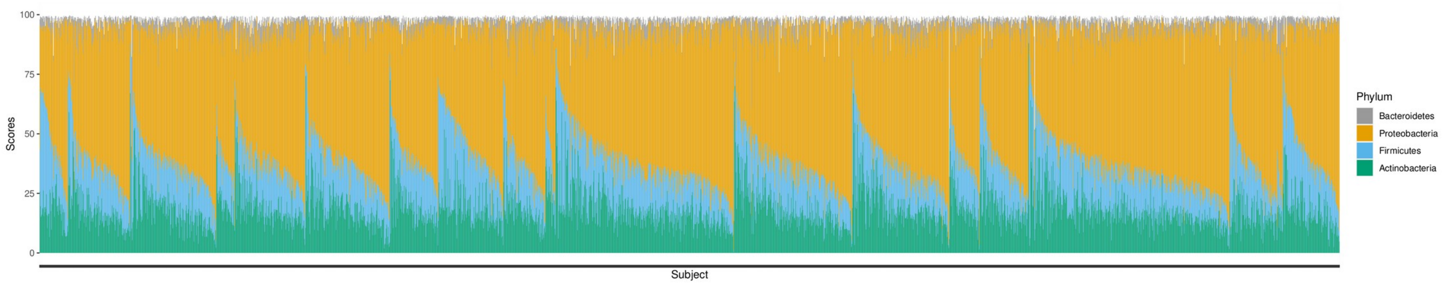

Figure S2. Abundance plots of the normalized scores for the top four phyla ordered by study center (created using the R package ggplot2 [11])

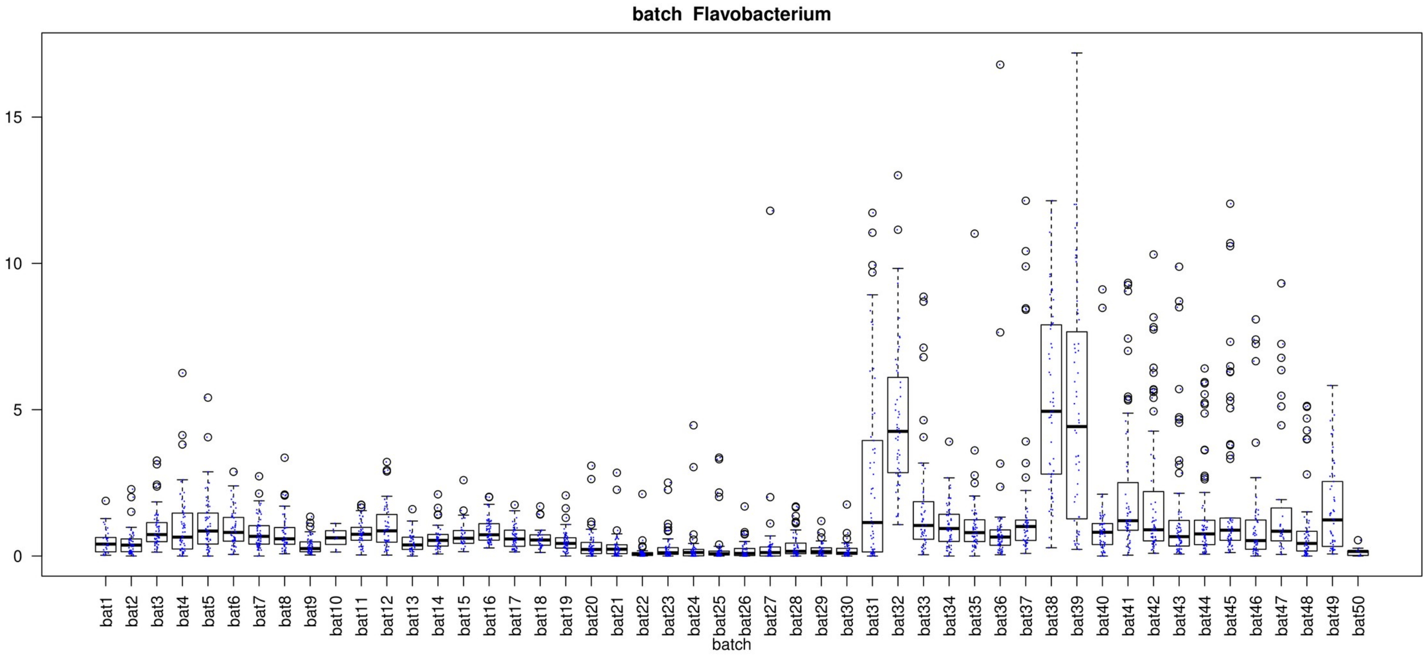

Figure S3. Box plots of inferred abundance values for the genus *Flavobacterium* for each processing batch (created using the statistical environment R [12])

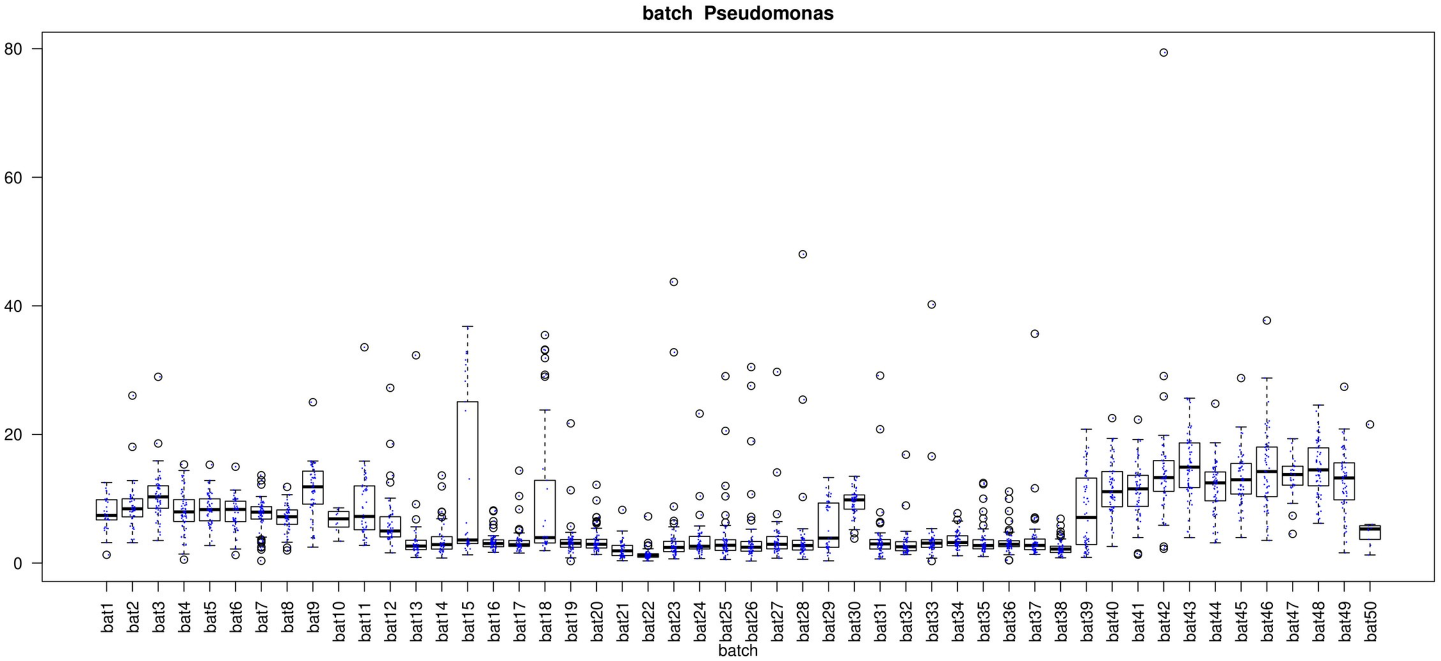

Figure S4. Box plots of inferred abundance values for the genus *Pseudomonas* for each processing batch (created using the statistical environment R [12])

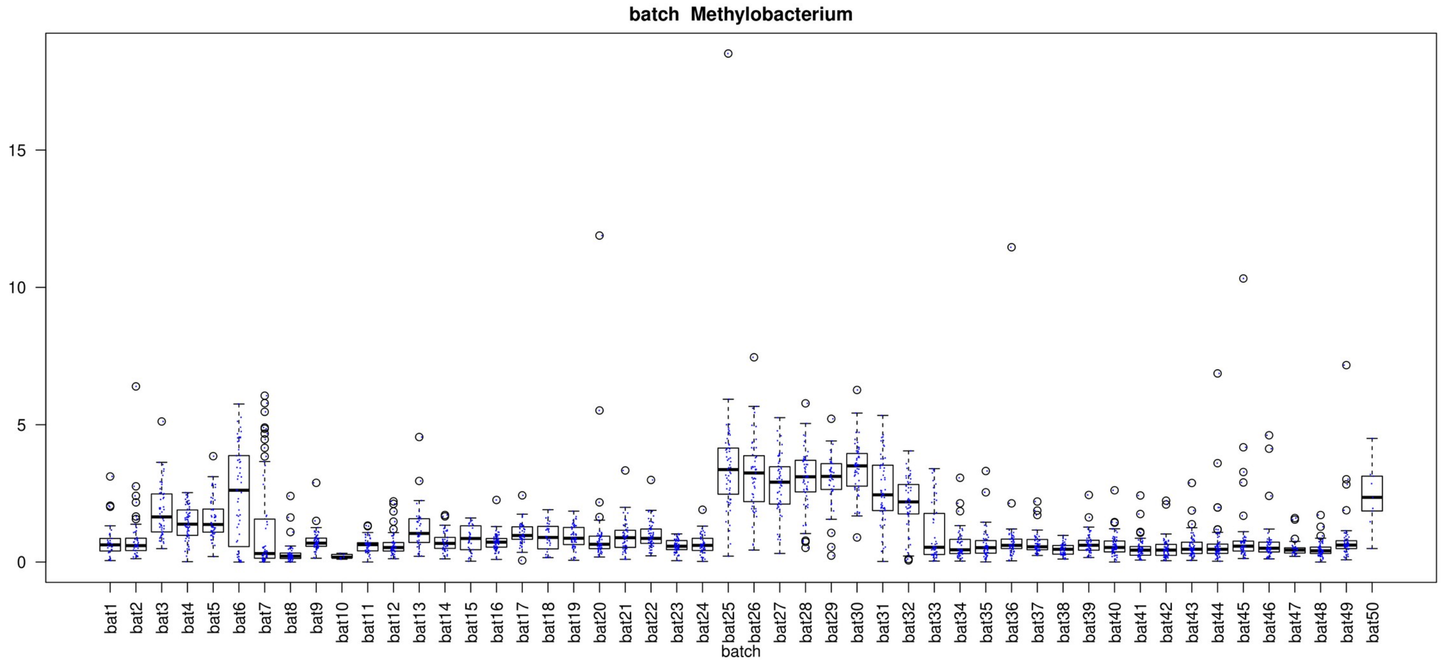

Figure S5. Box plots of inferred abundance values for the genus *Methylobacterium* for each processing batch (created using the statistical environment R [12])

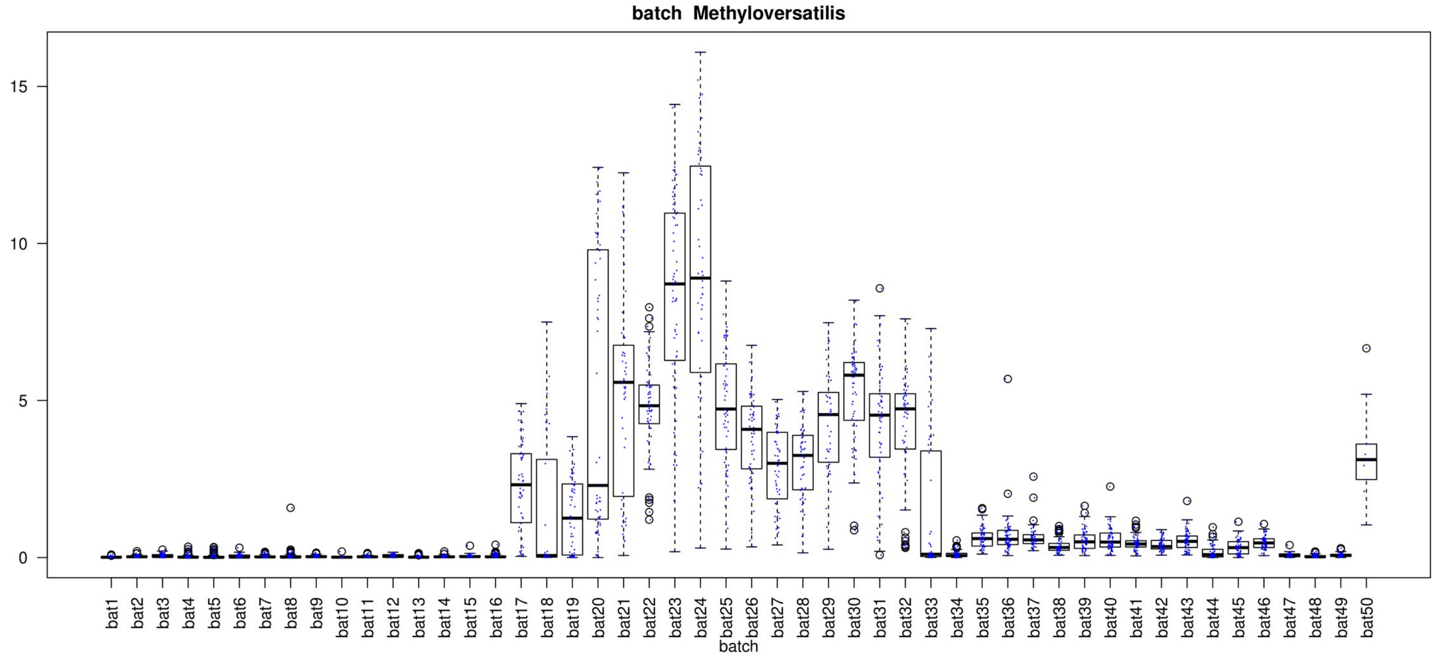

Figure S6. Box plots of inferred abundance values for the genus *Methyloversatilis* for each processing batch (created using the statistical environment R [12])

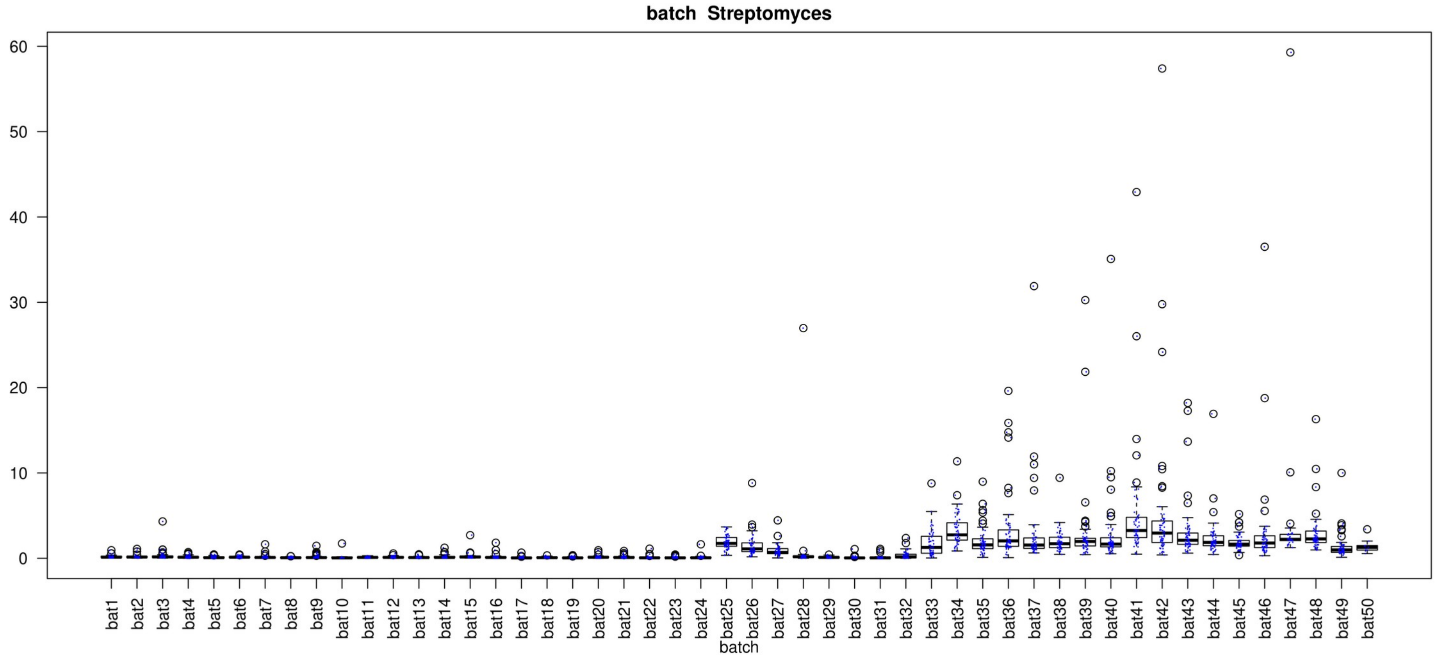

Figure S7. Box plots of inferred abundance values for the genus *Streptomyces* for each processing batch (created using the statistical environment R [12])

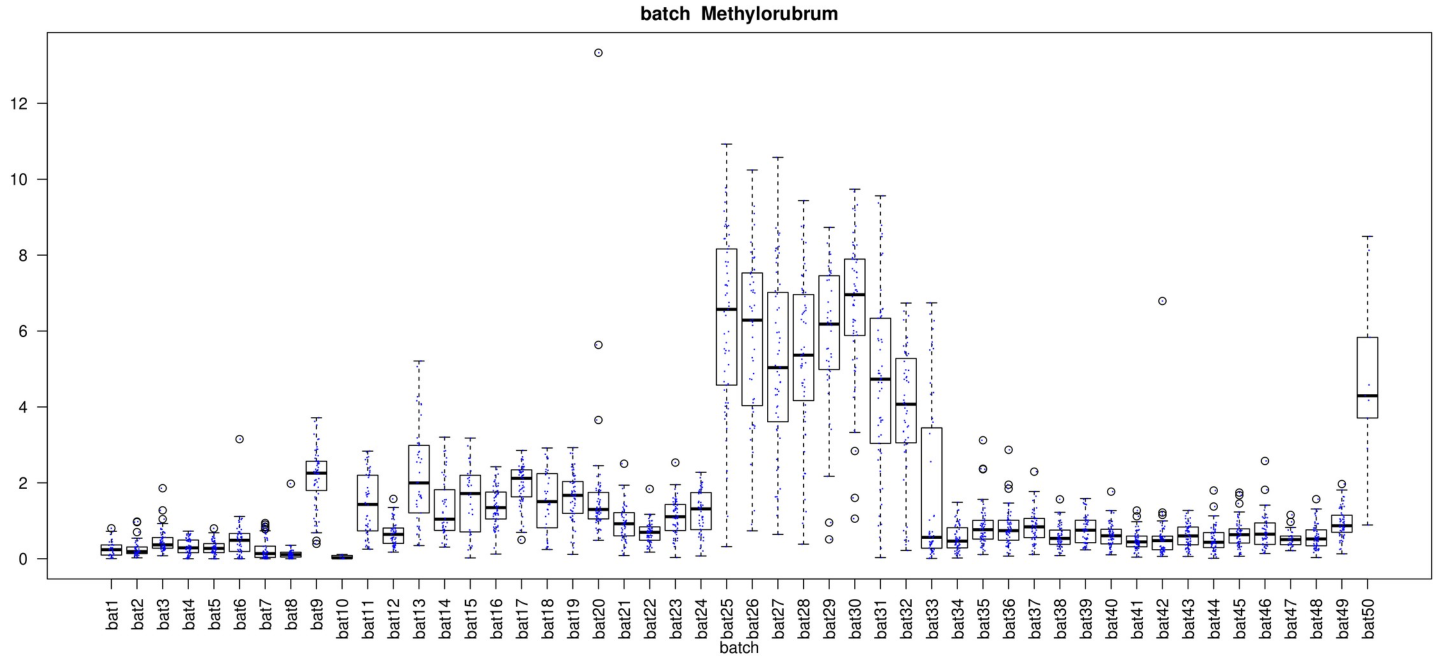

Figure S8. Box plots of inferred abundance values for the genus *Methylorubrum* for each processing batch (created using the statistical environment R [12])

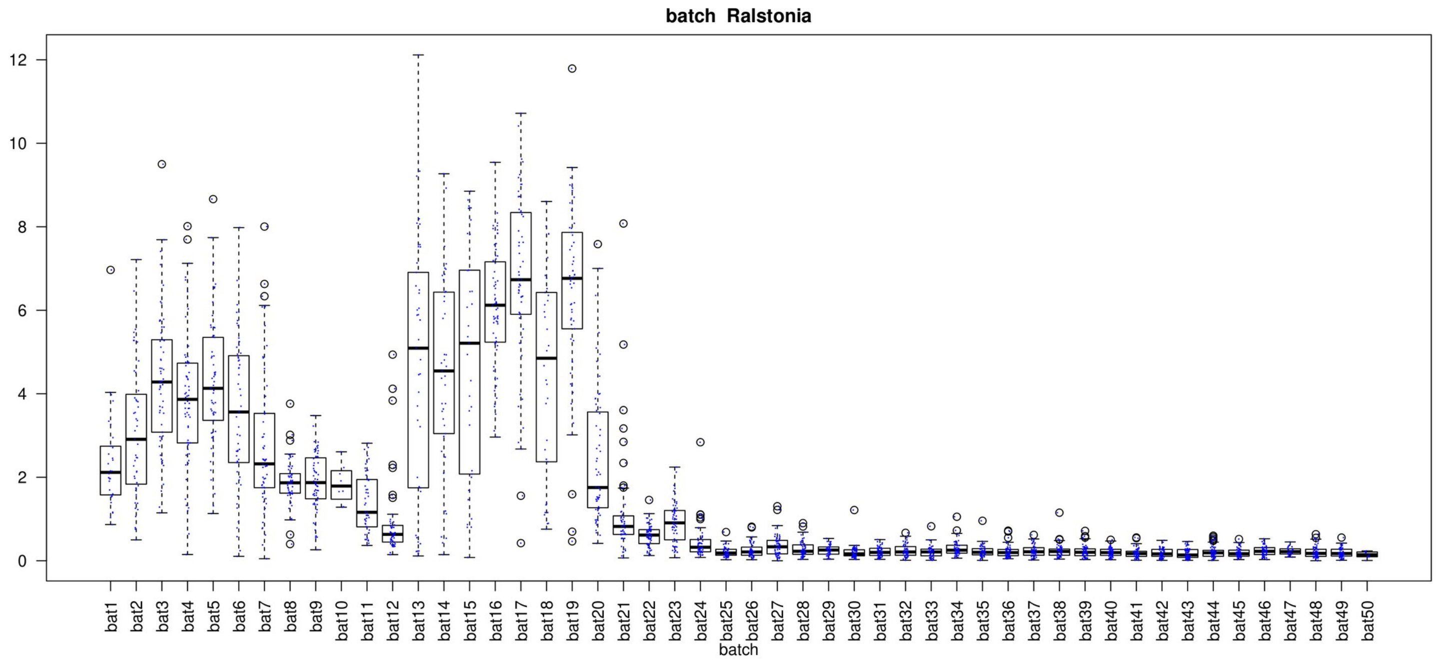

Figure S9. Box plots of inferred abundance values for the genus *Ralstonia* for each processing batch (created using the statistical environment R [12])

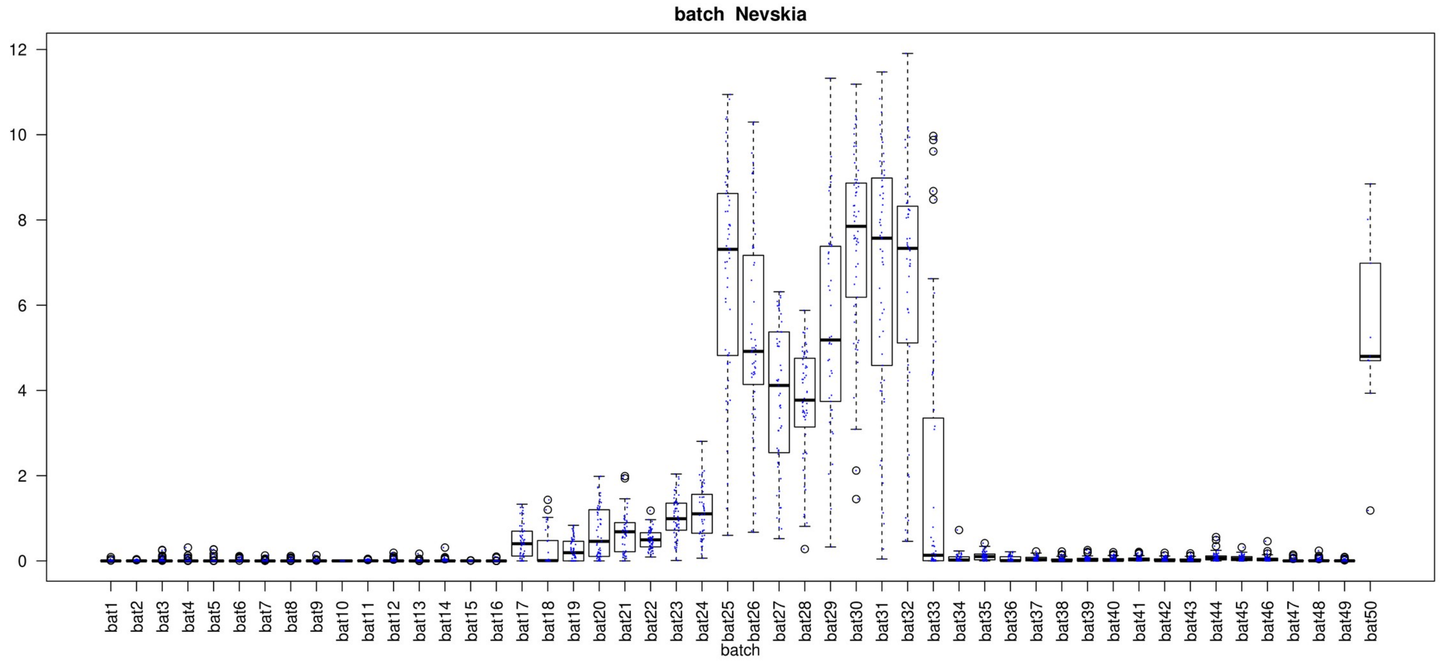

Figure S10. Box plots of inferred abundance values for the genus *Nevskia* for each processing batch (created using the statistical environment R [12])

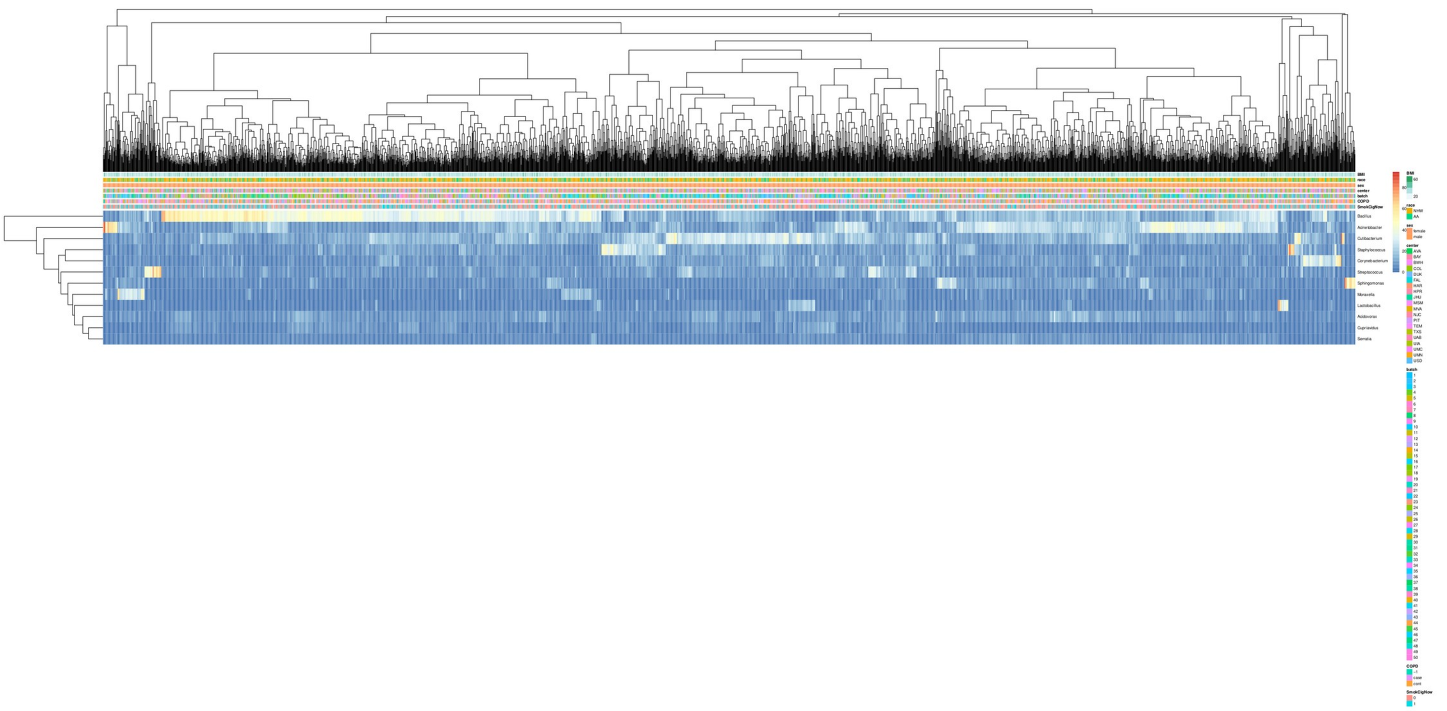

Figure S11. Heatmap of the normalized scores at the genus level, with clustering of samples in the columns by Bray-Curtis dissimilarity. Tracks are included for BMI, race, sex, batch, study center, COPD status and smoking status (created using the R package pheatmap [9]).

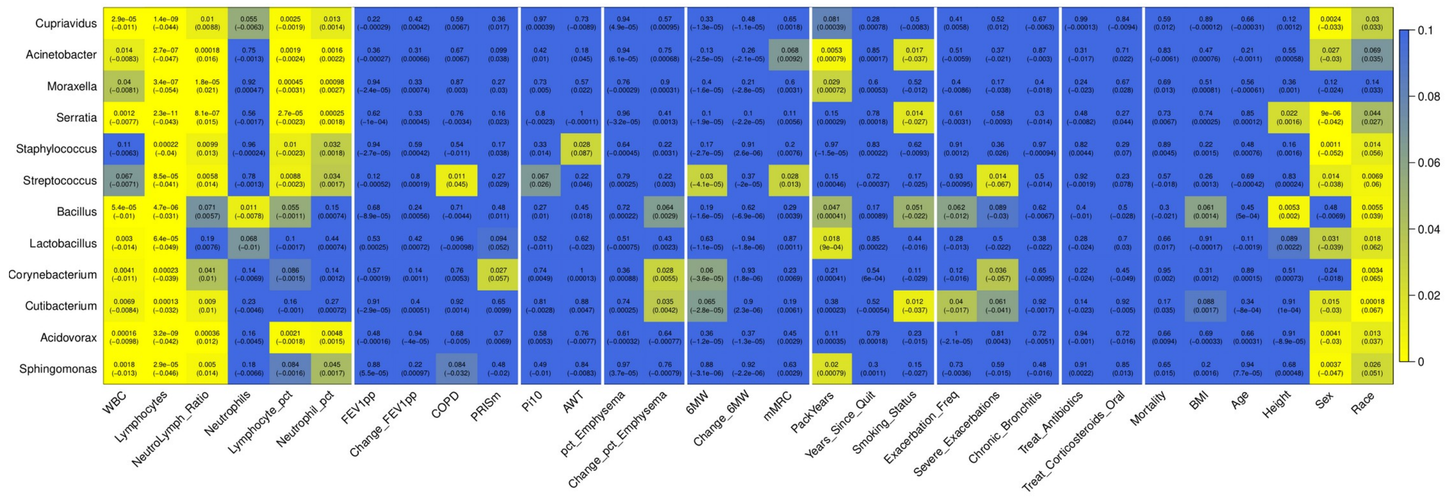

Figure S12. Heatmap of the associations between genera inferred abundance and host-related variables in the primary analysis. Top entry in each cell is the p-value and the bottom is the effect estimate from the MaAsLin2 model; color scale provided for p-values (created using the labeledHeatmap function from the R package WGCNA [13]).

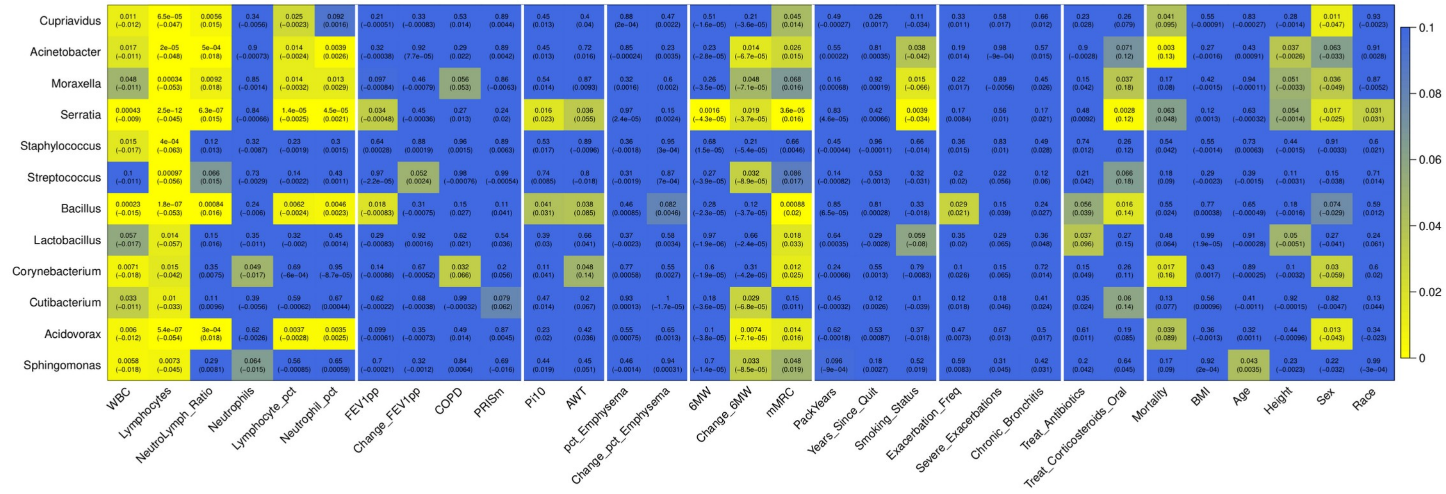

Figure S13. Heatmap of the associations between genera inferred abundance and host-related variables for the replication analysis in the second independent set of data. Top entry in each cell is the p-value and the bottom is the effect estimate from the MaAsLin2 model; color scale provided for p-values (created using the labeledHeatmap function from the R package WGCNA [13]).

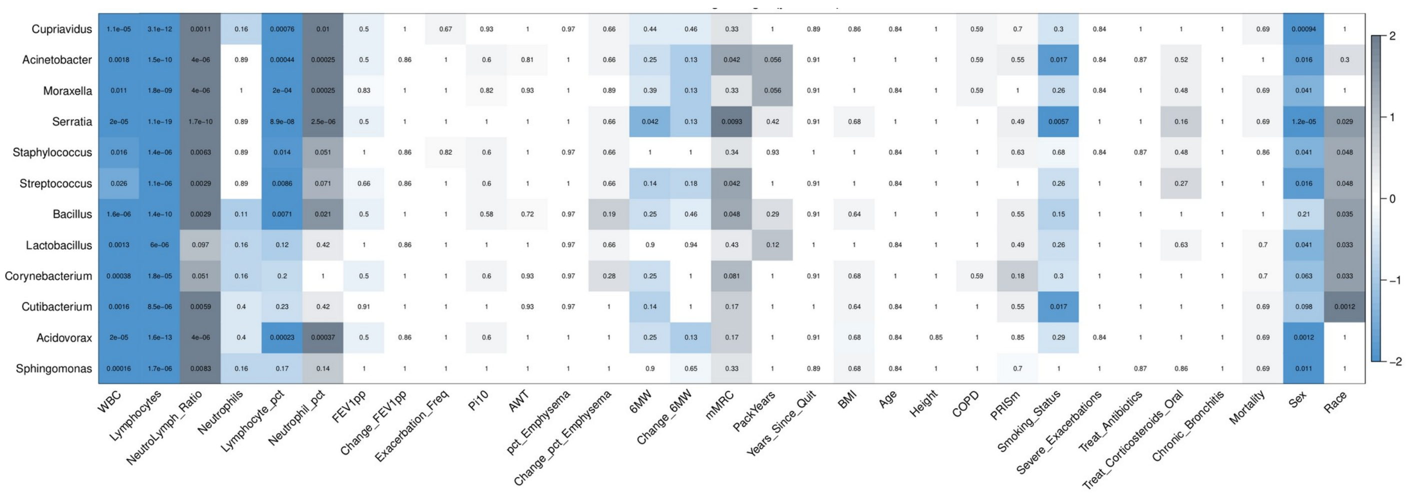

Figure S14. Heatmap of the associations between genera inferred abundance and host-related variables for the meta-analysis. Entry in each cell is the adjusted q-value. The color scale is provided for effect.sign * (-log10(q−values)), with intensity proportional to significance and gray representing positive correlation and blue representing negative correlation. Results with discordant directions of effect are set to q=1 (white) (created using the labeledHeatmap function from the R package WGCNA [13]).

Figure S15. Plots of the model residuals of the inferred TMM abundance for the significant (FDR < 5%) meta-analysis findings in the primary set of data to illustrate the relationships between taxa abundance and the variables of interest. (created using the statistical environment R [12])

See file: Supplemental_Figure_S15.pdf

Figure S16. Plots of the model residuals of the inferred TMM abundance for the significant (FDR < 5%) meta-analysis findings in the replication set of data to illustrate the relationships between taxa abundance and the variables of interest. (created using the statistical environment R [12])

See file: Supplemental_Figure_S16.pdf

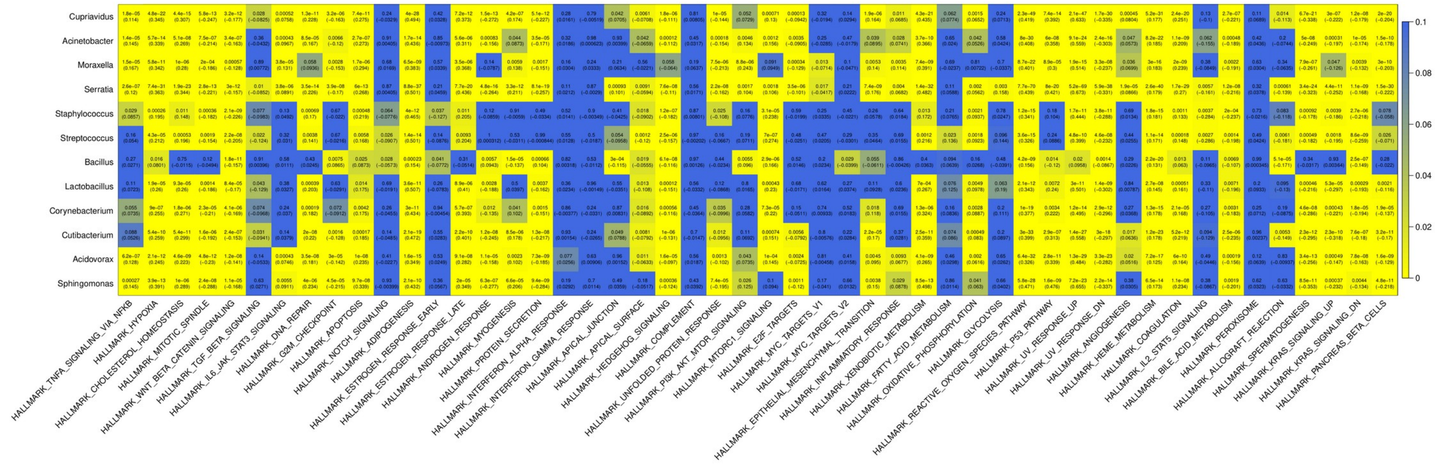

Figure S17. Heatmap of the associations between genera inferred relative abundance and Hallmark host pathways in the primary analysis. Top entry in each cell is the p-value and the bottom is the effect estimate from the MaAsLin2 model; color scale provided for p-values (created using the labeledHeatmap function from the R package WGCNA [13]).

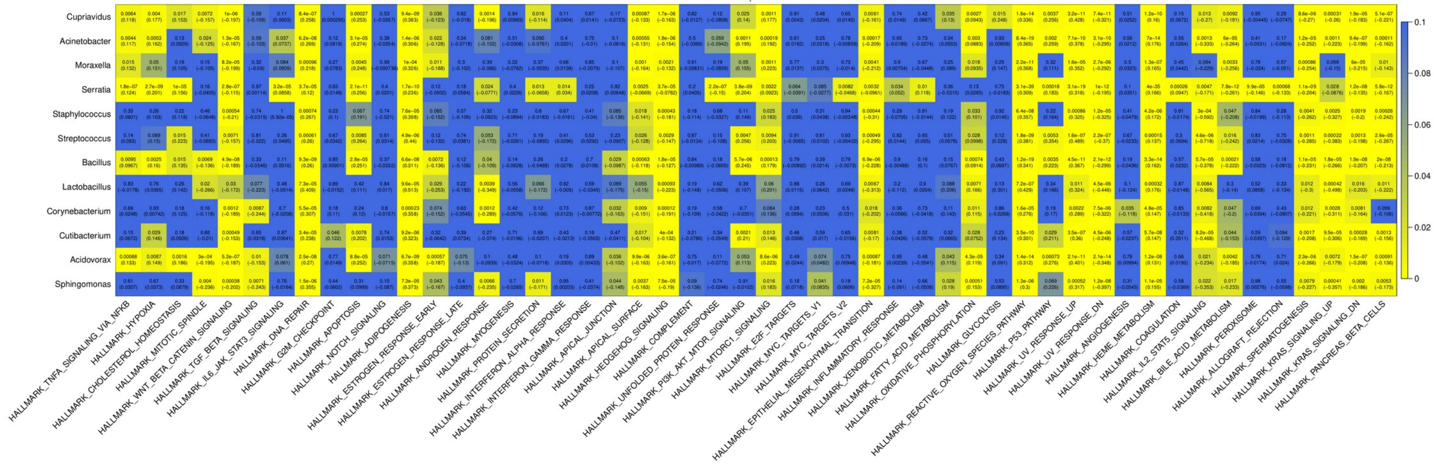

Figure S18. Heatmap of the associations between genera inferred relative abundance and Hallmark host pathways in the replication analysis in a second set of data. Top entry in each cell is the p-value and the bottom is the effect estimate from the MaAsLin2 model; color scale provided for p-values (created using the labeledHeatmap function from the R package WGCNA [13]).

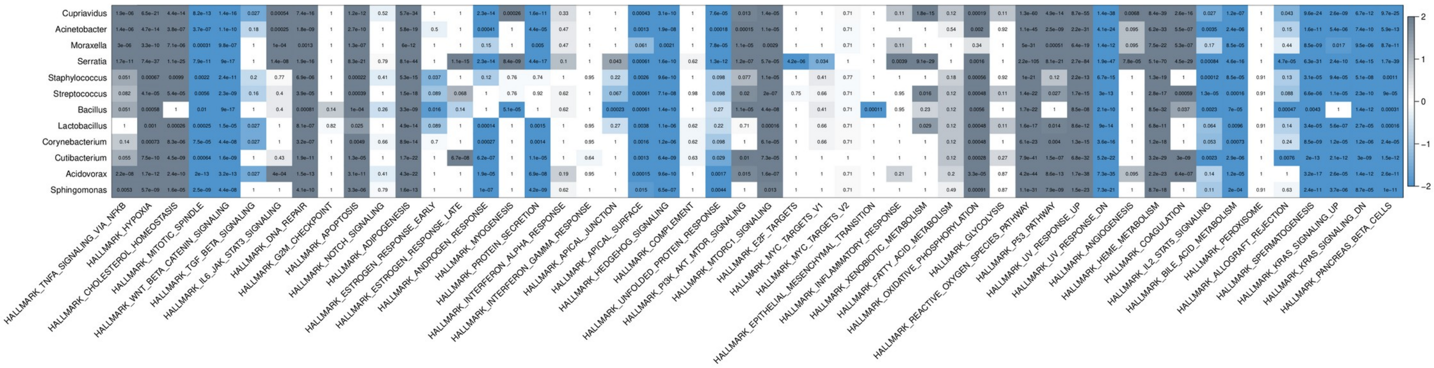

Figure S19. Heatmap of the associations between genera inferred relative abundance and Hallmark host pathways in the meta-analysis. Entry in each cell is the adjusted q-value. The color scale is provided for effect.sign * (-log10(q−values)), with intensity proportional to significance and gray representing positive correlation and blue representing negative correlation. Results with discordant directions of effect are set to q=1 (white) (created using the labeledHeatmap function from the R package WGCNA [13]).

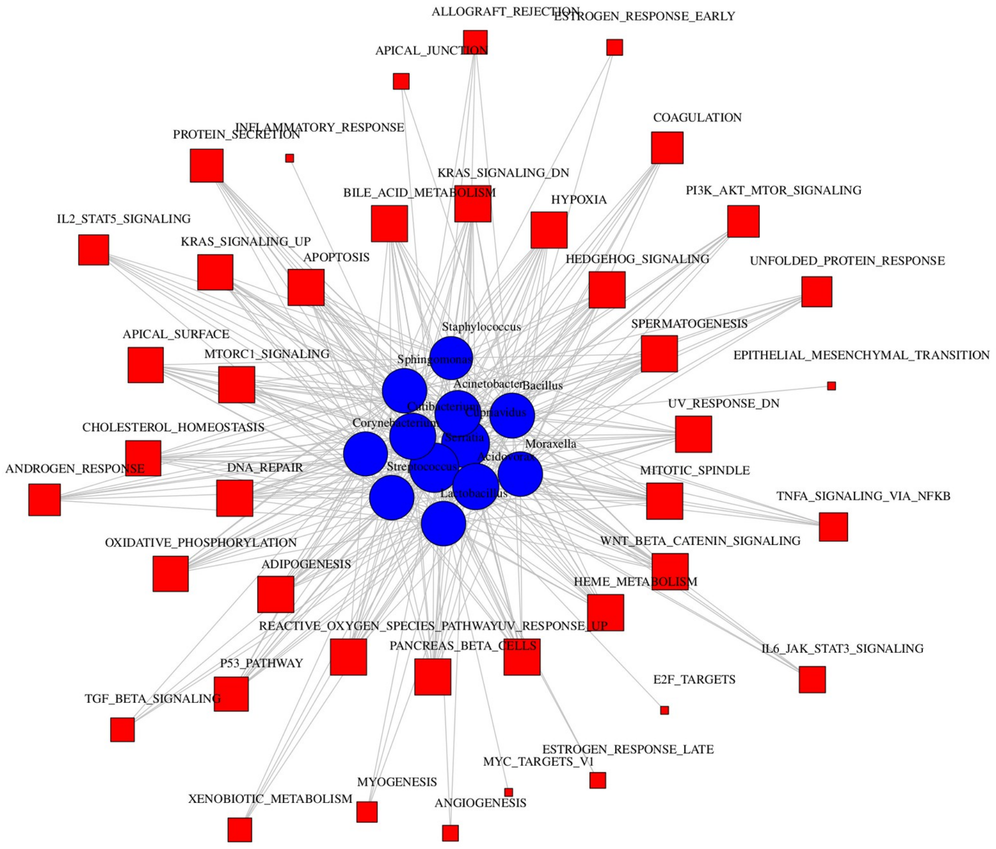

Figure S20. Bipartite network from the host-microbiome interaction analysis. Edges represent a significant (FDR < 5%) association in the meta-analysis between inferred genus abundance and the expression of the Hallmark pathway in the human host. The red squares represent Hallmark pathways from MSigDB and the blue circles represent genera (created using the R package igraph [14]).

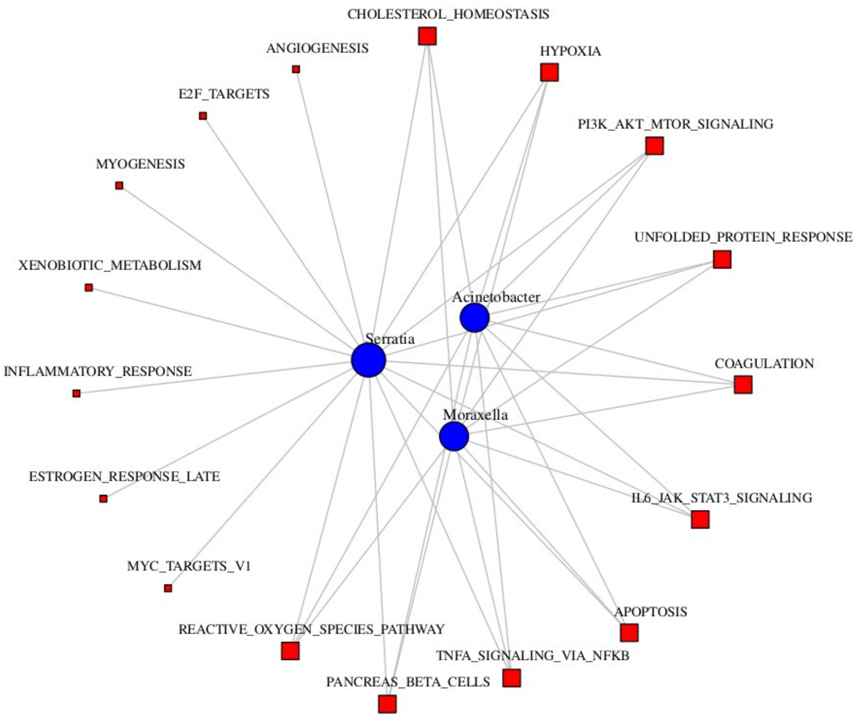

Figure S21. Community from the bipartite network from the host-microbiome interaction analysis. Edges represent a significant (FDR < 5%) association between inferred genus abundance and the expression of the Hallmark pathway in the human host in the meta-analysis. The red squares represent Hallmark pathways from MSigDB and the blue circles represent genera (created using the R package igraph [14]).

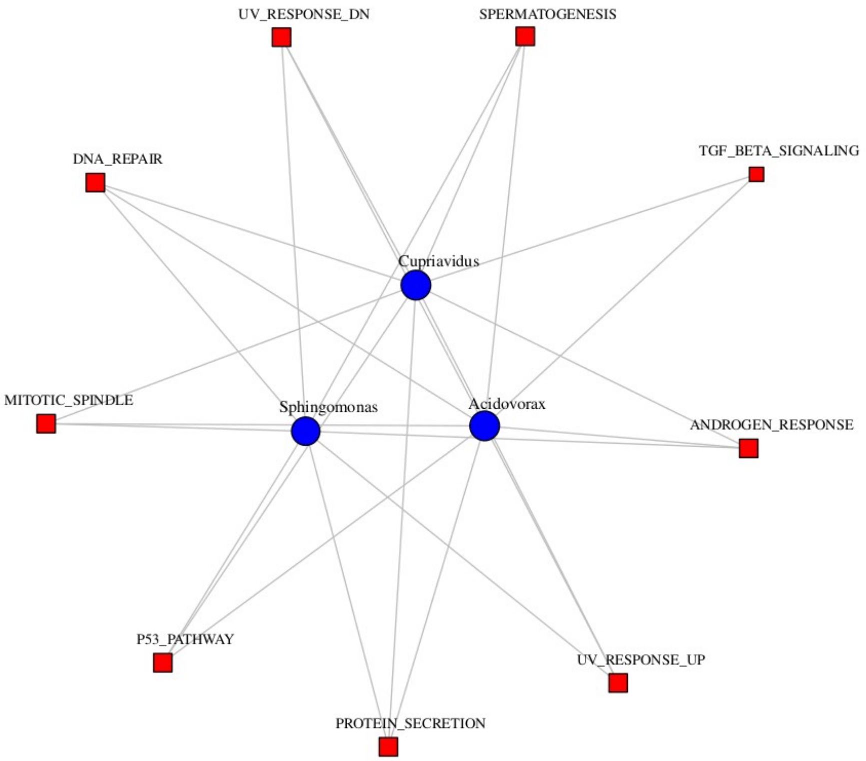

Figure S22. Community from the bipartite network from the host-microbiome interaction analysis. Edges represent a significant (FDR < 5%) association between inferred genus abundance and the expression of the Hallmark pathway in the human host in the meta-analysis. The red squares represent Hallmark pathways from MSigDB and the blue circles represent genera (created using the R package igraph [14]).

Supplemental Tables

Table S1. Read count summary for RNA-seq gene expression and microbiome signature detection

RNA-seq Mapping target	Primary data – read counts			Replication data – read counts		
	Minimum	Median	Maximum	Minimum	Median	Maximum
Summary of mean counts for all genes	45	111	542	44	145	546
Cupriavidus	0	44	3,125	1	39	2,965
Acinetobacter	2	98	101,060	5	163	24,976
Moraxella	0	31	15,773	1	34	6,031
Serratia	2	78	14,285	5	530	5,876
Staphylococcus	0	49	16,121	2	50	51,211
Streptococcus	0	42	50,377	0	43	6,412
Bacillus	4	159	8,383	3	121	4,344
Lactobacillus	0	23	6,657	0	18	9,058
Corynebacterium	0	50	7,475	1	51	8,577
Cutibacterium	2	97	13,422	9	149	13,531
Acidovorax	9	138	31,612	12	148	7,394
Sphingomonas	0	40	7,859	2	46	29,401

RNA-seq = RNA-sequencing, primary data N = 2590, secondary data N = 1065

Table S2. Models for microbial taxon associations with outcomes of interest (inferred taxonomic abundance is the outcome in each model)

**Predictor variable of interest**	**Model**
White blood cell count	~ WBC + Age + Sex + Race + Smoking_Status + PackYears + batch + center
Lymphocyte count	~ Lymphocytes + Age + Sex + Race + Smoking_Status + PackYears + batch + center
Neutrophil to lymphocytes ratio	~ NeutroLymph_Ratio + Age + Sex + Race + Smoking_Status + PackYears + batch + center
Neutrophil count	~ Neutrophils + Age + Sex + Race + Smoking_Status + PackYears + batch + center
Lymphocyte percentage	~ Lymphocyte_pct + Age + Sex + Race + Smoking_Status + PackYears + batch + center
Neutrophil percentage	~ Neutrophil_pct + Age + Sex + Race + Smoking_Status + PackYears + batch + center
FEV1 % predicted	~ FEV1pp + Age + Sex + Race + Smoking_Status + PackYears + batch + center
Change in FEV1 % predicted [#]	~ Change_FEV1pp + Age + Sex + Race + Smoking_Status + PackYears + batch + center
COPD: case-control [*]	~ COPD + Age + Sex + Race + Smoking_Status + PackYears + batch + center
PRISm-control [@]	~ PRISm + Age + Sex + Race + Smoking_Status + PackYears + batch + center
Pi10	~ Pi10 + Age + Sex + Race + Smoking_Status + PackYears + batch + center
Airway Wall Thickness	~ AWT + Age + Sex + Race + Smoking_Status + PackYears + batch + center
Percent Emphysema	~ pctEmphysema + Age + Sex + Race + Smoking_Status + BMI + PackYears + batch + center
Change in Percent Emphysema [#]	~ Change_pctEmphysema + Age + Sex + Race + Smoking_Status + BMI + PackYears + batch + center
6-minute walk distance (ft)	~ 6MW + Age + Sex + Race + Smoking_Status + PackYears + batch + center
Change in 6-minute walk distance (ft) [#]	~ Change_6MW + Age + Sex + Race + Smoking_Status + PackYears + batch + center
MMRC dyspnea score	~ mMRC + Age + Sex + Race + Smoking_Status + PackYears + batch + center
Pack years of smoking	~ PackYears + Age + Sex + Race + Smoking_Status + batch + center
Years since quit smoking	~ Years_Since_Quit + Age + Sex + Race + PackYears + batch + center
Smoking status	~ Smoking_Status + Age + Sex + Race + PackYears + batch + center
Exacerbation Frequency	~ Exacerbation_Frequency + Age + Sex + Race + Smoking_Status + PackYears + batch + center
Severe Exacerbations (yes / no)	~ Severe_Exacerbations + Age + Sex + Race + Smoking_Status + PackYears + batch + center
Chronic Bronchitis (yes / no)	~ Chronic_Bronchitis + Age + Sex + Race + Smoking_Status + PackYears + batch + center
Treated with antibiotics (yes / no)	~ Treat_Antibiotics + Age + Sex + Race + Smoking_Status + PackYears + batch + center
Treated with oral corticosteroids (yes / no)	~ Treat_Corticosteroids_Oral + Age + Sex + Race + Smoking_Status + PackYears + batch + center
Status (alive / diseased)	~ Mortality + Age + Sex + Race + Smoking_Status + PackYears + batch + center
Body mass index (kg/m^2)	~ BMI + Age + Sex + Race + Smoking_Status + PackYears + batch + center
Age (years)	~ Age + Sex + Race + Smoking_Status + PackYears + batch + center
Height (cm)	~ Height_CM + Age + Sex + Race + Smoking_Status + PackYears + batch + center
Sex	~ Sex + Age + Race + Smoking_Status + PackYears + batch + center
Race	~ Race + Age + Sex + Smoking_Status + PackYears + batch + center
Smoking_Status (ordinal variable): 0 former smoker, 1 current smoker	

Abbreviations: FEV1=forced expiratory volume in 1 sec; pctEmph=% emphysema; Pi10=SRWA-Pi10=square root wall area of a hypothetical airway with 10mm internal perimeter; AWT=airway wall thickness; batch=processing batch variable; center=study center

* PRISm subjects excluded

@ COPD cases (GOLD = 1,2,3,4) excluded

Change variables reflect COPDGene Phase 1 visit to Phase 2 visit

Table S3. COPDGene study subjects from second independent set

Demographics	N = 1065<br>Mean ± sd or distribution
Age, years	65.3 ± 9.0
Sex (Female / Male)	525 / 540
Race (Non-Hispanic White / African American)	705 / 360
Smoking status (Current / Former) (n = 1058)	445 / 613
Smoking History, pack-years (n = 1060)	43.9 ± 25.1
GOLD stage (n = 1050)<br>4<br>3<br>2<br>1<br>Control<br>PRISm *	 44<br>108<br>183<br>109<br>470<br>136
FEV_1 % predicted (n = 1050)	78.7 ± 24.6
FEV_1 / FVC (n = 1050)	0.68 ± 0.14
Percent emphysema at -950HU (n = 976)	5.0 ± 8.4
Body mass index kg/m^2 (n = 1061)	28.8 ± 6.3
Airway wall thickness, segmental bronchi (n = 976)	1.04 ± 0.22
Severe exacerbation in the year prior ** (no / yes) (n = 1058)	951 / 107
Treated with chronic oral corticosteroids (no / yes) (n = 1047)	1027 / 20
Survival (alive / deceased) ***	1018 / 47
MMRC dyspnea score (n = 1059)<br>0<br>1<br>2<br>3<br>4	 537<br>124<br>137<br>179<br>82
6-minute walk distance ft (n = 1028)	1302 ± 426
Comorbidity score **** (range 0 to 14) (n = 1059)	2.84 ± 1.95

Abbreviations: FEV1=forced expiratory volume in 1 sec; FVC= forced vital capacity; PRISm = Preserved Ratio Impaired Spirometry; mMRC=Modified Medical Research Council dyspnea score

* PRISm (FEV1<80% predicted with FEV1/FVC≥0.7) [15]

** Emergency department or hospital admission

*** Survival status as of October 2018

**** Sum of comorbidities reported, considering Coronary Heart disease, Diabetes, Congestive heart failure, Stroke, Osteoarthritis, Osteoporosis, Hypertension, High cholesterol, Gastroesophageal reflux disease, Stomach ulcers, Obesity, Sleep apnea, Hay fever, Peripheral Vascular Disease [16].

References

1. Parker MM, Chase RP, Lamb A, Reyes A, Saferali A, Yun JH, et al. RNA sequencing identifies novel non-coding RNA and exon-specific effects associated with cigarette smoking. BMC Med Genomics. 2017;10:58.

2. Andrews S. Fastqc: A Quality Control Tool For High Throughput Sequence Data. [Internet]. 2010 [cited 2016 May 1]. Available from: https://www.bioinformatics.babraham.ac.uk/projects/fastqc/

3. DeLuca DS, Levin JZ, Sivachenko A, Fennell T, Nazaire M-D, Williams C, et al. RNA-SeQC: RNA-seq metrics for quality control and process optimization. Bioinformatics. 2012;28:1530–2.

4. Jiang H, Lei R, Ding S-W, Zhu S. Skewer: a fast and accurate adapter trimmer for next-generation sequencing paired-end reads. BMC Bioinformatics. 2014;15:182.

5. Dobin A, Davis CA, Schlesinger F, Drenkow J, Zaleski C, Jha S, et al. STAR: ultrafast universal RNA-seq aligner. Bioinformatics. 2013;29:15–21.

6. Liao Y, Smyth GK, Shi W. The Subread aligner: fast, accurate and scalable read mapping by seed-and-vote. Nucleic Acids Res. 2013;41:e108–e108.

7. Kersey PJ, Allen JE, Armean I, Boddu S, Bolt BJ, Carvalho-Silva D, et al. Ensembl Genomes 2016: more genomes, more complexity. Nucleic Acids Res. 2016;44:D574–80.

8. Liberzon A, Birger C, Thorvaldsdóttir H, Ghandi M, Mesirov JP, Tamayo P. The Molecular Signatures Database Hallmark Gene Set Collection. Cell Syst. 2015;1:417–25.

9. Kolde R. pheatmap: Pretty Heatmaps [Internet]. 2019. Available from: https://CRAN.R-project.org/package=pheatmap

10. Oksanen J, Blanchet FG, Friendly M, Kindt R, Legendre P, McGlinn D, et al. vegan: Community Ecology Package [Internet]. 2019. Available from: https://CRAN.R-project.org/package=vegan

11. Wickham H. ggplot2: Elegant Graphics for Data Analysis [Internet]. Springer-Verlag New York; 2016. Available from: https://ggplot2.tidyverse.org

12. R Core Team. R: A Language and Environment for Statistical Computing [Internet]. Vienna, Austria: R Foundation for Statistical Computing; 2020. Available from: http://www.r-project.org

13. Langfelder P, Horvath S. WGCNA: an R package for weighted correlation network analysis. BMC Bioinformatics. 2008;9:559.

14. Csardi G, Nepusz T. The igraph software package for complex network research. InterJournal. 2006;Complex Systems:1695.

15. William W. Stringer MD, Janos Porszasz MD, Surya P. Bhatt MD, Barry J. Make MD, Meredith C. McCormack MD, Richard Casaburi P. Physiologic Insights from the COPD Genetic Epidemiology Study. Chronic Obstr Pulm Dis COPD Found. 6:256–66.

16. Putcha N, Puhan MA, Drummond MB, Han MK, Regan EA, Hanania NA, et al. A Simplified Score to Quantify Comorbidity in COPD. PLOS ONE. Public Library of Science; 2014;9:e114438.
