# Supplementary material for: Peripheral blood microbial signatures in current and former smokers
Source: Sci Rep. 2021 Oct 6;11:19875. doi: 10.1038/s41598-021-99238-4 (PMC8494912; doi:10.1038/s41598-021-99238-4)
Supplement: Supplementary file 2 — Supplementary Information 2. [file 41598_2021_99238_MOESM2_ESM.pdf]

Plot of model residuals

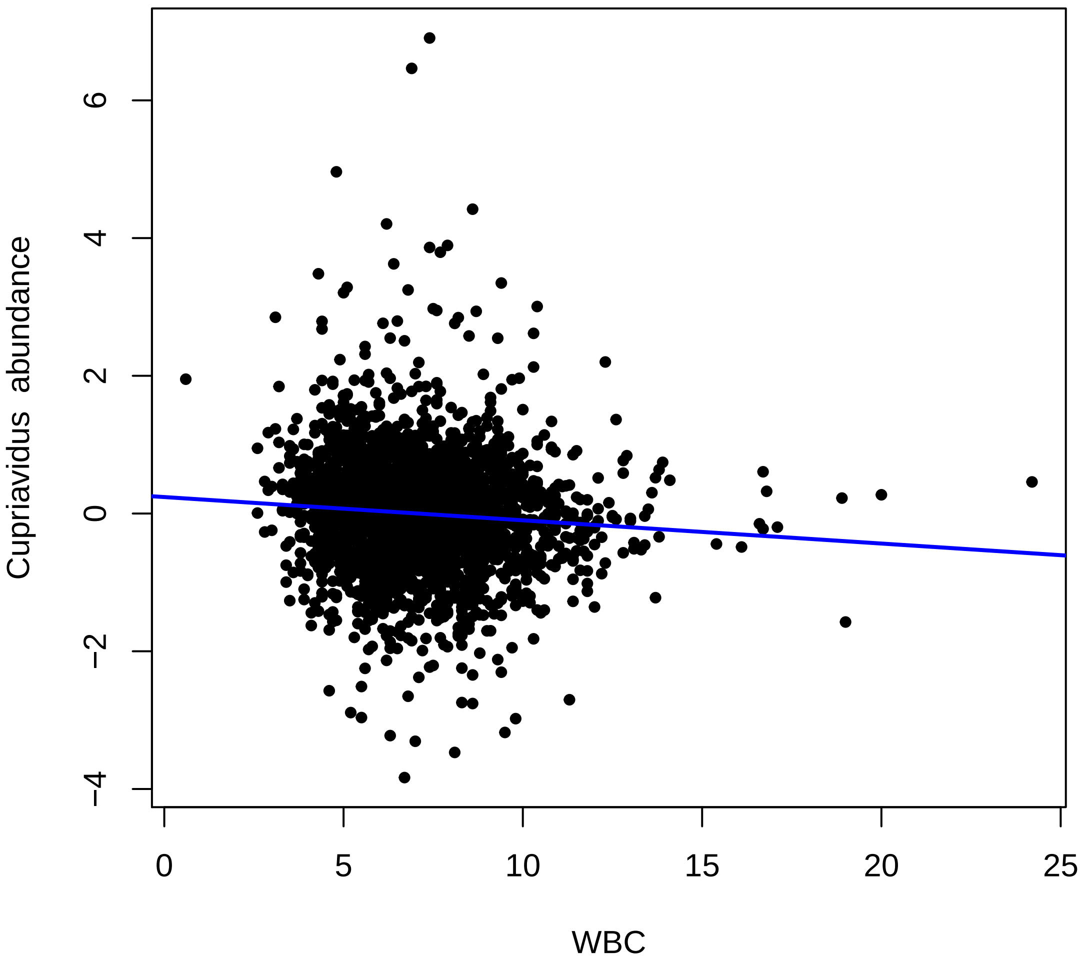

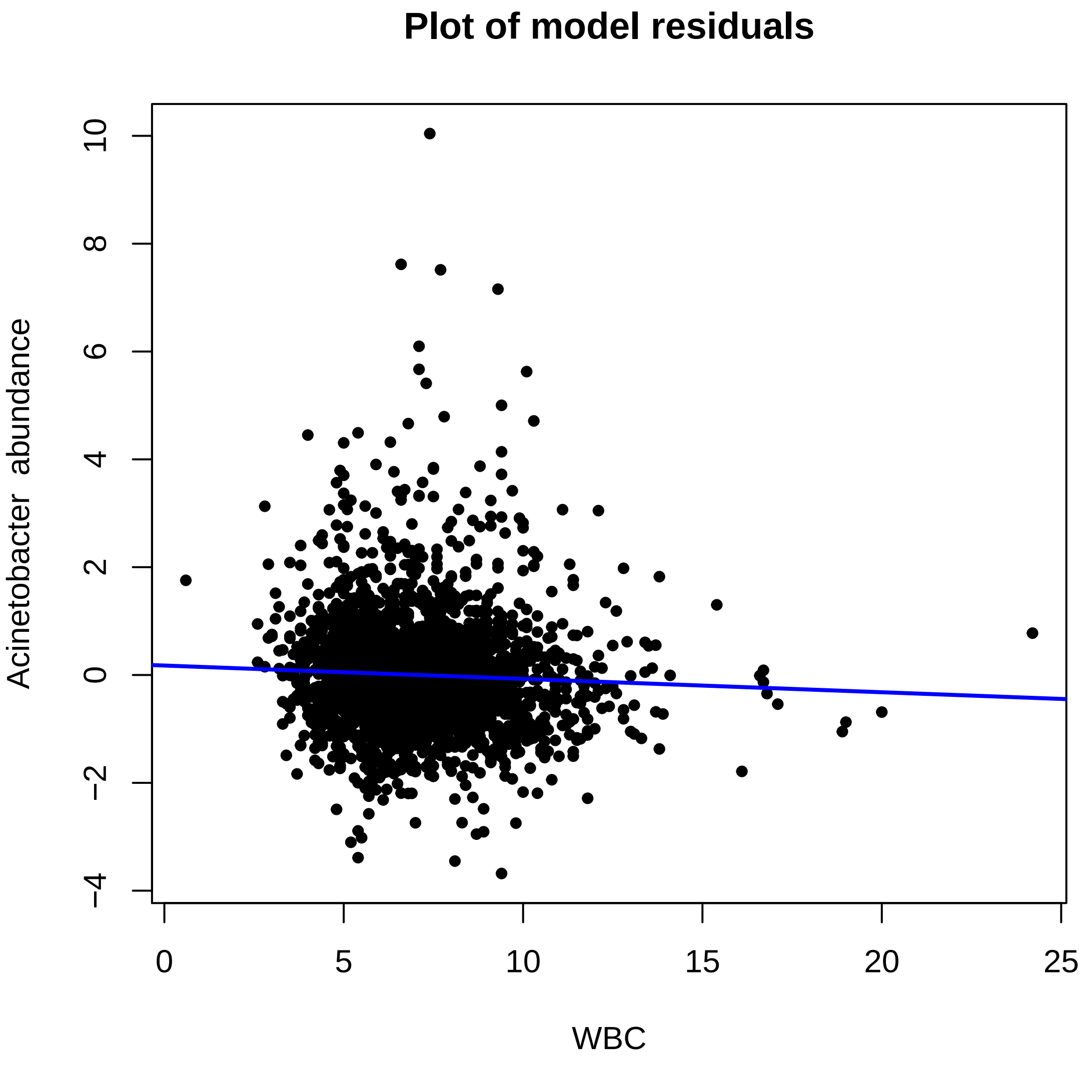
Plot of model residuals
Acinetobacter abundance
WBC
−4
−2
0
2
4
6
8
10
0
5
10
15
20
25

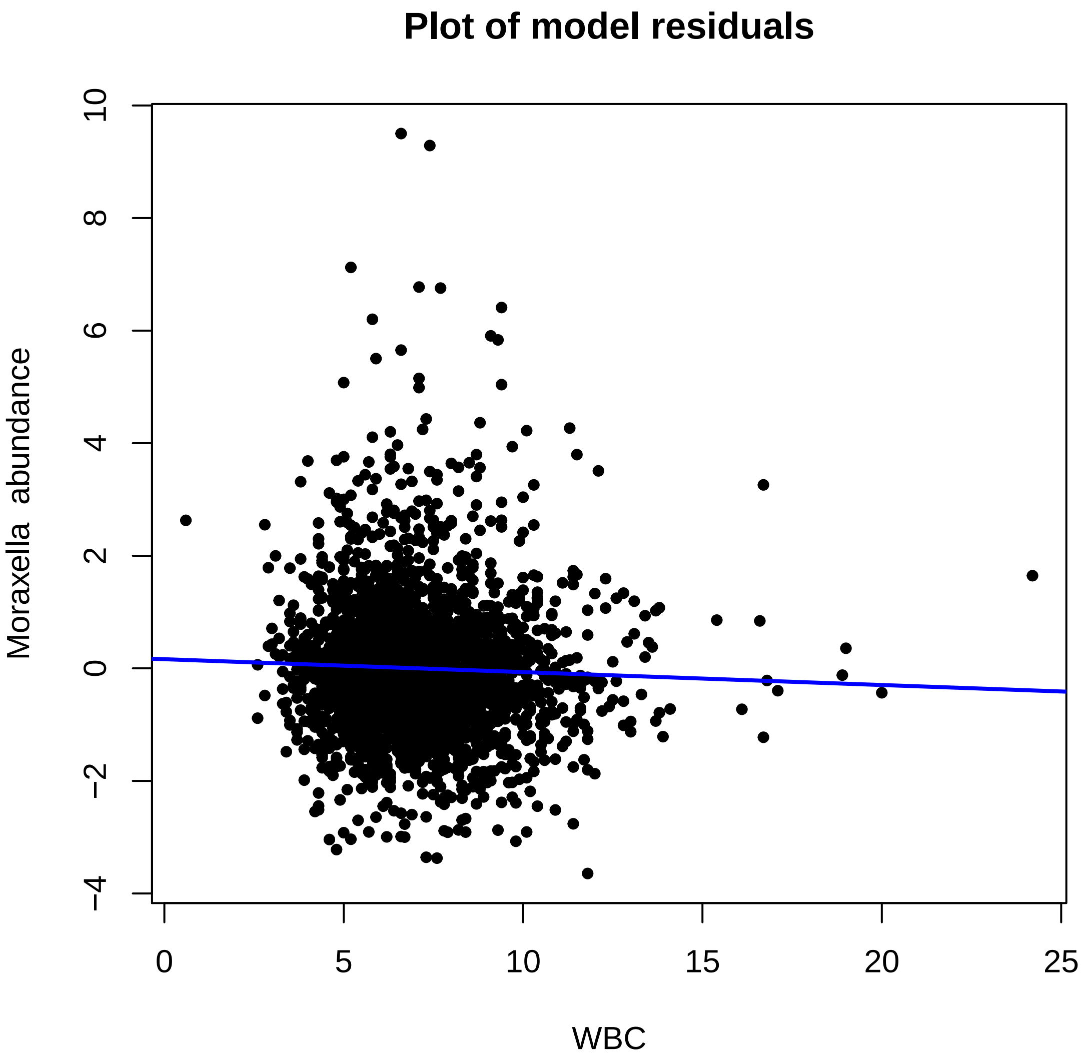

Plot of model residuals
Moraxella abundance
WBC
10
8
6
4
2
0
−2
−4
0
5
10
15
20
25

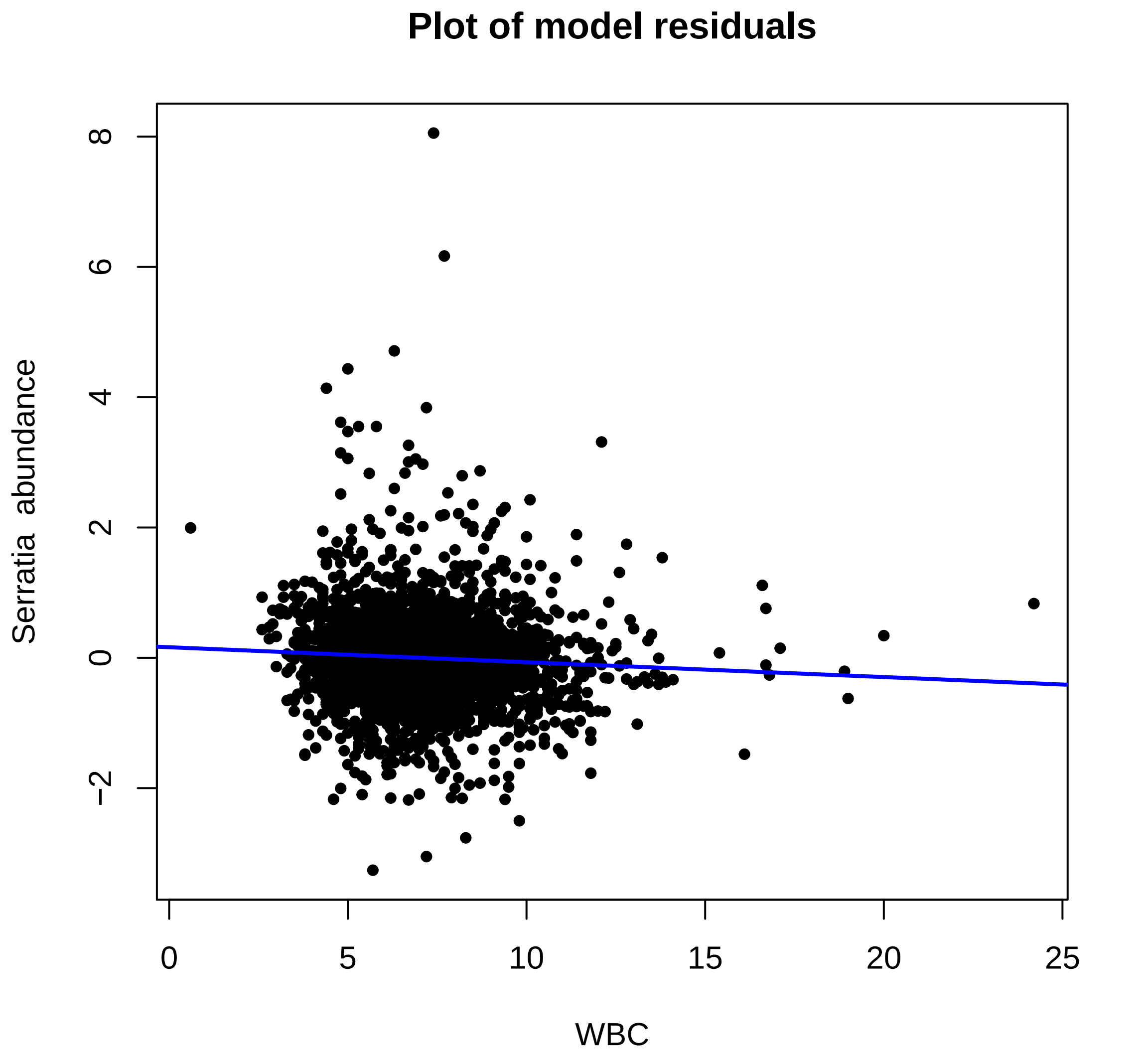
Plot of model residuals
Serratia abundance
WBC
8
6
4
2
0
−2
0
5
10
15
20
25

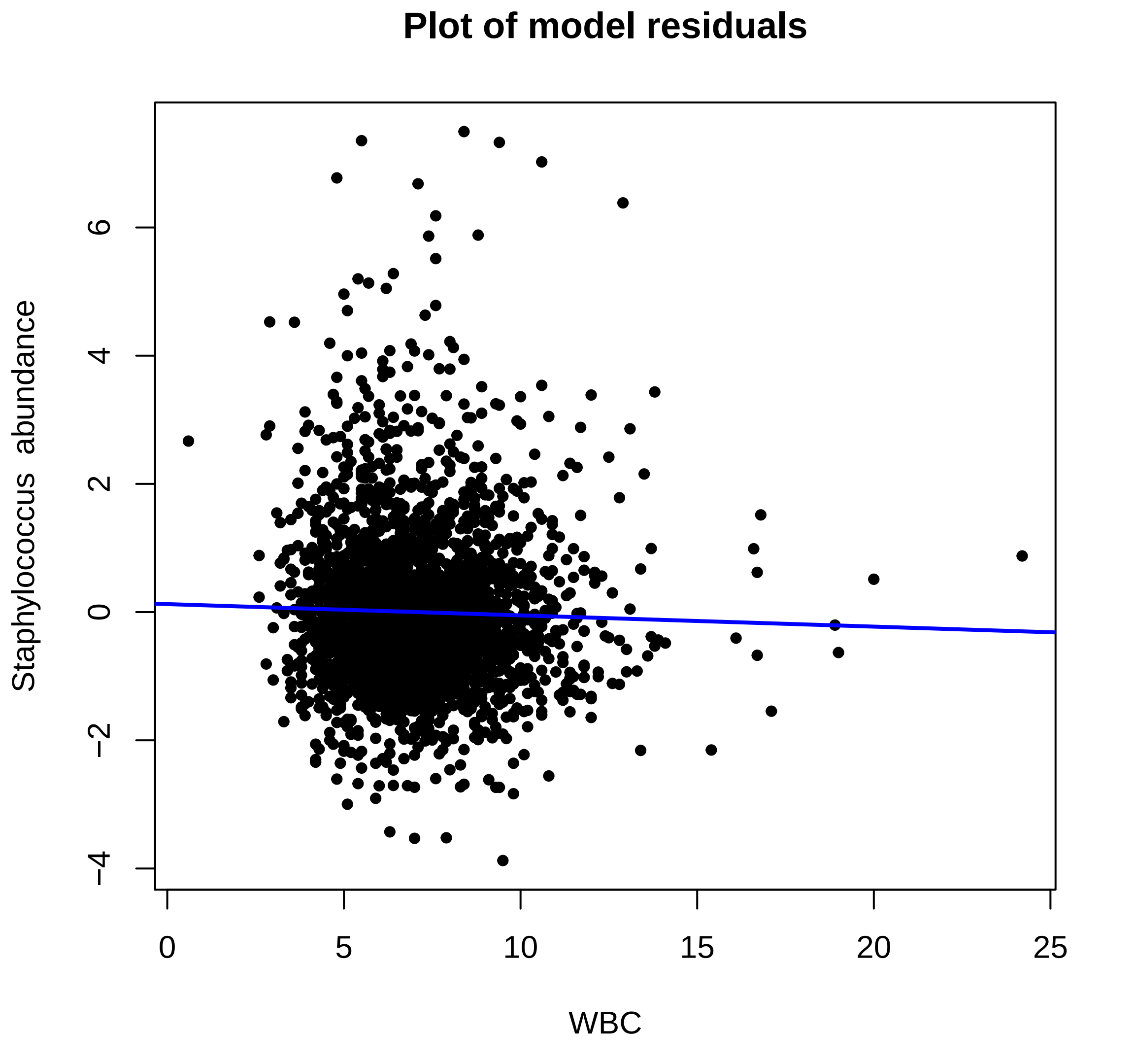
Plot of model residuals
Staphylococcus abundance
WBC
−4
−2
0
2
4
6
0
5
10
15
20
25

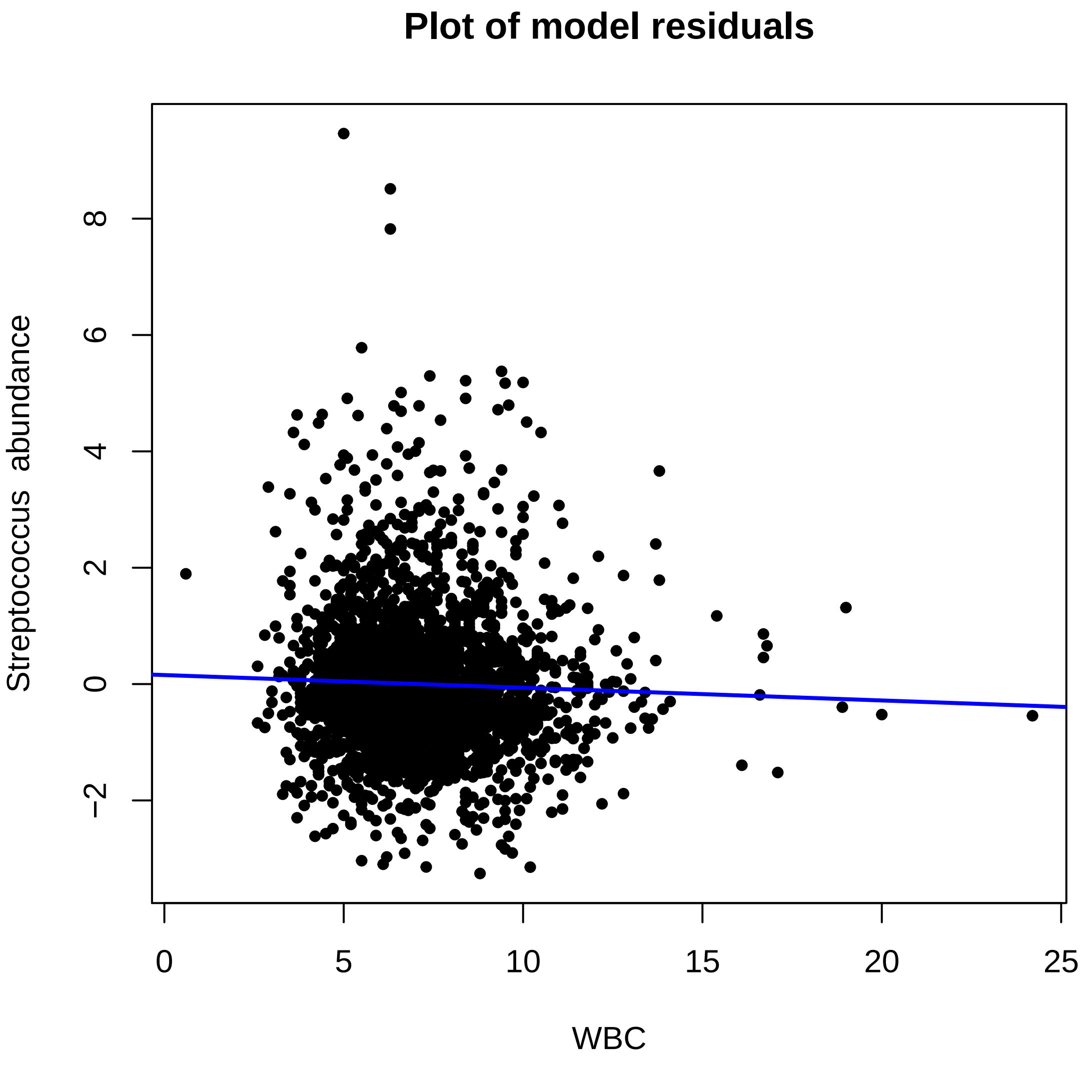
Plot of model residuals
Streptococcus abundance
WBC
8
6
4
2
0
−2
0
5
10
15
20
25

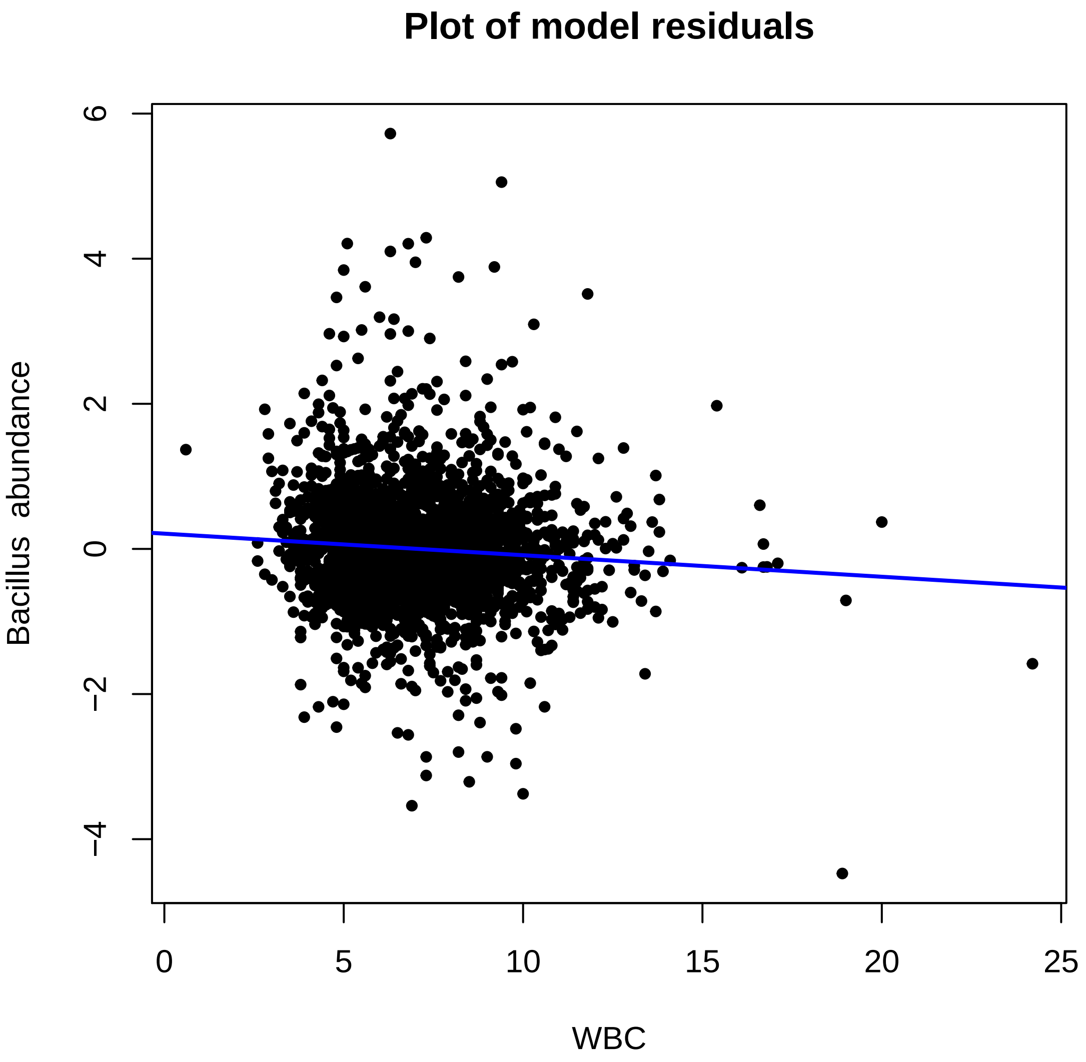

Plot of model residuals
Bacillus abundance
WBC
6
4
2
0
−2
−4
0
5
10
15
20
25

Plot of model residuals

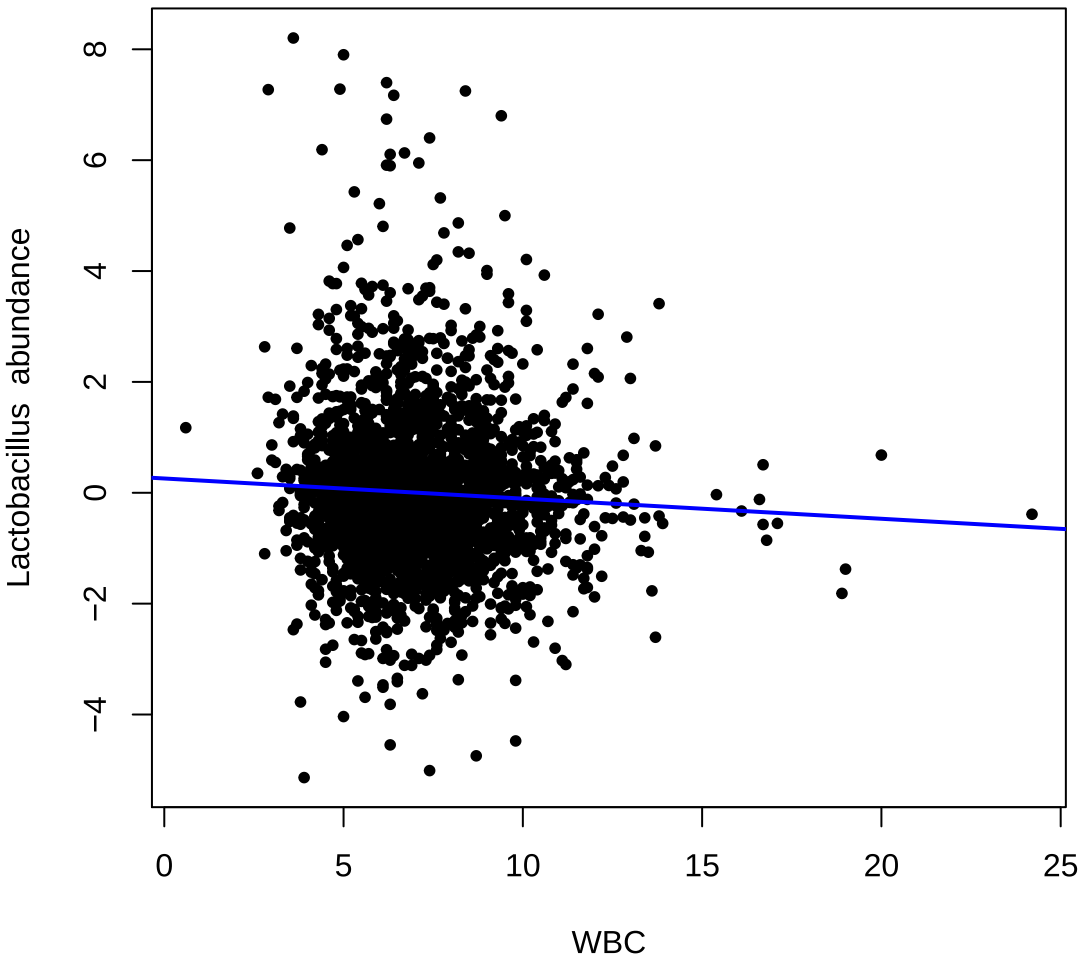

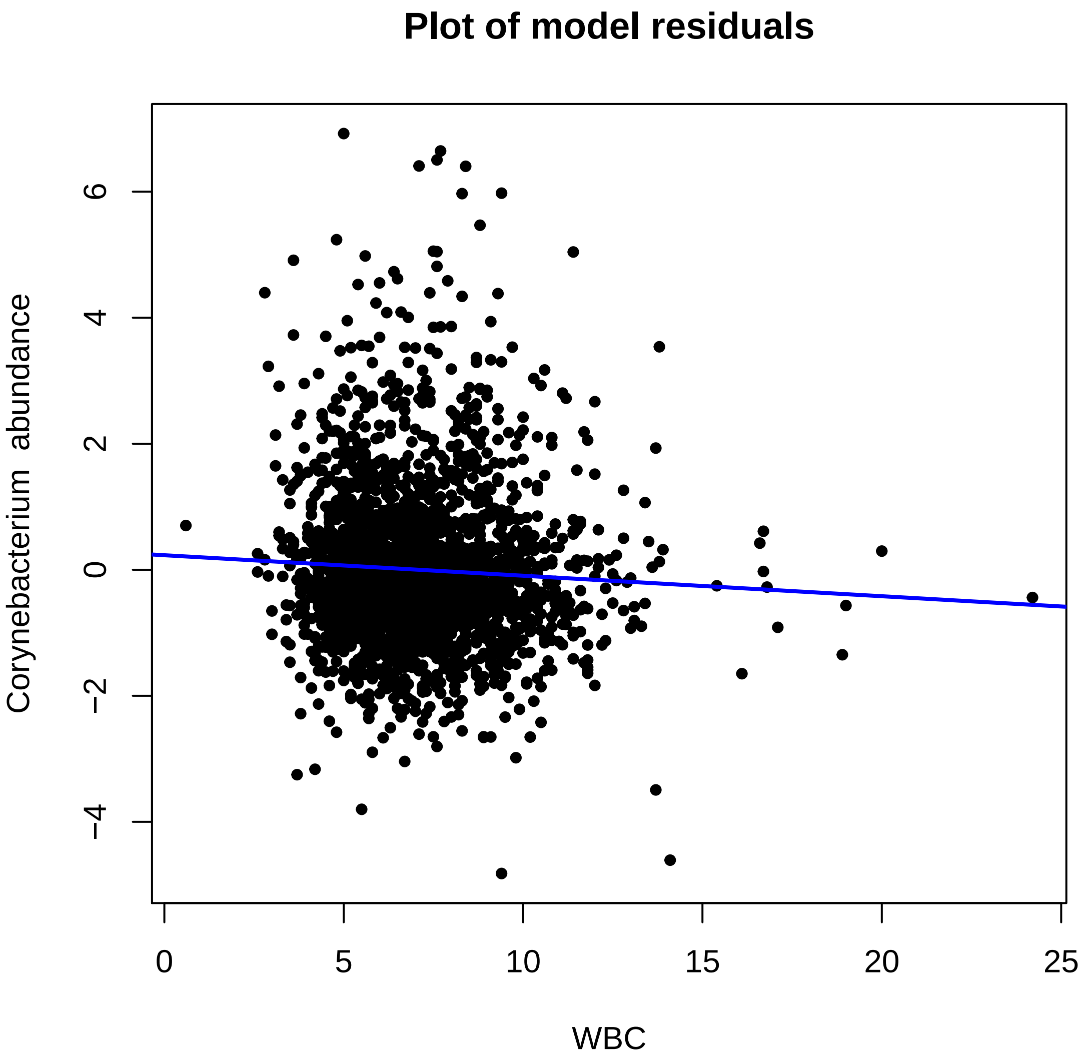

Plot of model residuals
Corynebacterium abundance
WBC
6
4
2
0
−2
−4
0
5
10
15
20
25

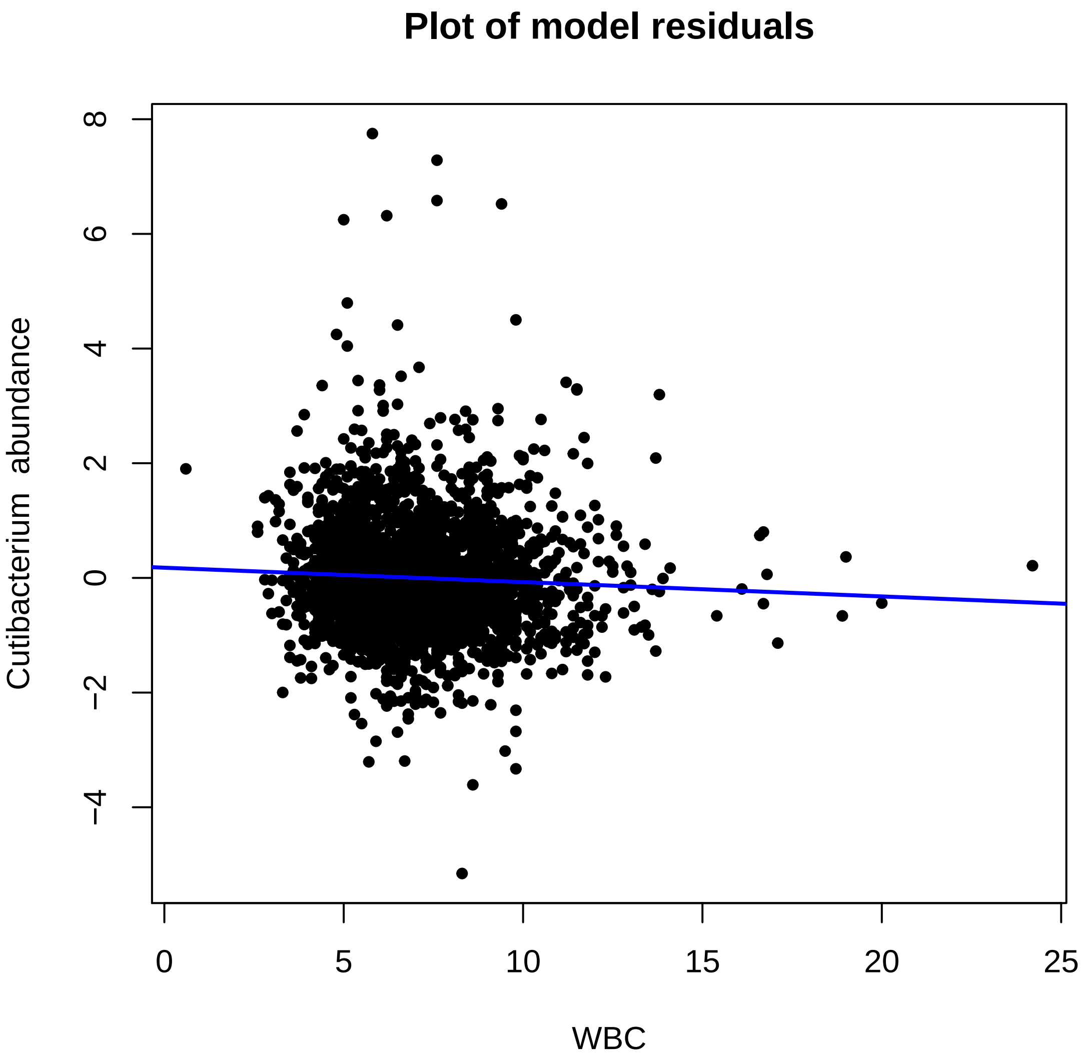
Plot of model residuals
Cutibacterium abundance
WBC
8
6
4
2
0
−2
−4
0
5
10
15
20
25

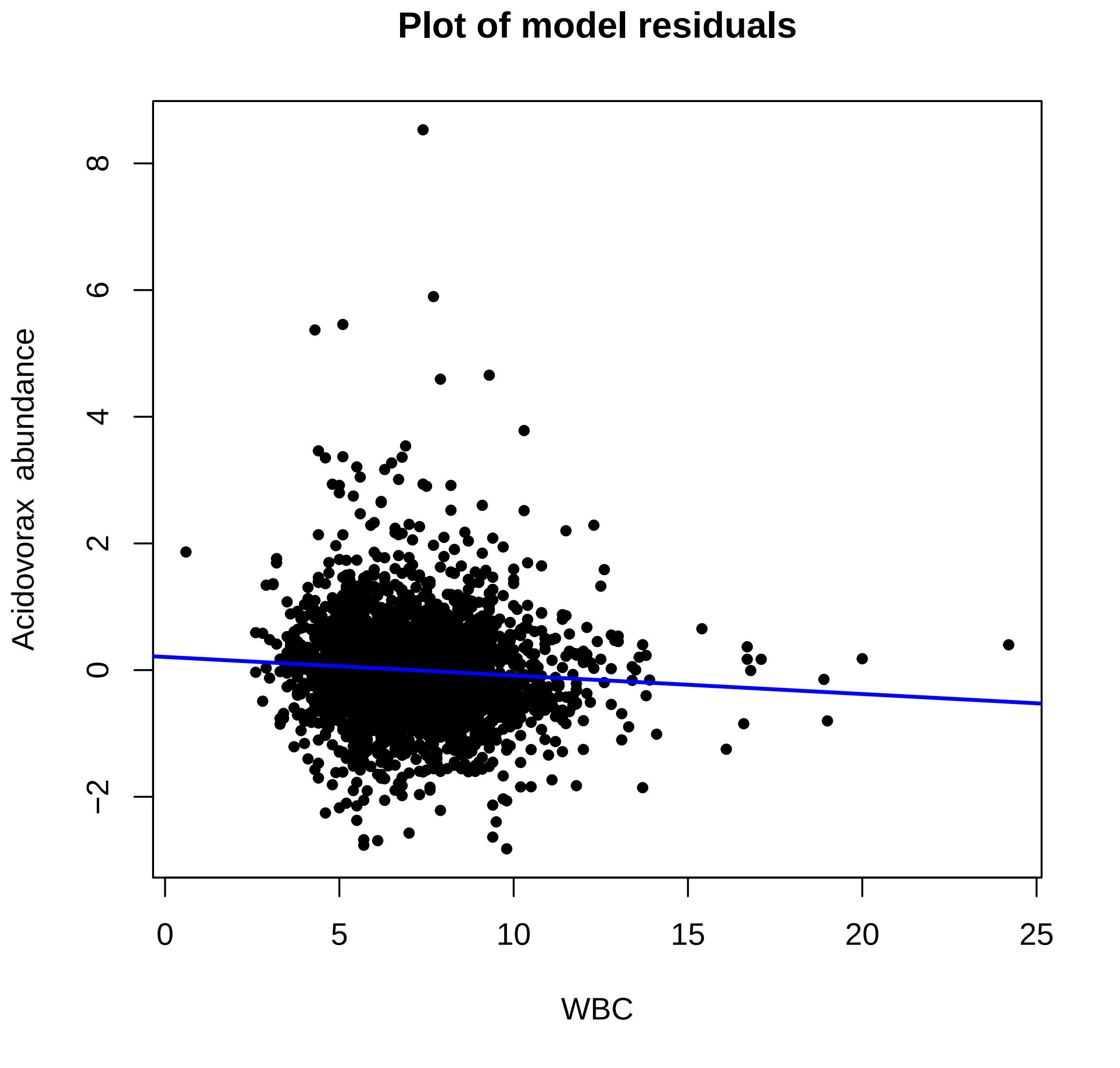
Plot of model residuals
Acidovorax abundance
8
6
4
2
0
−2
0
5
10
15
20
25
WBC

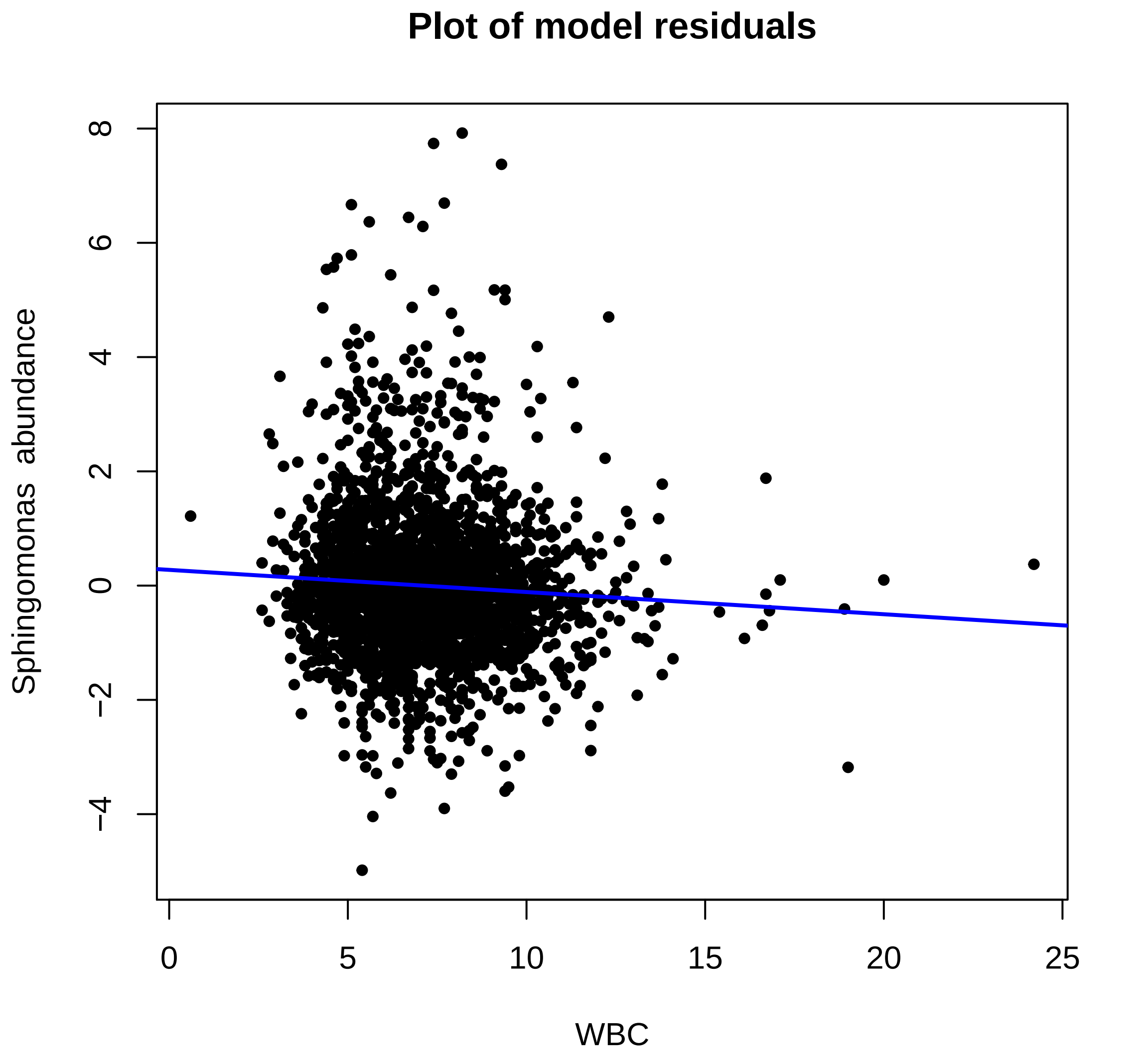
Plot of model residuals
Sphingomonas abundance
WBC
8
6
4
2
0
−2
−4
0
5
10
15
20
25

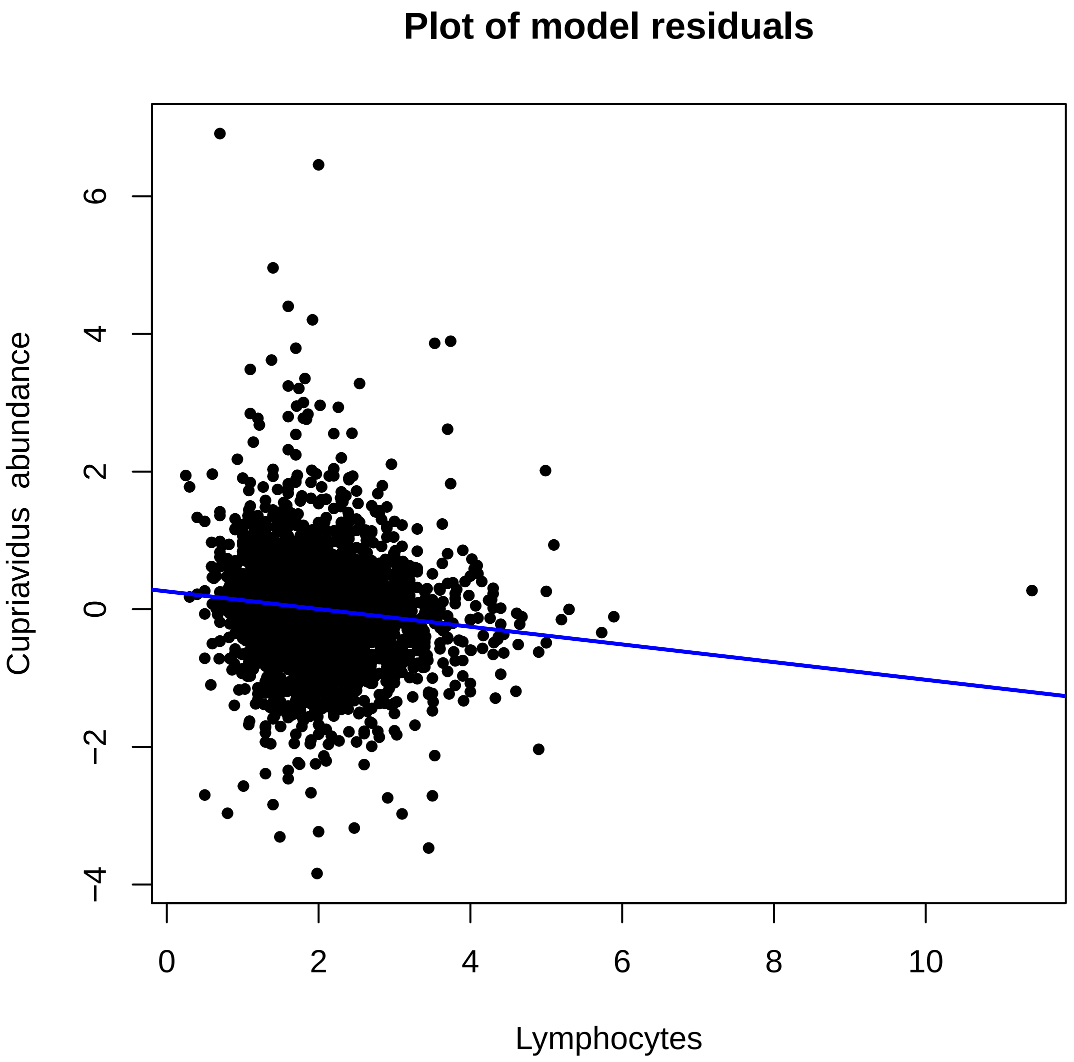
Plot of model residuals
Cupriavidus abundance
Lymphocytes
6
4
2
0
−2
−4
0
2
4
6
8
10

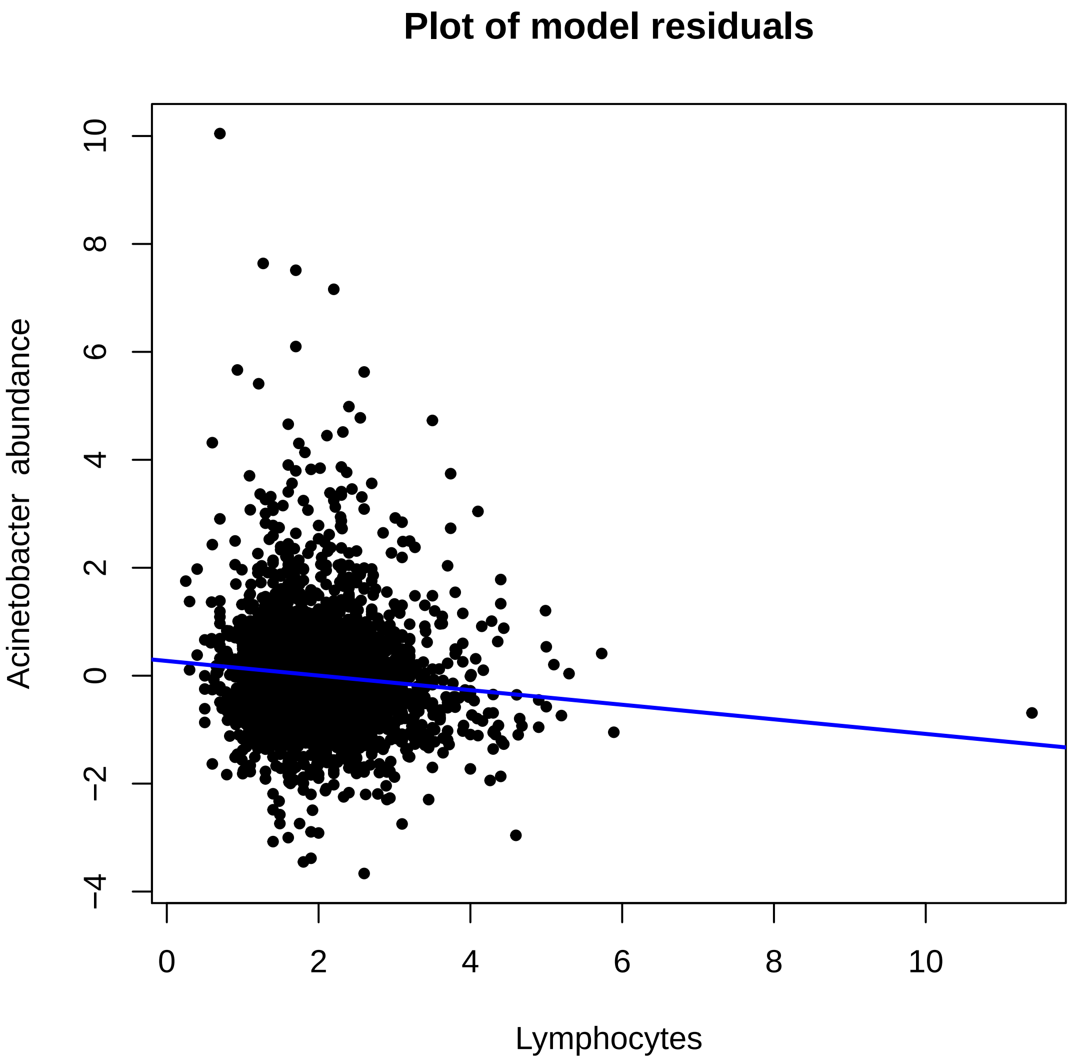

Plot of model residuals
Acinetobacter abundance
Lymphocytes
10
8
6
4
2
0
−2
−4
0
2
4
6
8
10

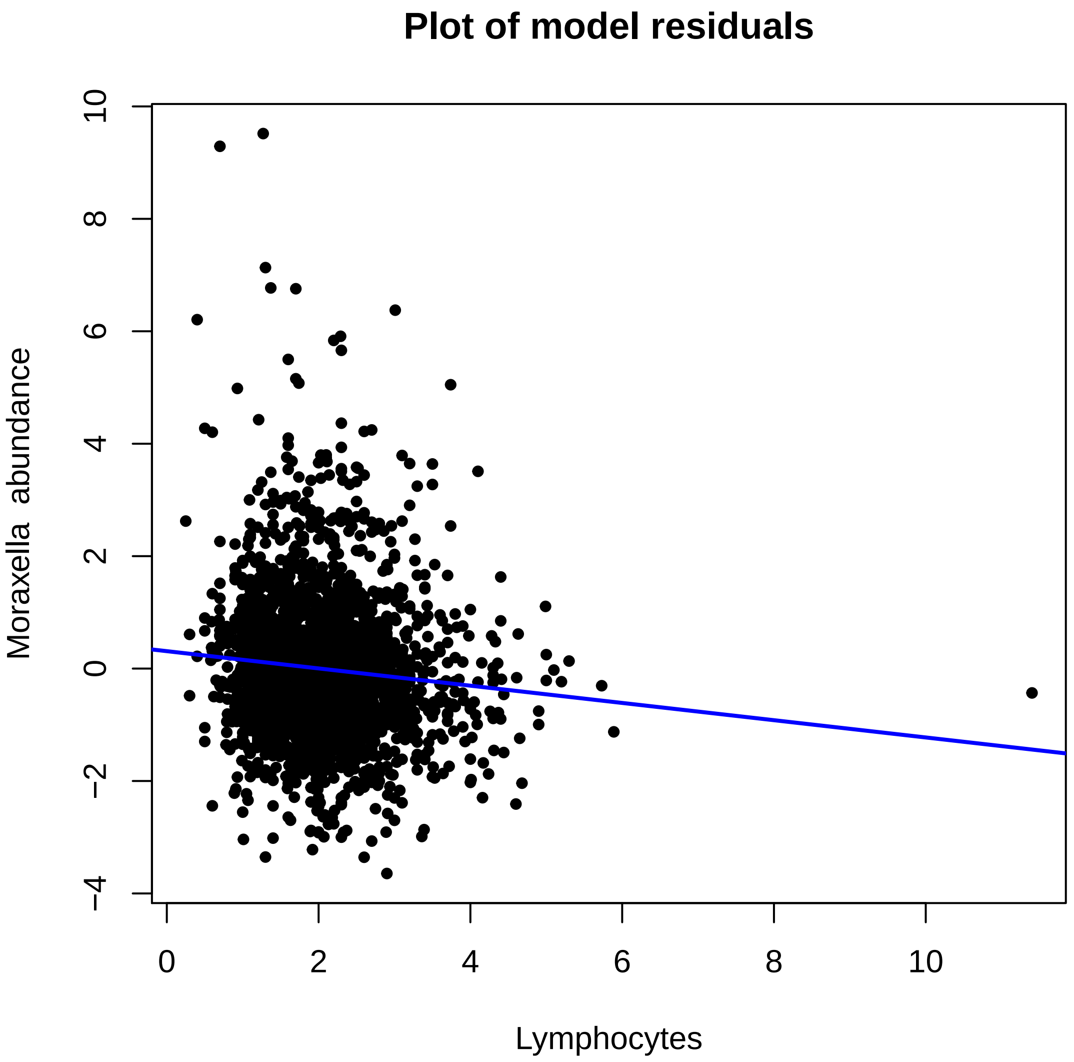
Plot of model residuals
Moraxella abundance
Lymphocytes
10
8
6
4
2
0
−2
−4
0
2
4
6
8
10

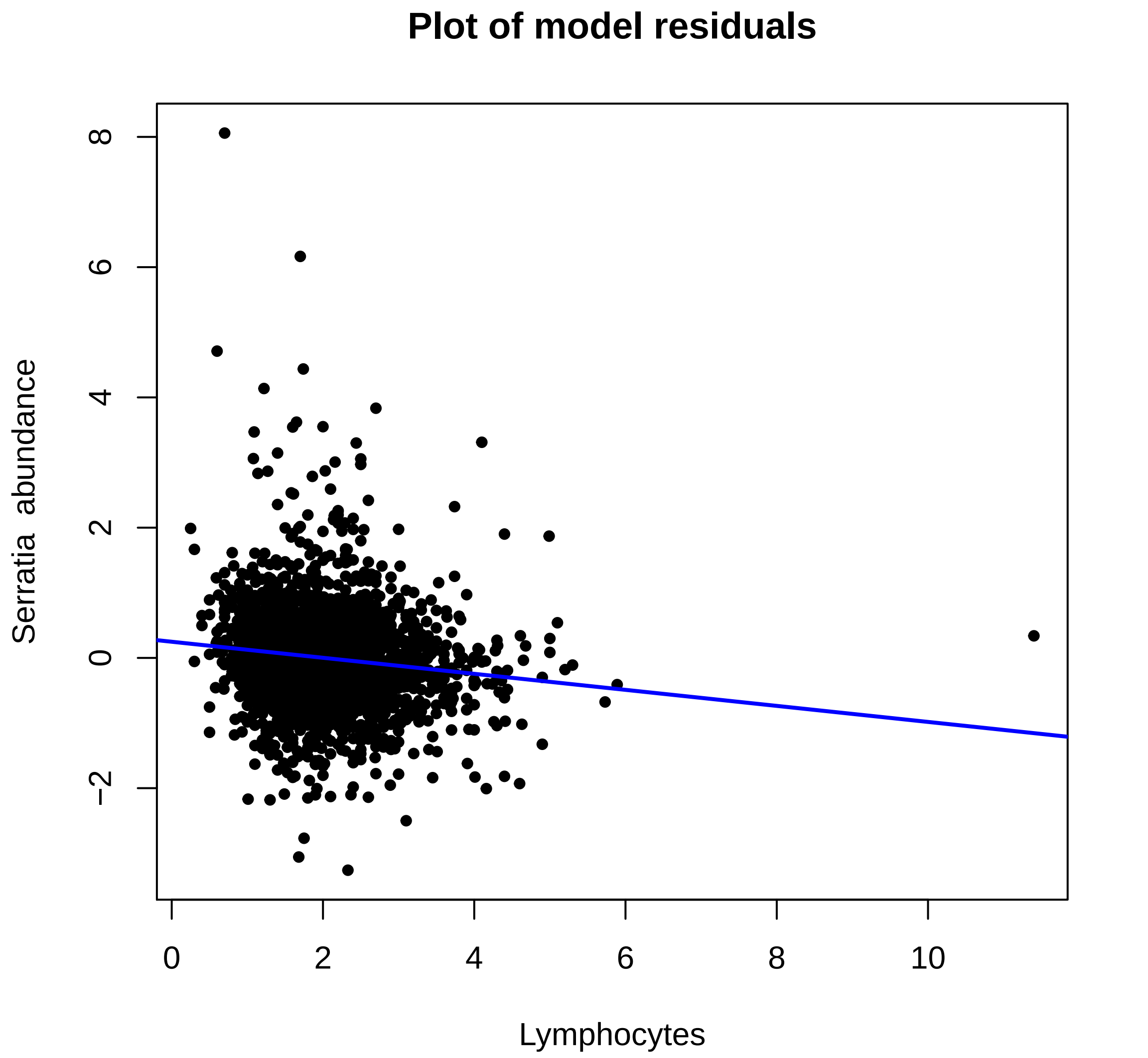

Plot of model residuals
Serratia abundance
8
6
4
2
0
−2
0
2
4
6
8
10
Lymphocytes

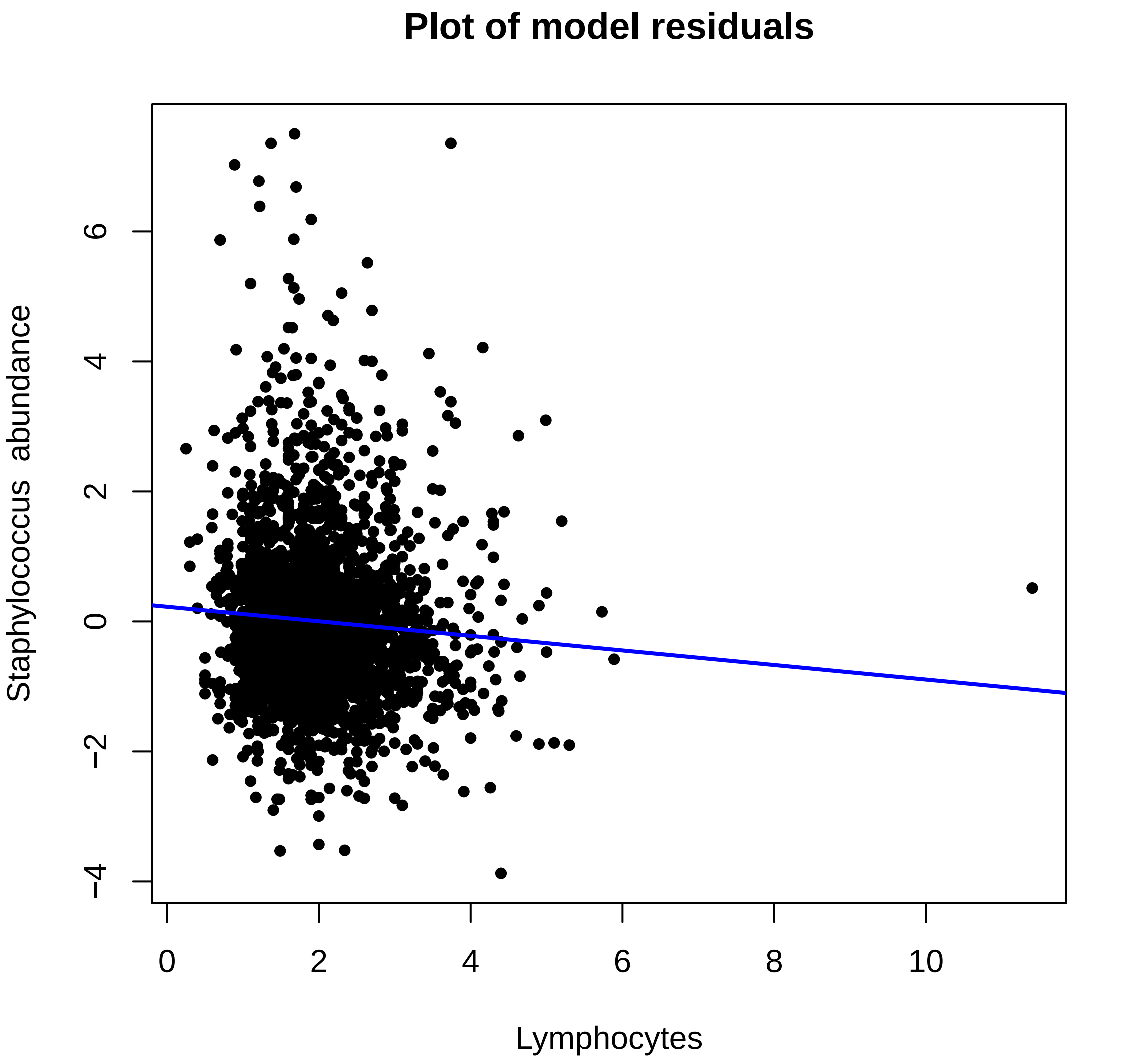
Plot of model residuals
Staphylococcus abundance
Lymphocytes
0
2
4
6
8
10
−4
−2
0
2
4
6

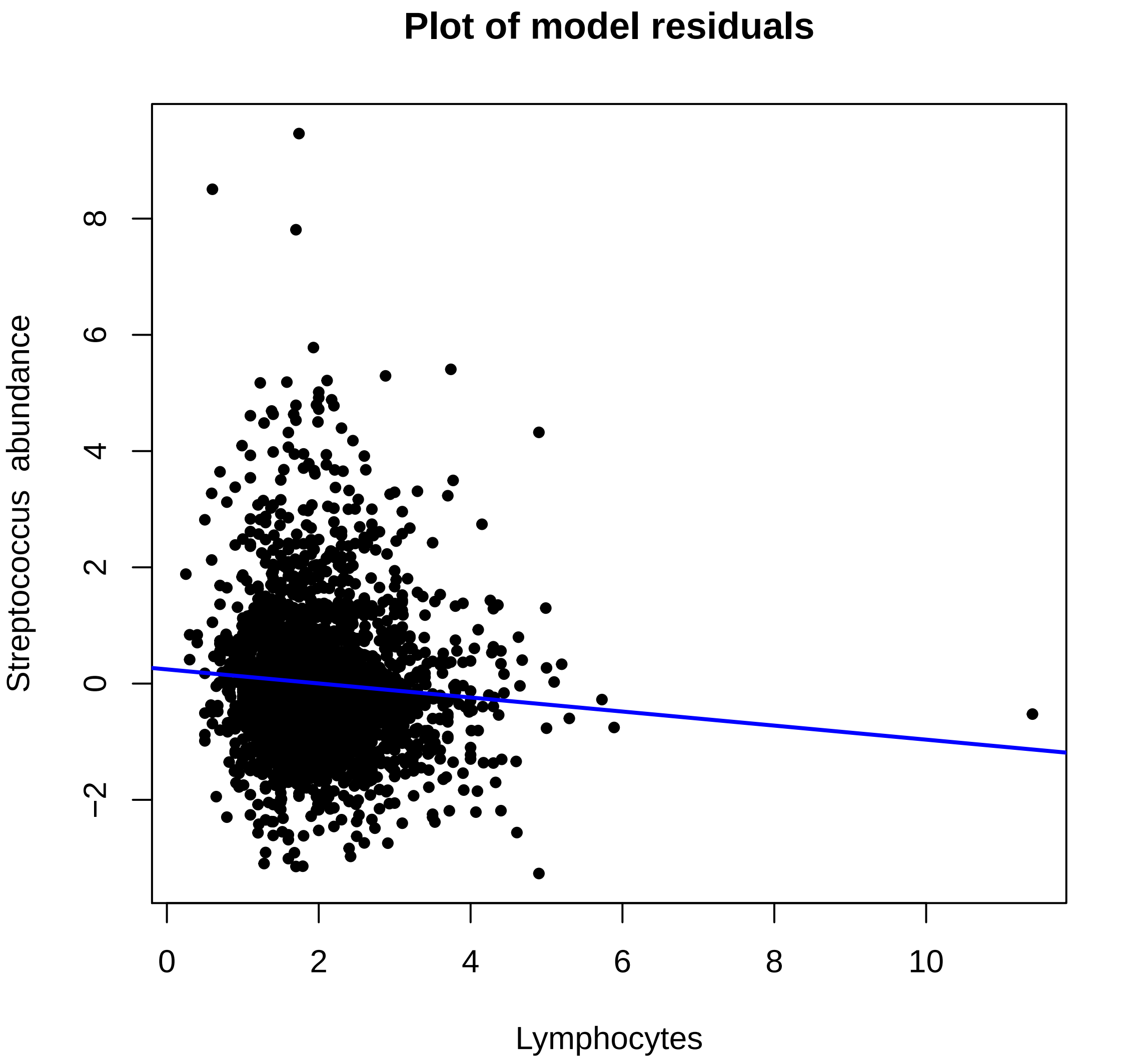
Plot of model residuals
Streptococcus abundance
Lymphocytes
−2
0
2
4
6
8
0
2
4
6
8
10

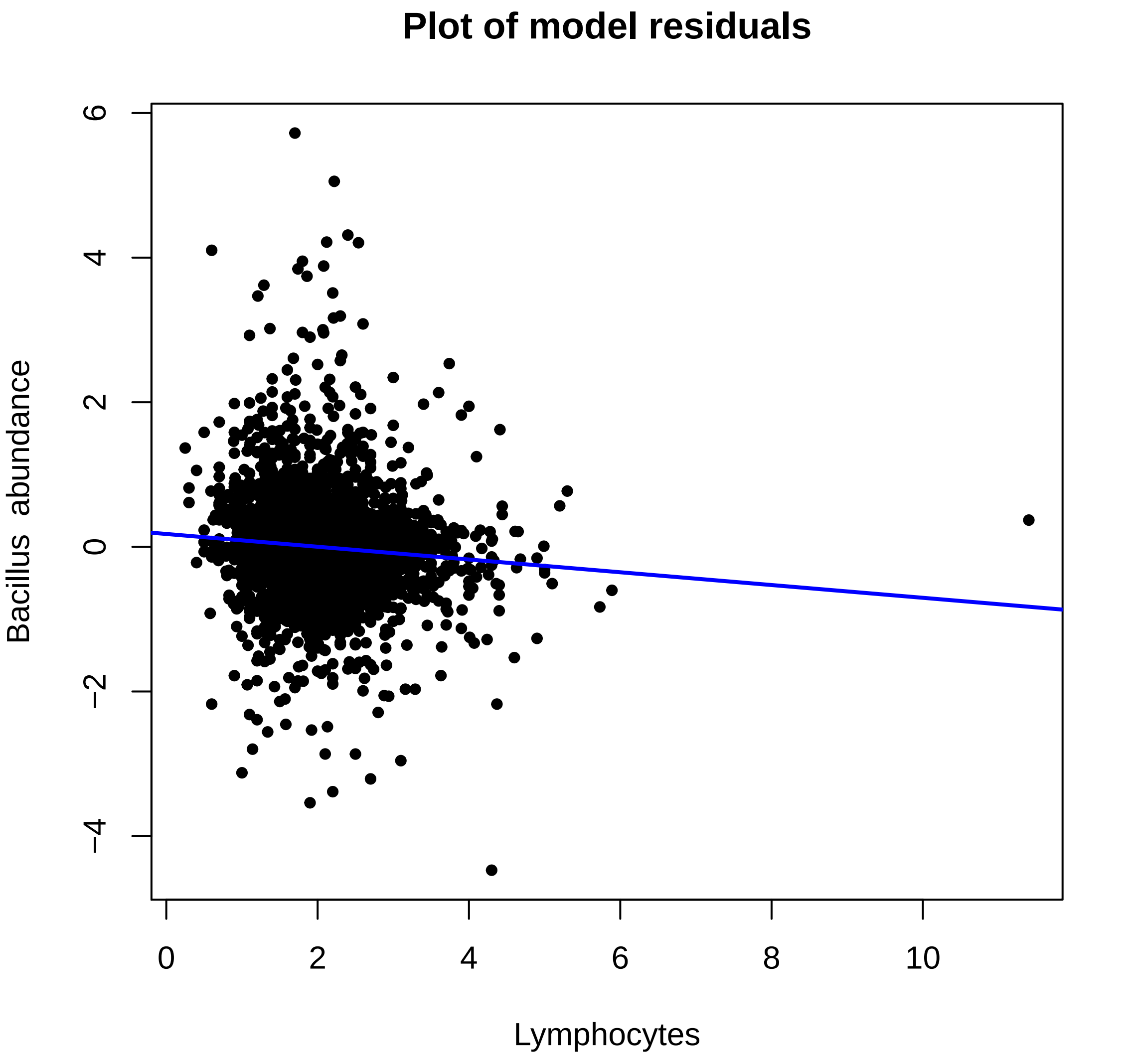

Plot of model residuals
Bacillus abundance
Lymphocytes
6
4
2
0
−2
−4
0
2
4
6
8
10

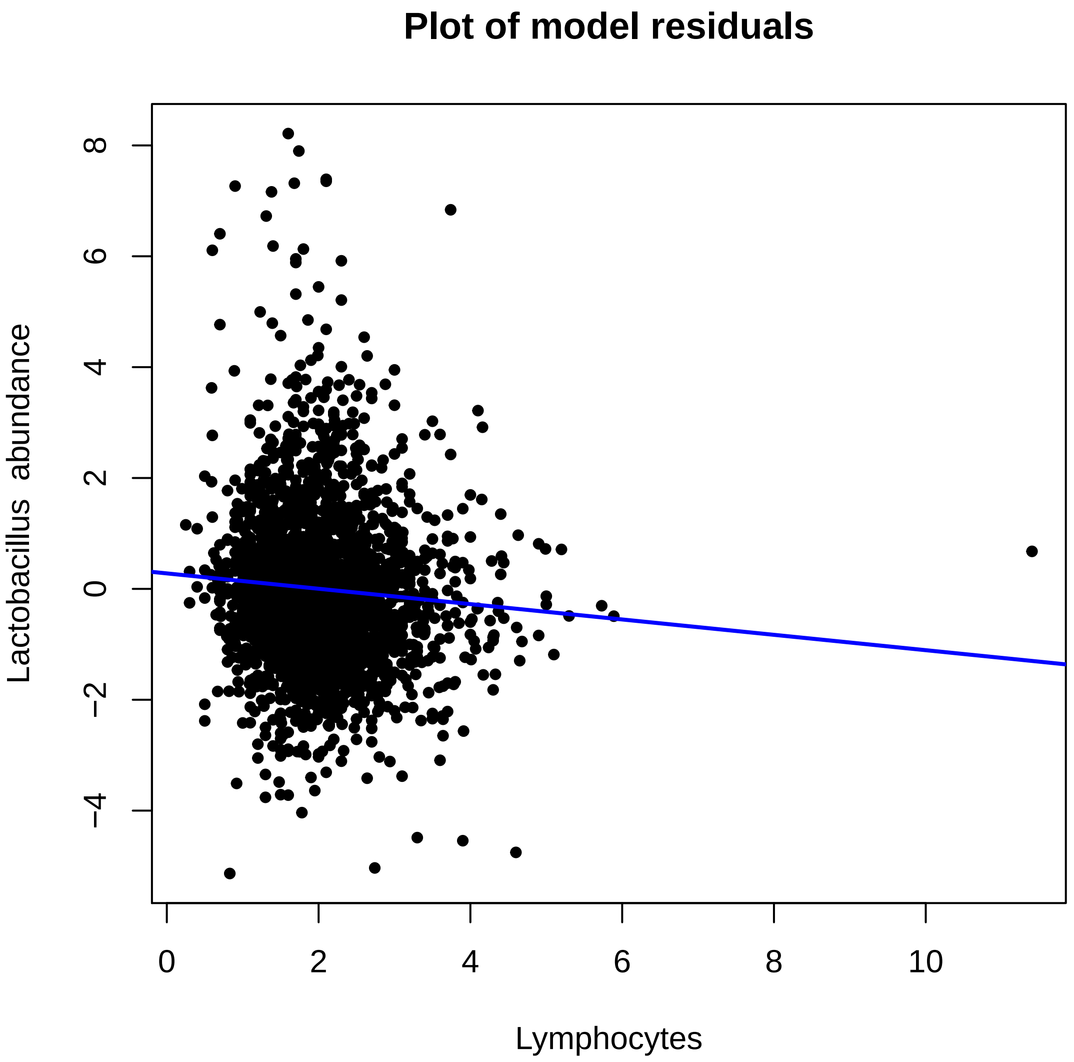

Plot of model residuals
Lactobacillus abundance
Lymphocytes
−4
−2
0
2
4
6
8
0
2
4
6
8
10

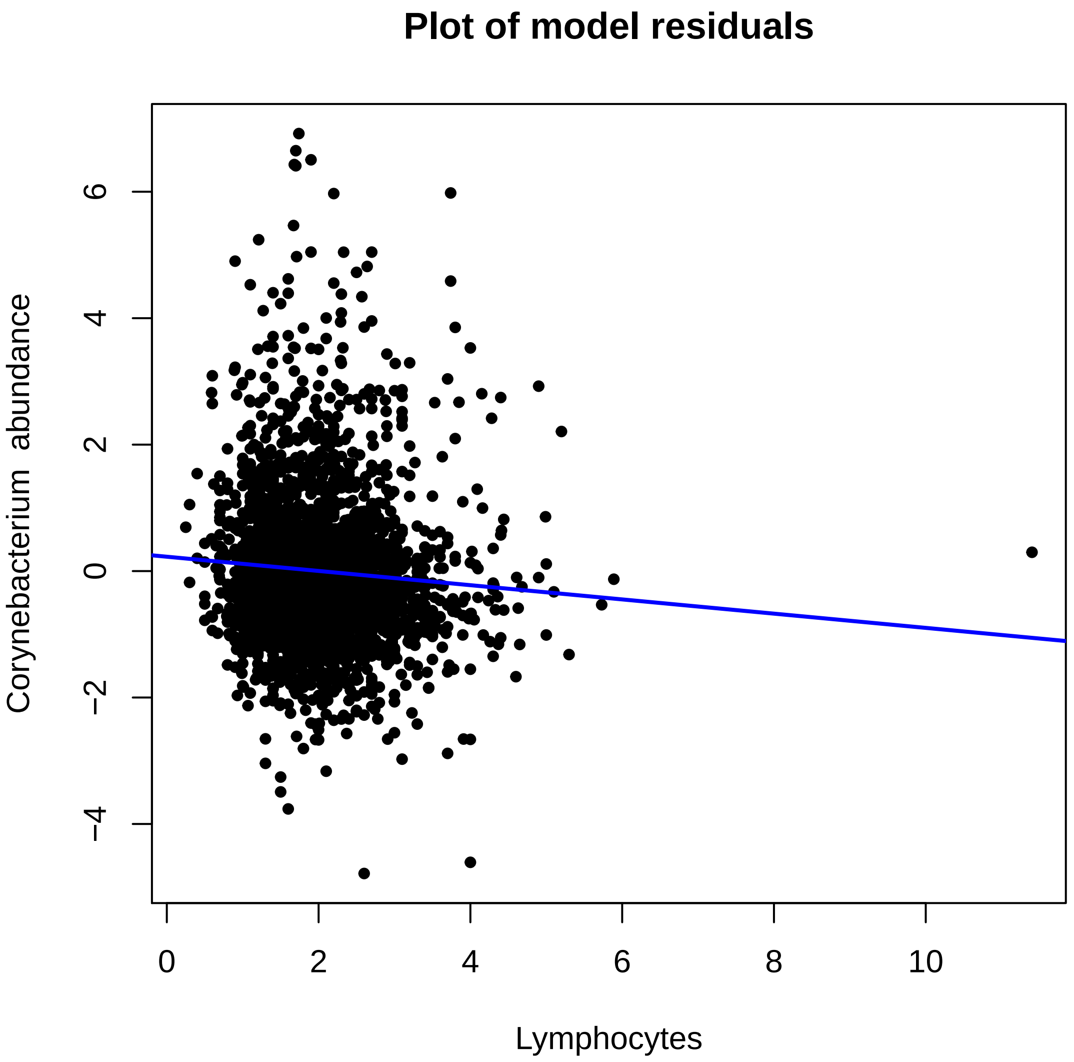

Plot of model residuals
Corynebacterium abundance
Lymphocytes
6
4
2
0
−2
−4
0
2
4
6
8
10

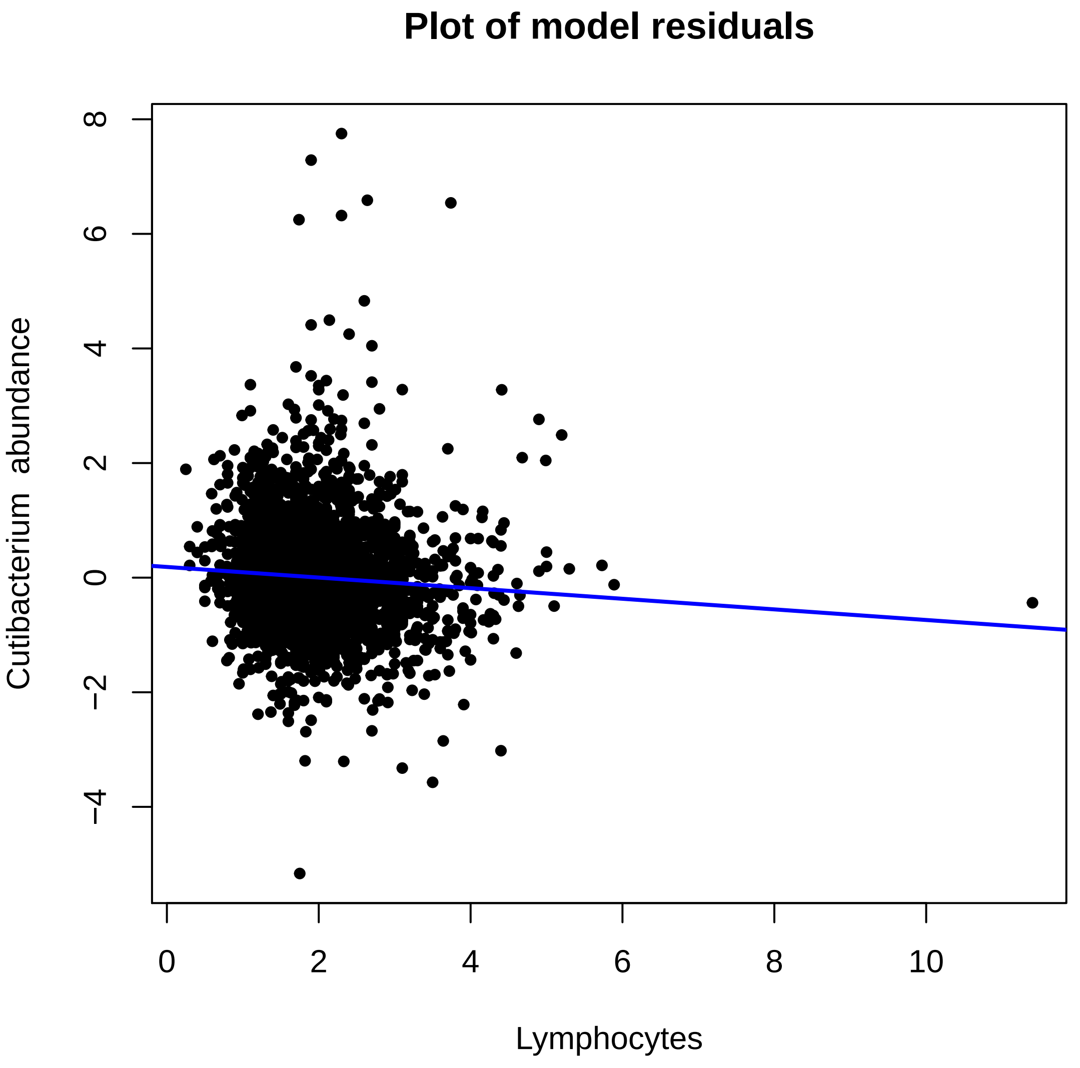
Plot of model residuals
Cutibacterium abundance
Lymphocytes
8
6
4
2
0
−2
−4
0
2
4
6
8
10

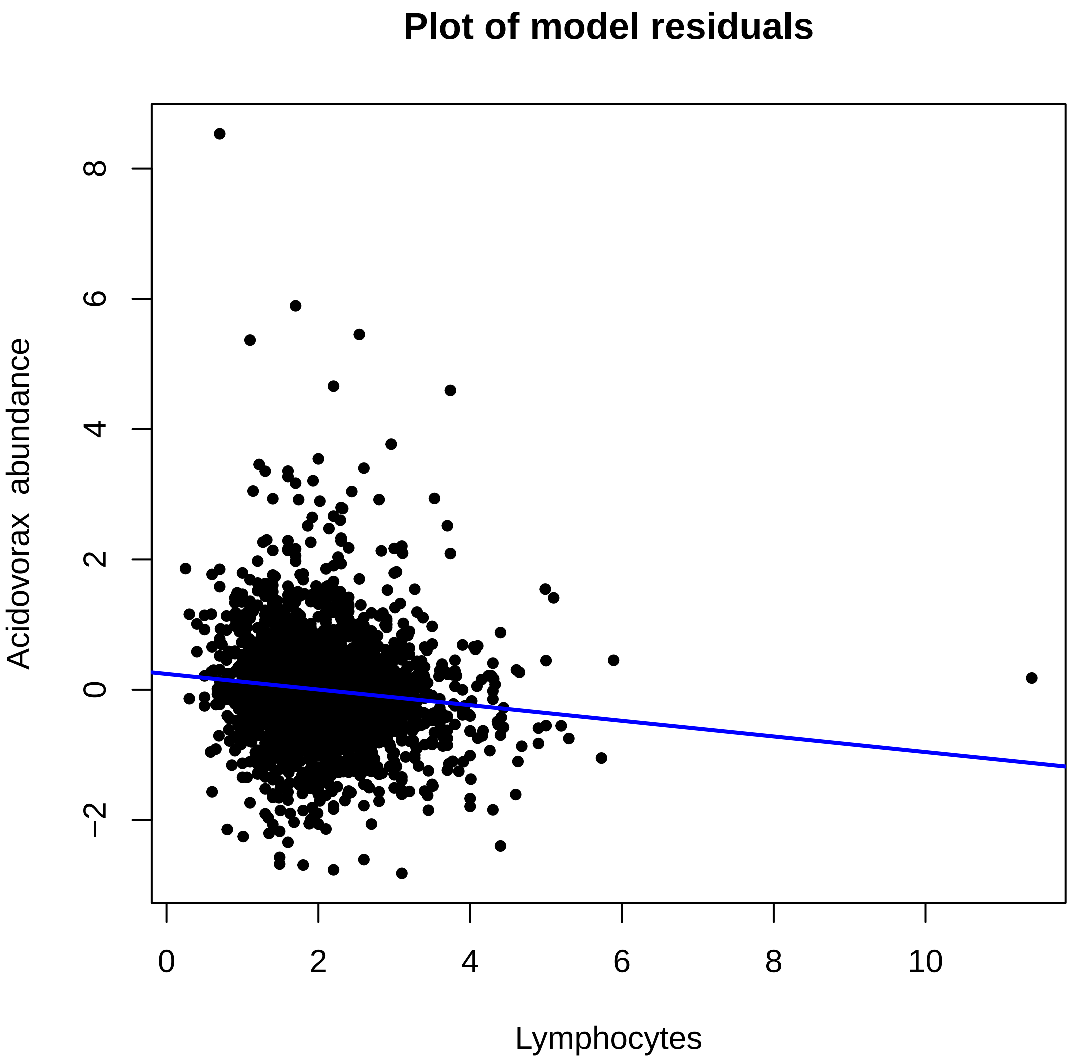
Plot of model residuals
Acidovorax abundance
Lymphocytes
0
2
4
6
8
10
−2

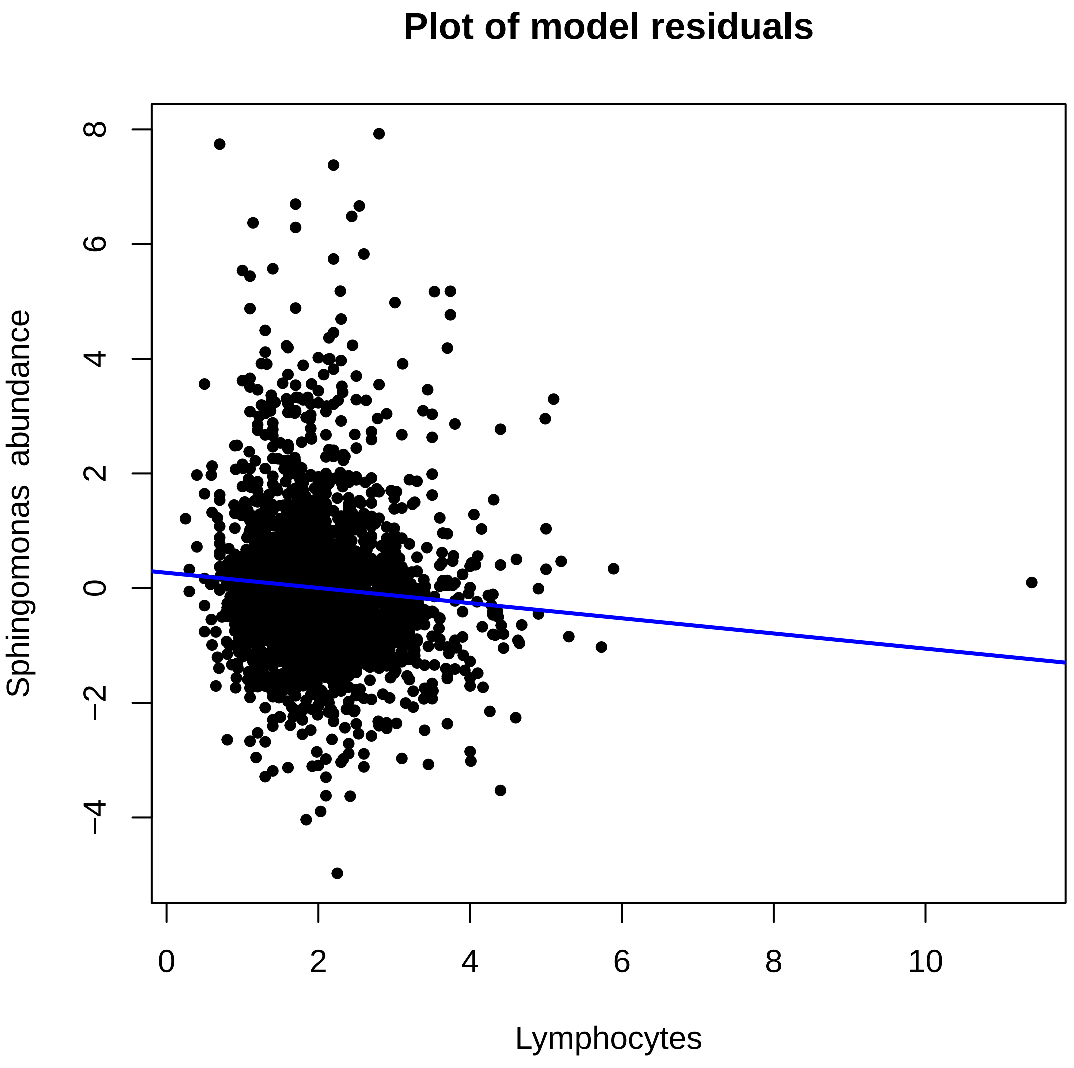
Plot of model residuals
Sphingomonas abundance
Lymphocytes
8
6
4
2
0
−2
−4
0
2
4
6
8
10

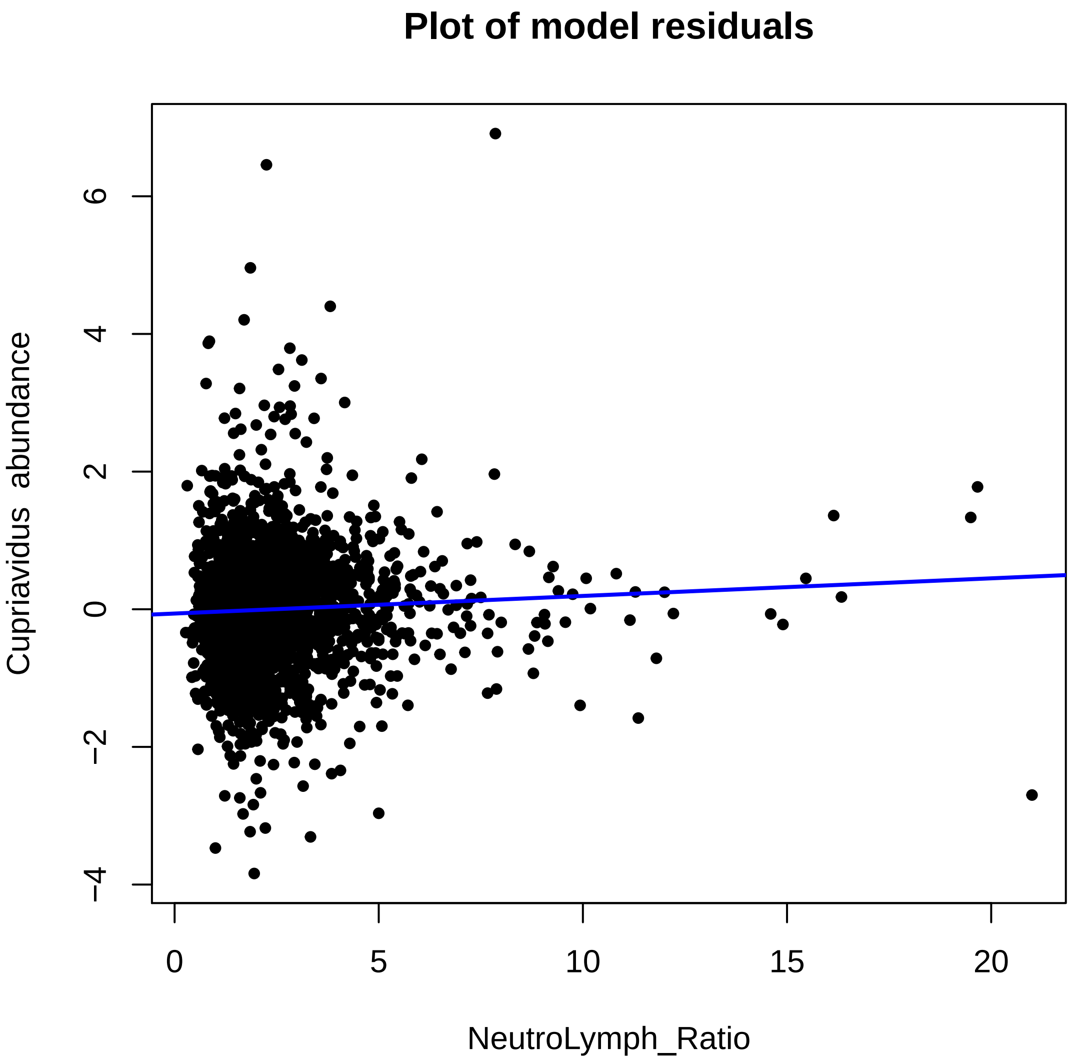
Plot of model residuals
Cupriavidus_abundance
NeutroLymph_Ratio
−4
−2
0
2
4
6
0
5
10
15
20

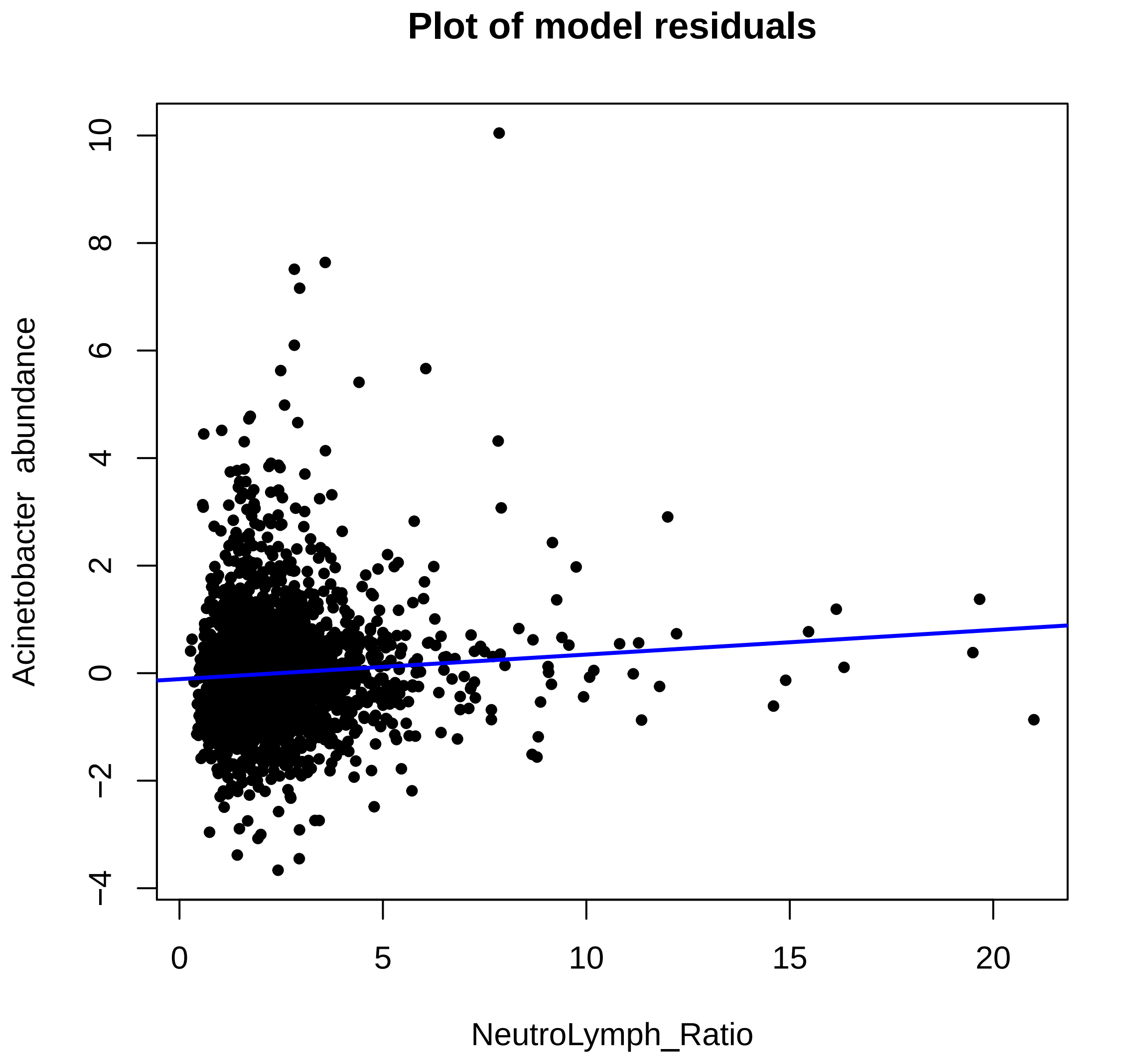

Plot of model residuals
Acinetobacter abundance
NeutroLymph_Ratio
10
8
6
4
2
0
−2
−4
0
5
10
15
20

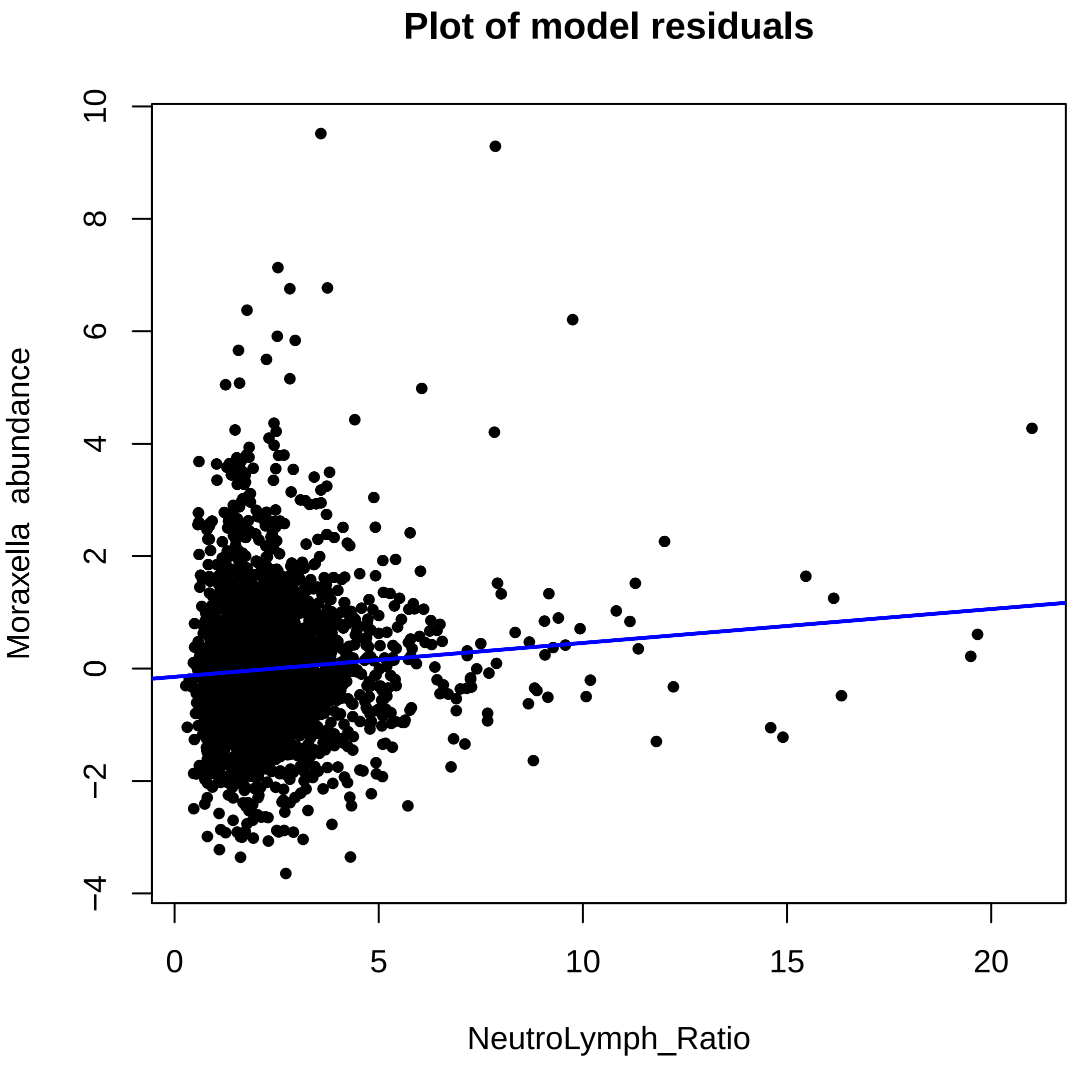

Plot of model residuals
Moraxella abundance
NeutroLymph_Ratio
−4
−2
0
2
4
6
8
10
0
5
10
15
20

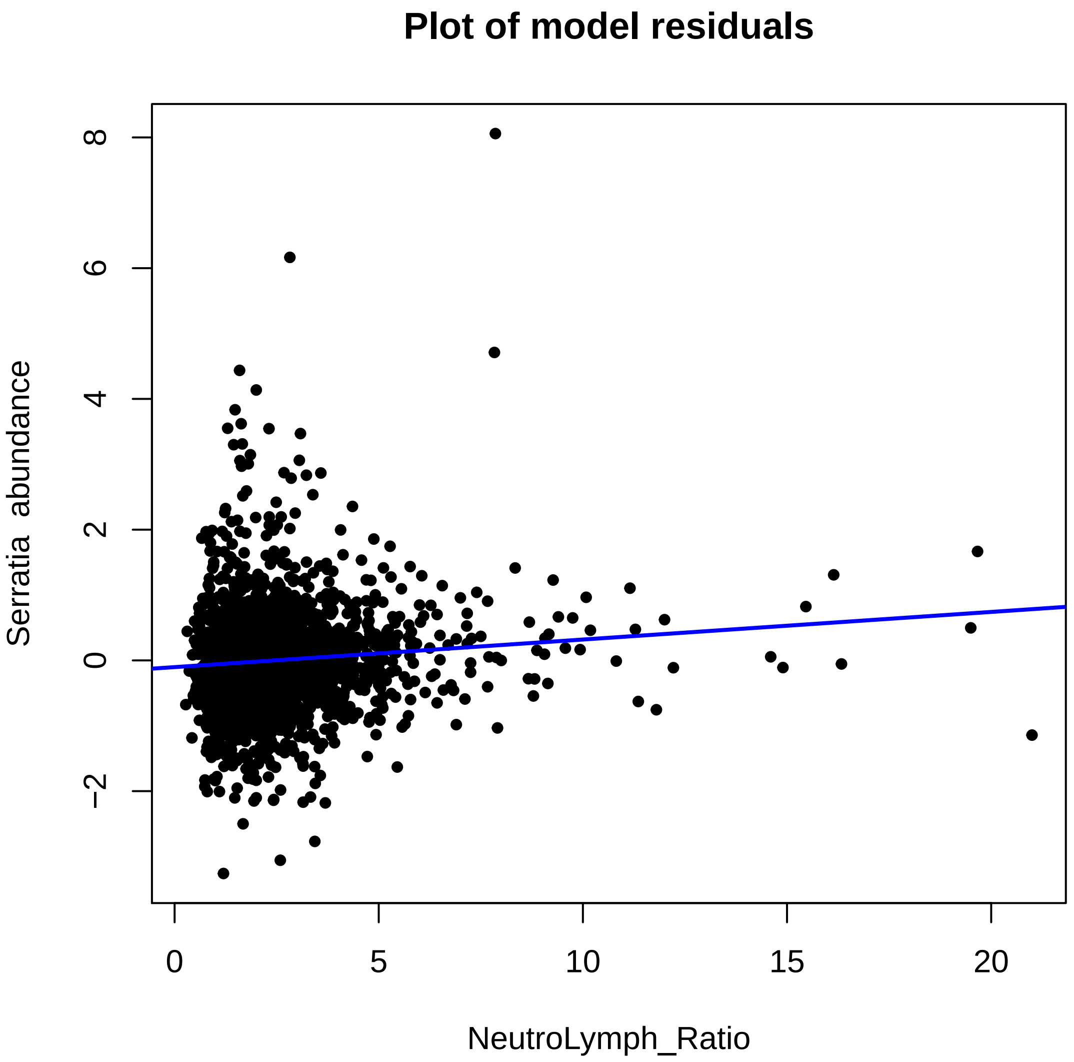
Plot of model residuals
Serratia abundance
NeutroLymph_Ratio
−2
0
2
4
6
8
0
5
10
15
20

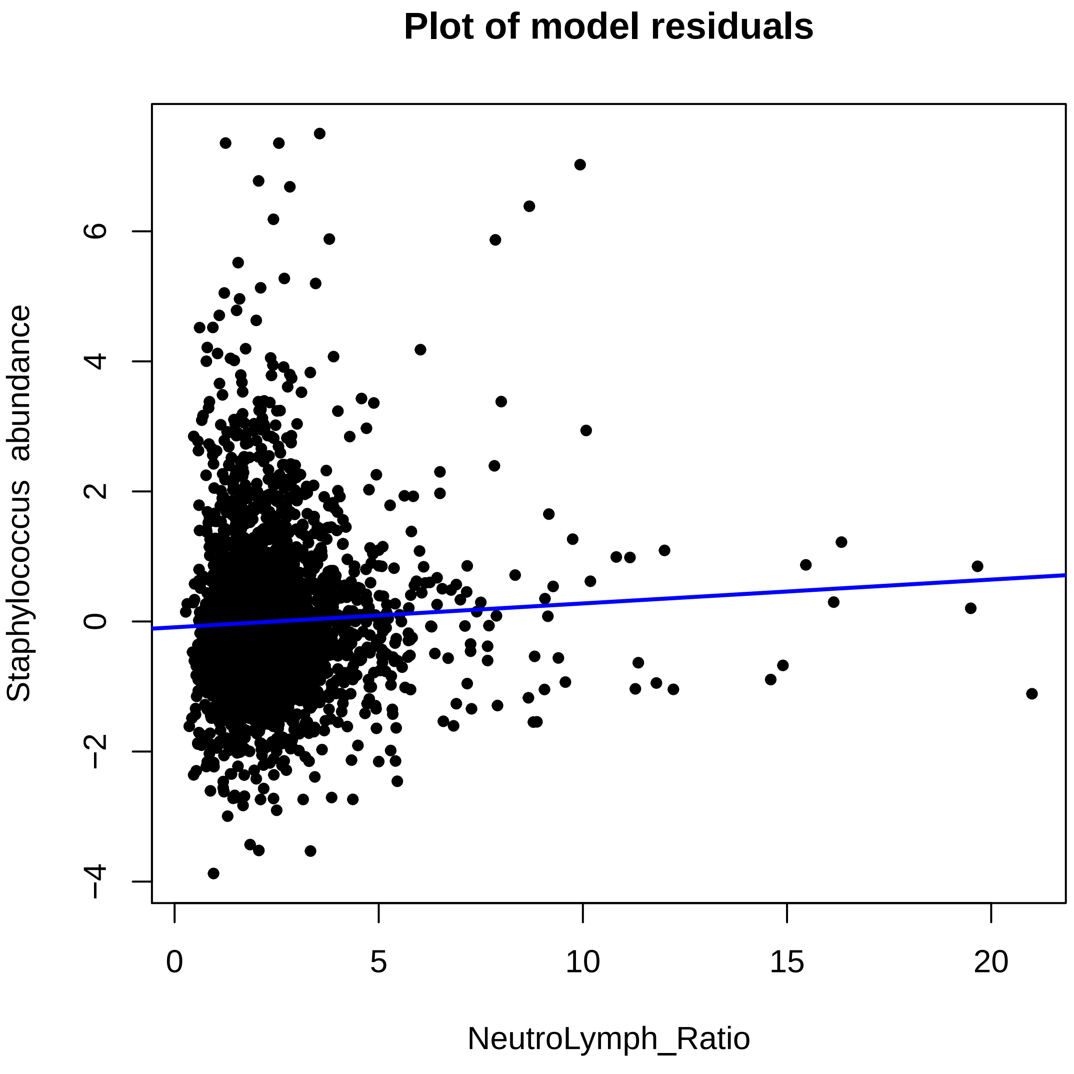

Plot of model residuals
Staphylococcus abundance
−4
−2
0
2
4
6
0
5
10
15
20
NeutroLymph_Ratio

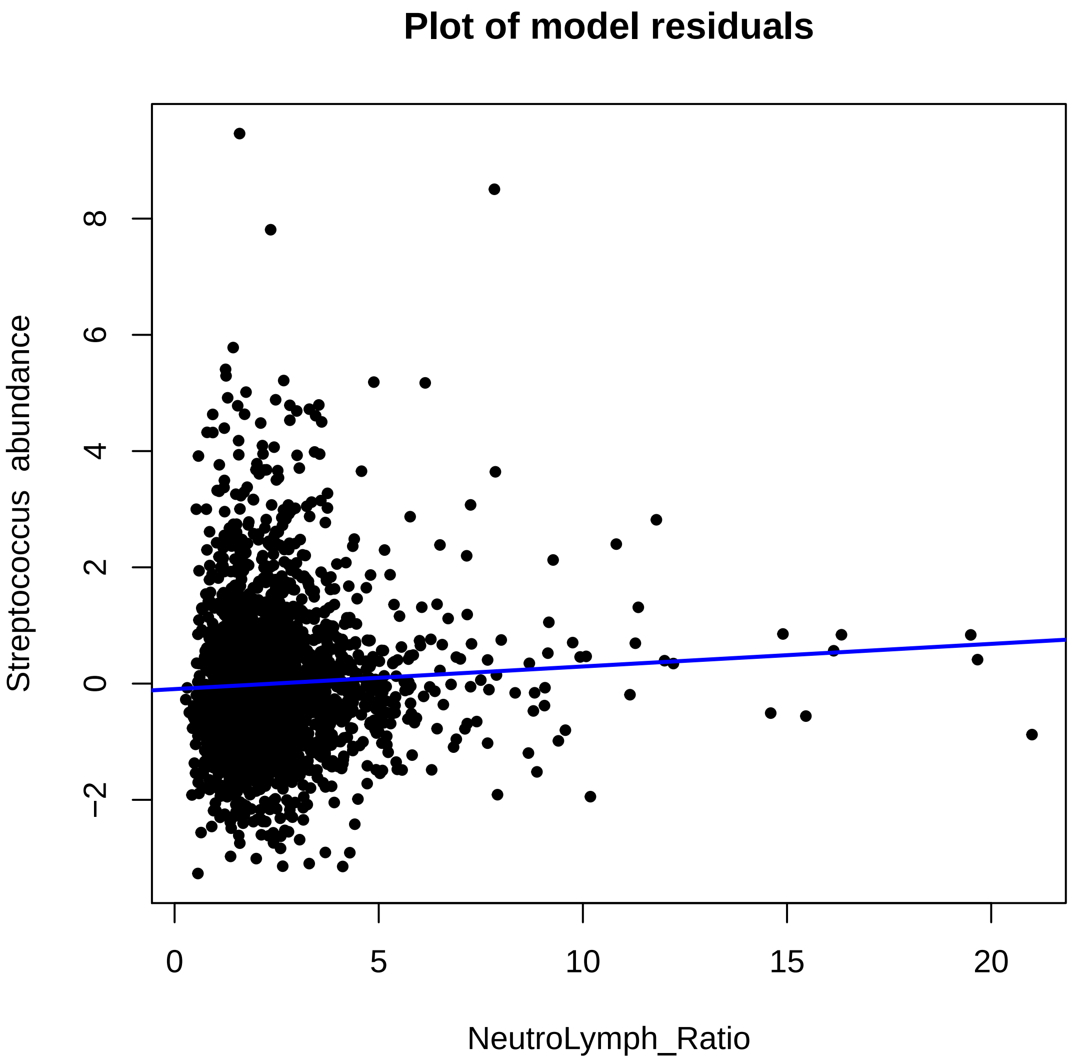

Plot of model residuals
Streptococcus_abundance
NeutroLymph_Ratio
−2
0
2
4
6
8
0
5
10
15
20

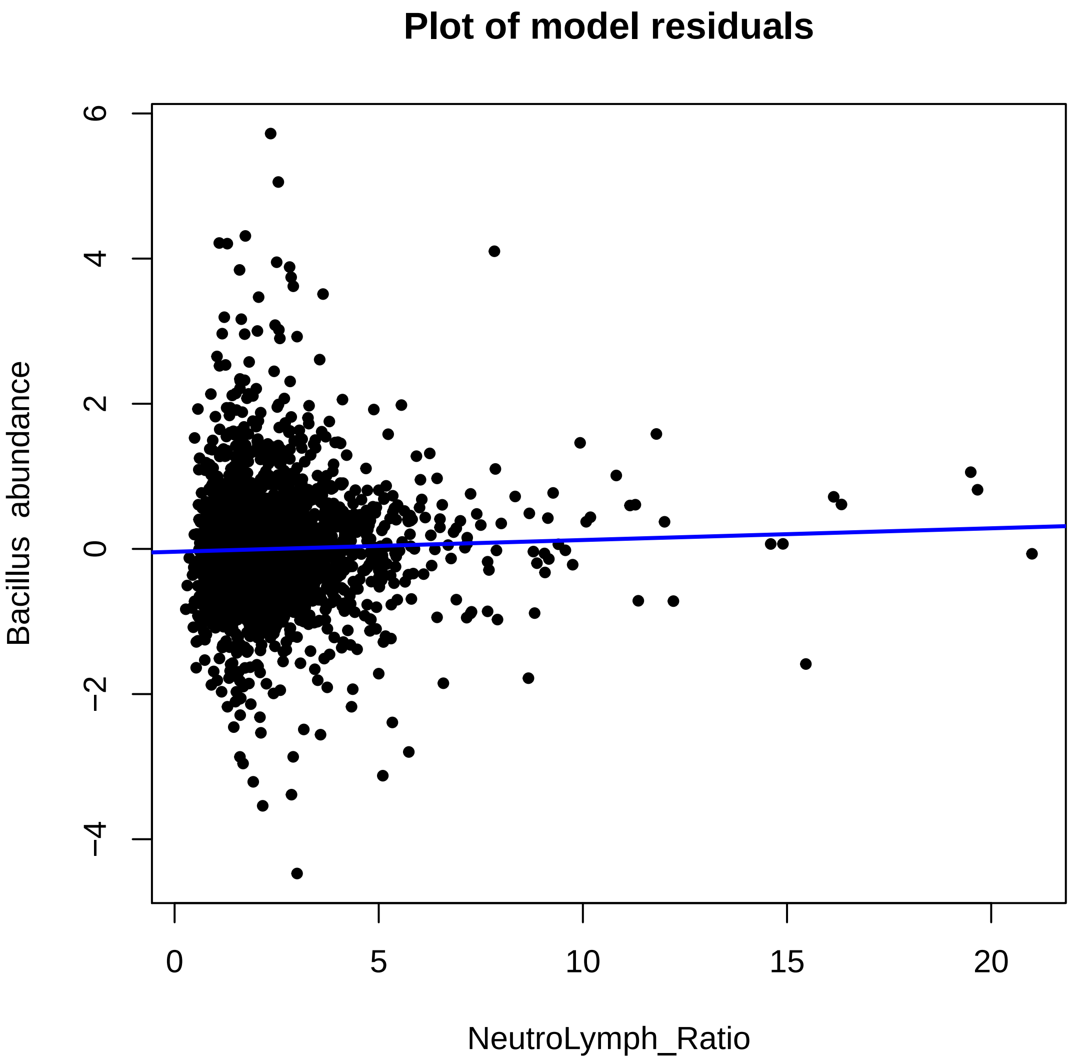
Plot of model residuals
Bacillus abundance
NeutroLymph_Ratio
6
4
2
0
−2
−4
0
5
10
15
20

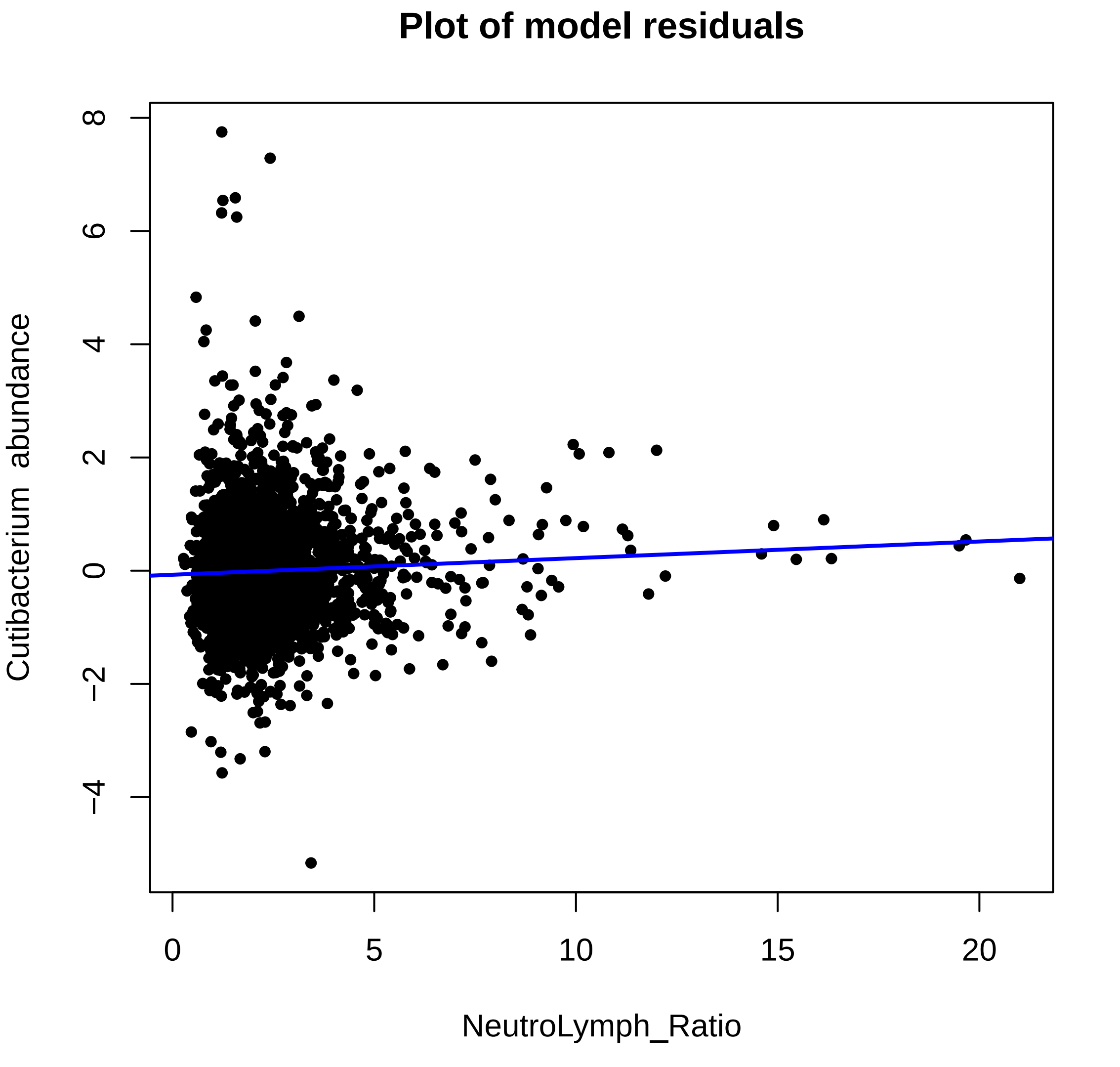
Plot of model residuals
Cutibacterium abundance
NeutroLymph_Ratio
8
6
4
2
0
−2
−4
0
5
10
15
20

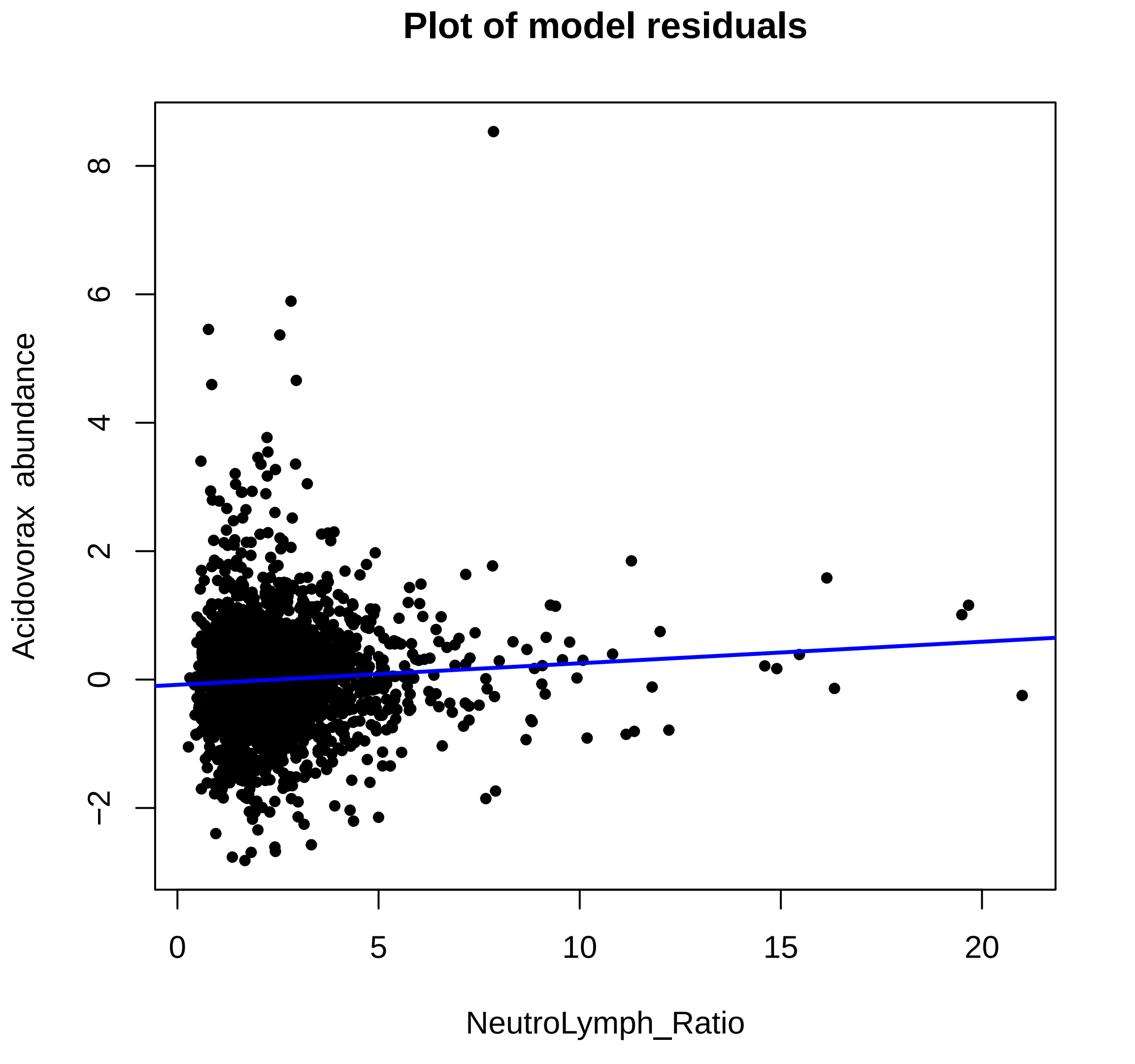

Plot of model residuals
Acidovorax abundance
NeutroLymph_Ratio
0
5
10
15
20
−2
0
2
4
6
8

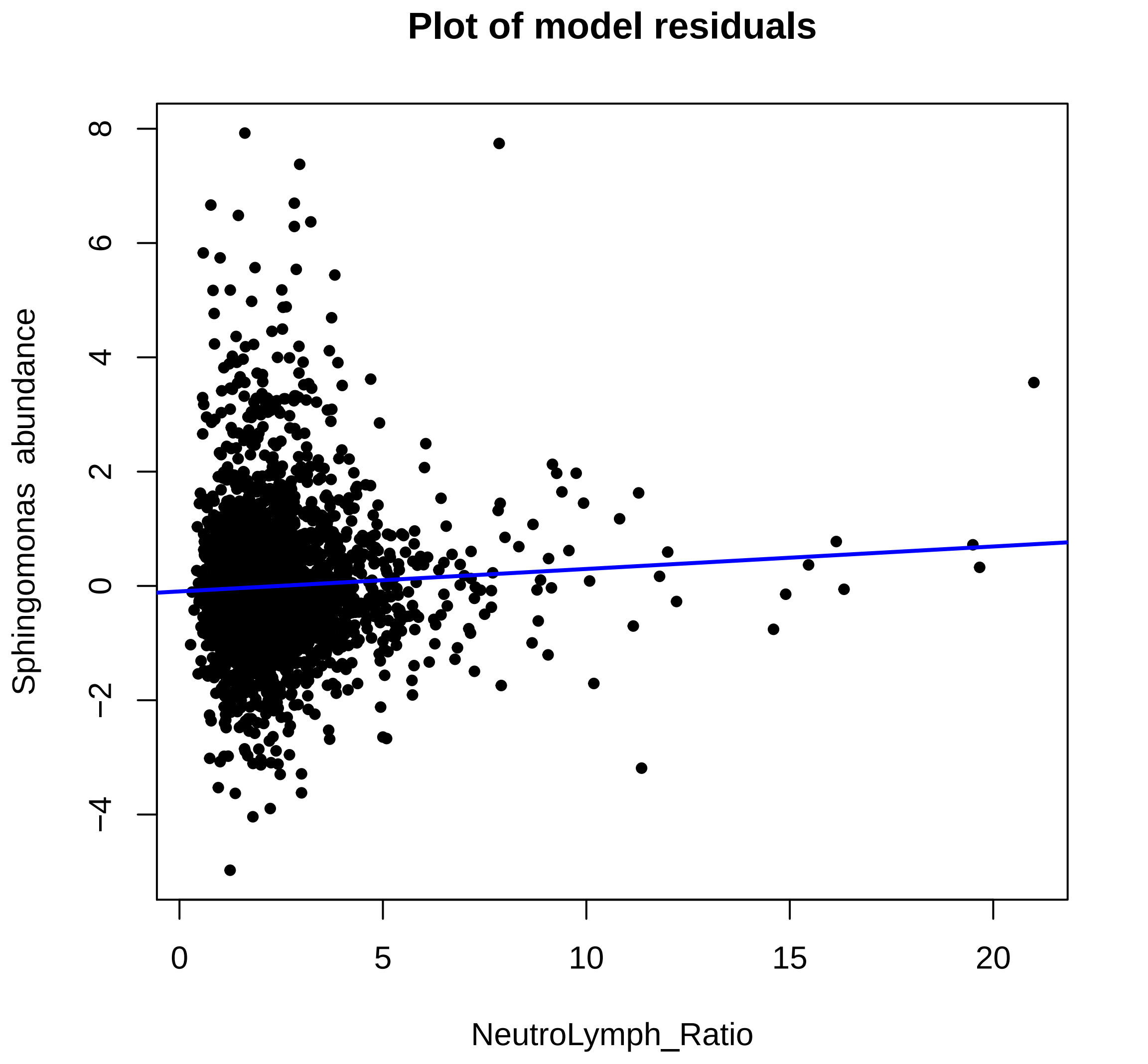
Plot of model residuals
Sphingomonas abundance
NeutroLymph_Ratio
8
6
4
2
0
−2
−4
0
5
10
15
20

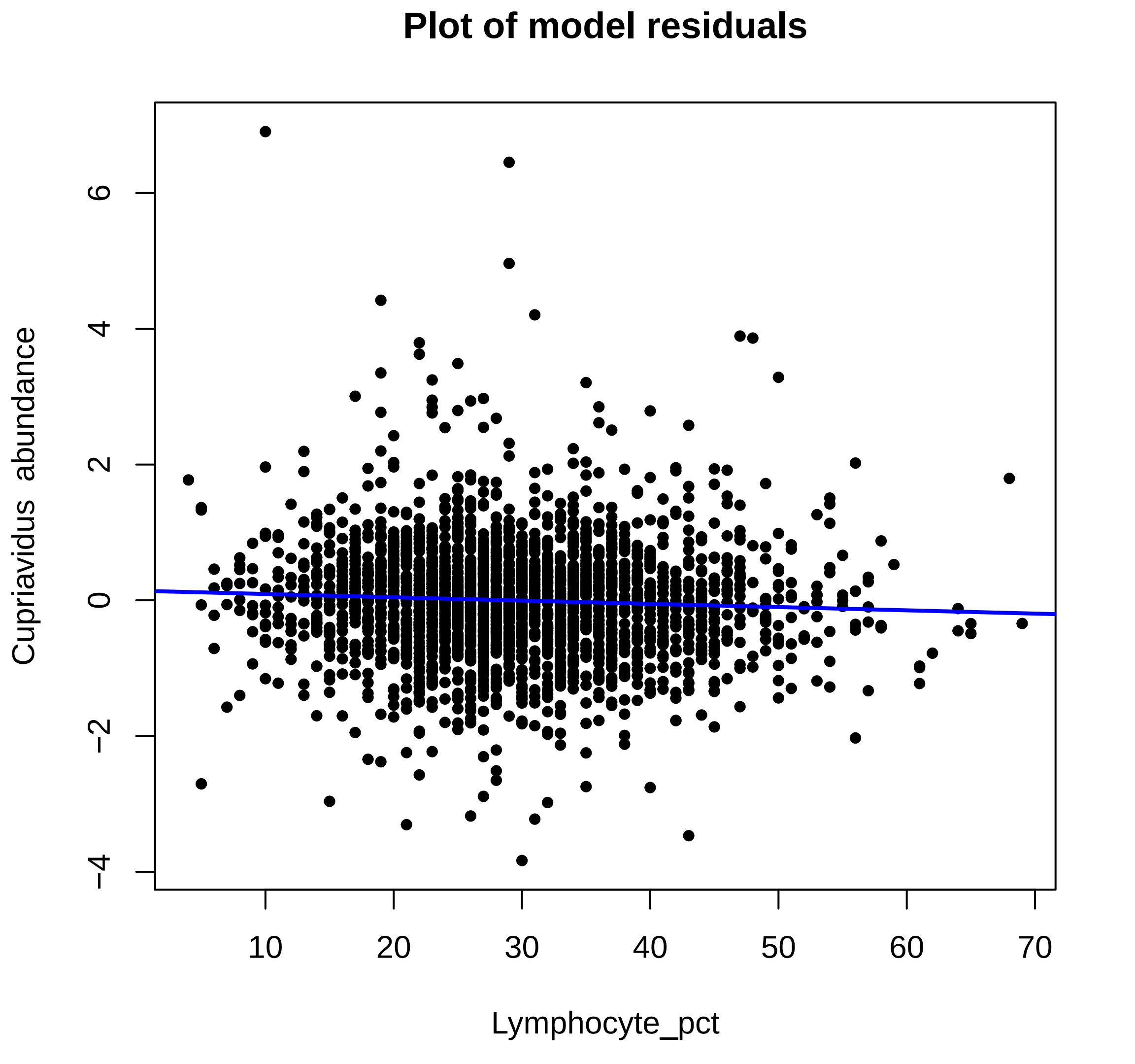
Plot of model residuals
Cupriavidus abundance
Lymphocyte_pct
−4
−2
0
2
4
6
10
20
30
40
50
60
70

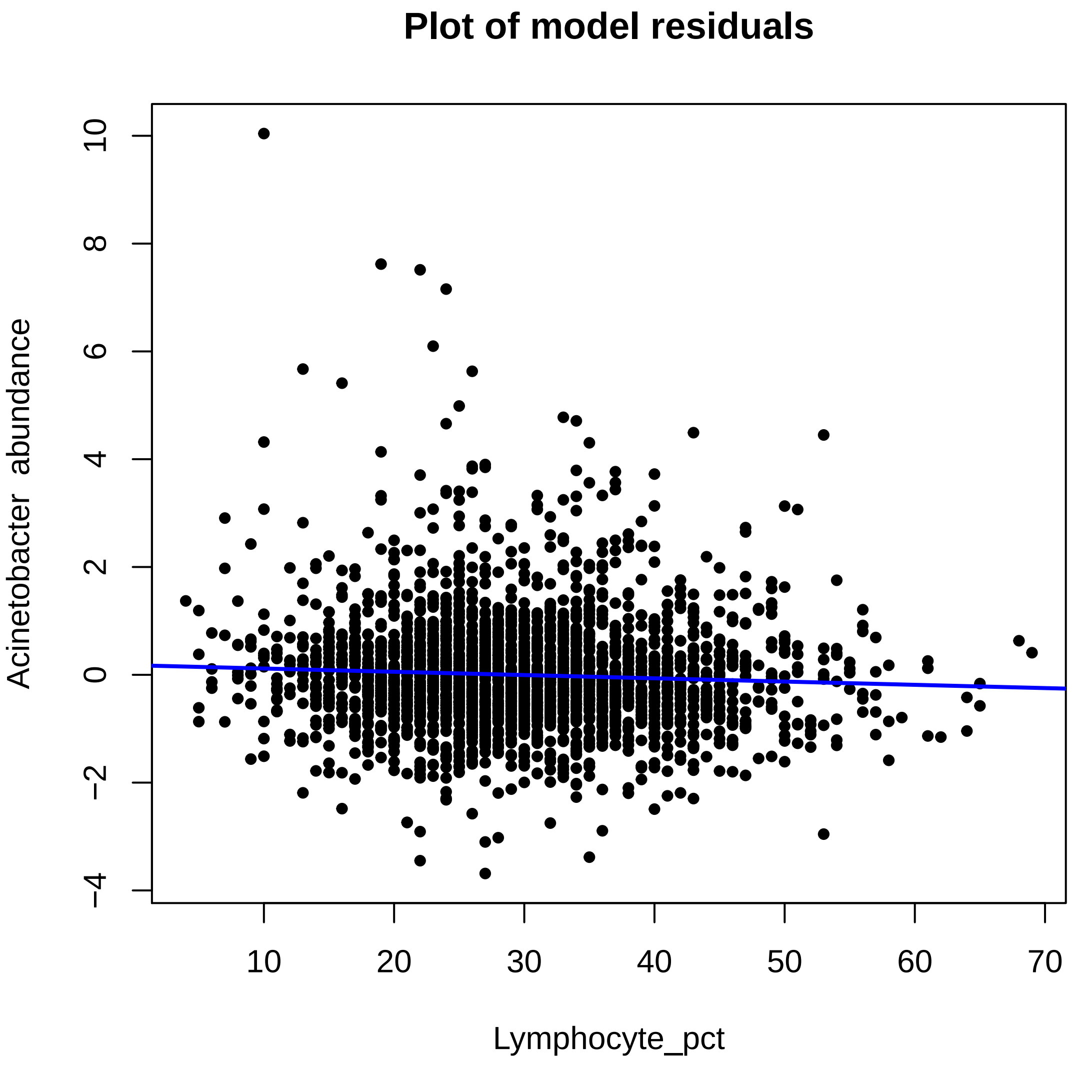

Plot of model residuals
Acinetobacter abundance
Lymphocyte_pct
10
8
6
4
2
0
−2
−4
10
20
30
40
50
60
70

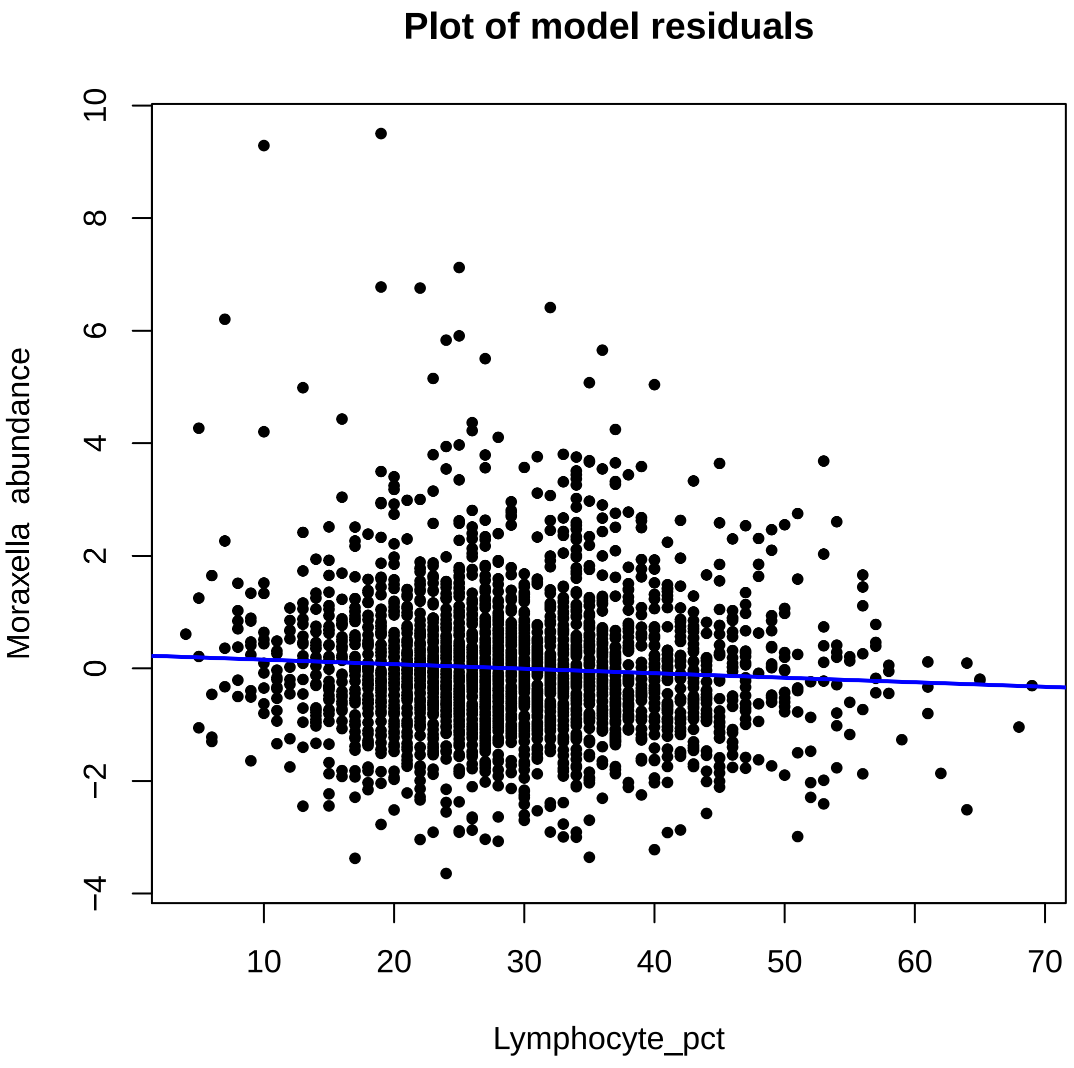
Plot of model residuals
Moraxella abundance
Lymphocyte_pct
10
8
6
4
2
0
−2
−4
10
20
30
40
50
60
70

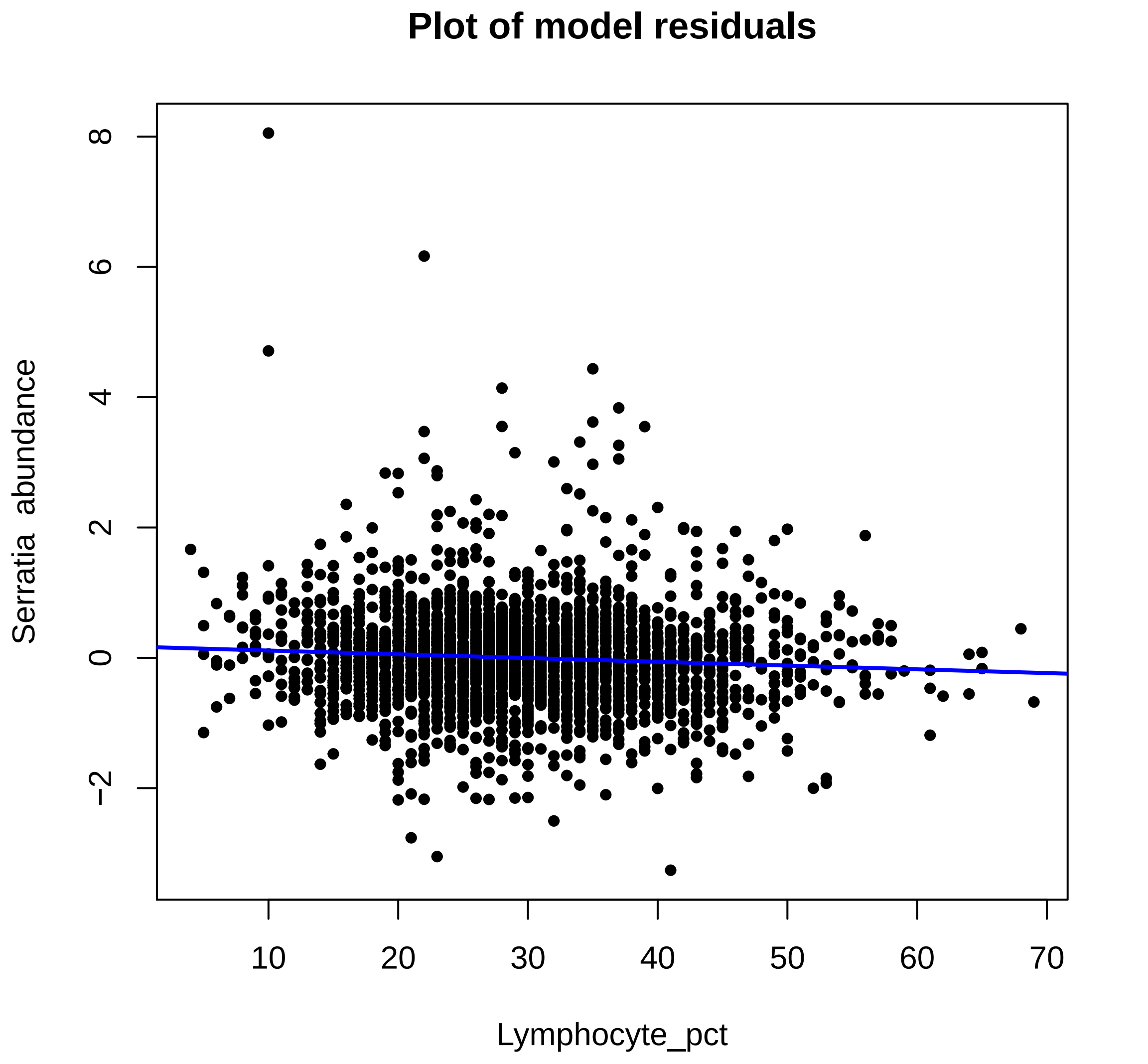
Plot of model residuals
Serratia abundance
8
6
4
2
0
−2
10
20
30
40
50
60
70
Lymphocyte_pct

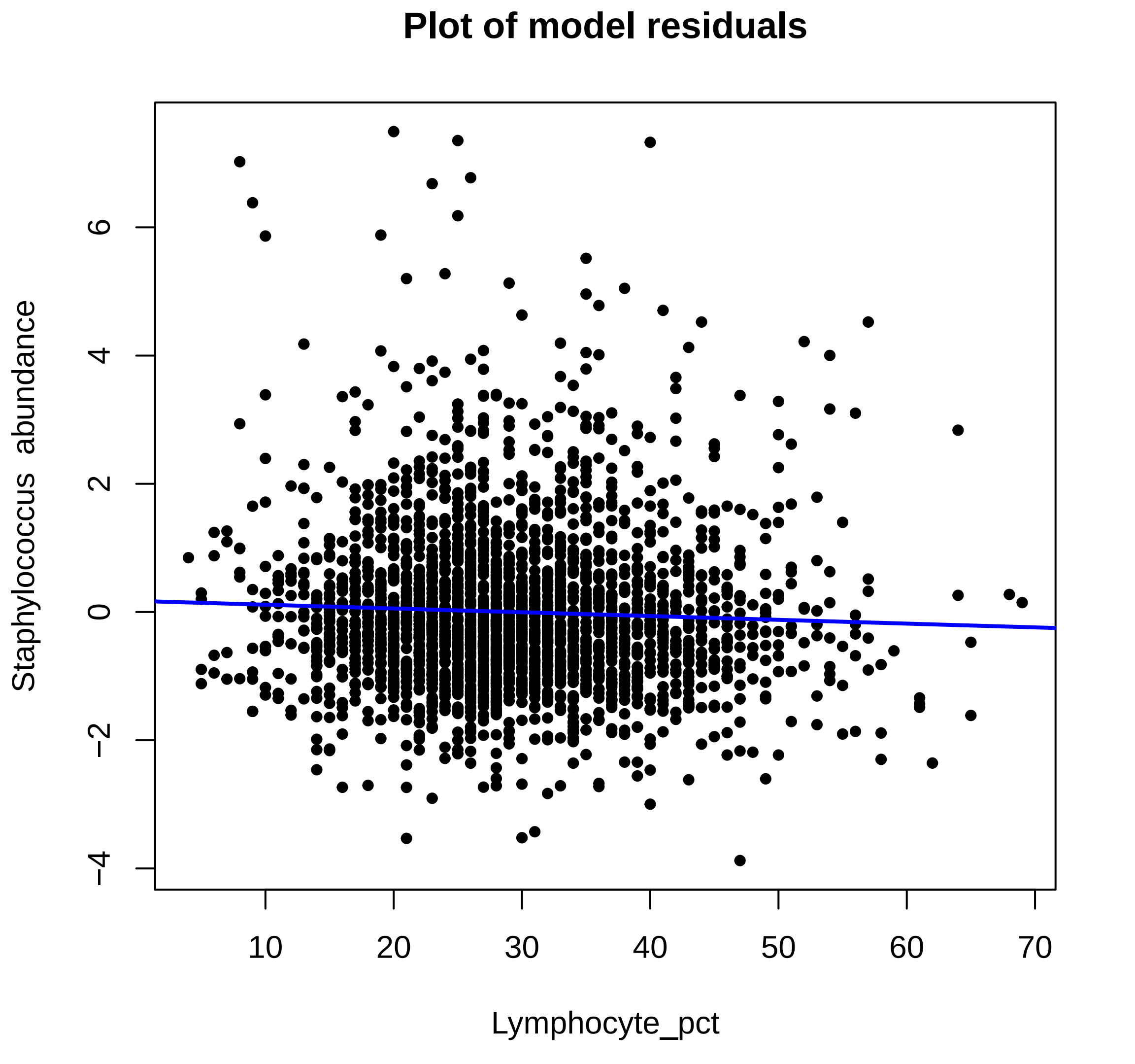

Plot of model residuals
Staphylococcus abundance
Lymphocyte_pct
−4
−2
0
2
4
6
10
20
30
40
50
60
70

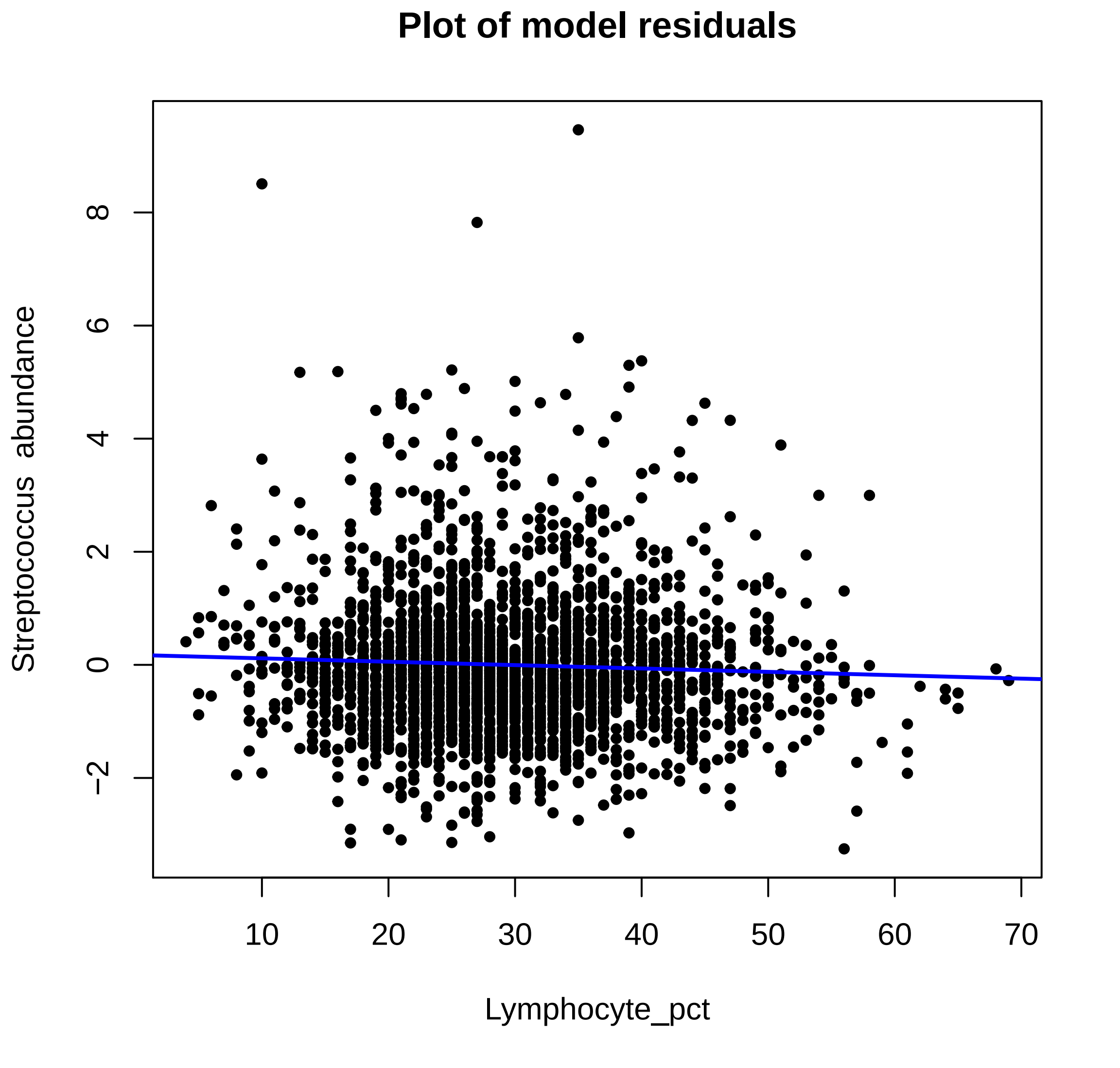
Plot of model residuals
Streptococcus abundance
Lymphocyte_pct
−2
0
2
4
6
8
10
20
30
40
50
60
70

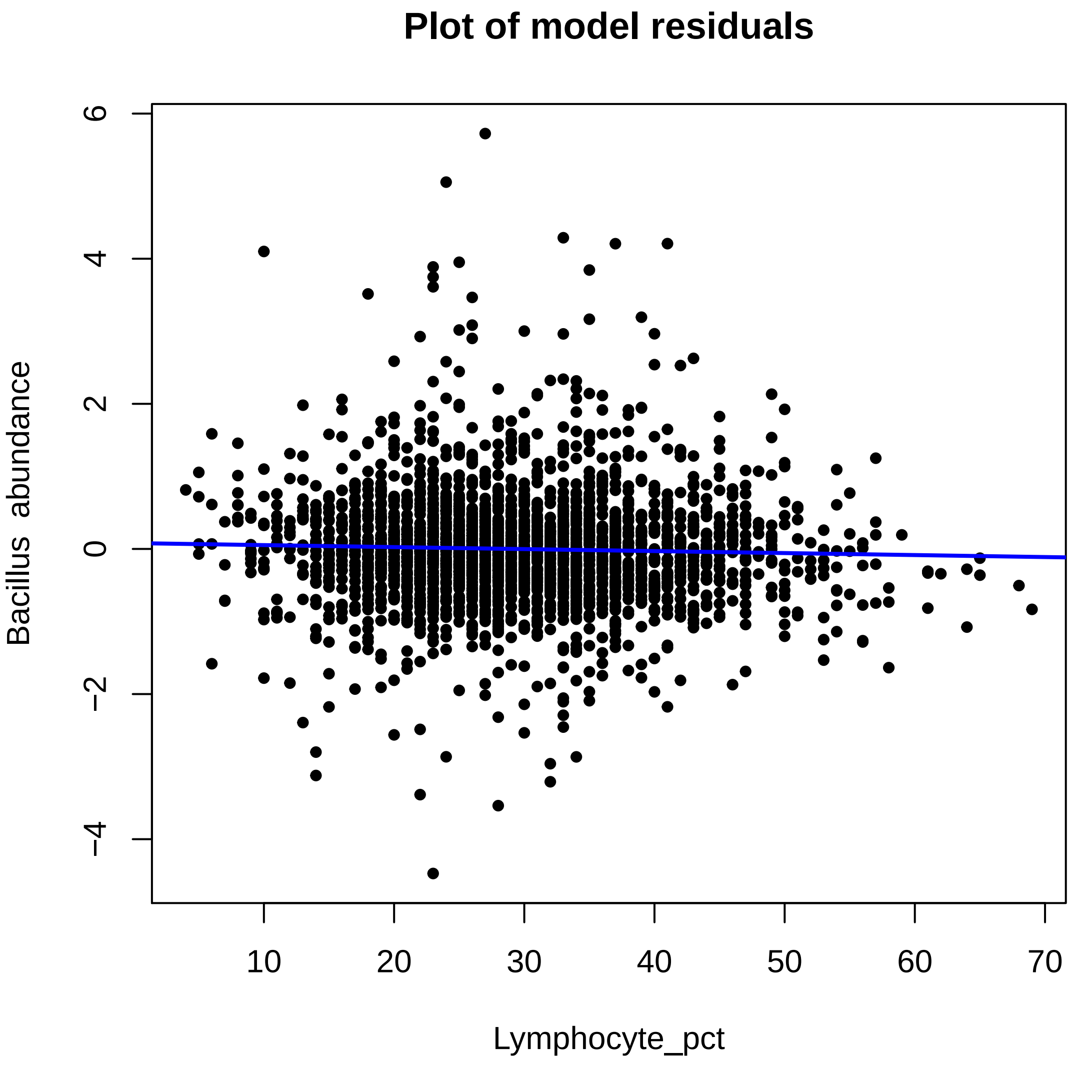

Plot of model residuals
Bacillus abundance
Lymphocyte_pct
6
4
2
0
−2
−4
10
20
30
40
50
60
70

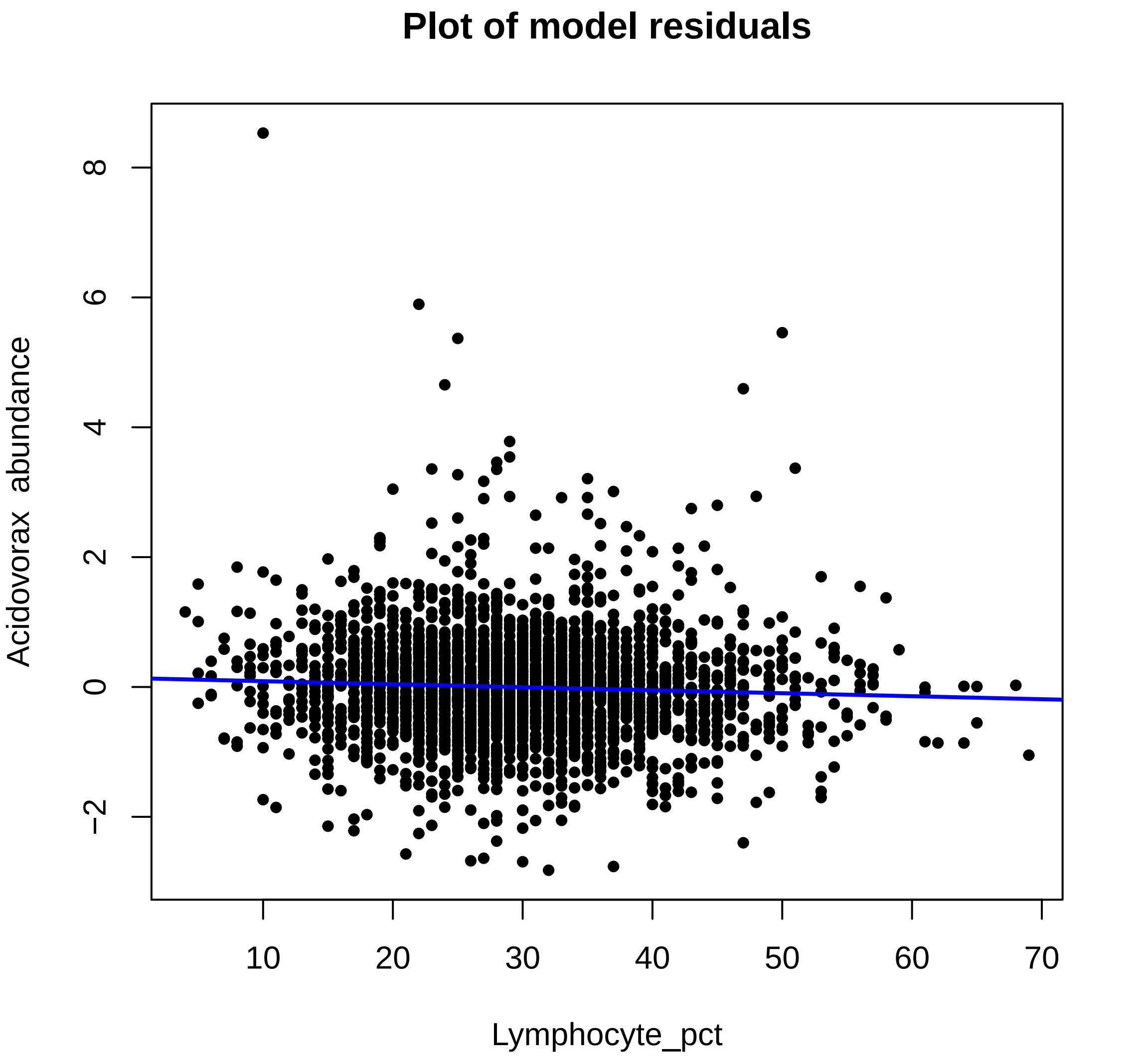
Plot of model residuals
Acidovorax abundance
Lymphocyte_pct
−2
0
2
4
6
8
10
20
30
40
50
60
70

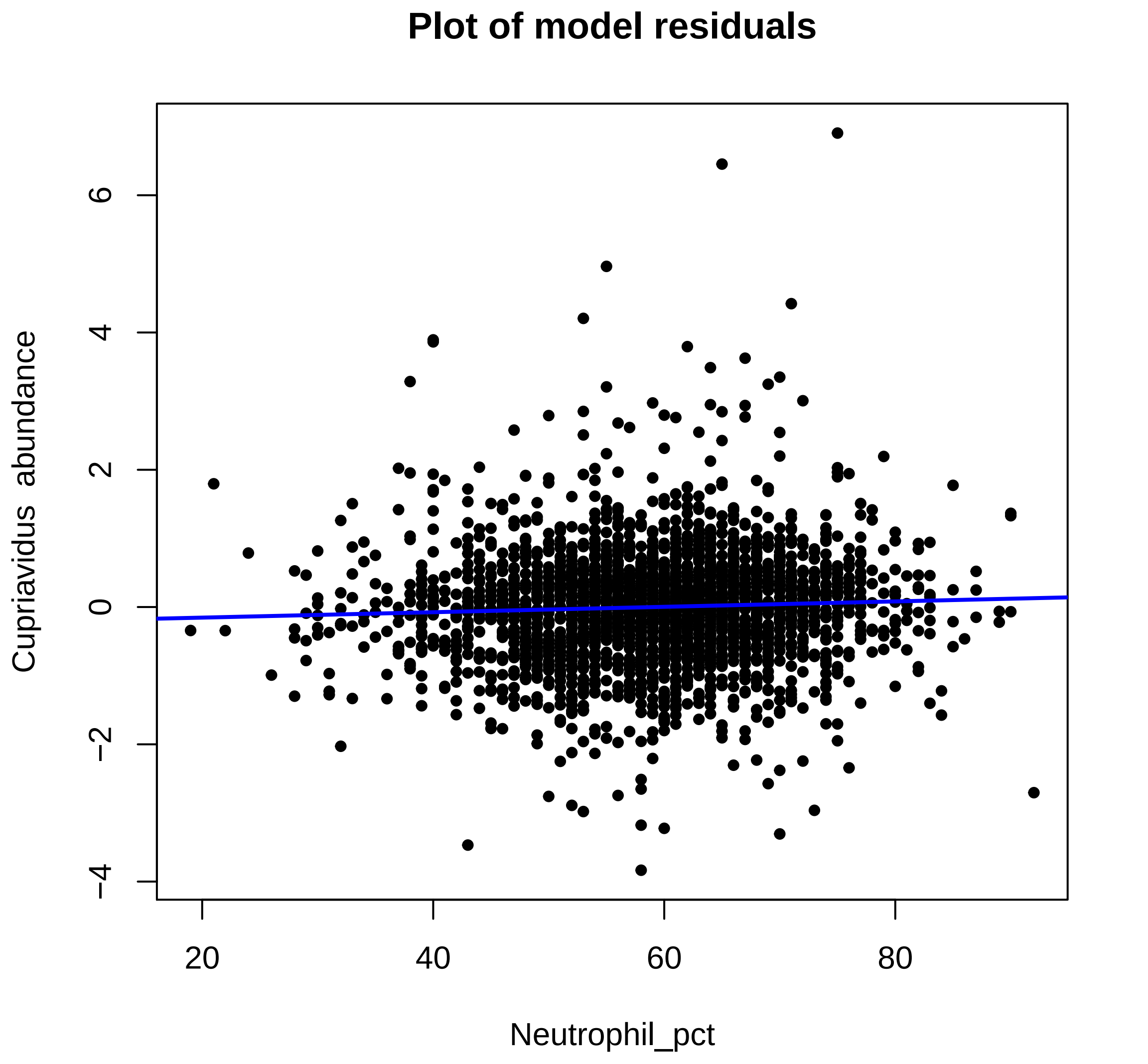
Plot of model residuals
Cupriavidus abundance
6
4
2
0
−2
−4
20
40
60
80
Neutrophil_pct

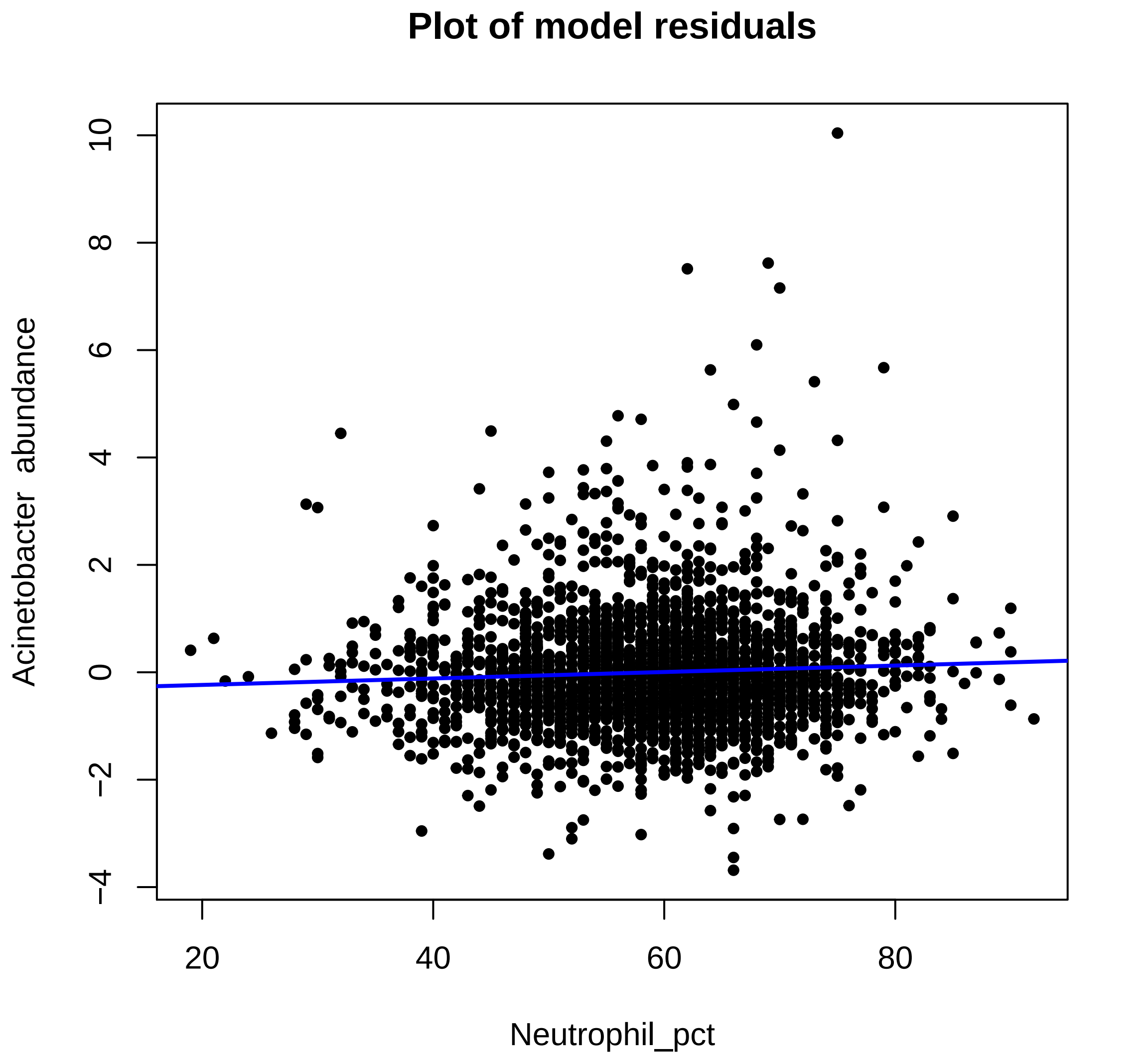
Plot of model residuals
Acinetobacter abundance
Neutrophil_pct
−4
−2
0
2
4
6
8
10
20
40
60
80

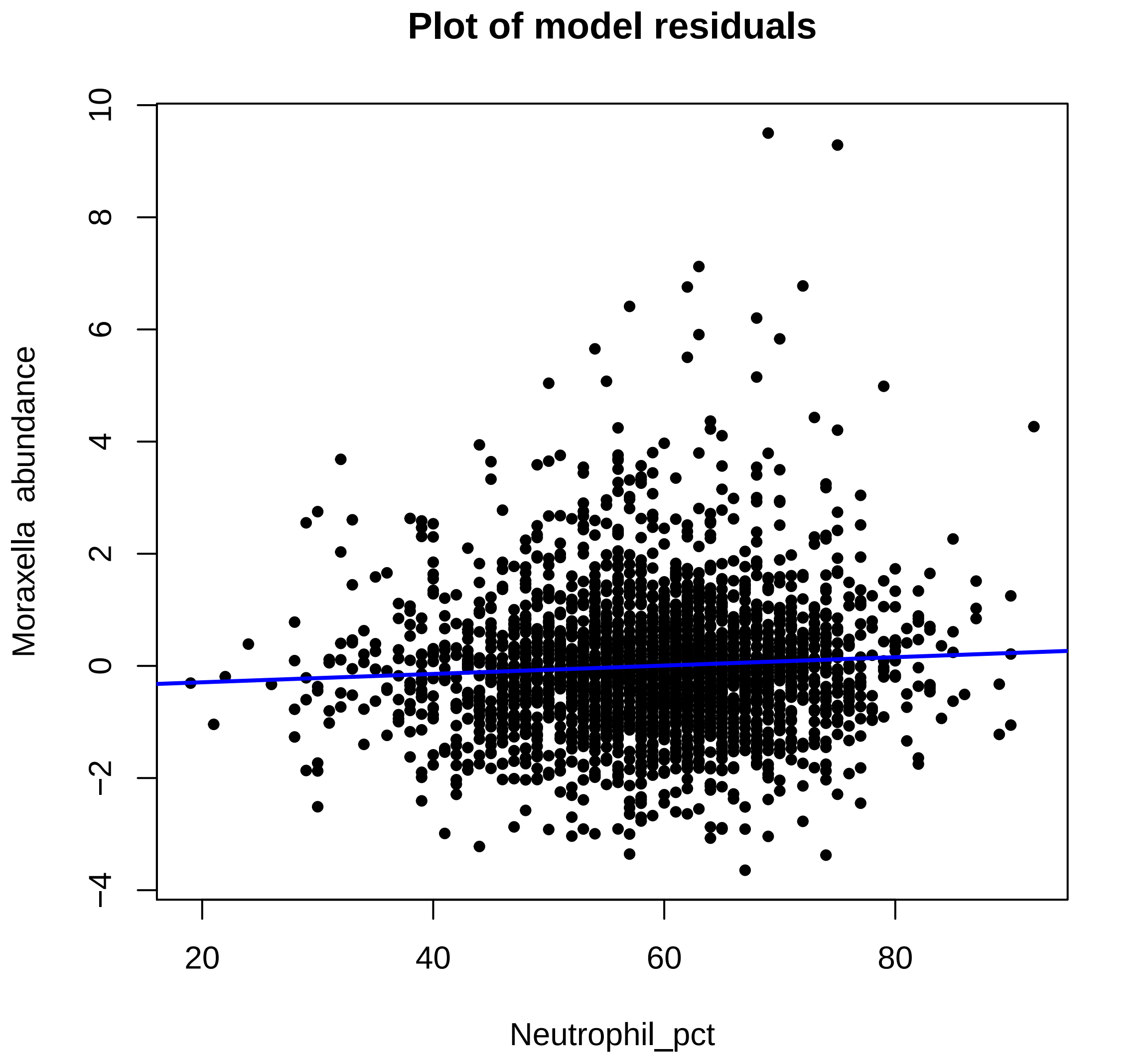
Plot of model residuals
Moraxella abundance
Neutrophil_pct
10
8
6
4
2
0
−2
−4
20
40
60
80

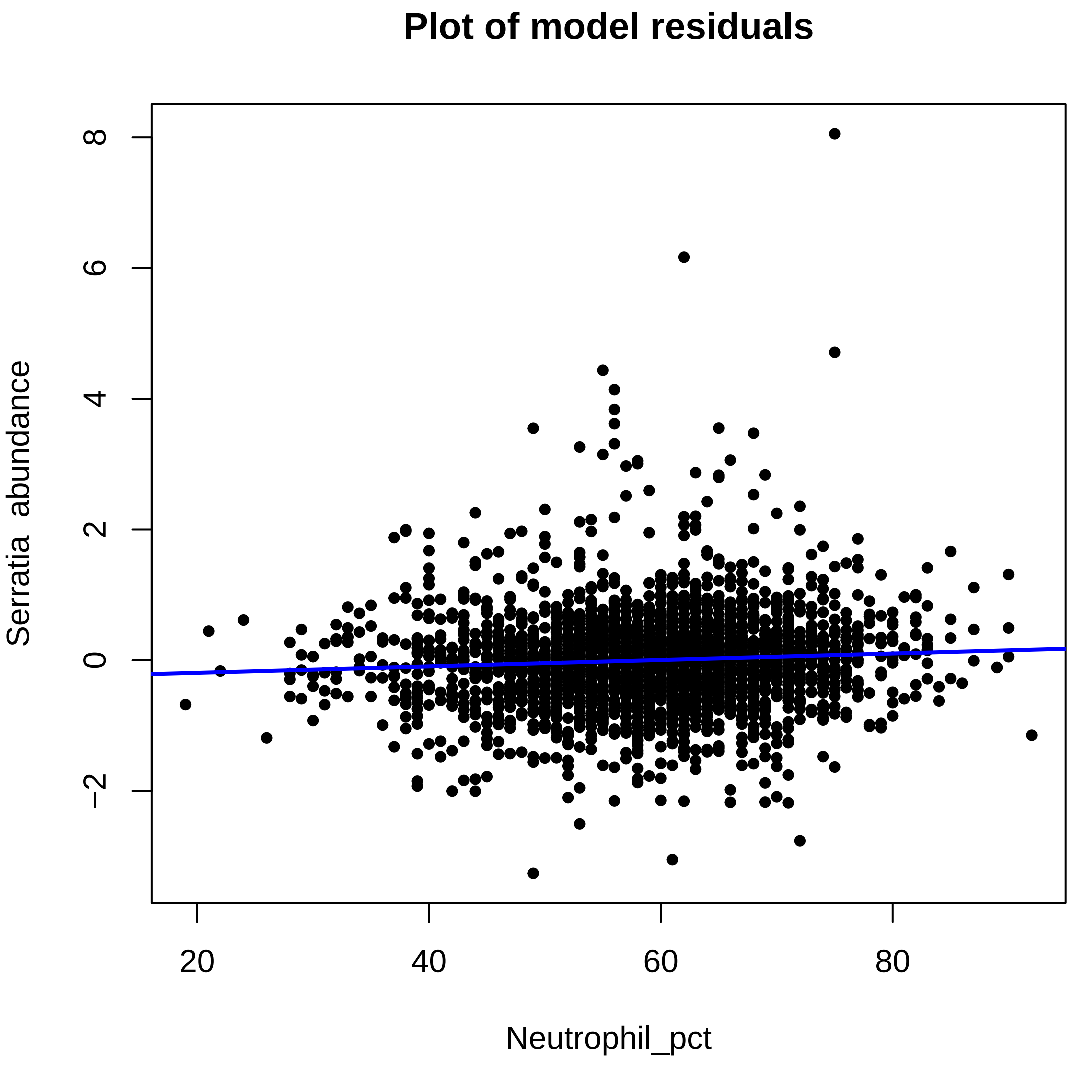
Plot of model residuals
Serratia abundance
Neutrophil_pct
8
6
4
2
0
−2
20
40
60
80

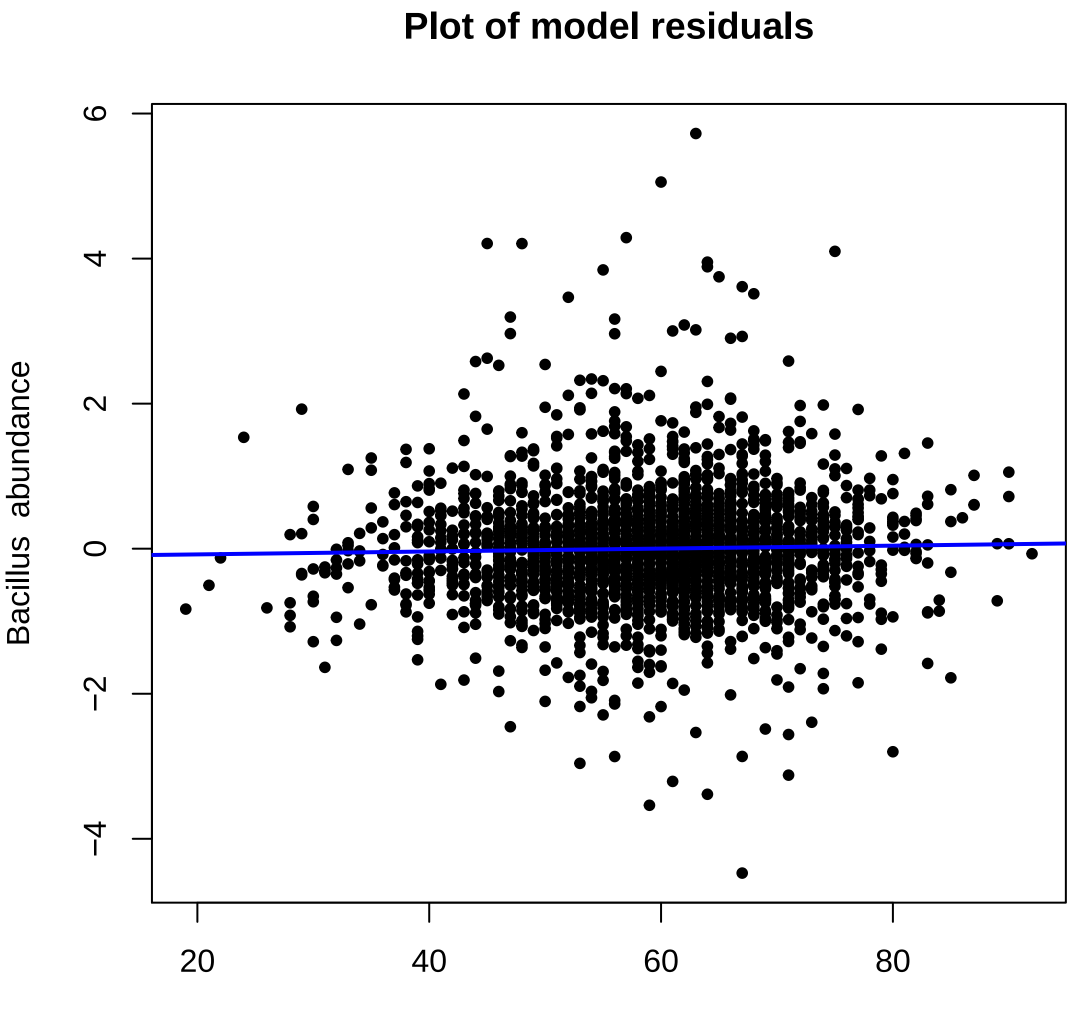
Plot of model residuals
Bacillus abundance
Neutrophil_pct
−4
−2
0
2
4
6
20
40
60
80

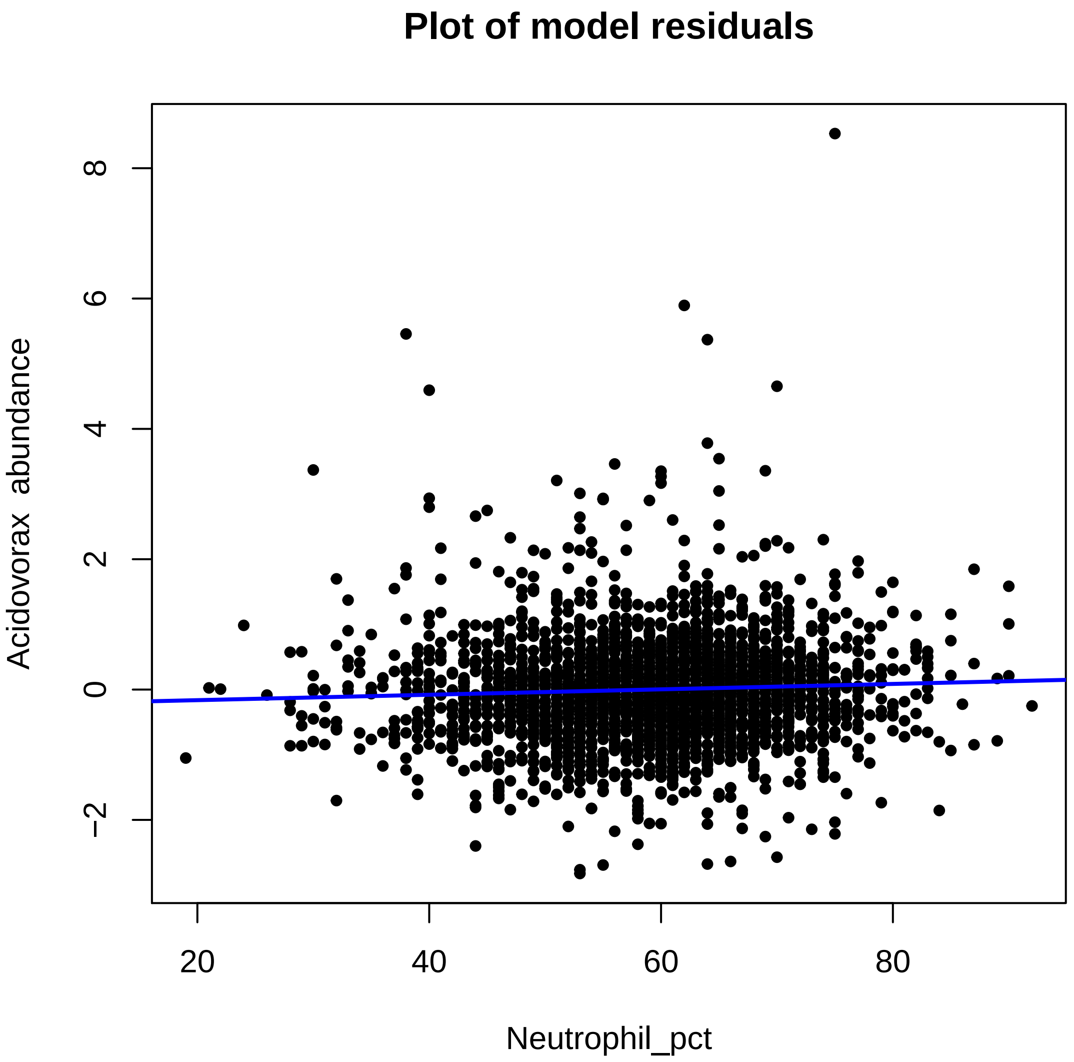

Plot of model residuals
Acidovorax abundance
Neutrophil_pct
−2
0
2
4
6
8
20
40
60
80

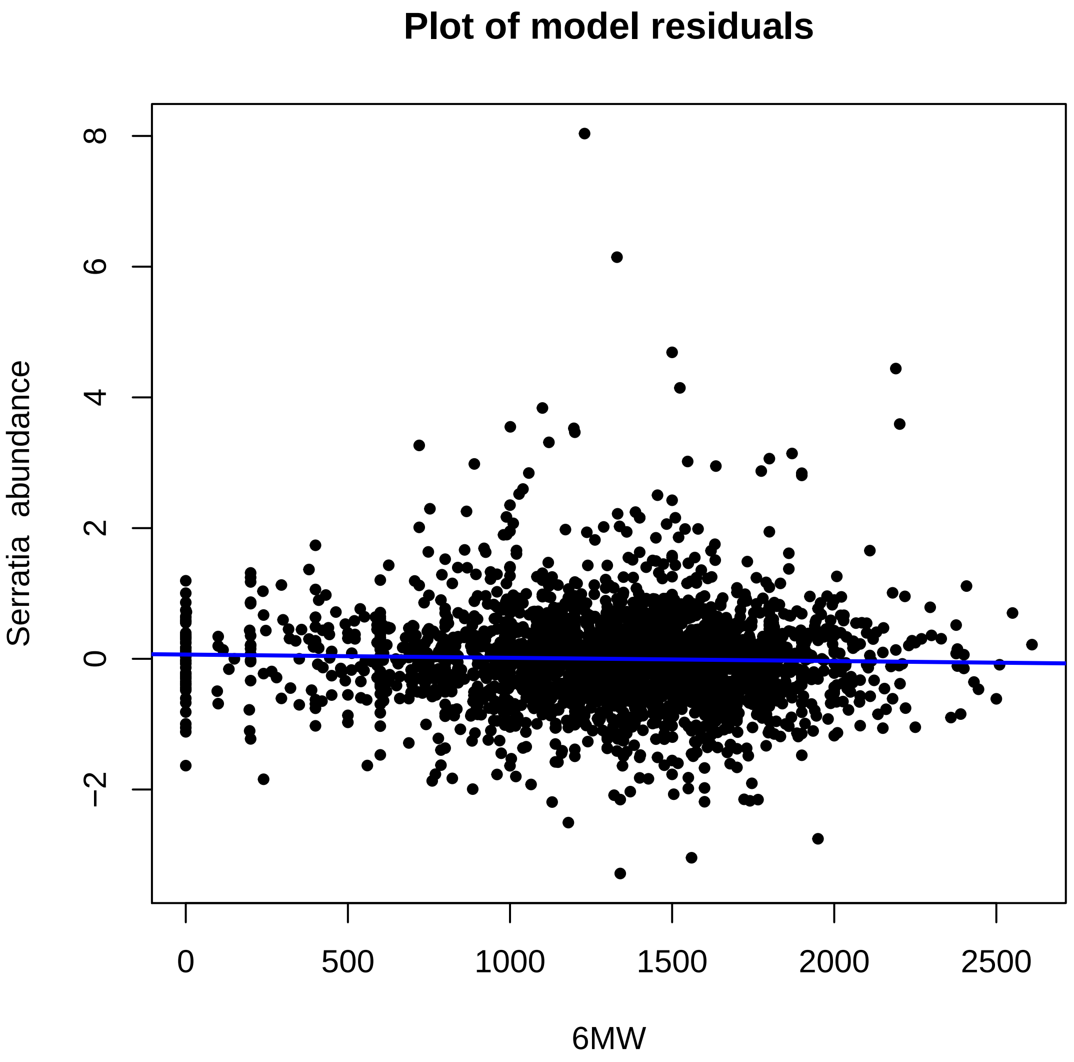

Plot of model residuals
Serratia abundance
6MW
8
6
4
2
0
−2
0
500
1000
1500
2000
2500

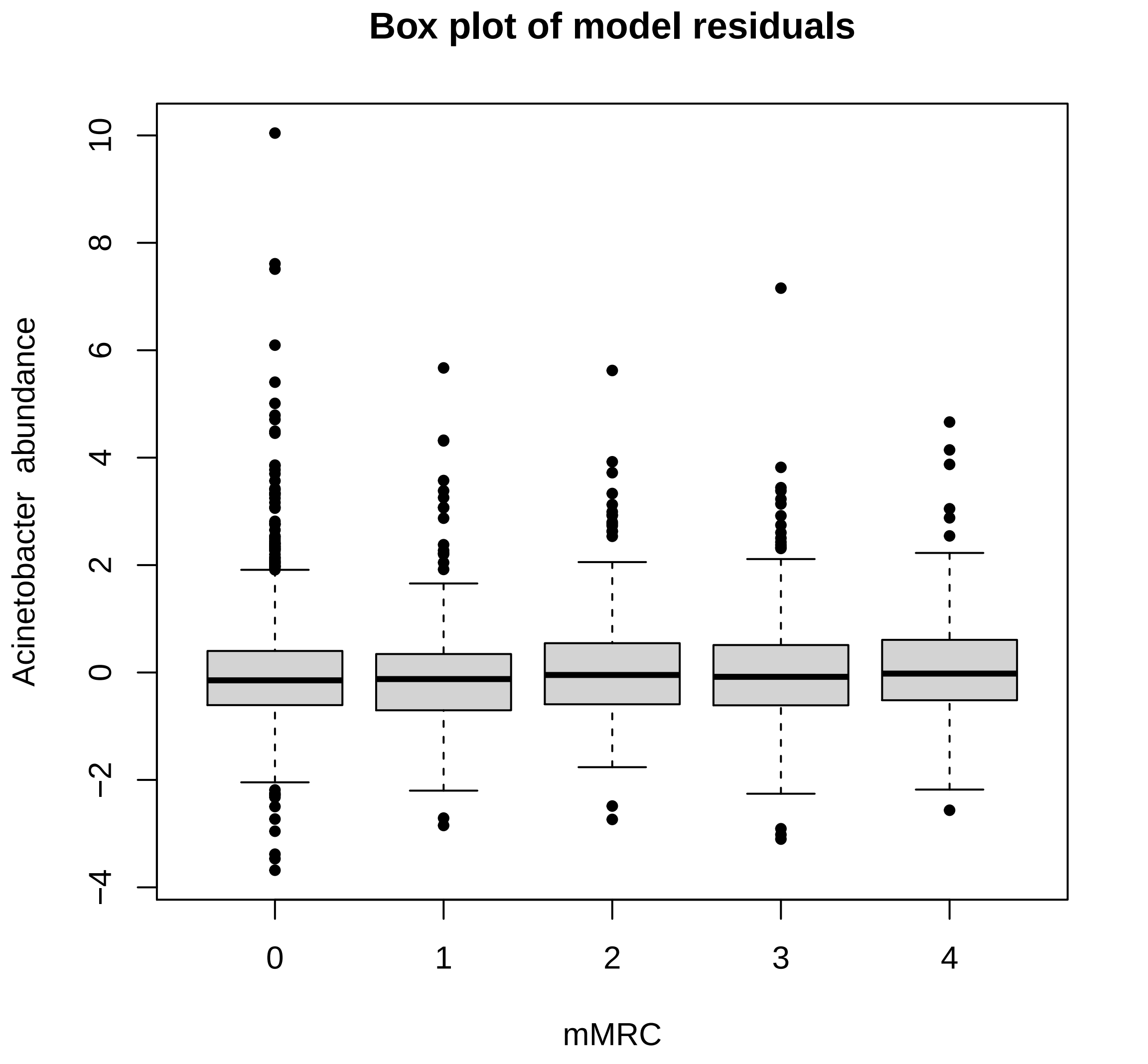
Box plot of model residuals
Acinetobacter abundance
10
8
6
4
2
0
−2
−4
0
1
2
3
4
mMRC

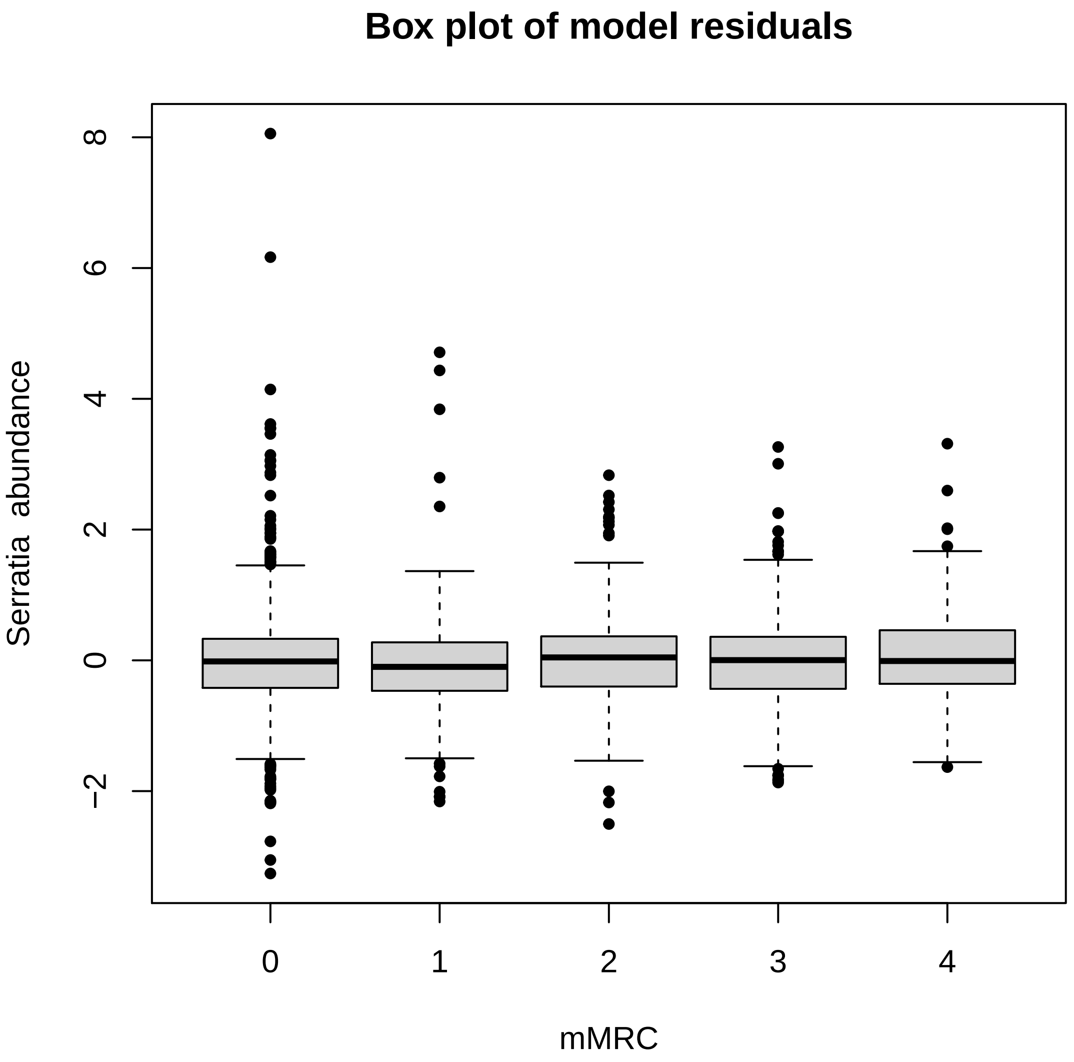
Box plot of model residuals
Serratia abundance
mMRC
8
6
4
2
0
−2
0
1
2
3
4

Box plot of model residuals

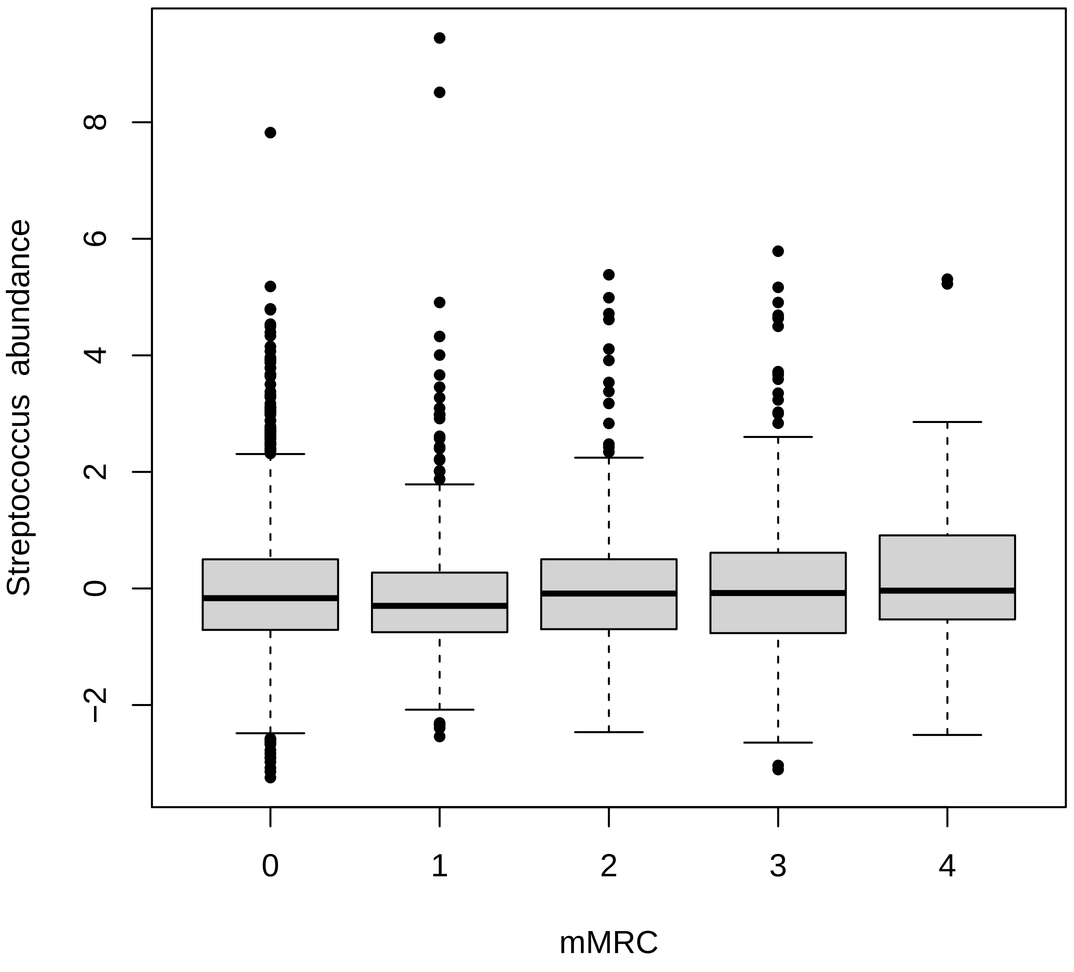

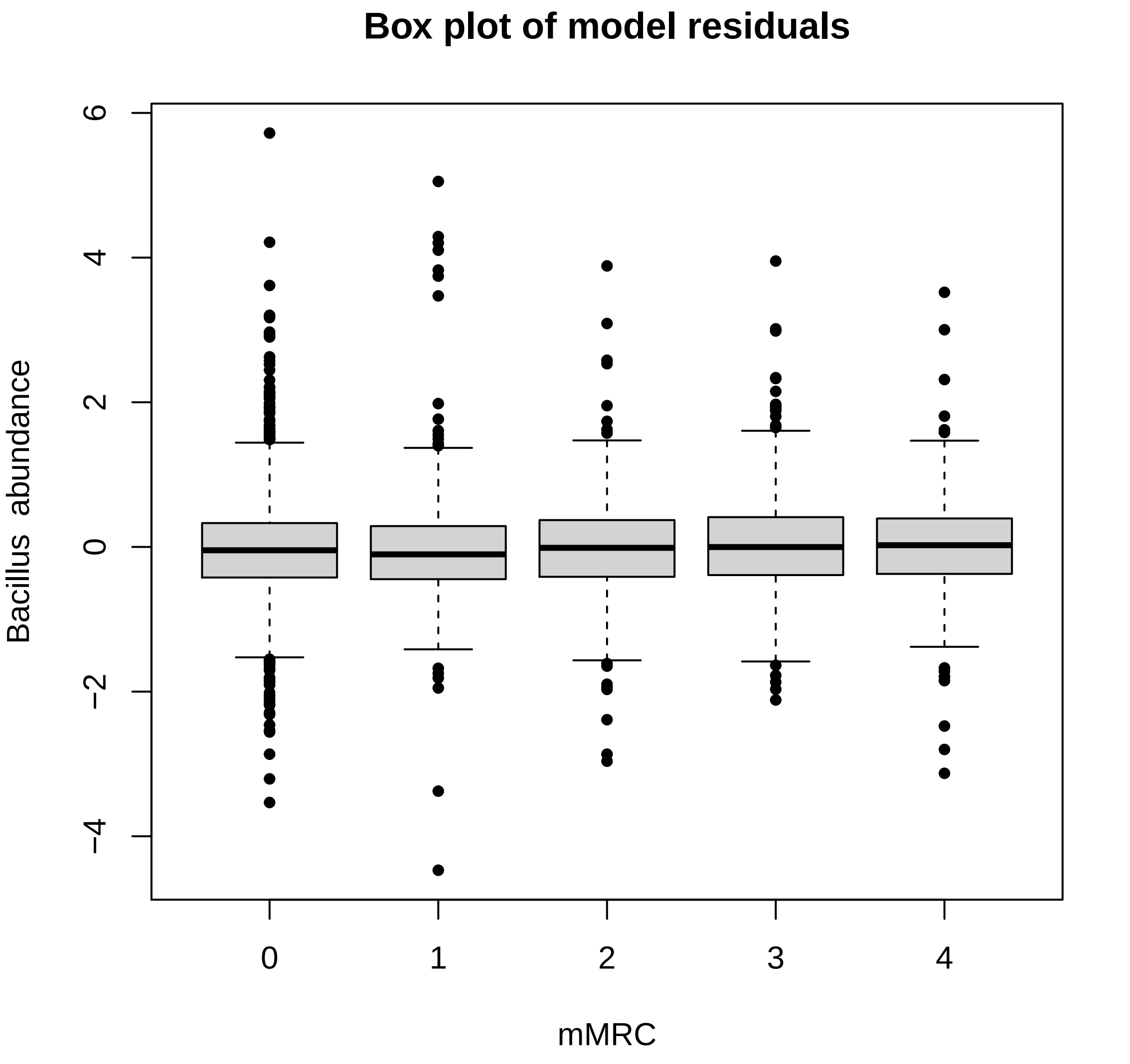
Box plot of model residuals
Bacillus abundance
6
4
2
0
−2
−4
0
1
2
3
4
mMRC

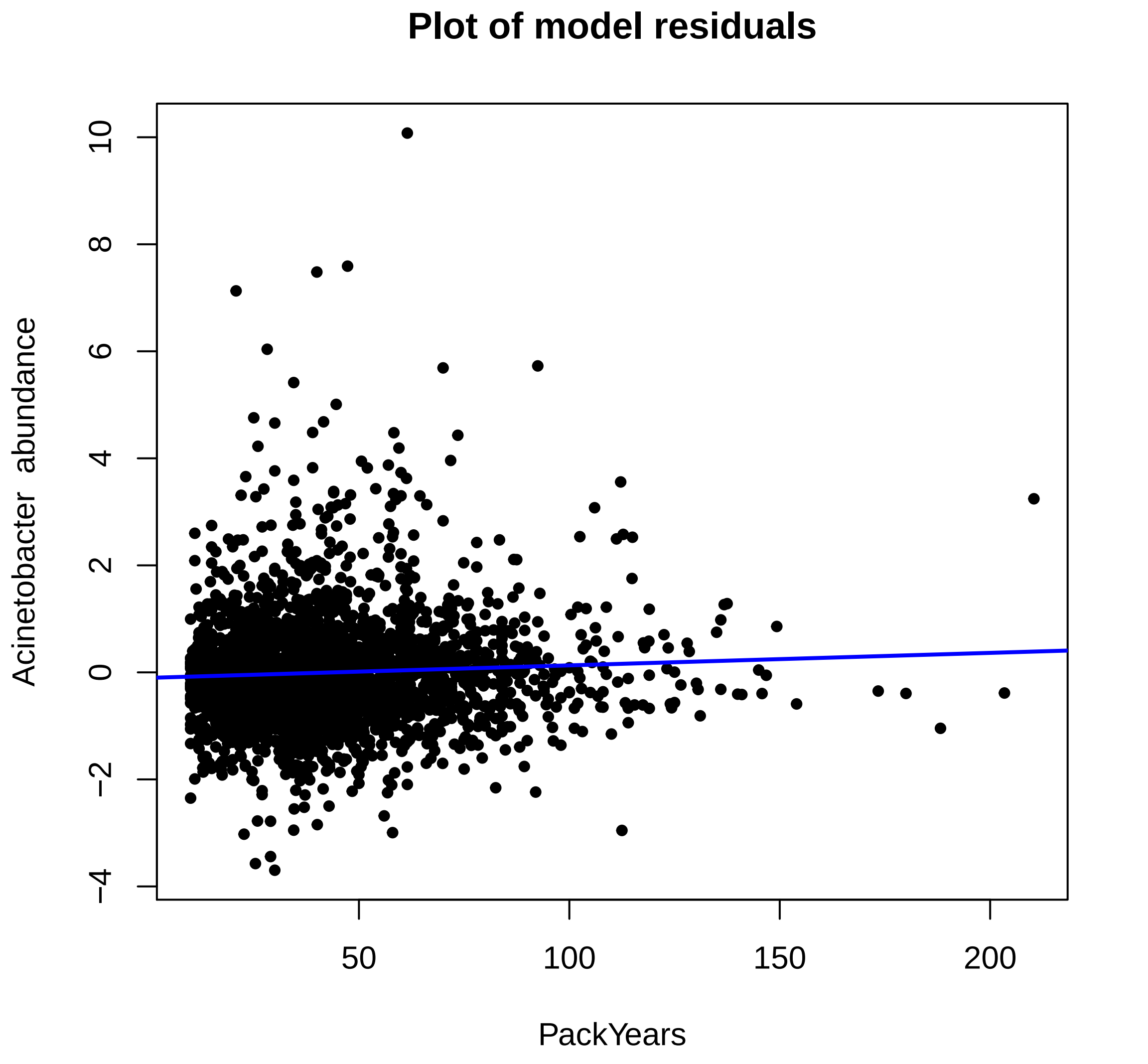
Plot of model residuals
Acinetobacter abundance
PackYears
10
8
6
4
2
0
−2
−4
50
100
150
200

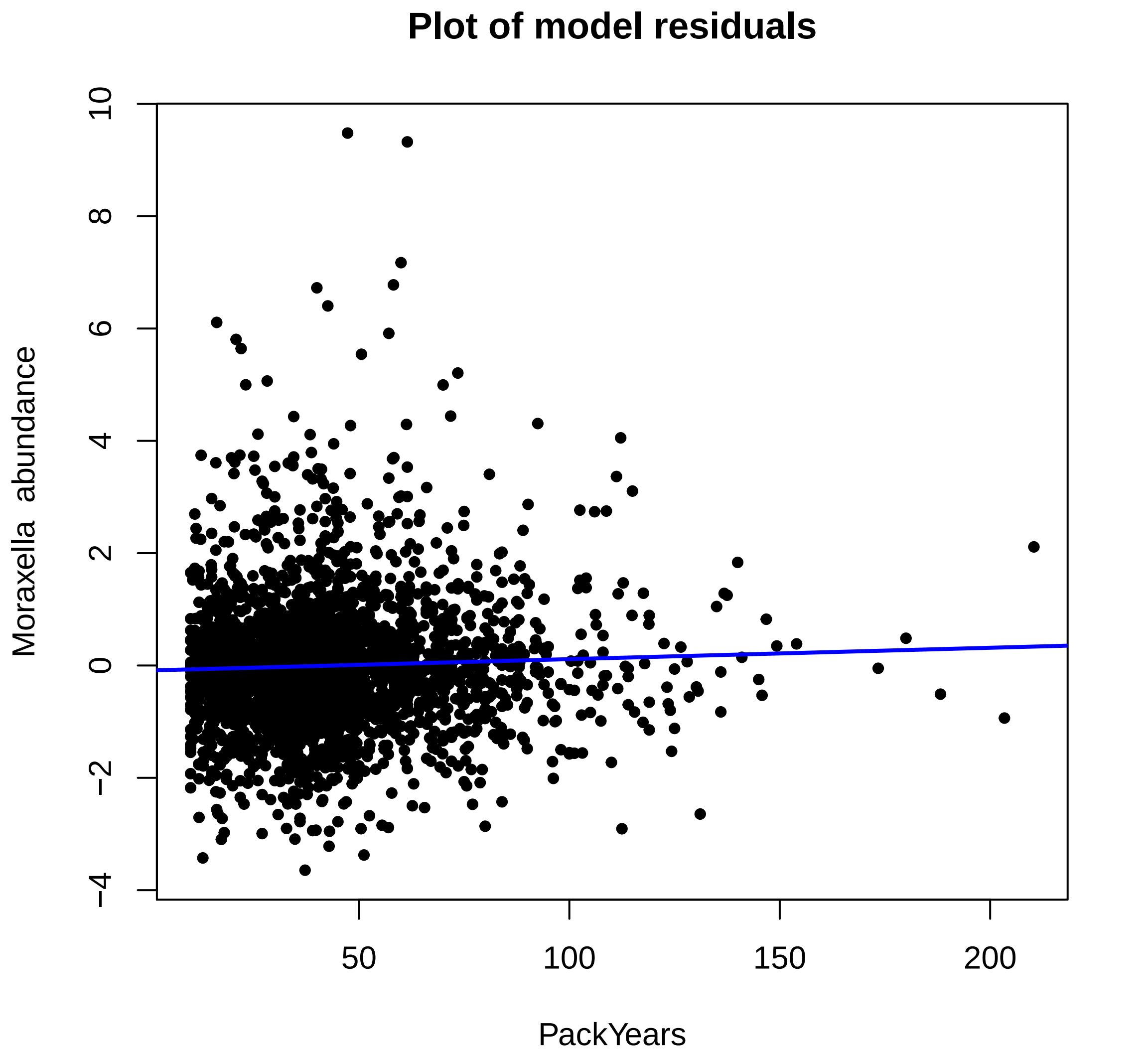
Plot of model residuals
Moraxella abundance
PackYears
10
8
6
4
2
0
−2
−4
50
100
150
200

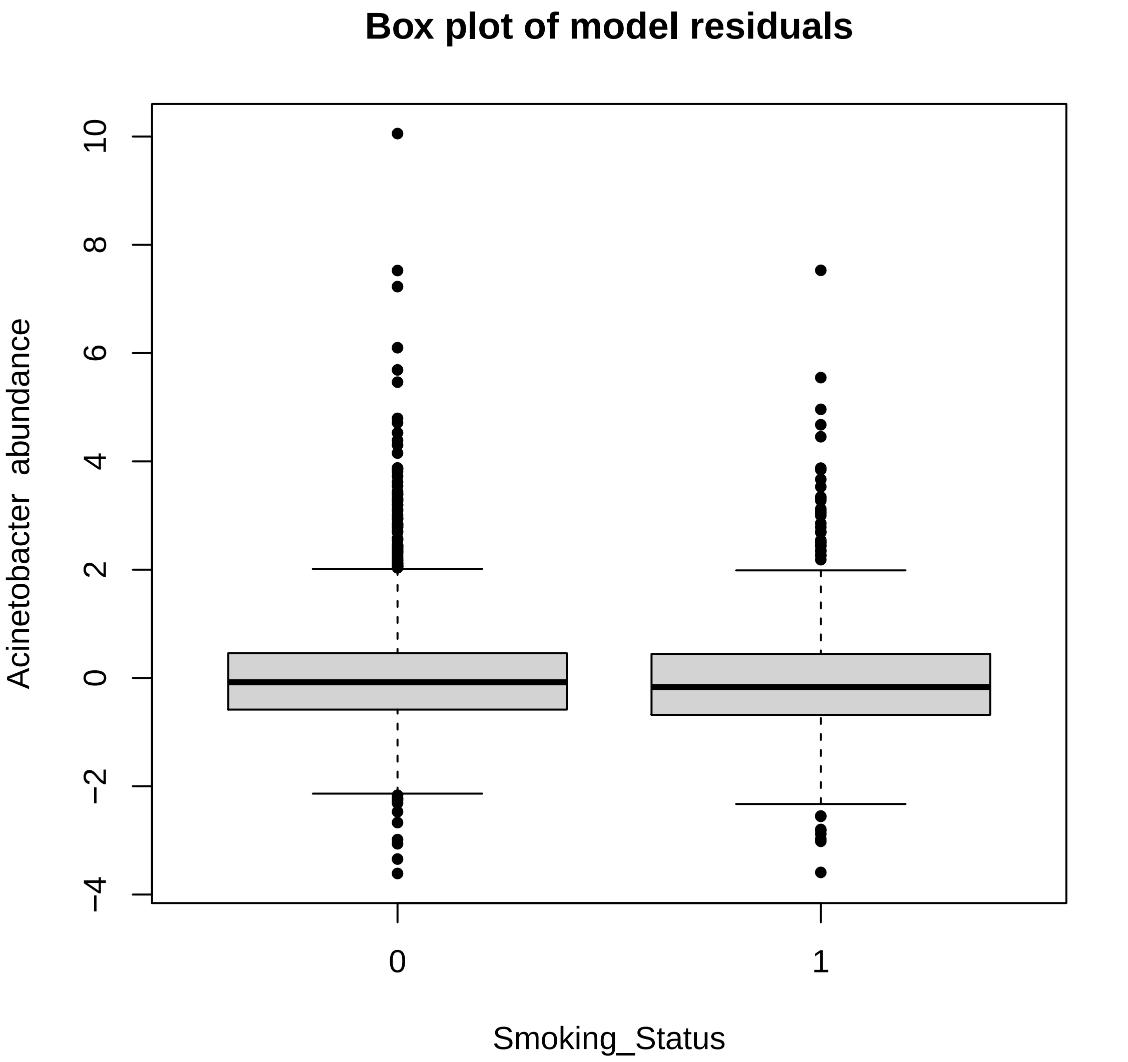
Box plot of model residuals
Acinetobacter abundance
10
8
6
4
2
0
−2
−4
0
1
Smoking_Status

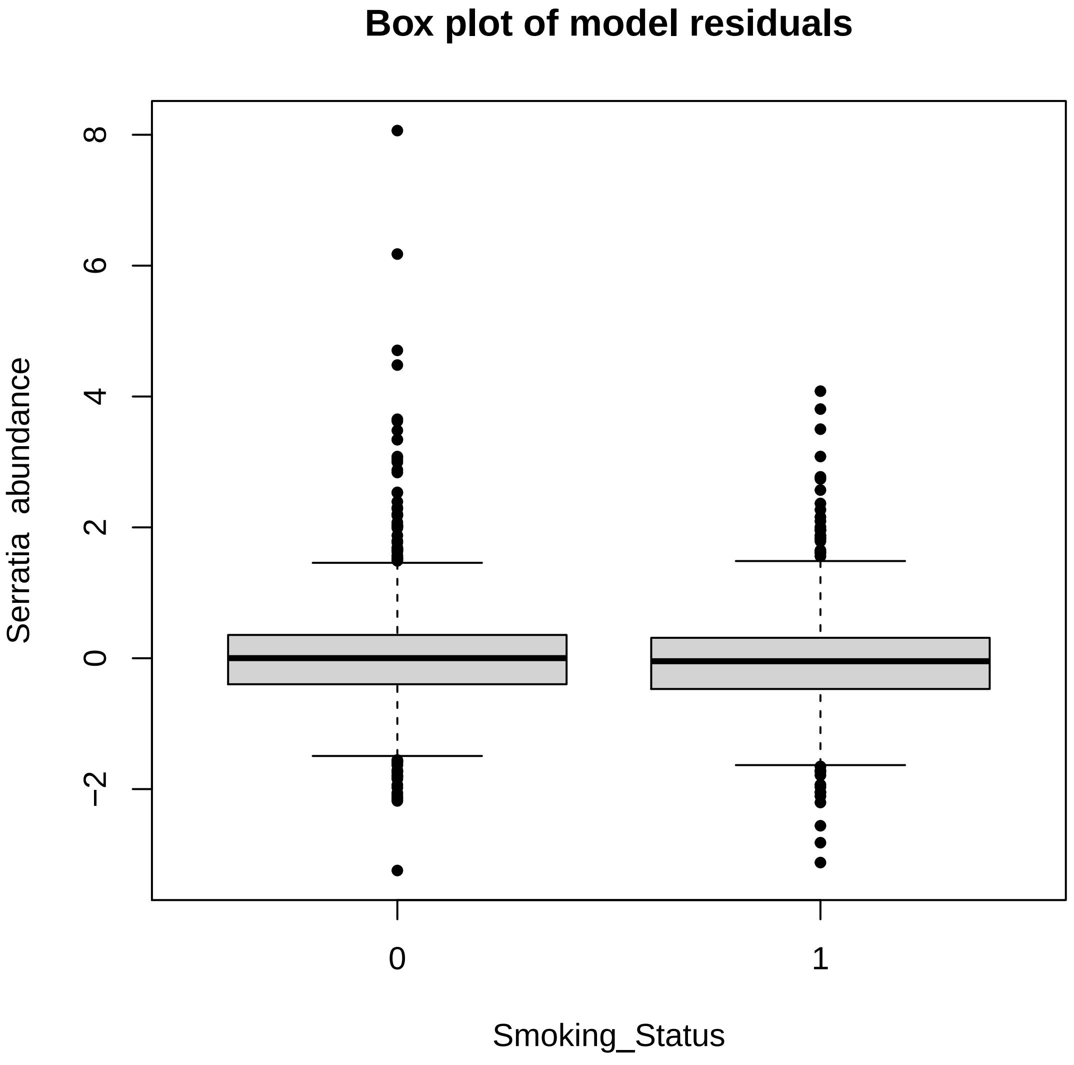

Box plot of model residuals
Serratia abundance
8
6
4
2
0
−2
0
1
Smoking_Status

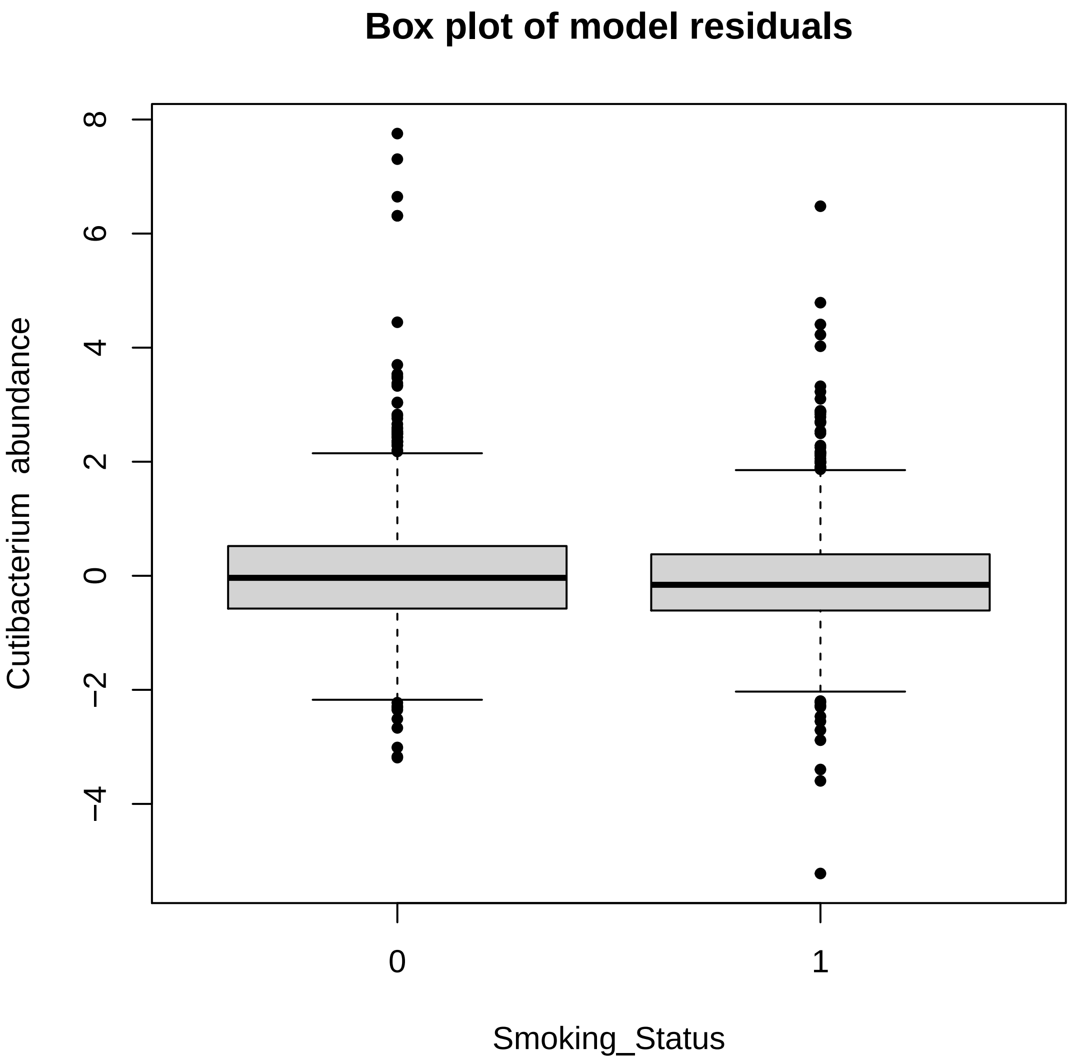
Box plot of model residuals
Cutibacterium abundance
8
6
4
2
0
−2
−4
0
1
Smoking_Status

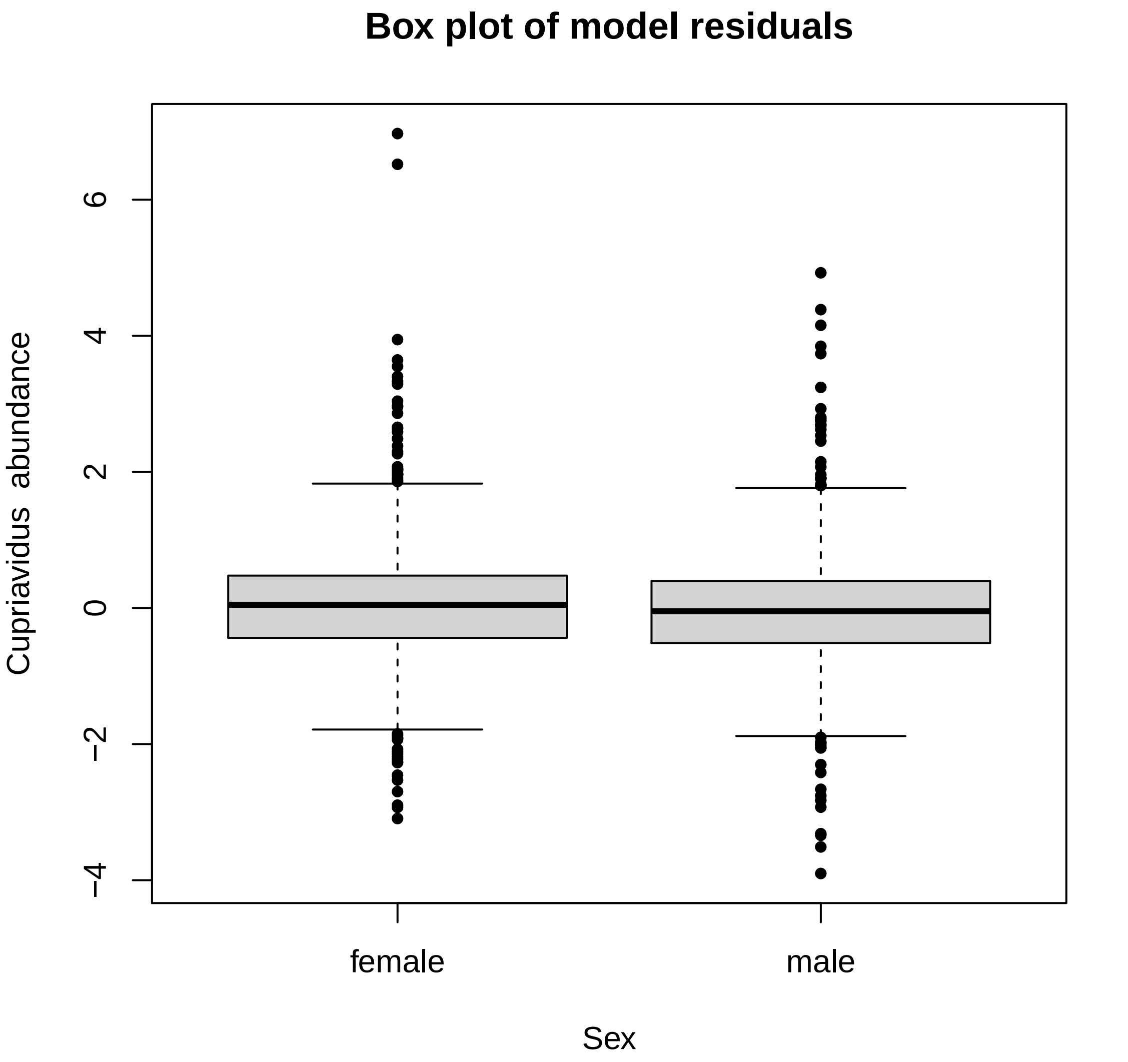
Box plot of model residuals
Cupriavidus abundance
6
4
2
0
−2
−4
female
male
Sex

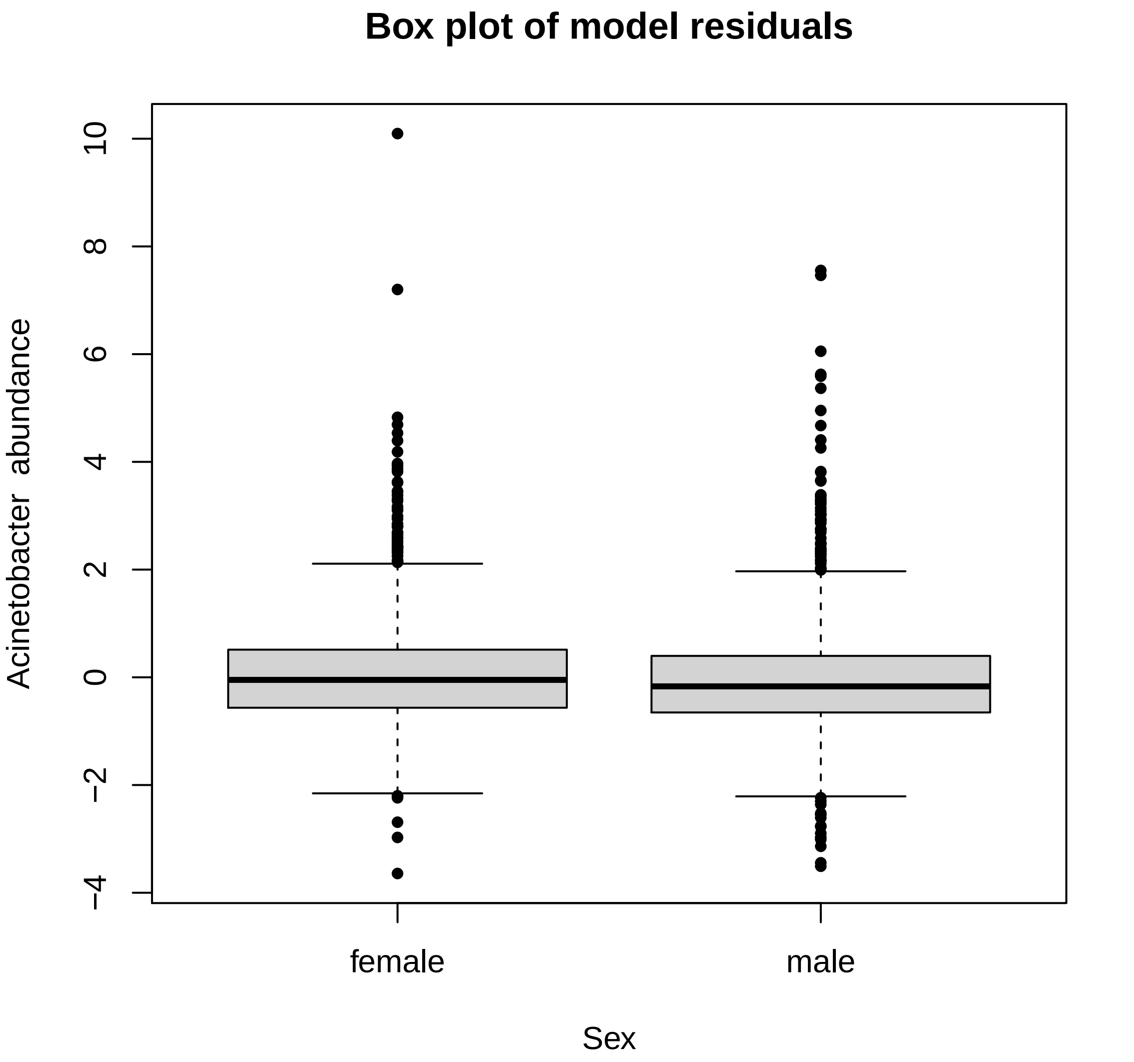

Box plot of model residuals
Acinetobacter abundance
10
8
6
4
2
0
−2
−4
female
male
Sex

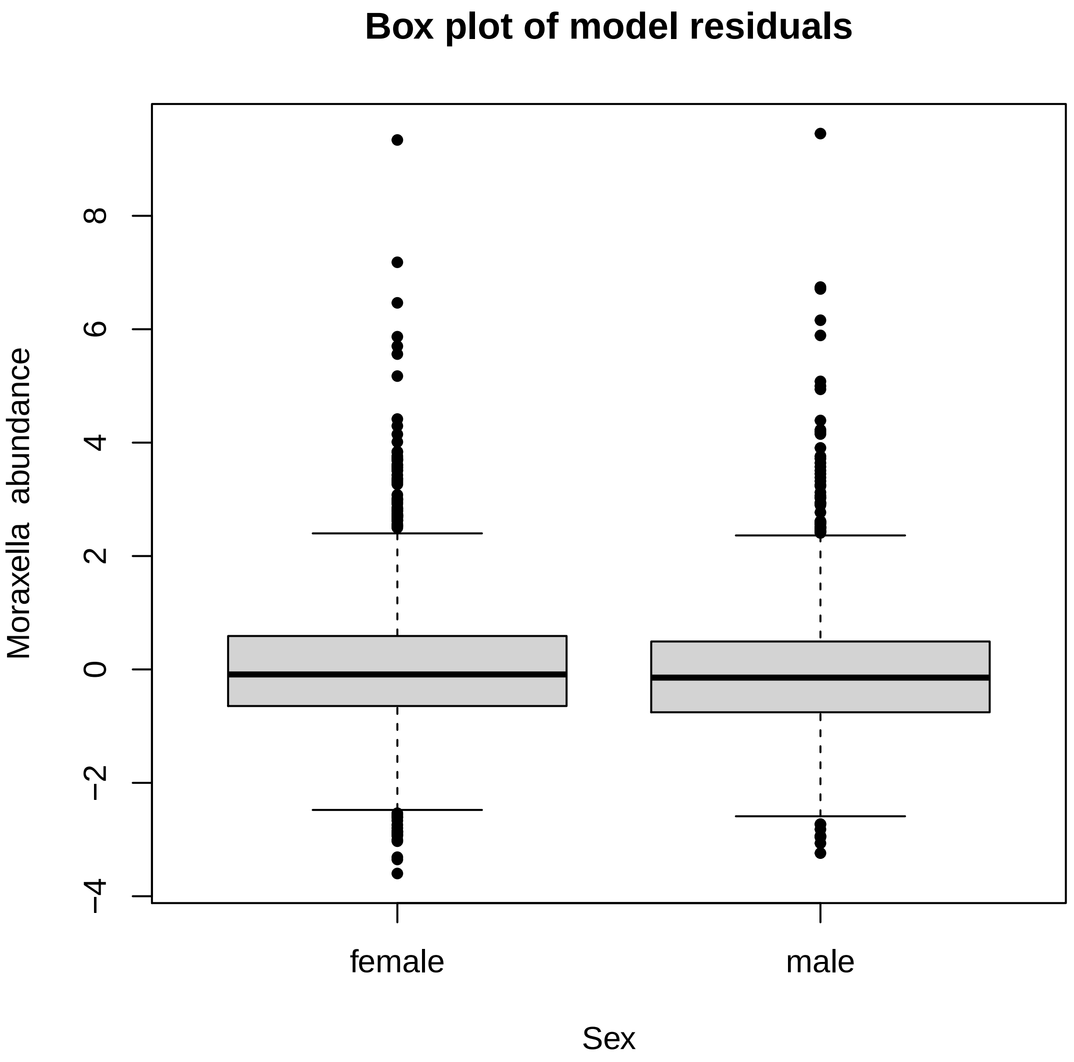

Box plot of model residuals
Moraxella abundance
8
6
4
2
0
−2
−4
female
male
Sex

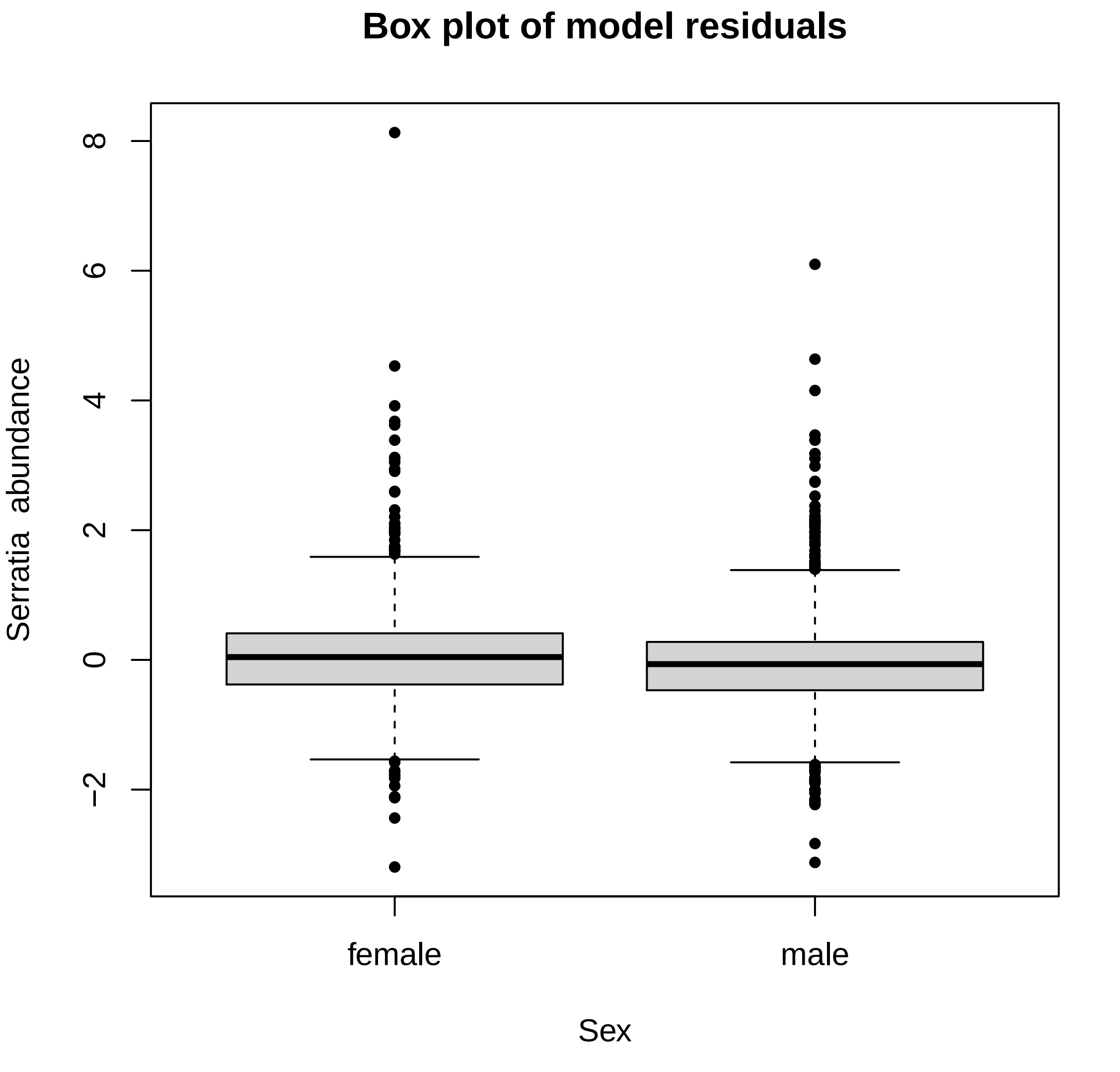
Box plot of model residuals
Serratia abundance
8
6
4
2
0
−2
female
male
Sex

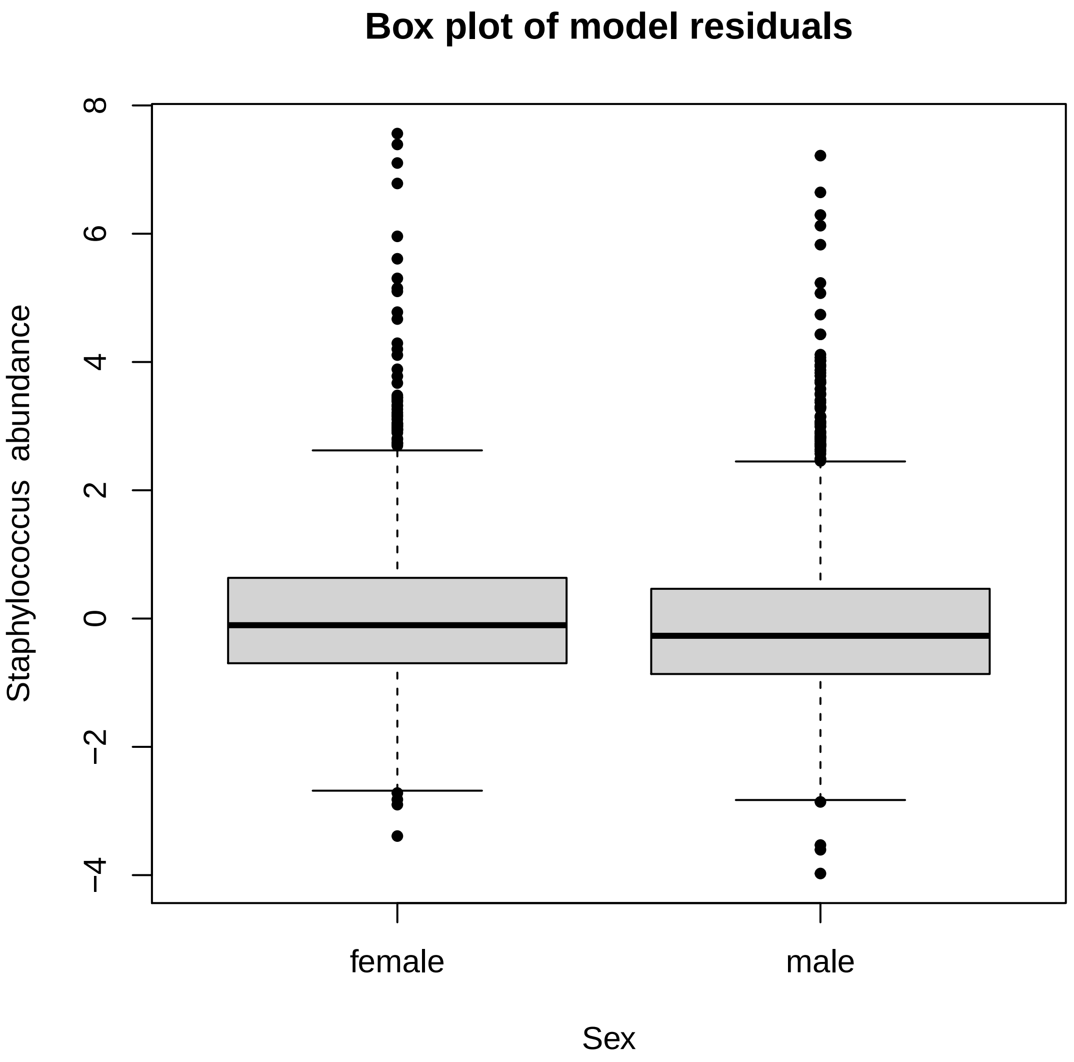
Box plot of model residuals
Staphylococcus abundance
8
6
4
2
0
−2
−4
female
male
Sex

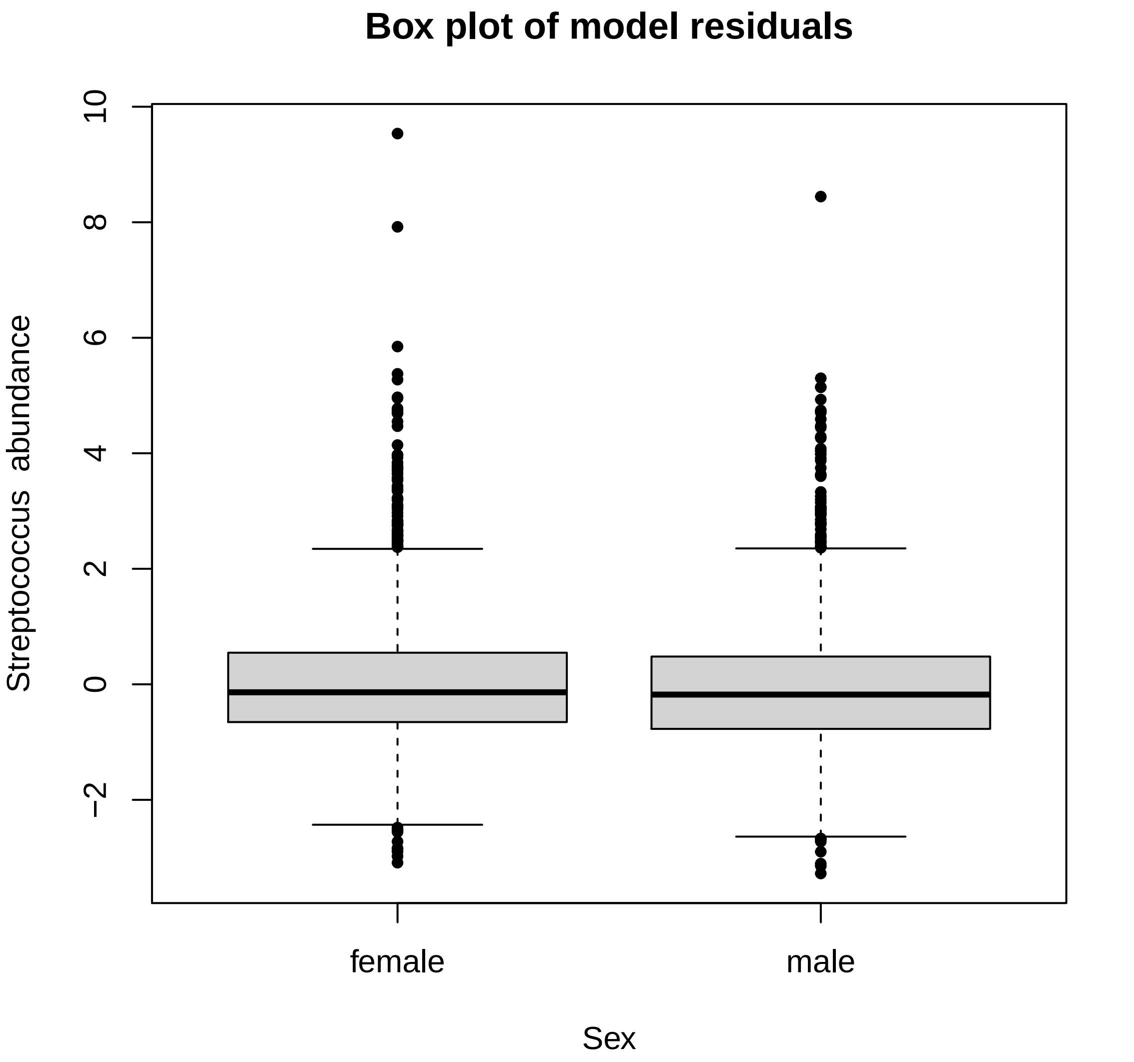
Box plot of model residuals
Streptococcus abundance
10
8
6
4
2
0
−2
female
male
Sex

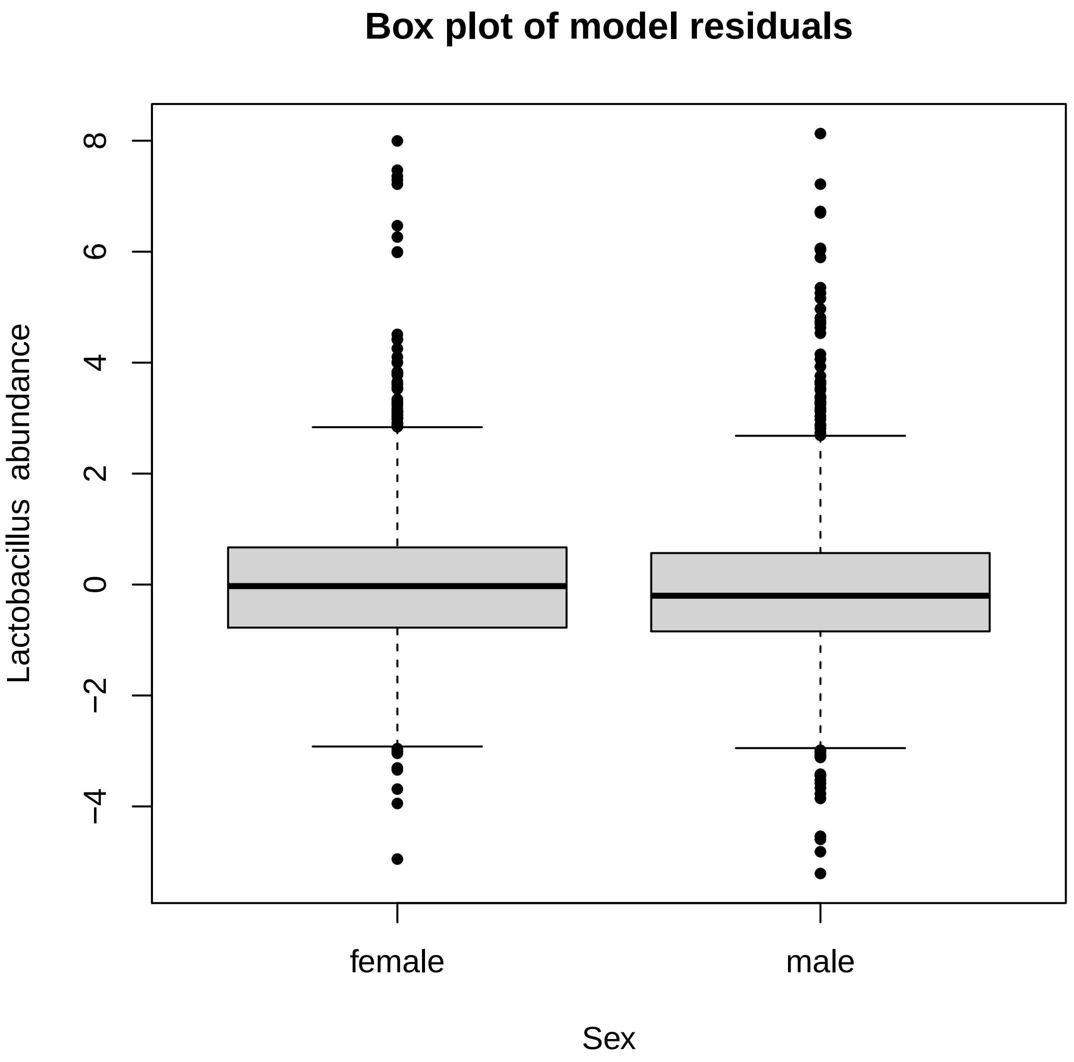
Box plot of model residuals
Lactobacillus abundance
8
6
4
2
0
−2
−4
female
male
Sex

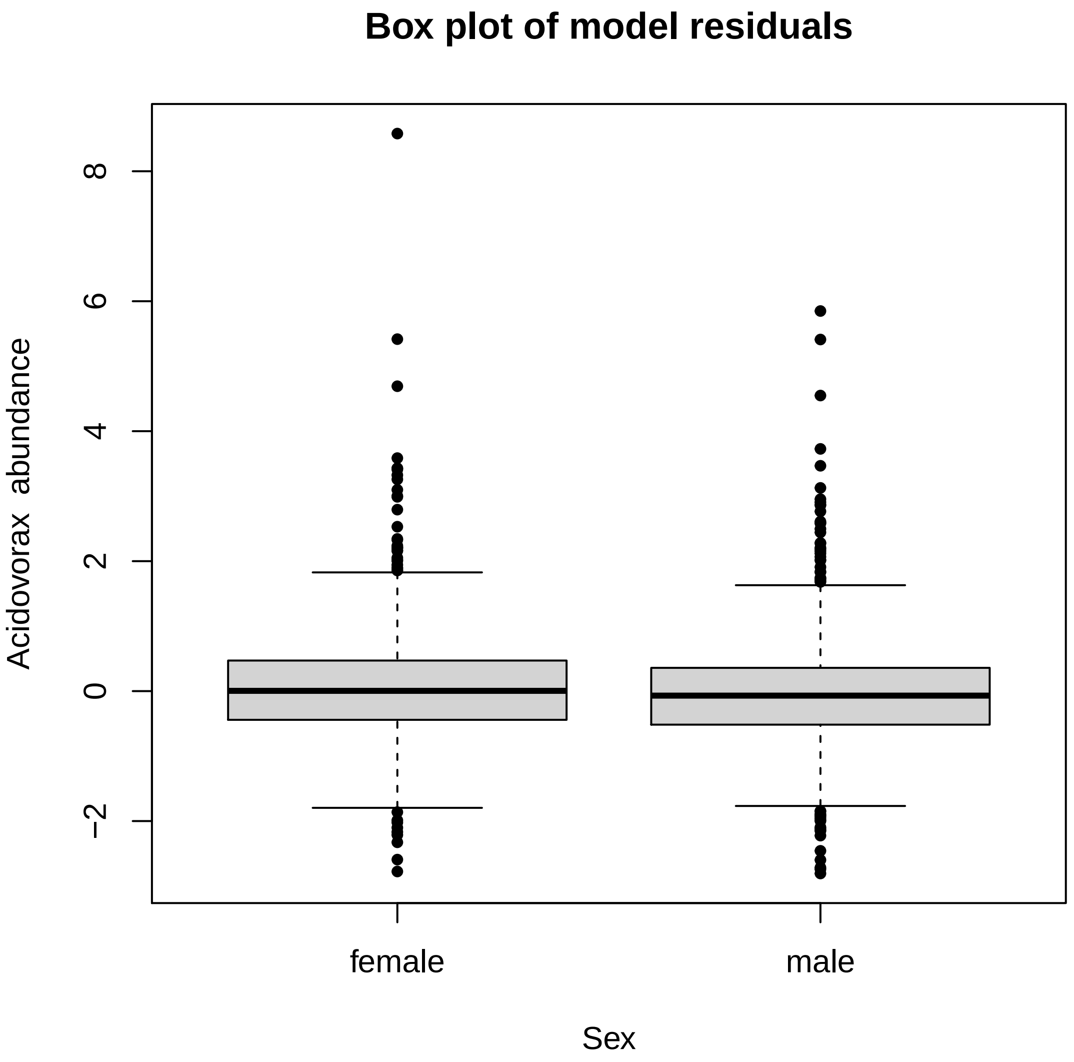
Box plot of model residuals
Acidovorax abundance
8
6
4
2
0
−2
female
male
Sex

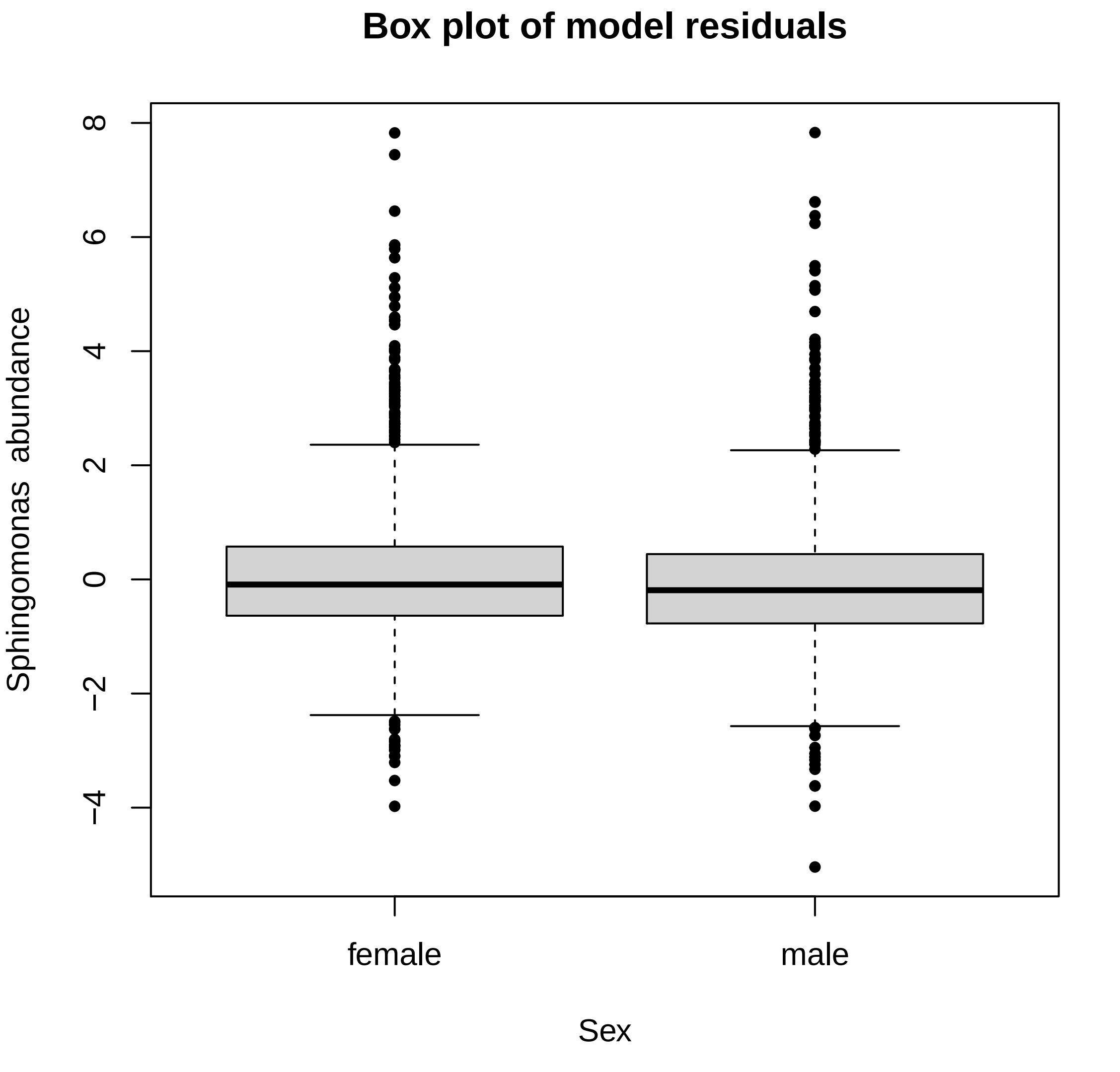
Box plot of model residuals
8
6
4
2
0
−2
−4
Sphingomonas abundance
female
male
Sex

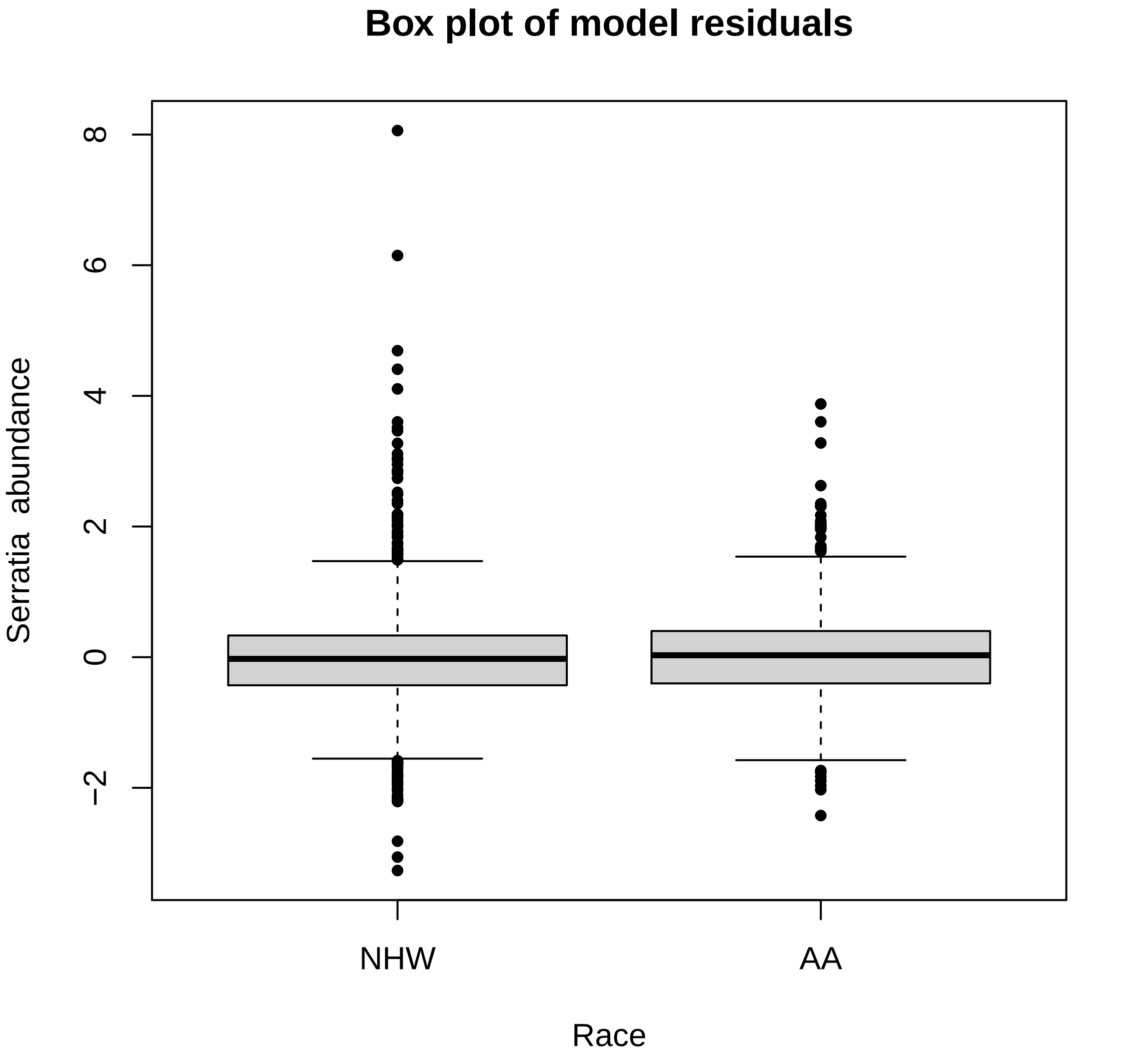
Box plot of model residuals
8
6
4
2
0
−2
Serratia abundance
NHW
AA
Race

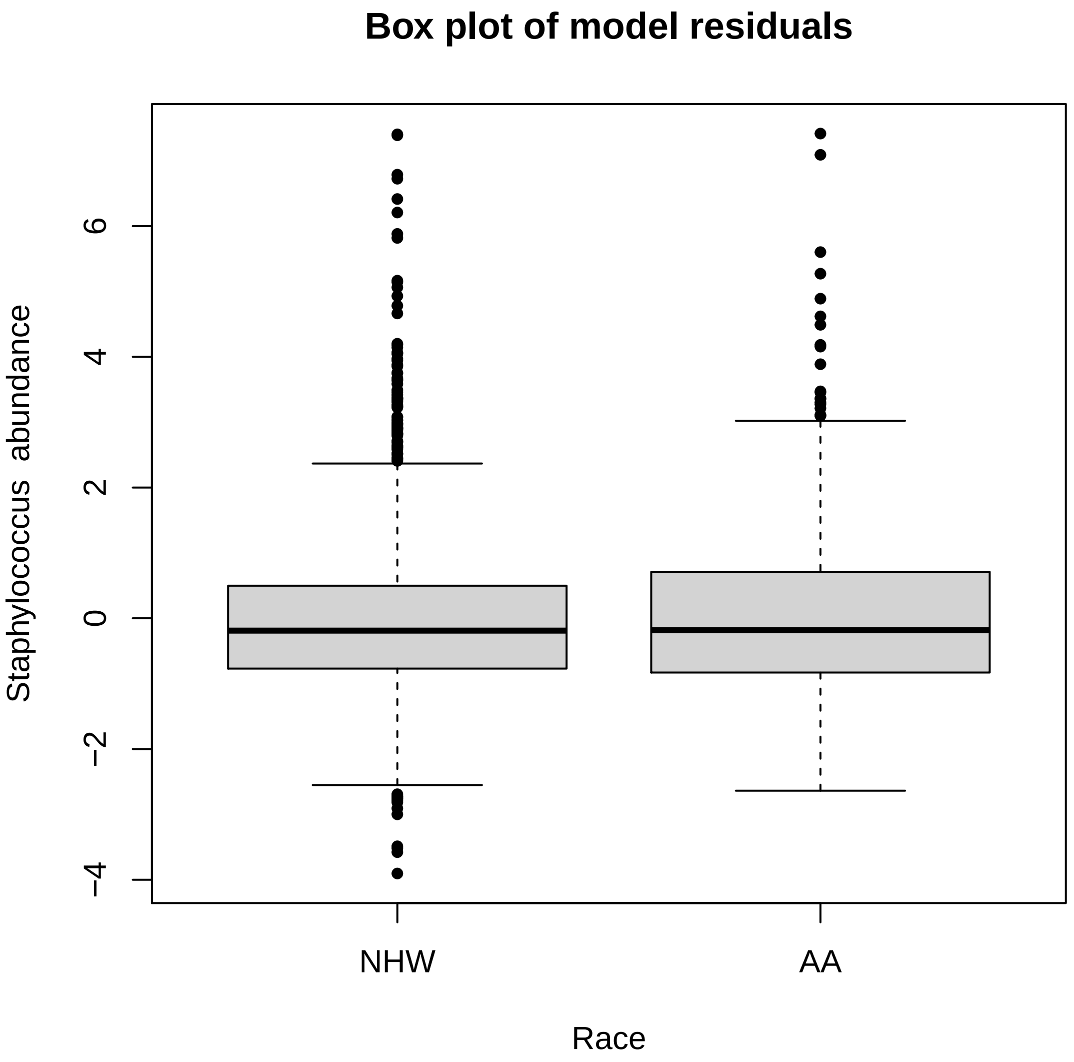
Box plot of model residuals
Staphylococcus abundance
6
4
2
0
−2
−4
NHW
AA
Race

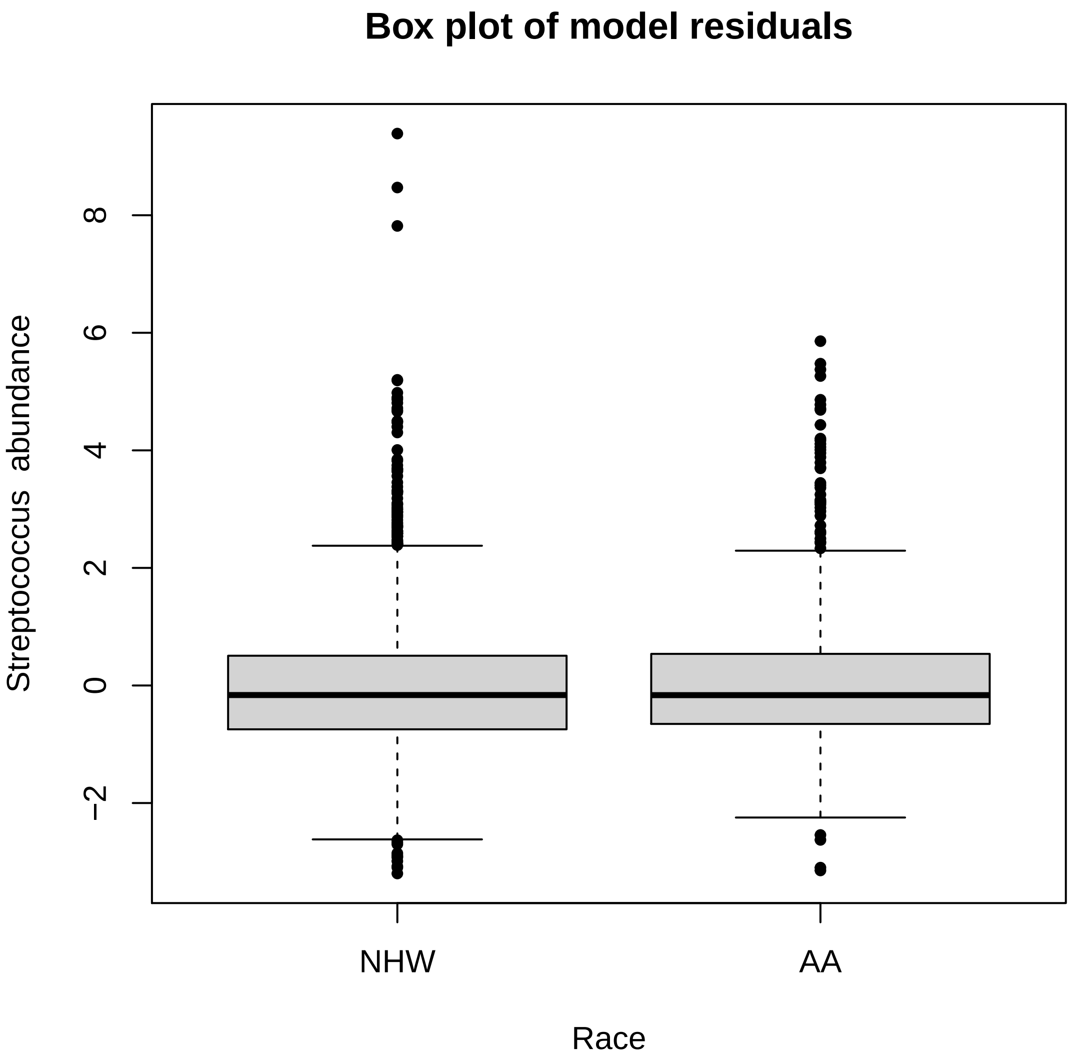

Box plot of model residuals
Streptococcus abundance
8
6
4
2
0
−2
NHW
AA
Race

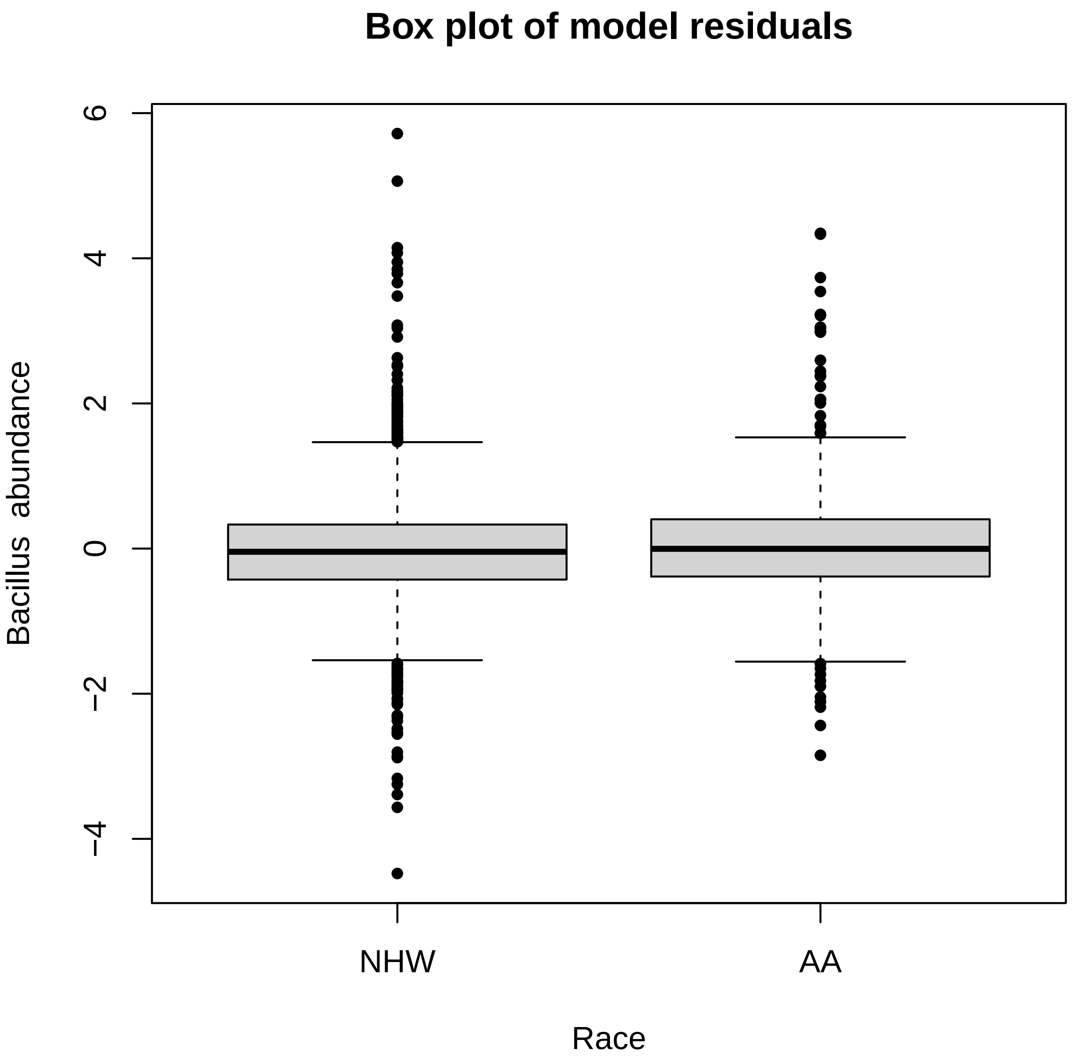
Box plot of model residuals
Bacillus abundance
6
4
2
0
−2
−4
NHW
AA
Race

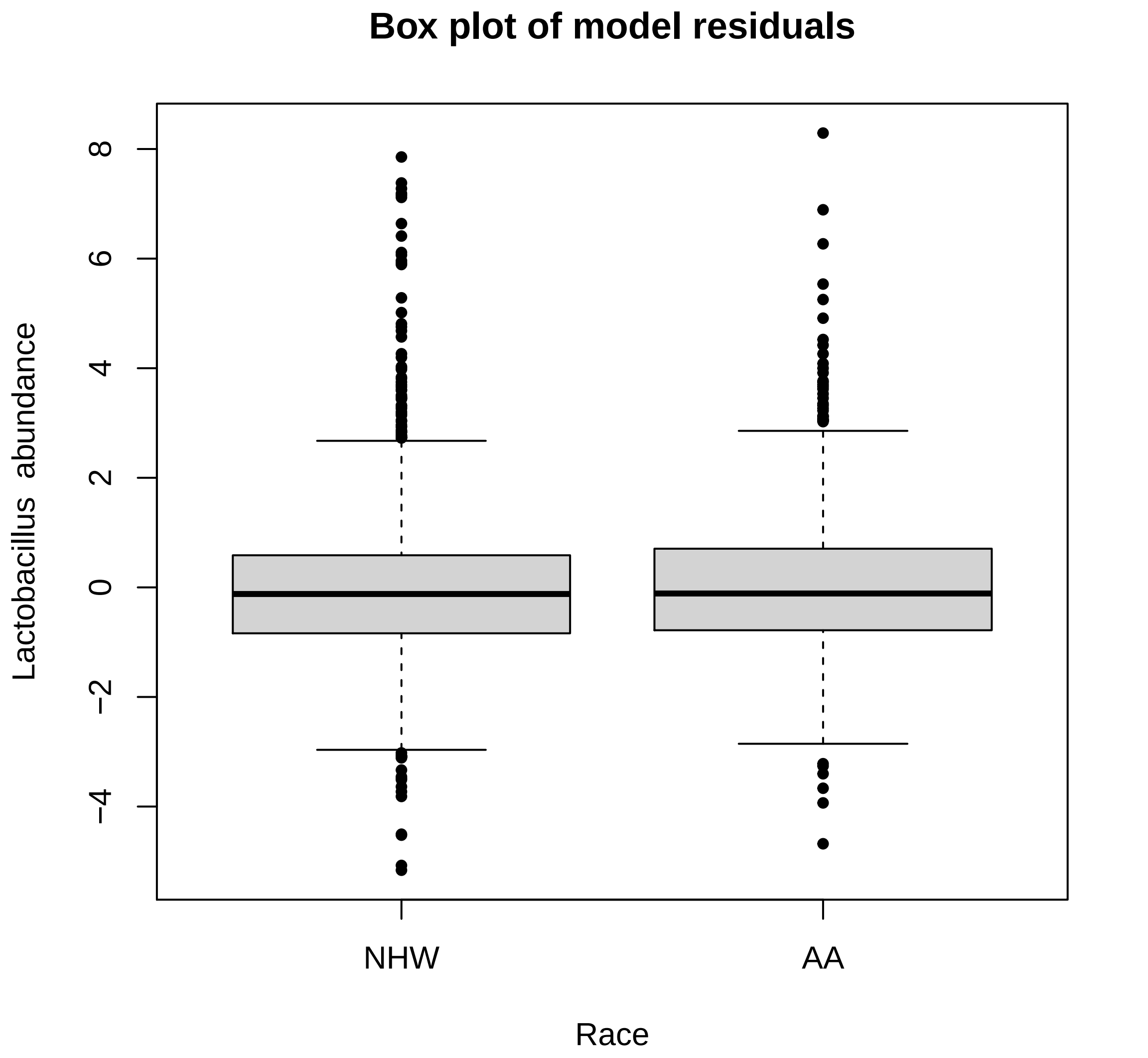
Box plot of model residuals
Lactobacillus abundance
8
6
4
2
0
−2
−4
NHW
AA
Race

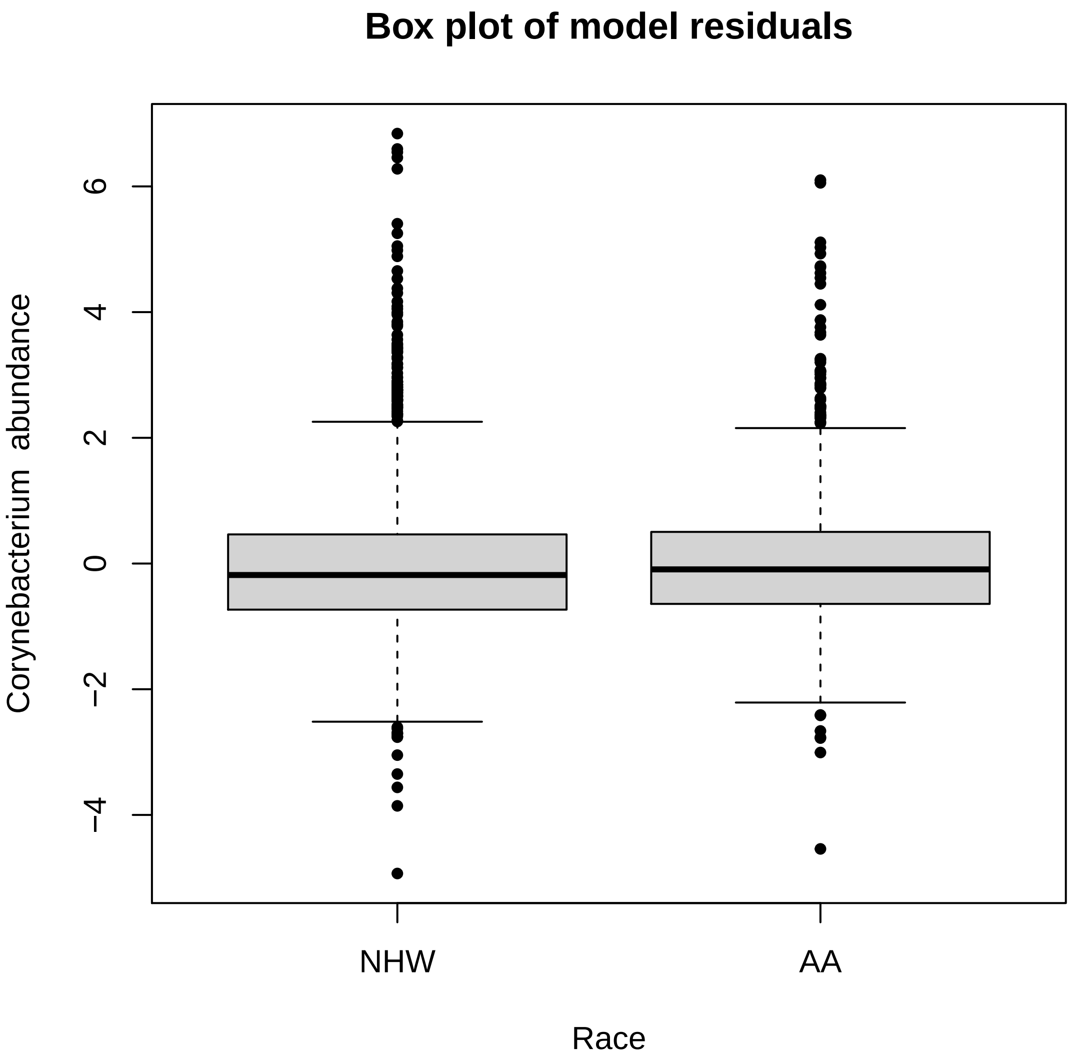
Box plot of model residuals
Corynebacterium abundance
6
4
2
0
−2
−4
NHW
AA
Race

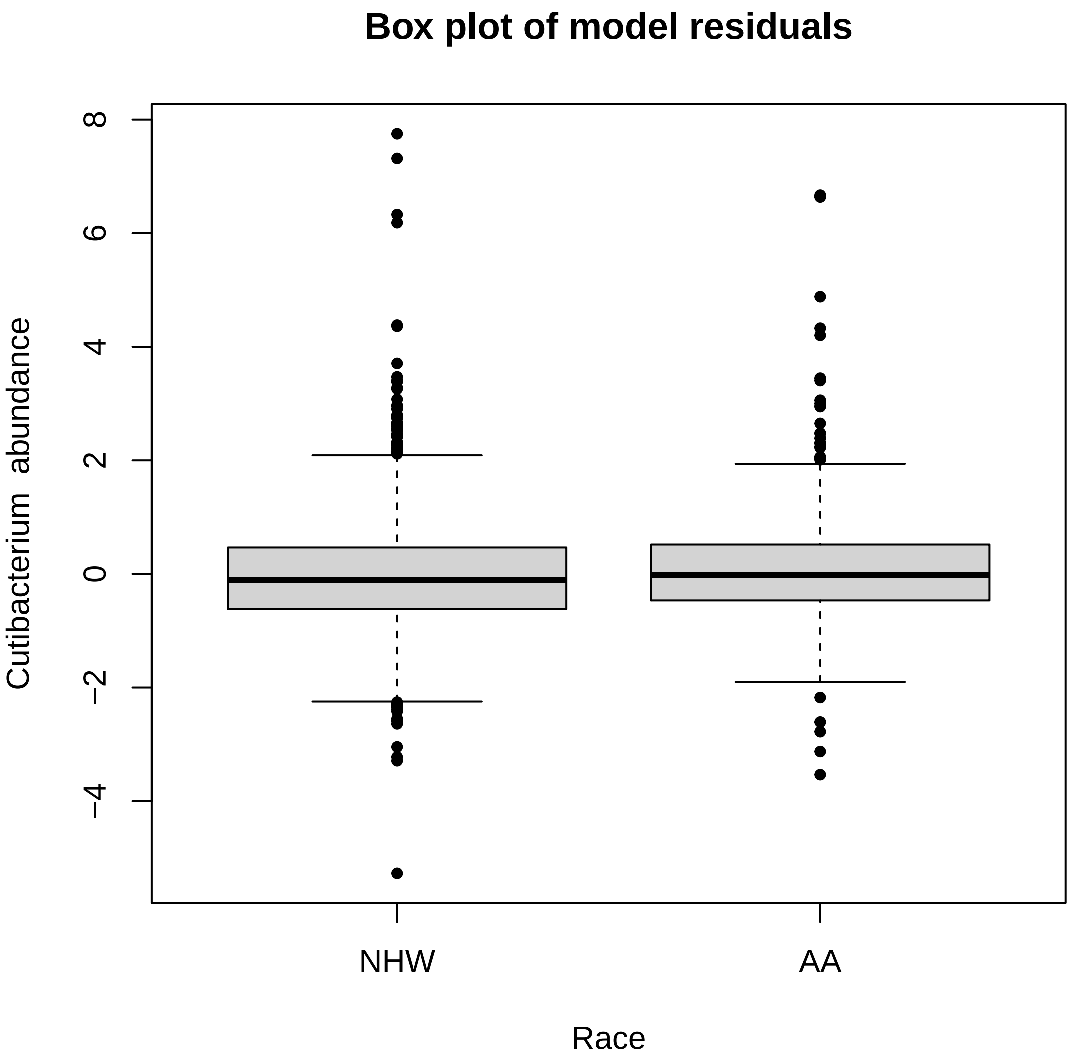
Box plot of model residuals
Cutibacterium abundance
8
6
4
2
0
−2
−4
NHW
AA
Race
