# Supplementary material for: Peripheral blood microbial signatures in current and former smokers
Source: Sci Rep. 2021 Oct 6;11:19875. doi: 10.1038/s41598-021-99238-4 (PMC8494912; doi:10.1038/s41598-021-99238-4)
Supplement: Supplementary file 3 — Supplementary Information 3. [file 41598_2021_99238_MOESM3_ESM.pdf]

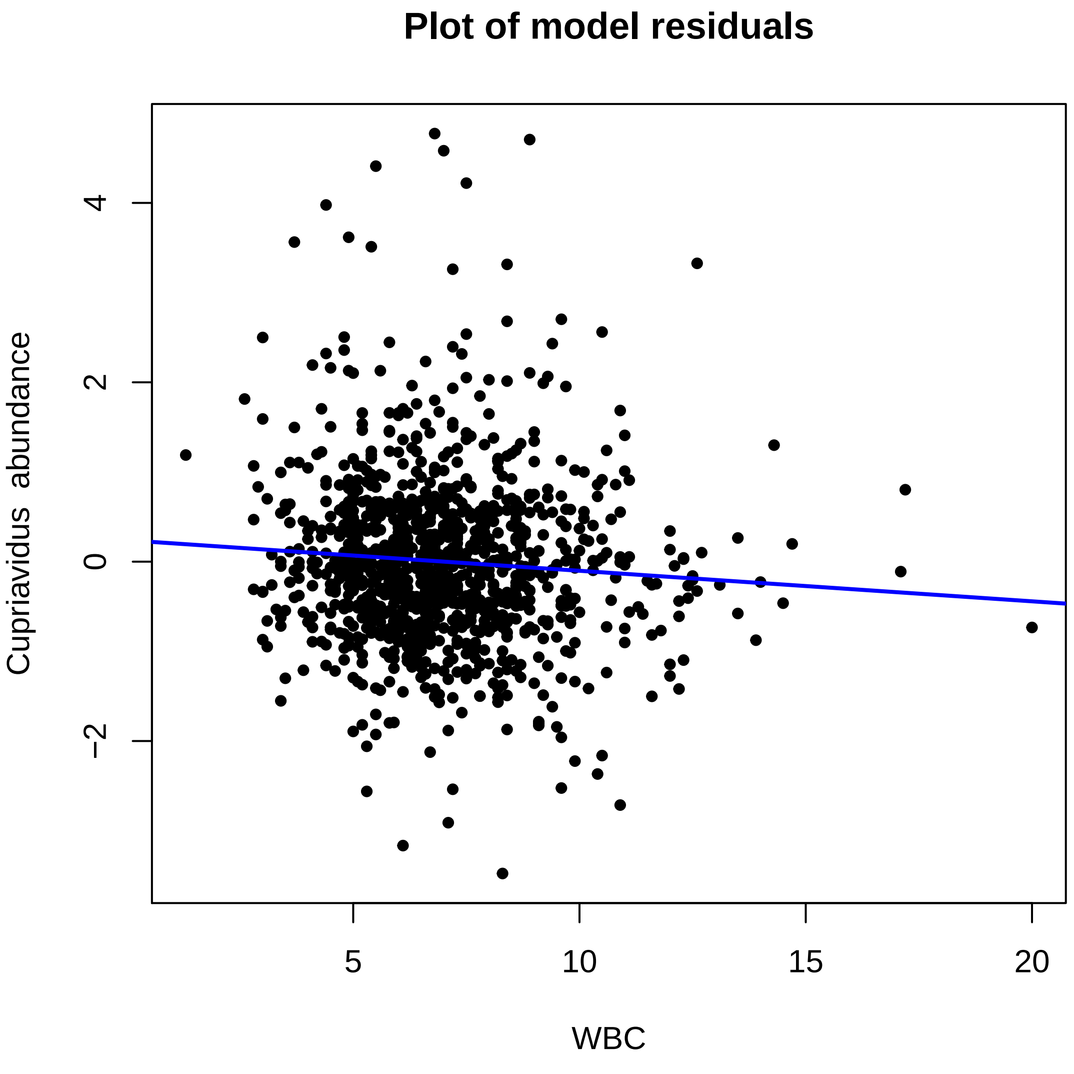

Plot of model residuals
Cupriavidus abundance
WBC
4
2
0
−2
5
10
15
20

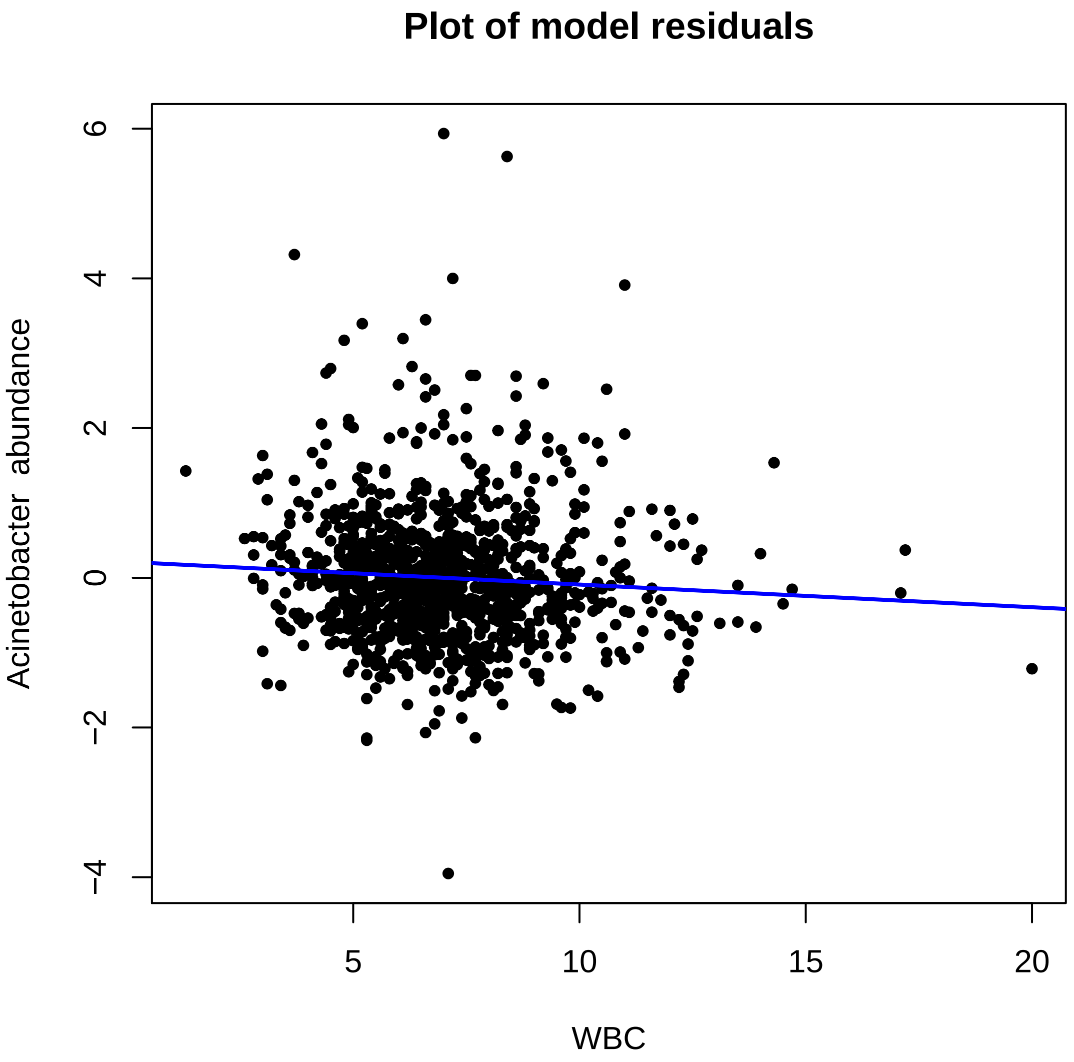

Plot of model residuals
Acinetobacter abundance
6
4
2
0
−2
−4
5
10
15
20
WBC

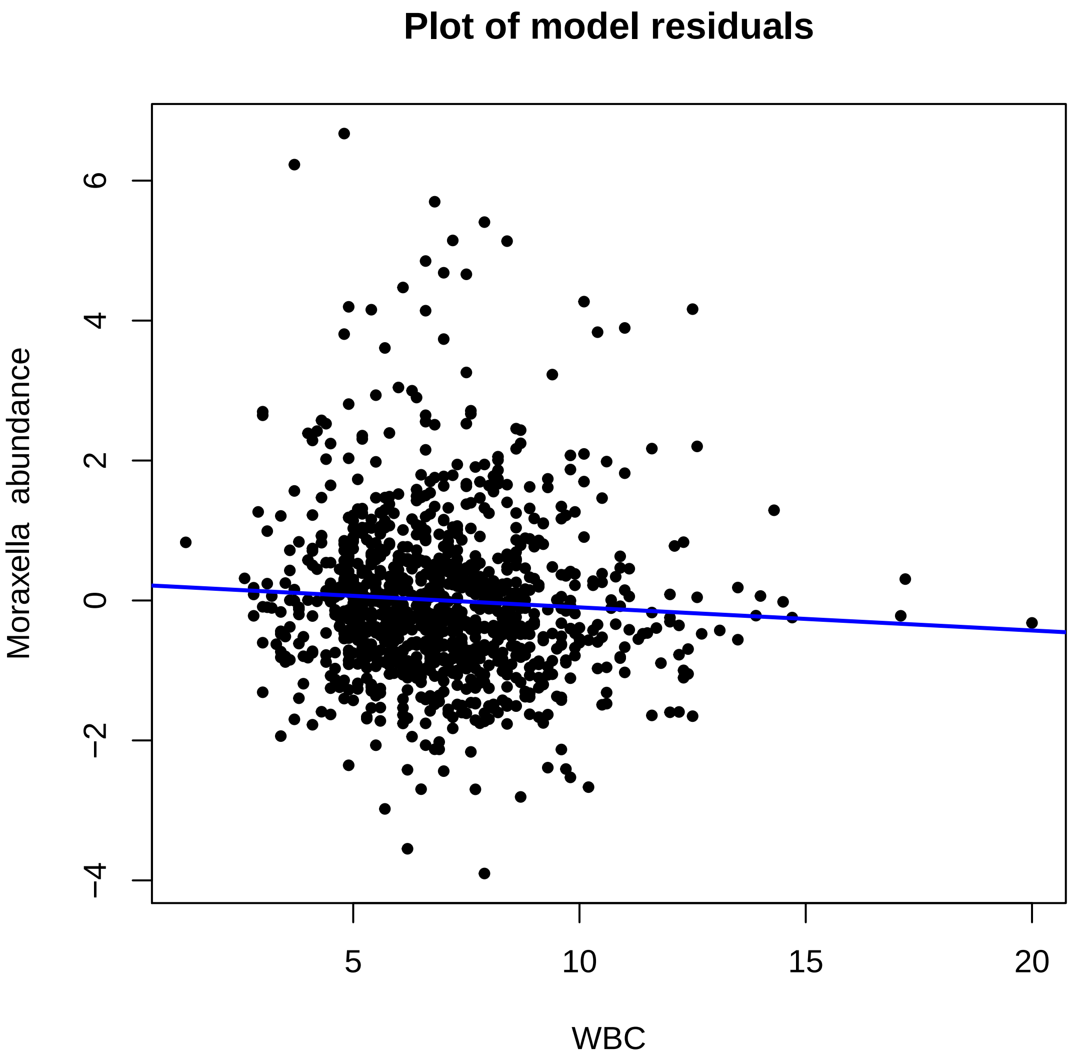
Plot of model residuals
Moraxella abundance
WBC
6
4
2
0
−2
−4
5
10
15
20

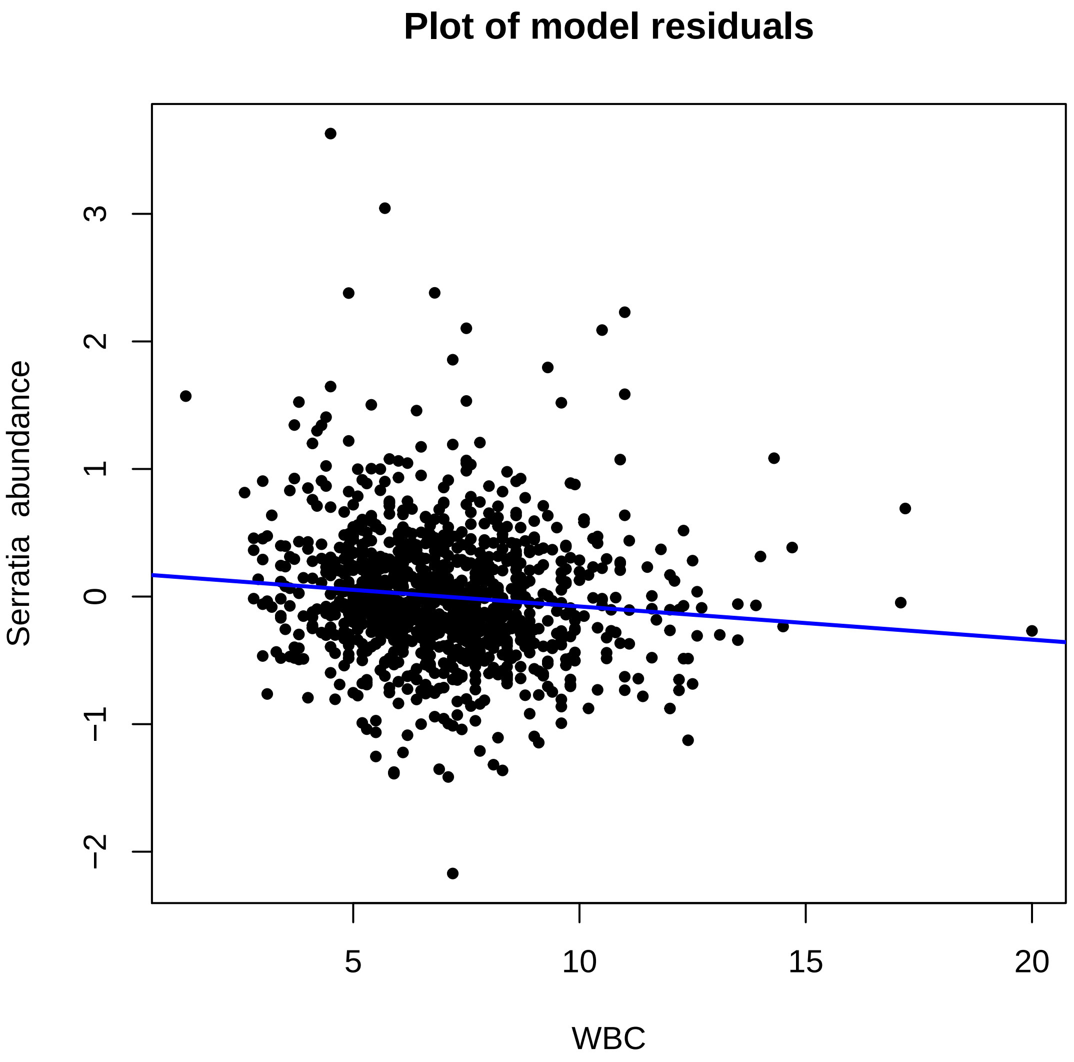
Plot of model residuals
Serratia abundance
WBC
3
2
1
0
−1
−2
5
10
15
20

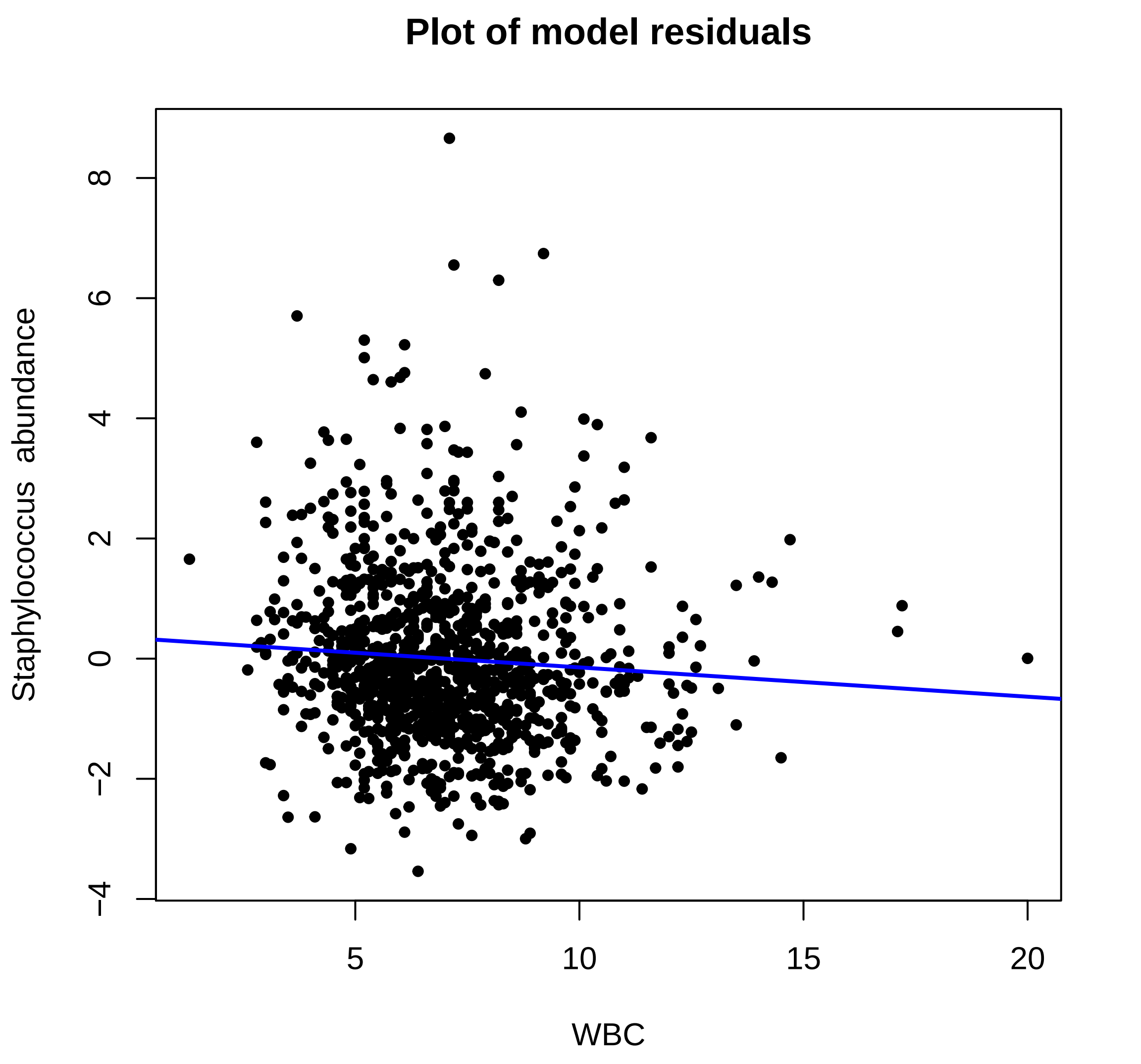

Plot of model residuals
Staphylococcus abundance
WBC
−4
−2
0
2
4
6
8
5
10
15
20

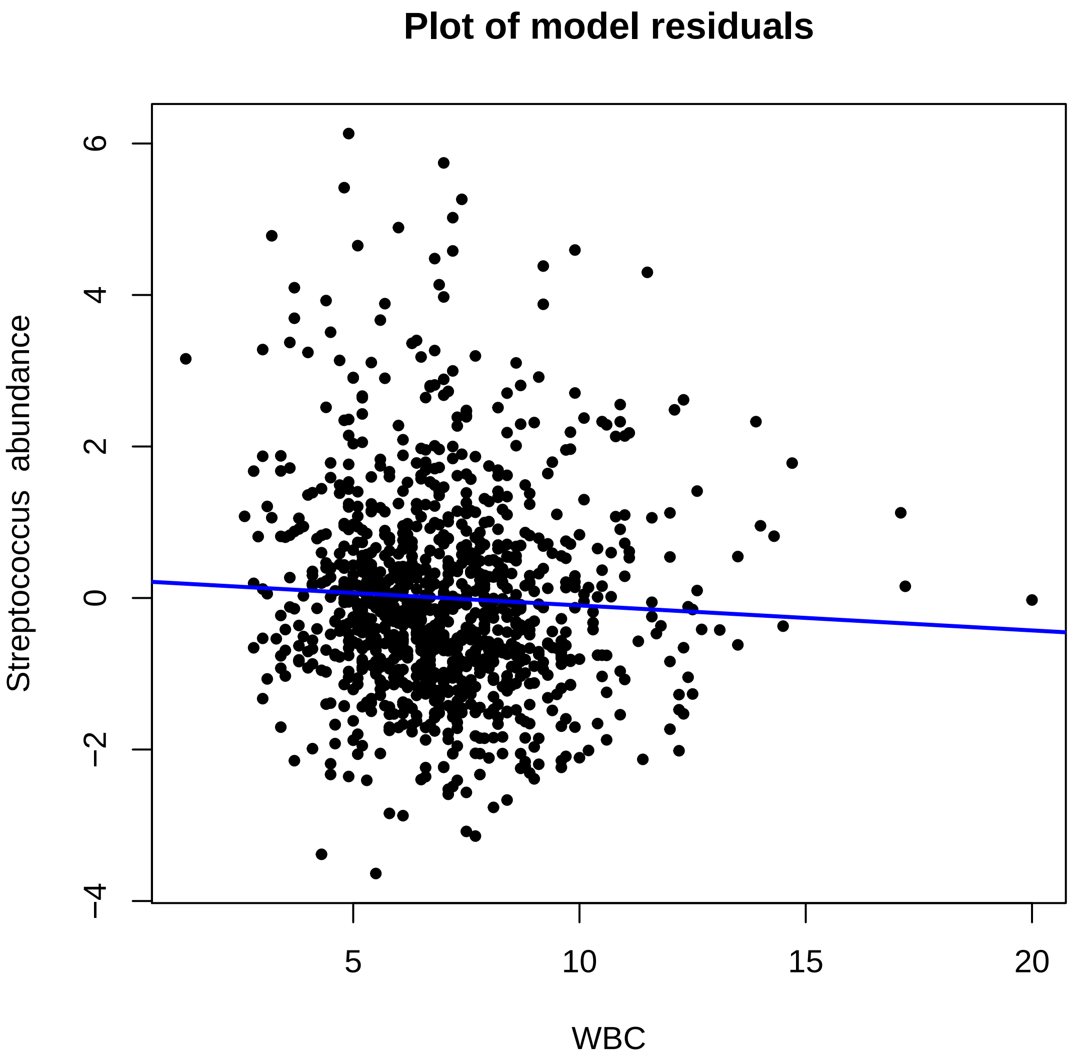
Plot of model residuals
Streptococcus abundance
WBC
6
4
2
0
−2
−4
5
10
15
20

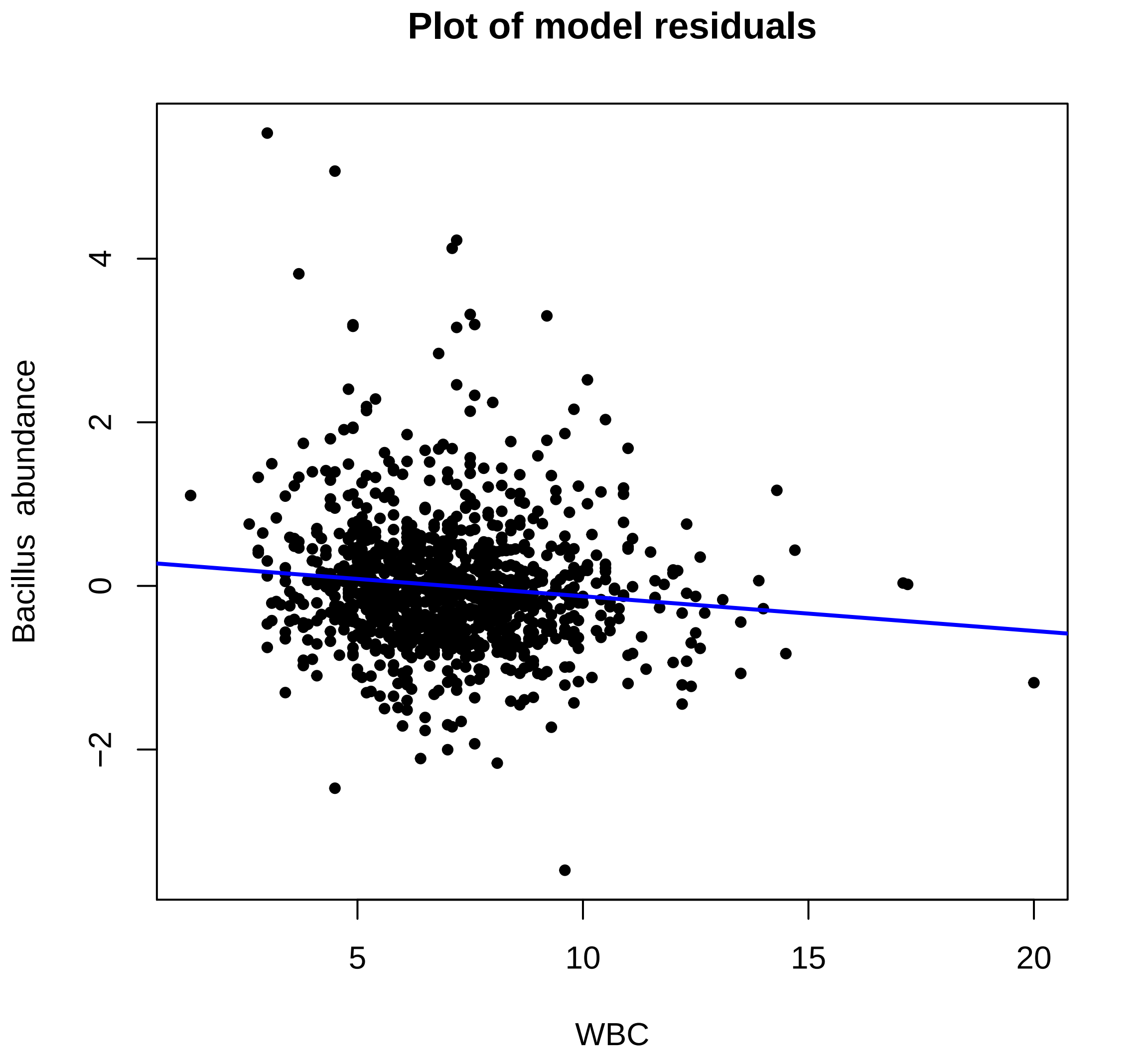

Plot of model residuals
Bacillus abundance
4
2
0
−2
5
10
15
20
WBC

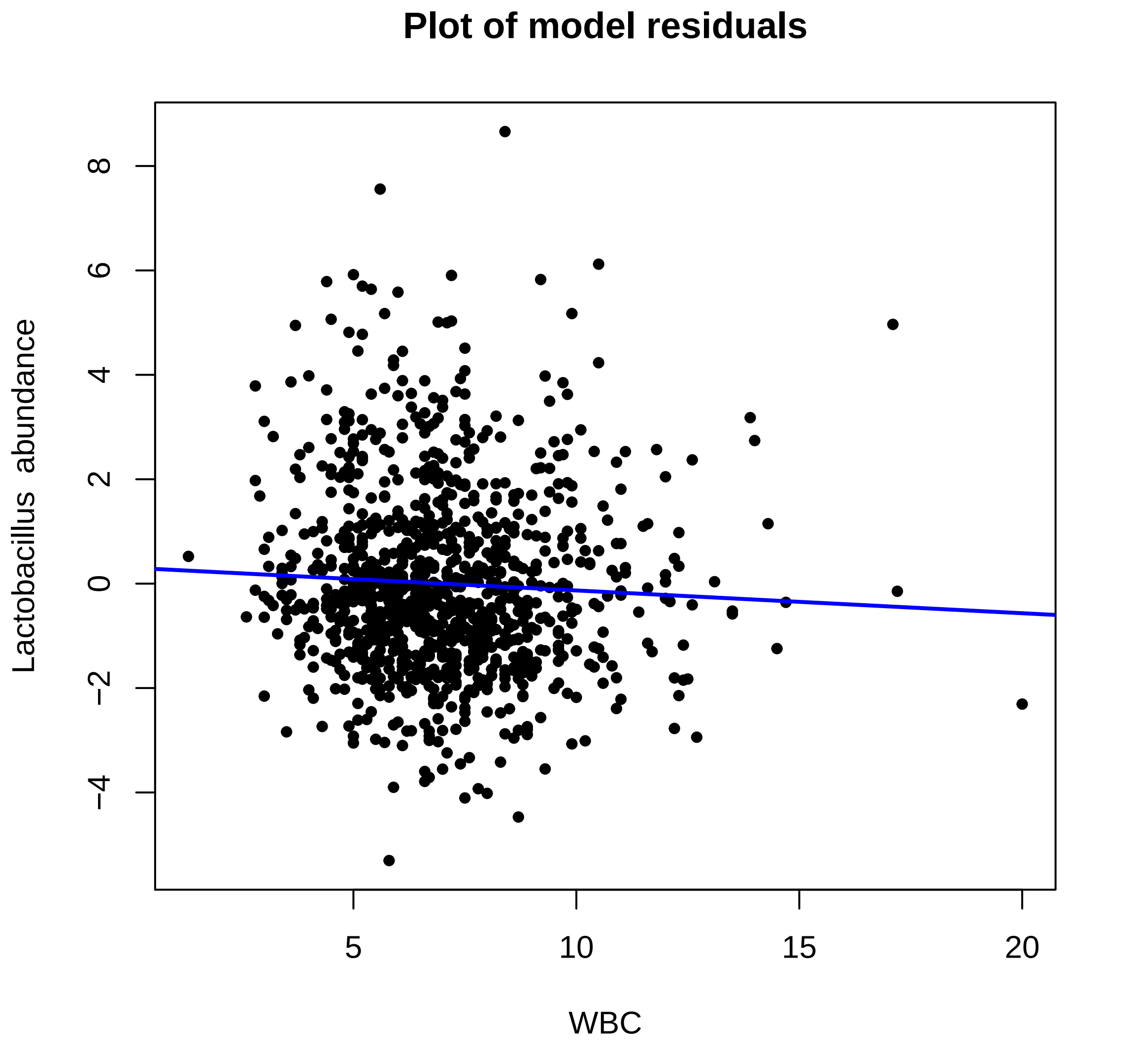
Plot of model residuals
Lactobacillus abundance
WBC
8
6
4
2
0
−2
−4
5
10
15
20

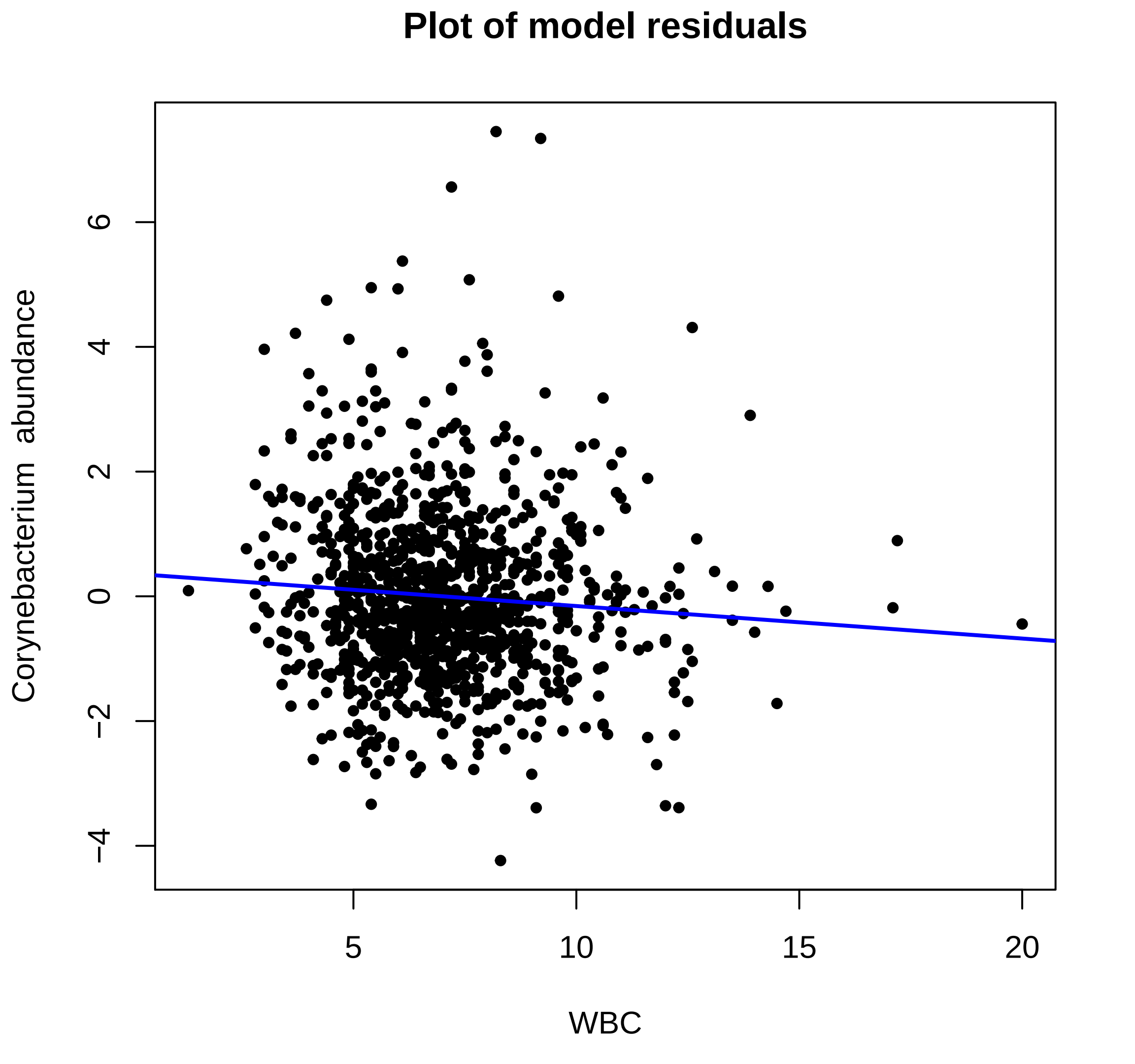

Plot of model residuals
Corynebacterium abundance
WBC
6
4
2
0
−2
−4
5
10
15
20

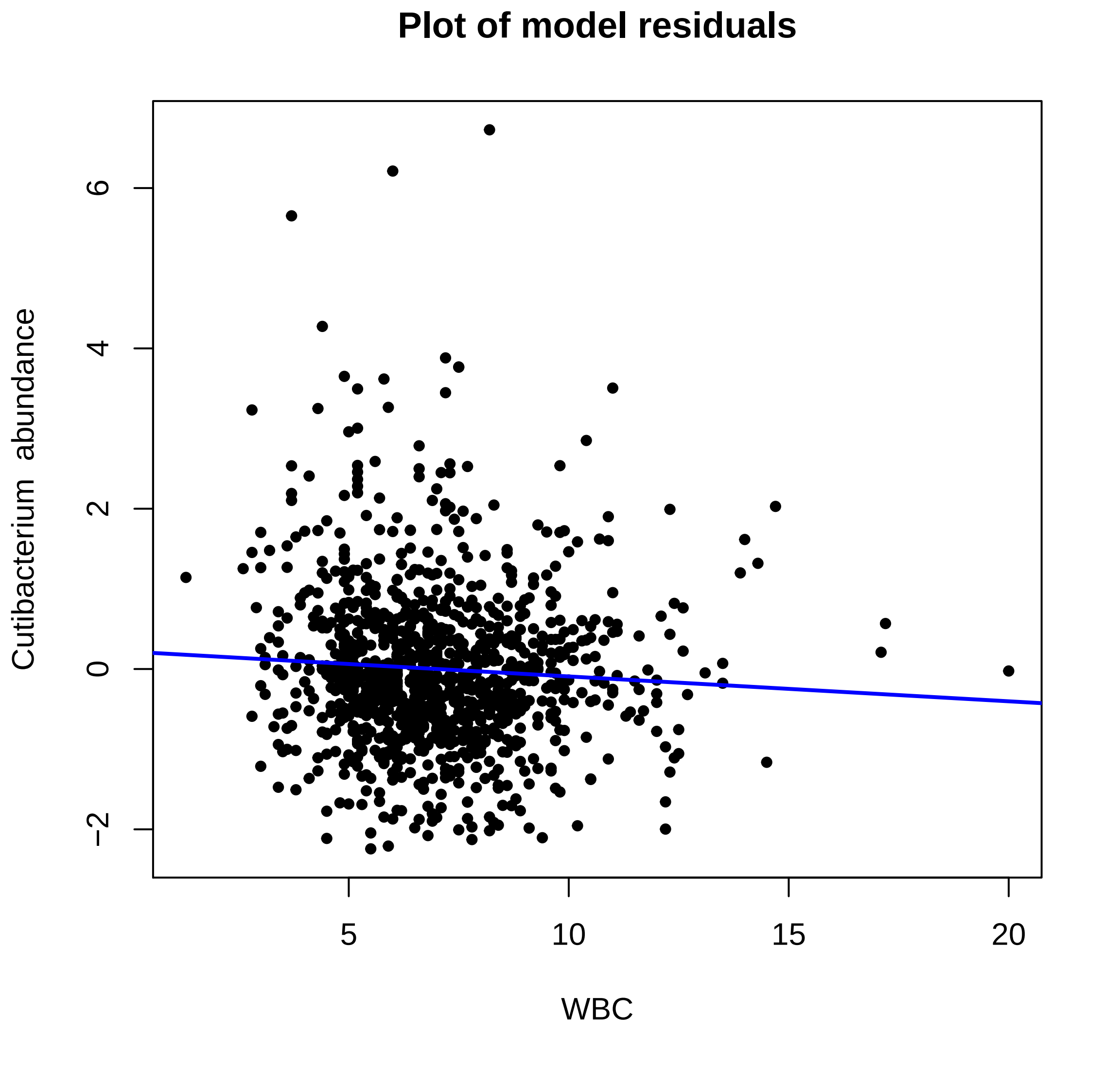
Plot of model residuals
Cutibacterium abundance
WBC
−2
0
2
4
6
5
10
15
20

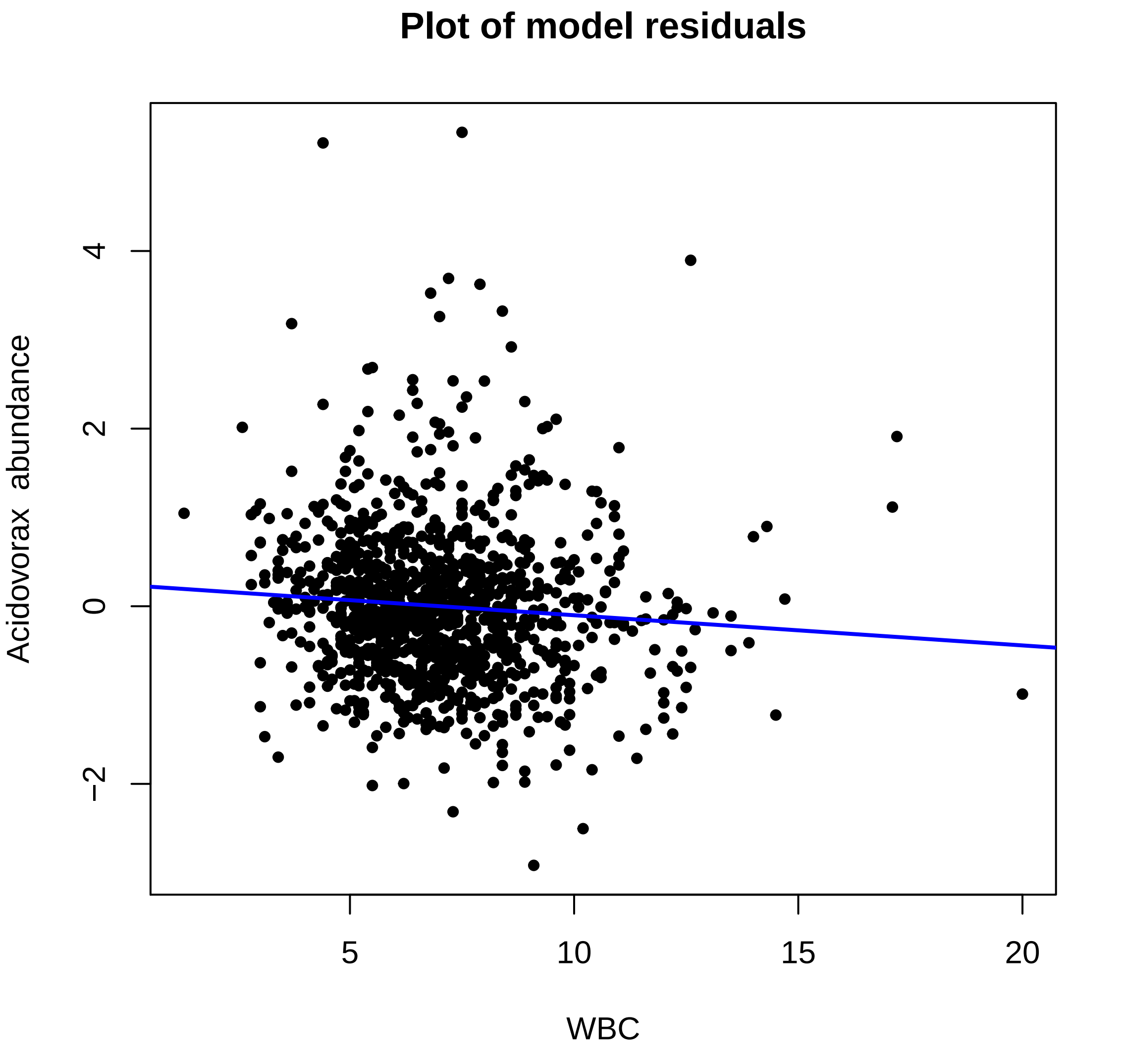
Plot of model residuals
Acidovorax abundance
4
2
0
−2
5
10
15
20
WBC

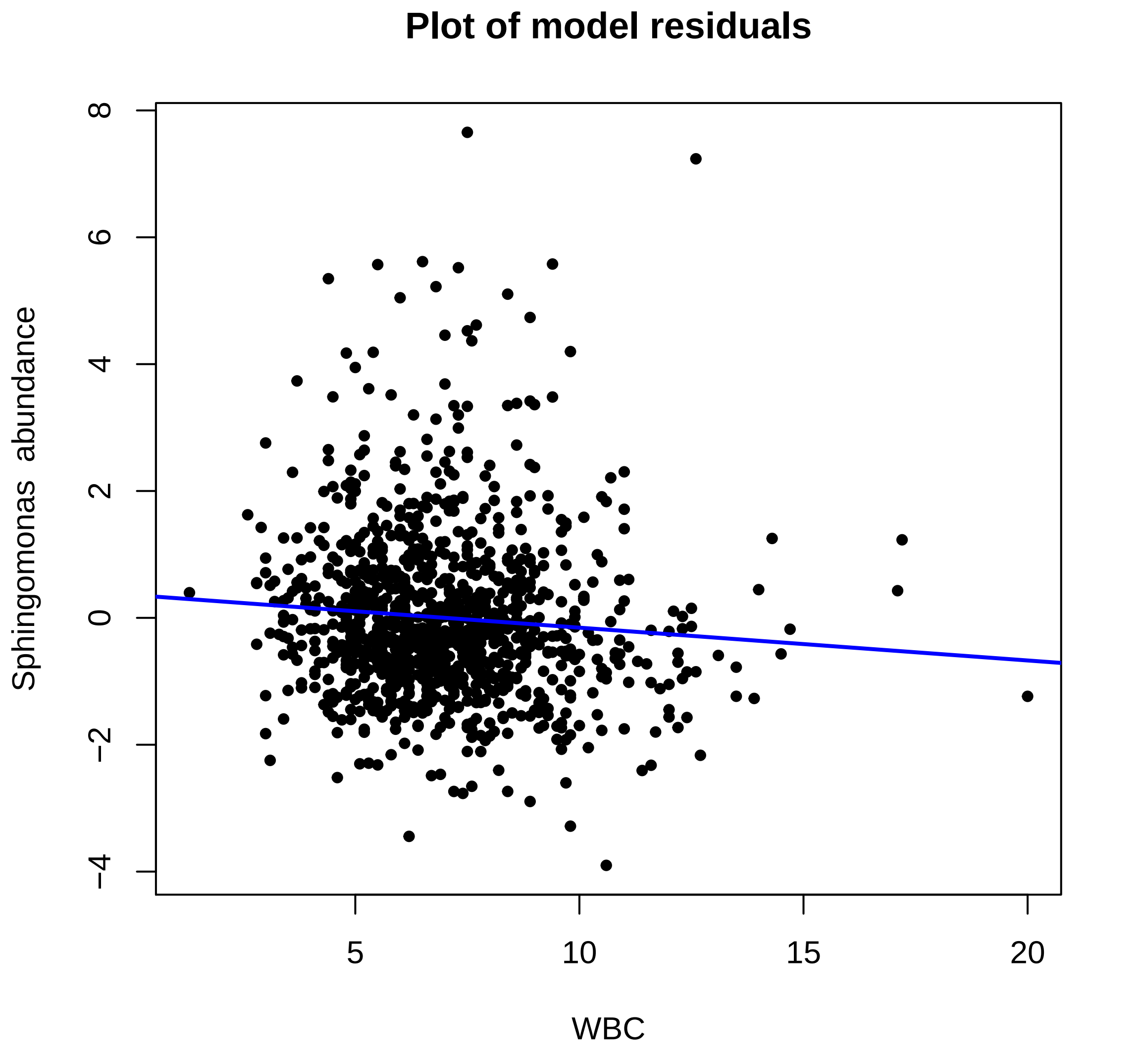

Plot of model residuals
Sphingomonas abundance
WBC
8
6
4
2
0
−2
−4
5
10
15
20

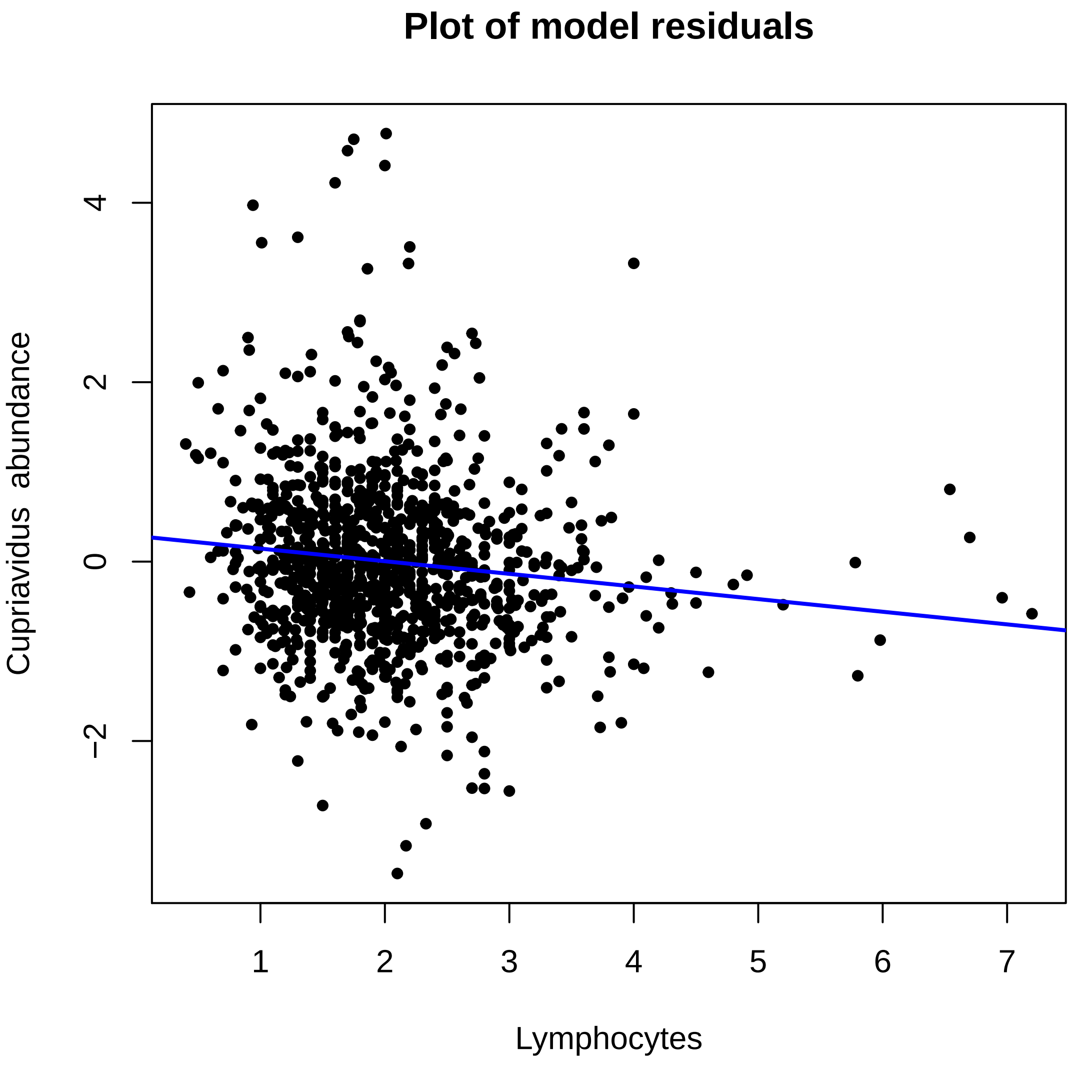

Plot of model residuals
Cupriavidus abundance
Lymphocytes
4
2
0
−2
1
2
3
4
5
6
7

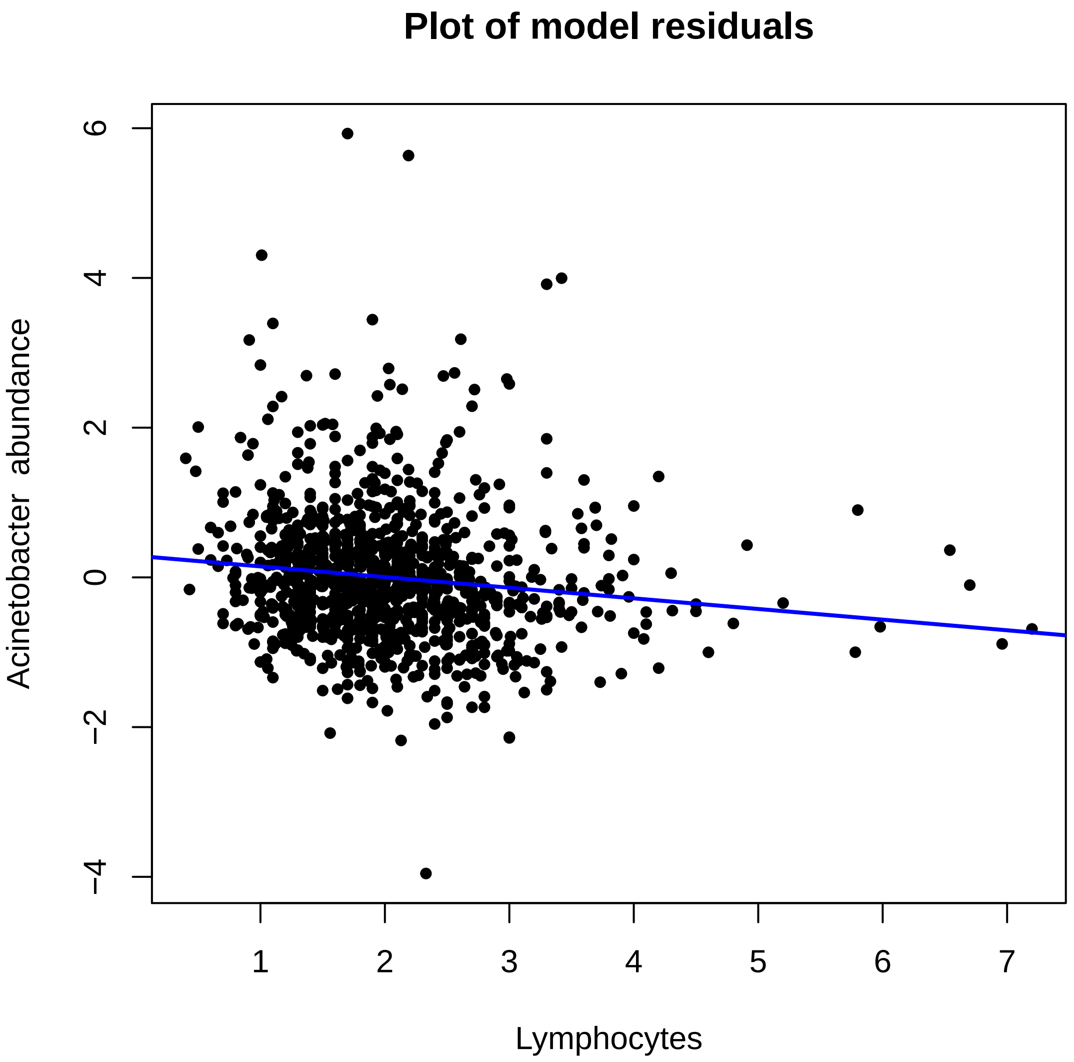
Plot of model residuals
Acinetobacter abundance
Lymphocytes
6
4
2
0
−2
−4
1
2
3
4
5
6
7

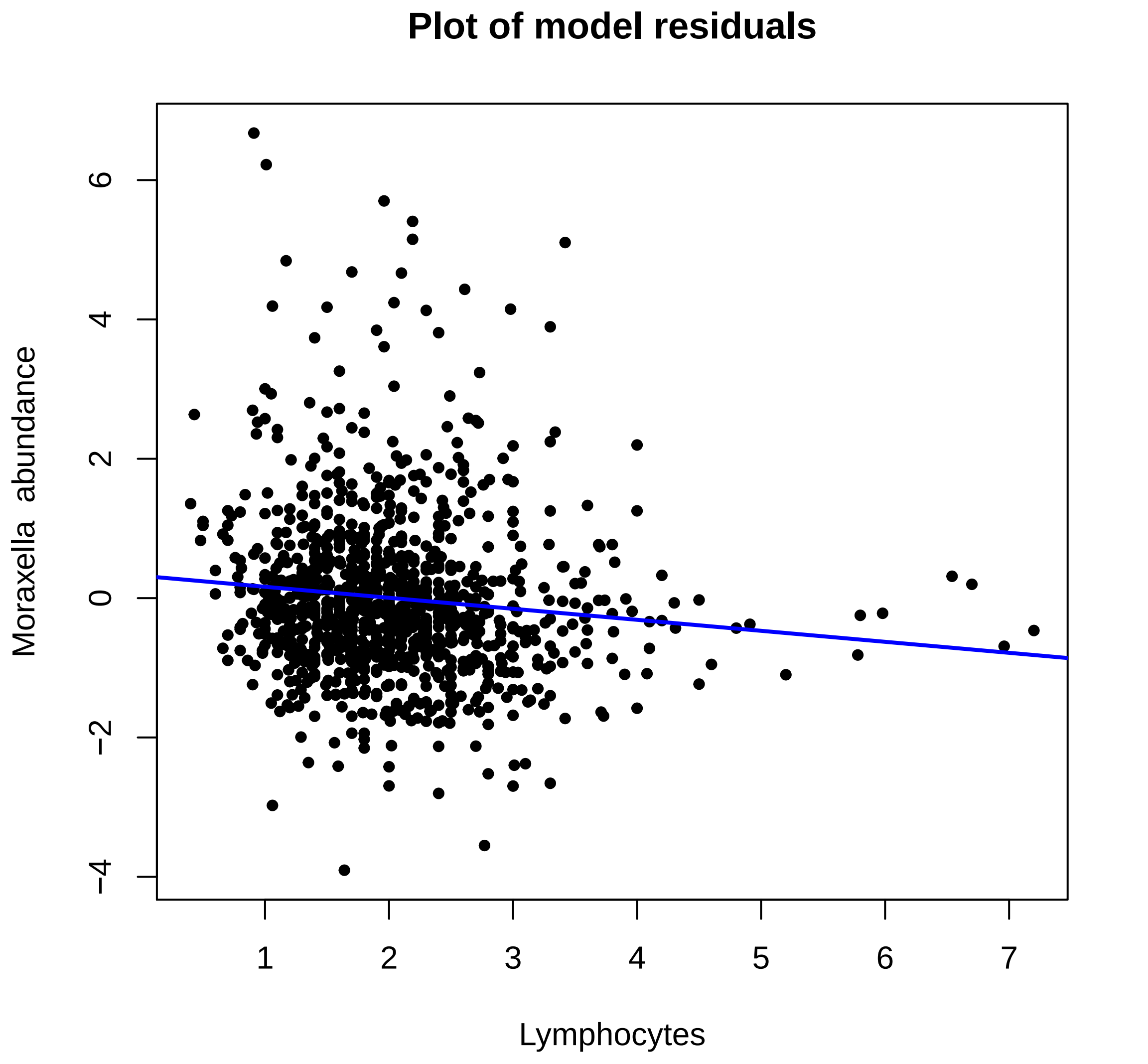

Plot of model residuals
Moraxella abundance
Lymphocytes
6
4
2
0
−2
−4
1
2
3
4
5
6
7

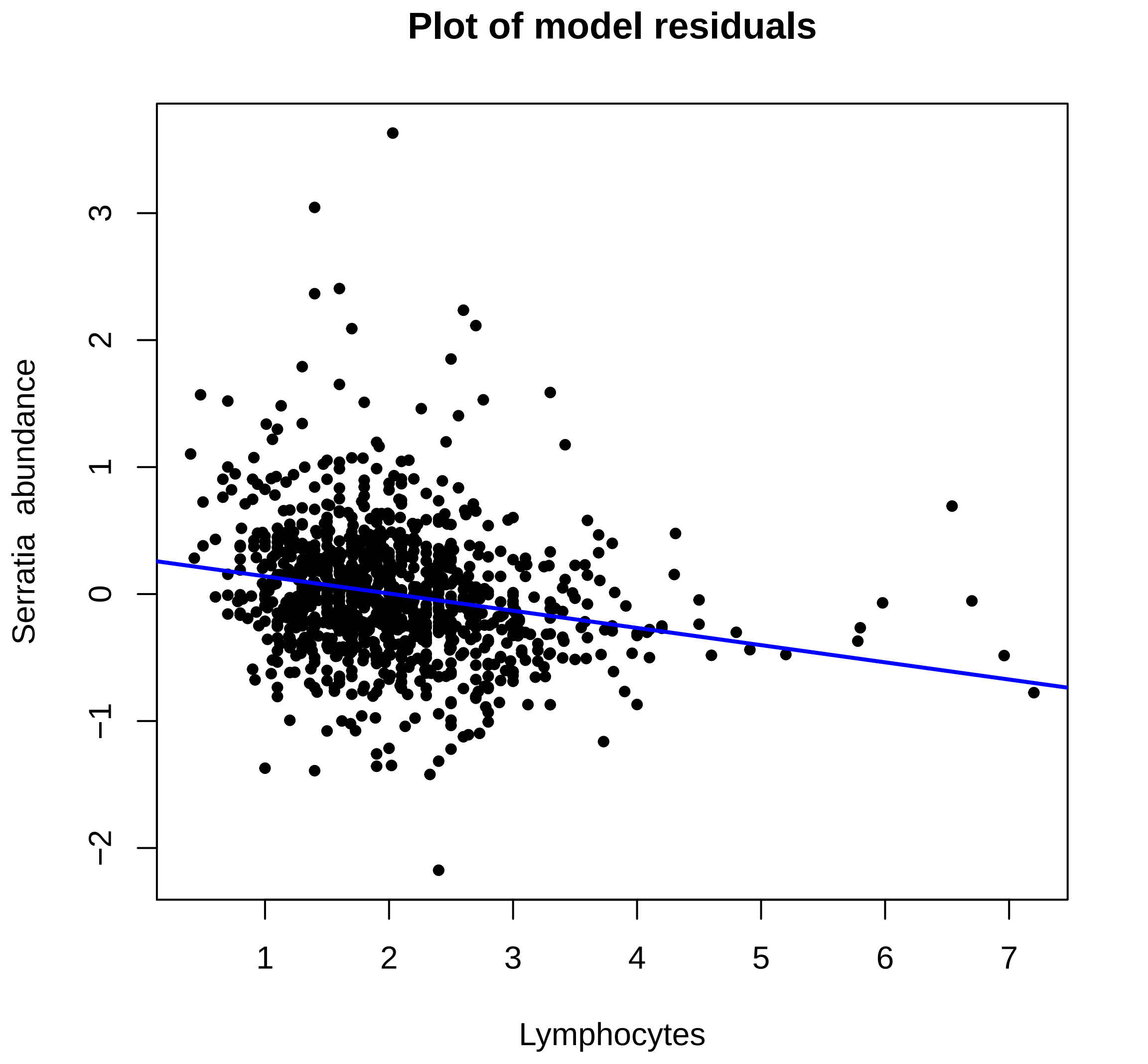
Plot of model residuals
Serratia abundance
Lymphocytes
3
2
1
0
−1
−2
1
2
3
4
5
6
7

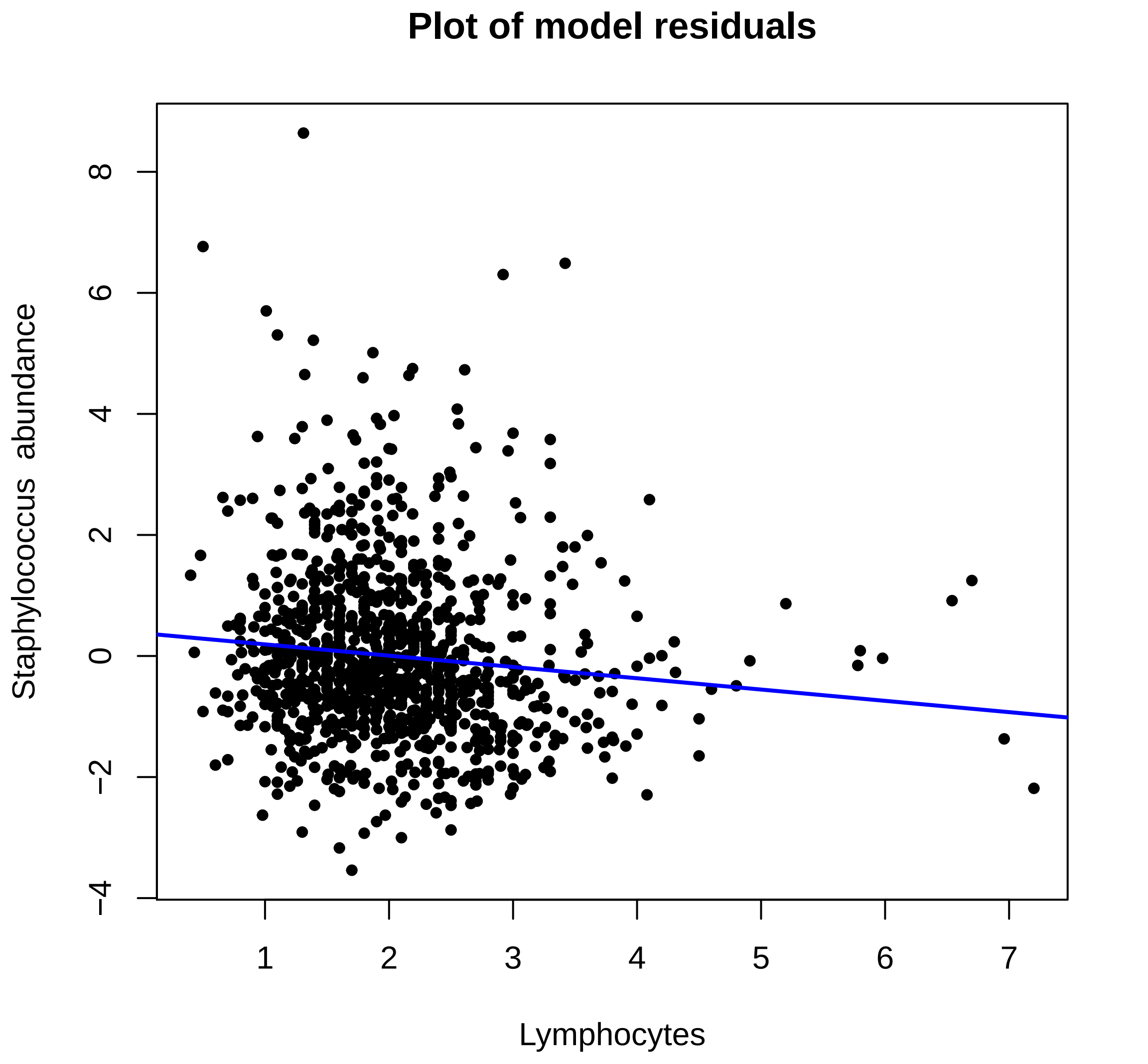

Plot of model residuals
Staphylococcus abundance
Lymphocytes
8
6
4
2
0
−2
−4
1
2
3
4
5
6
7

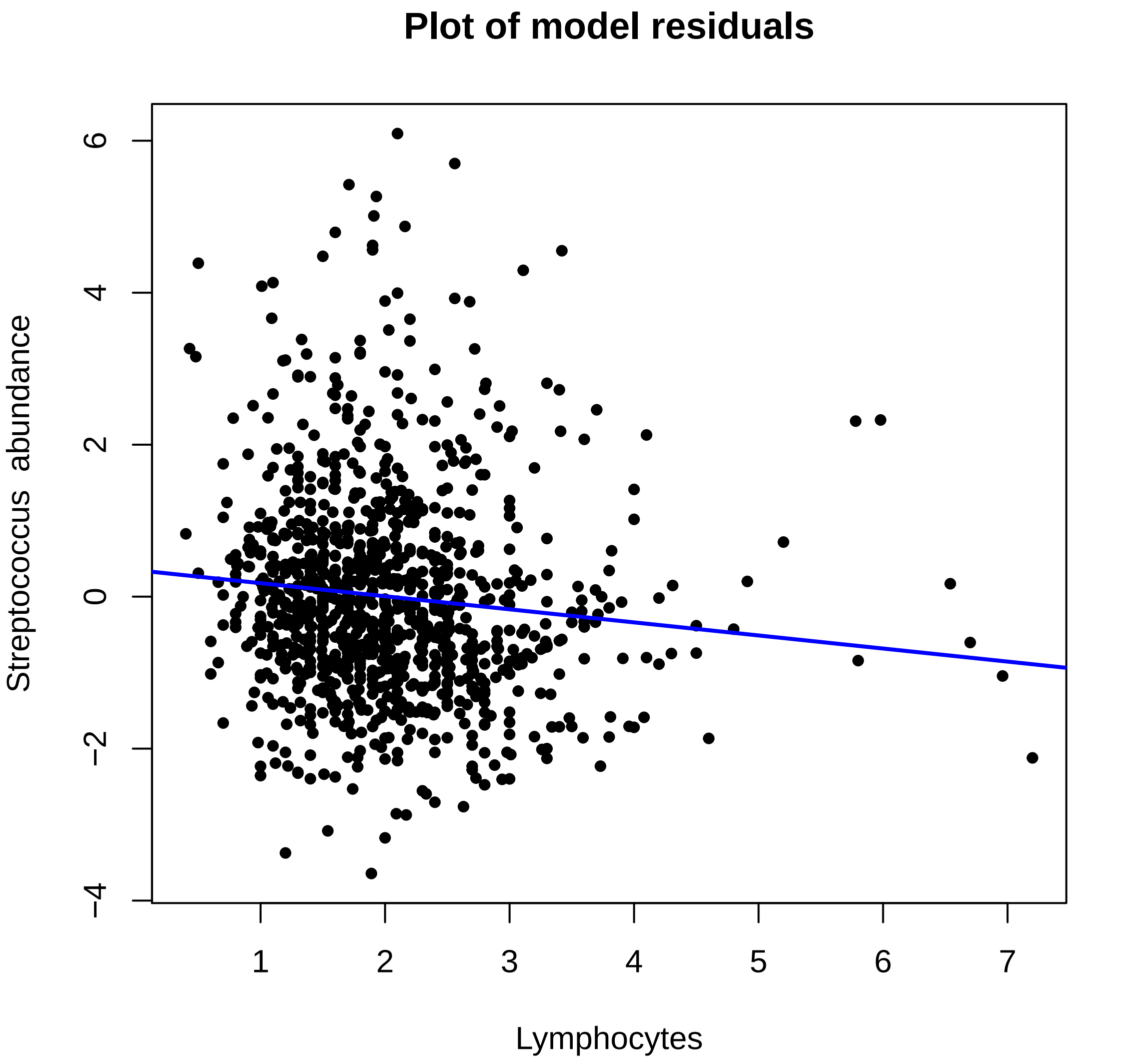

Plot of model residuals
Streptococcus abundance
Lymphocytes
6
4
2
0
−2
−4
1
2
3
4
5
6
7

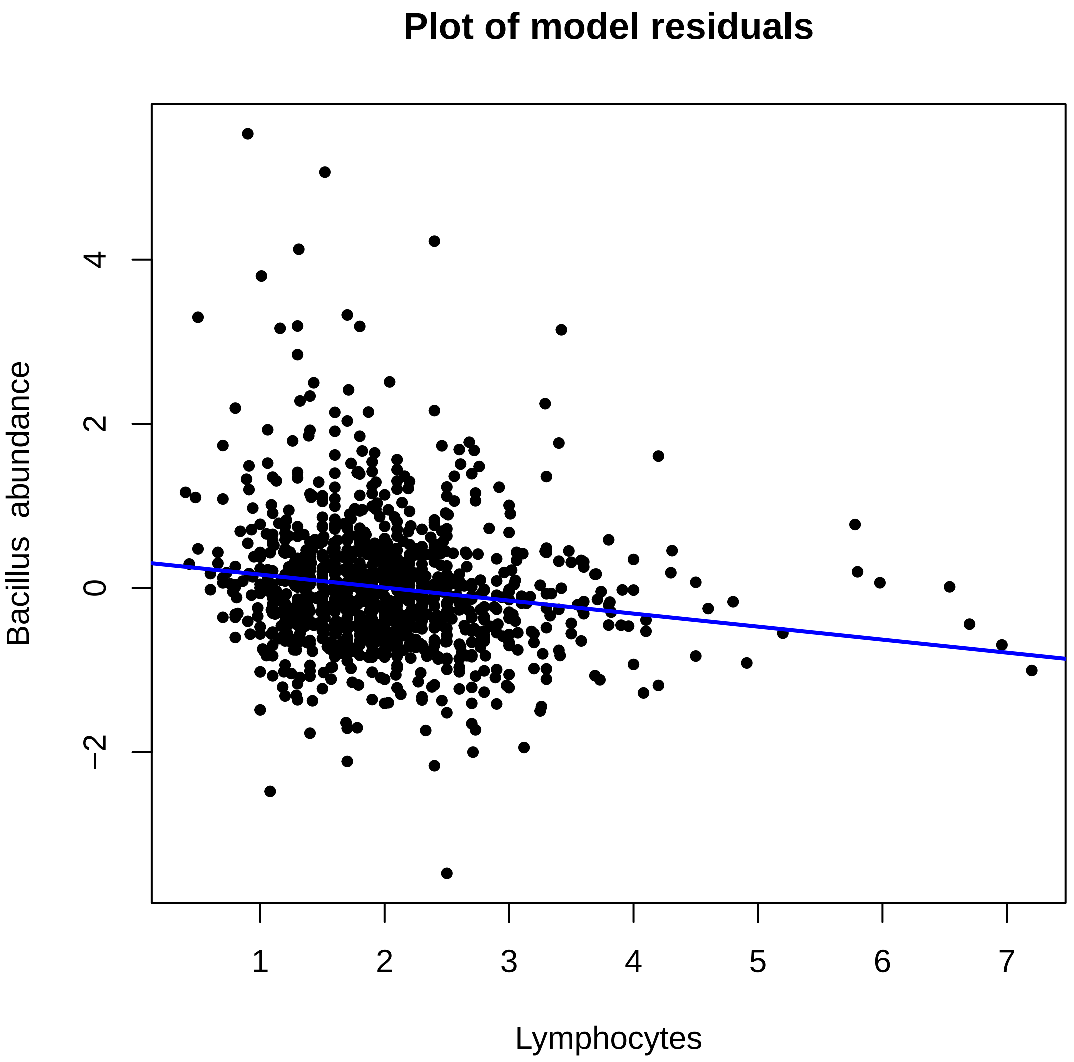

Plot of model residuals
Bacillus abundance
Lymphocytes
4
2
0
−2
1
2
3
4
5
6
7

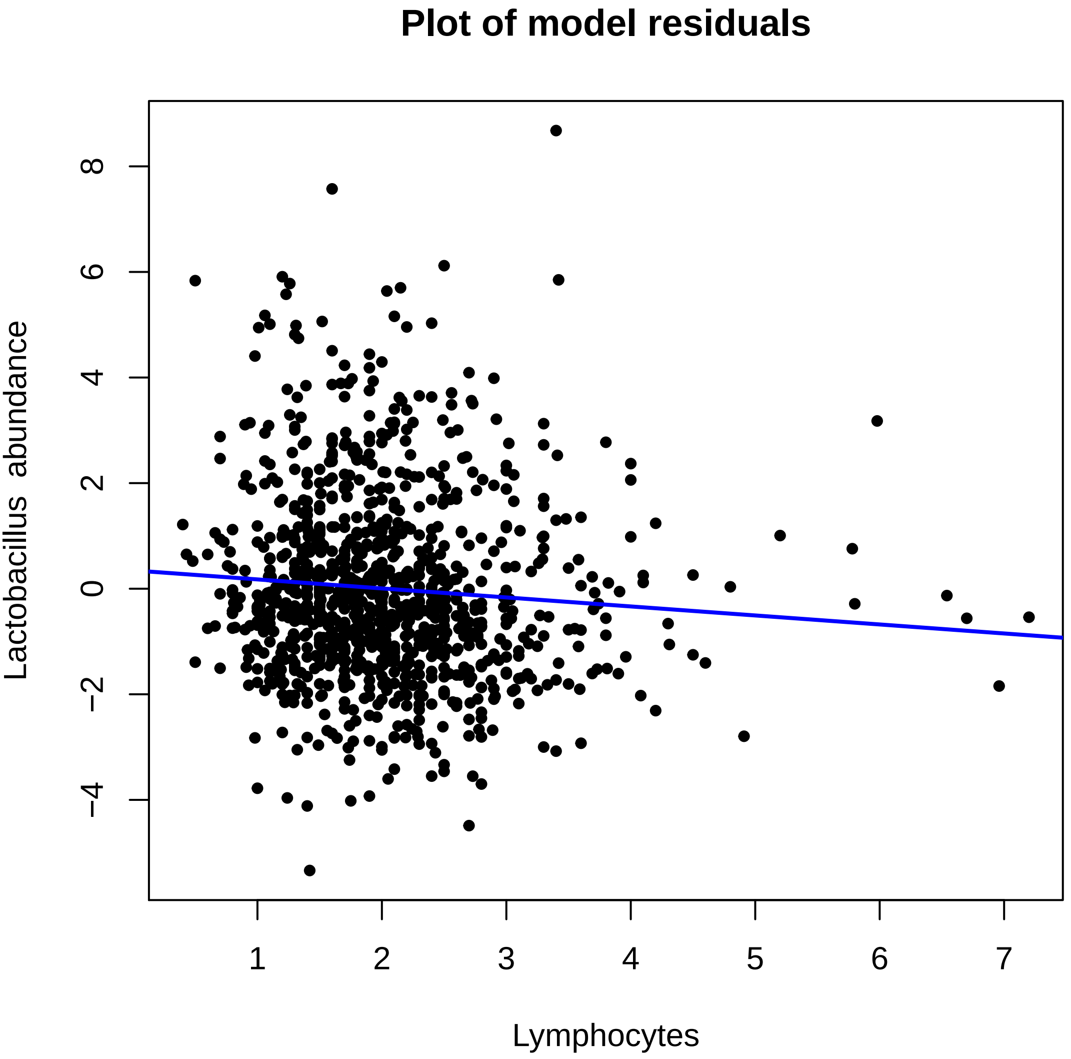

Plot of model residuals
Lactobacillus abundance
Lymphocytes
8
6
4
2
0
−2
−4
1
2
3
4
5
6
7

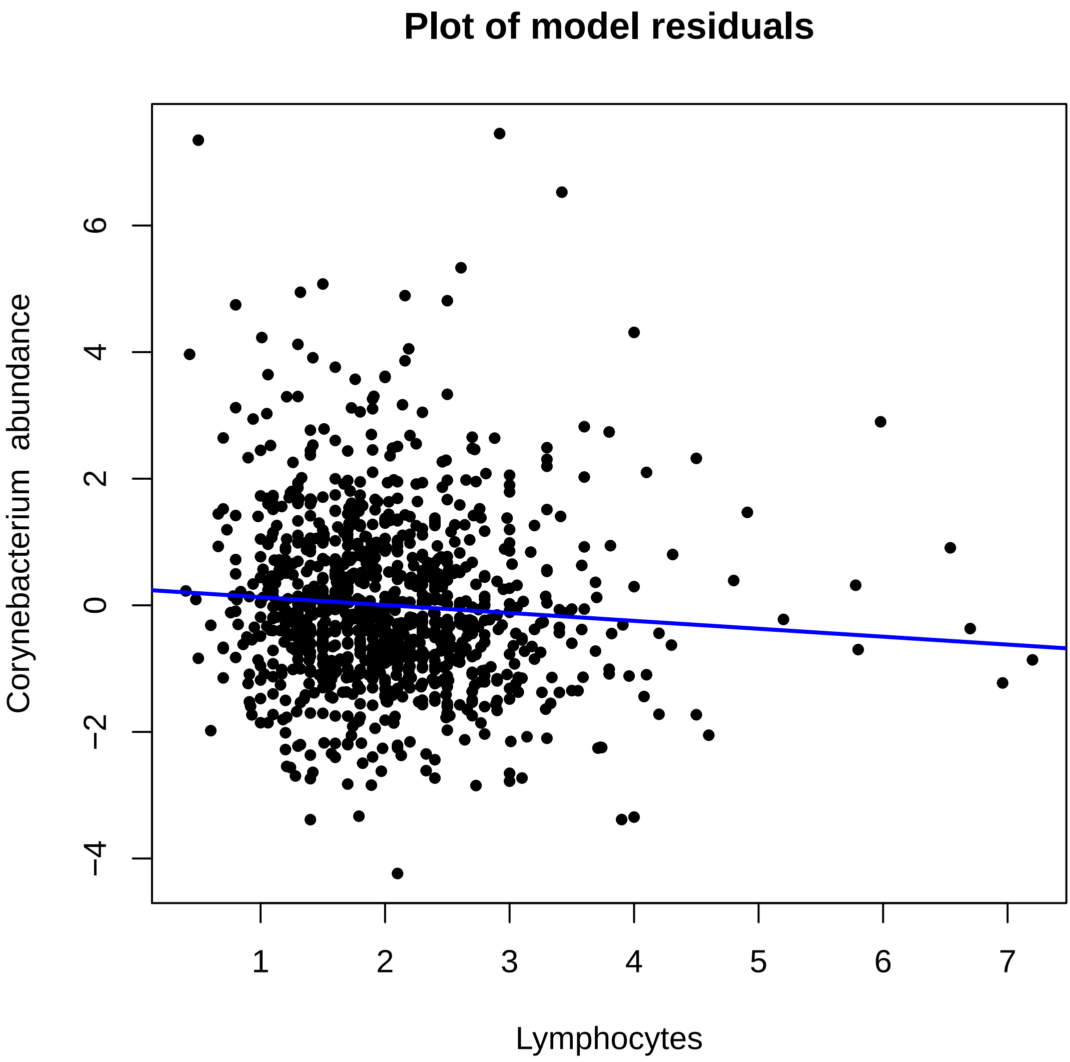

Plot of model residuals
Corynebacterium abundance
Lymphocytes
6
4
2
0
−2
−4
1
2
3
4
5
6
7

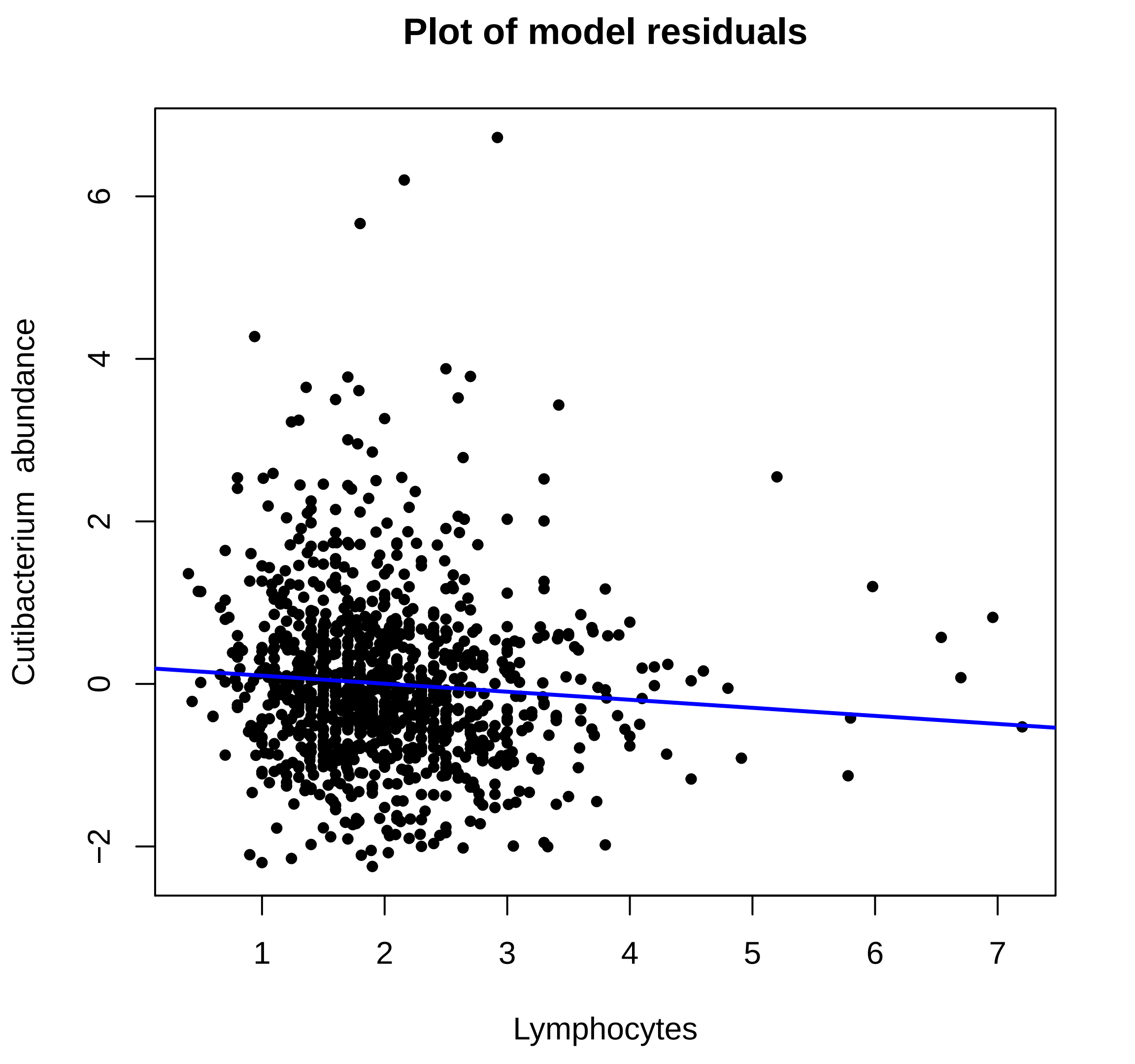

Plot of model residuals
Cutibacterium abundance
Lymphocytes
−2
0
2
4
6
1
2
3
4
5
6
7

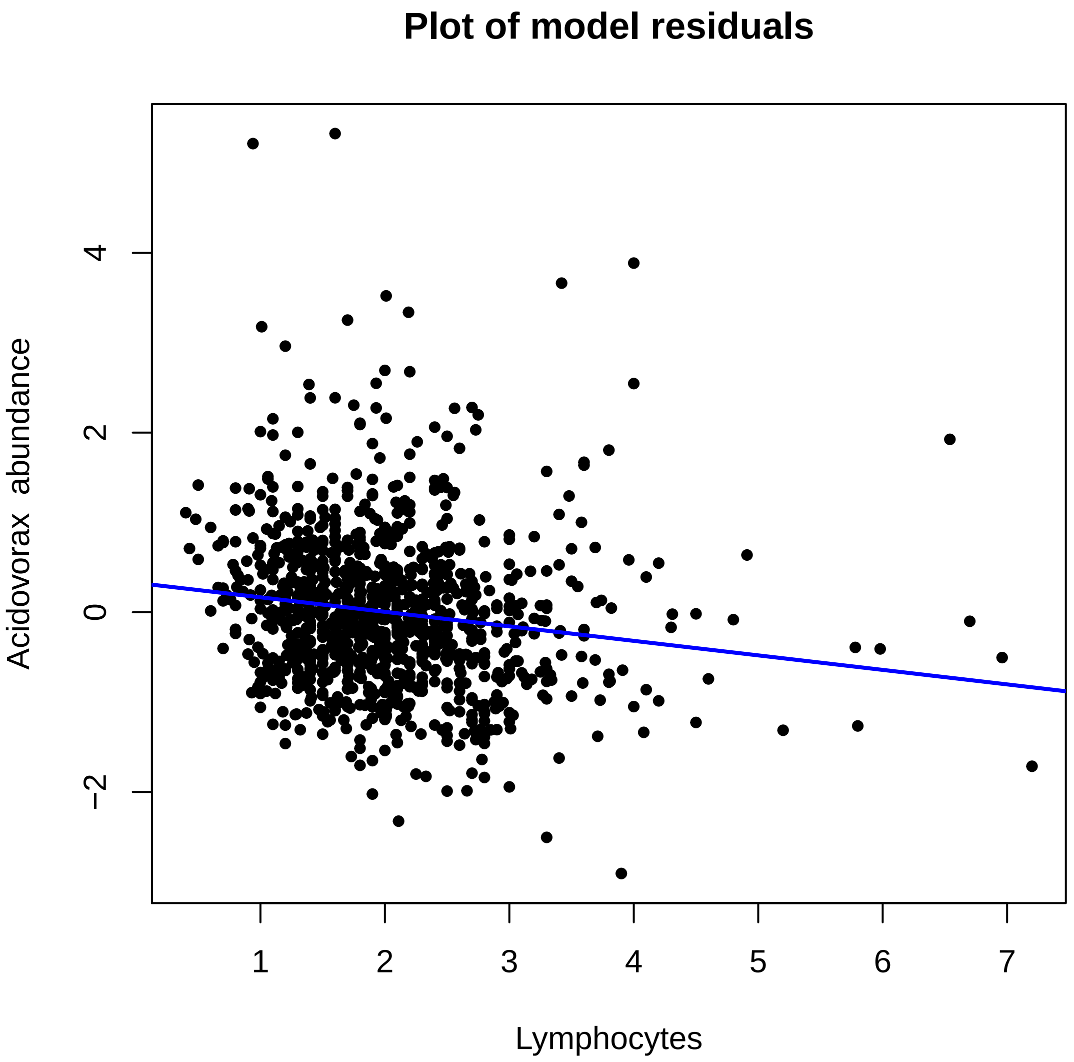

Plot of model residuals
Acidovorax abundance
Lymphocytes
4
2
0
−2
1
2
3
4
5
6
7

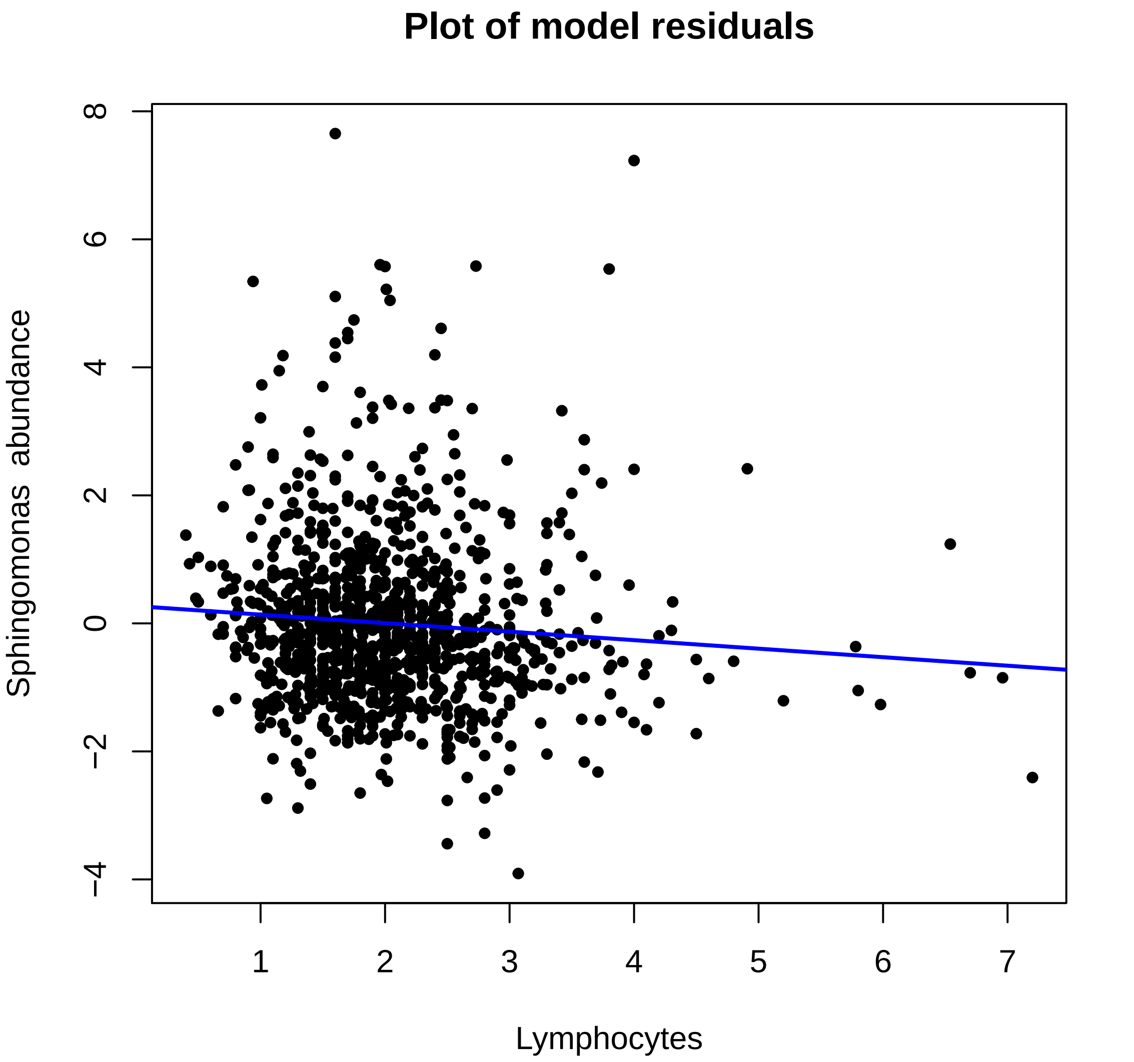

Plot of model residuals
Sphingomonas abundance
Lymphocytes
8
6
4
2
0
−2
−4
1
2
3
4
5
6
7

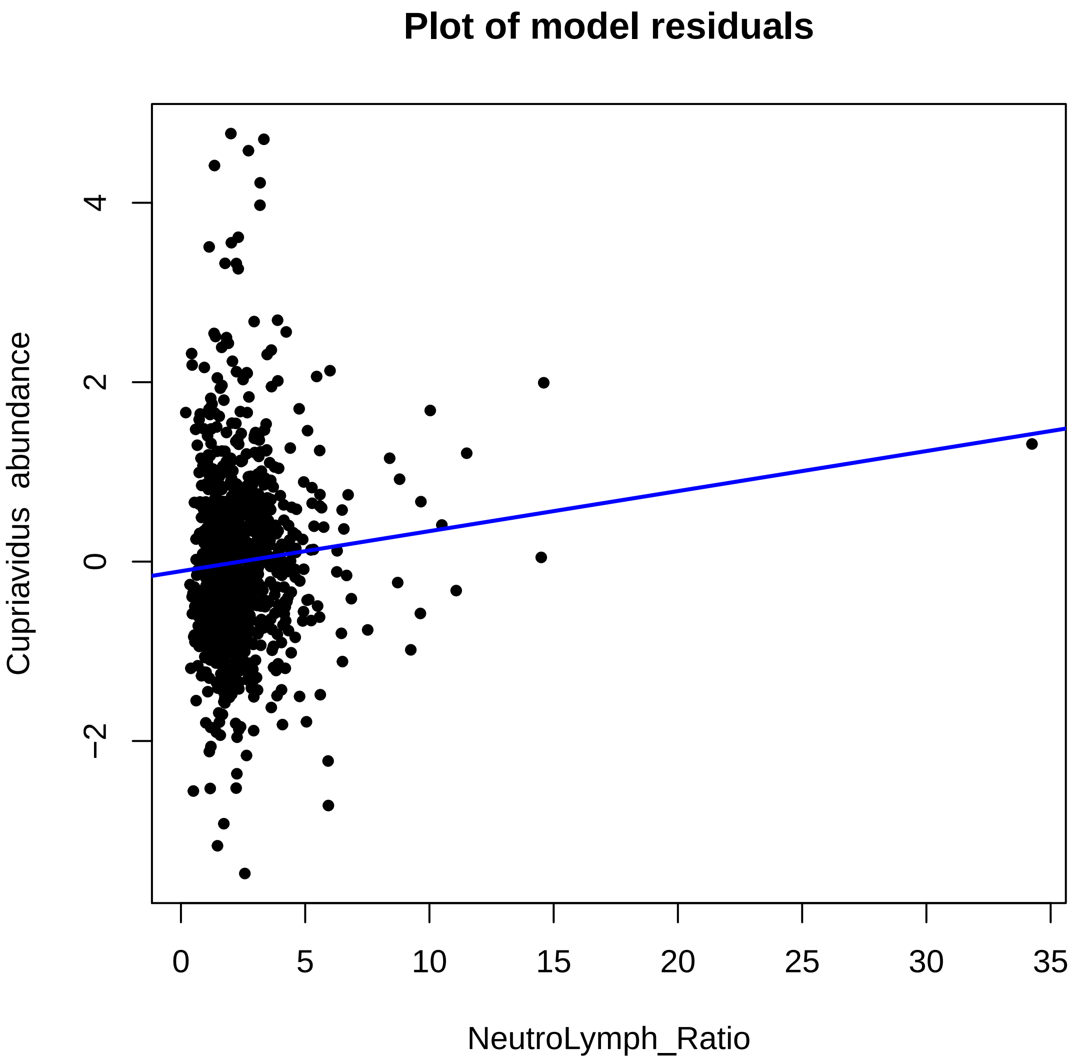
Plot of model residuals
Cupriavidus_abundance
NeutroLymph_Ratio
4
2
0
−2
0
5
10
15
20
25
30
35

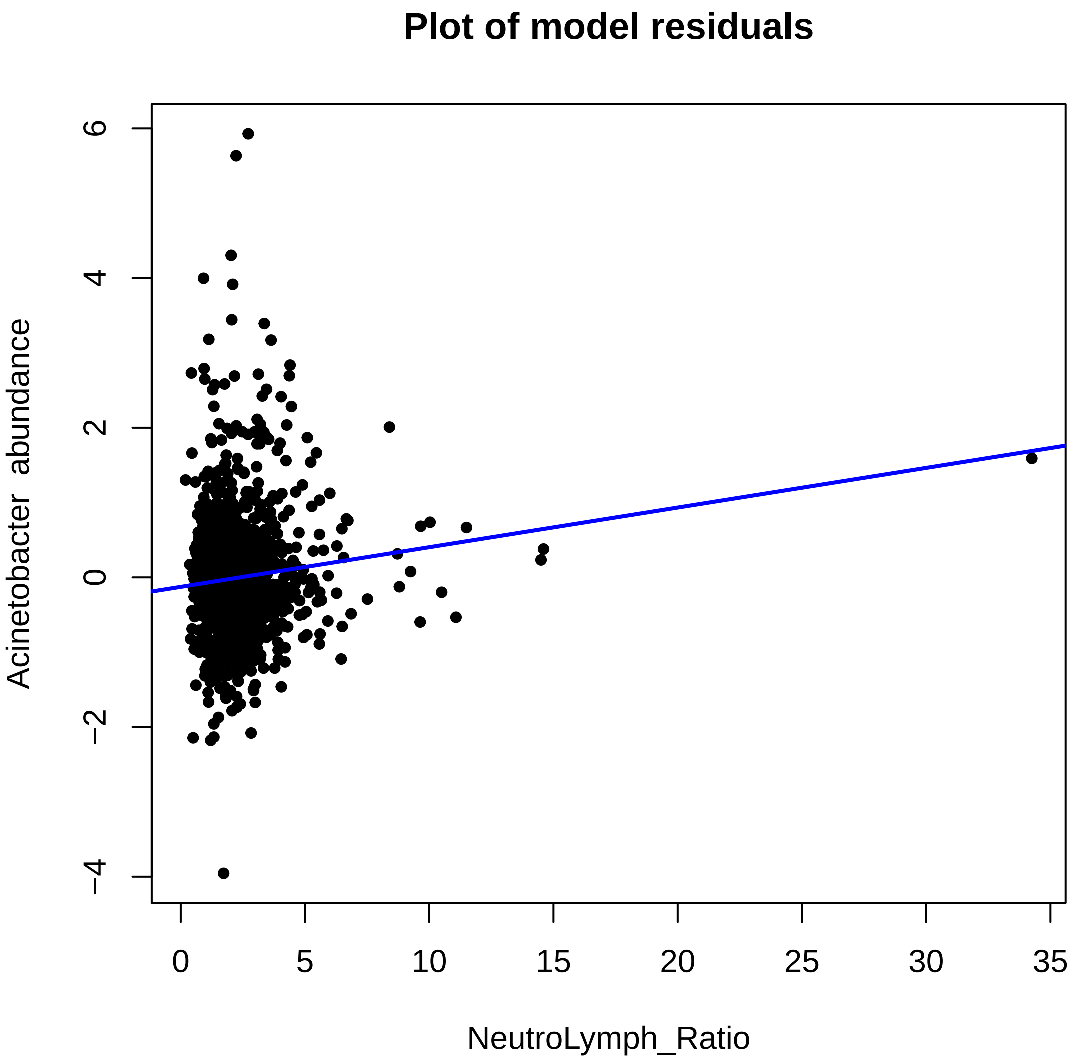
Plot of model residuals
Acinetobacter abundance
NeutroLymph_Ratio
6
4
2
0
−2
−4
0
5
10
15
20
25
30
35

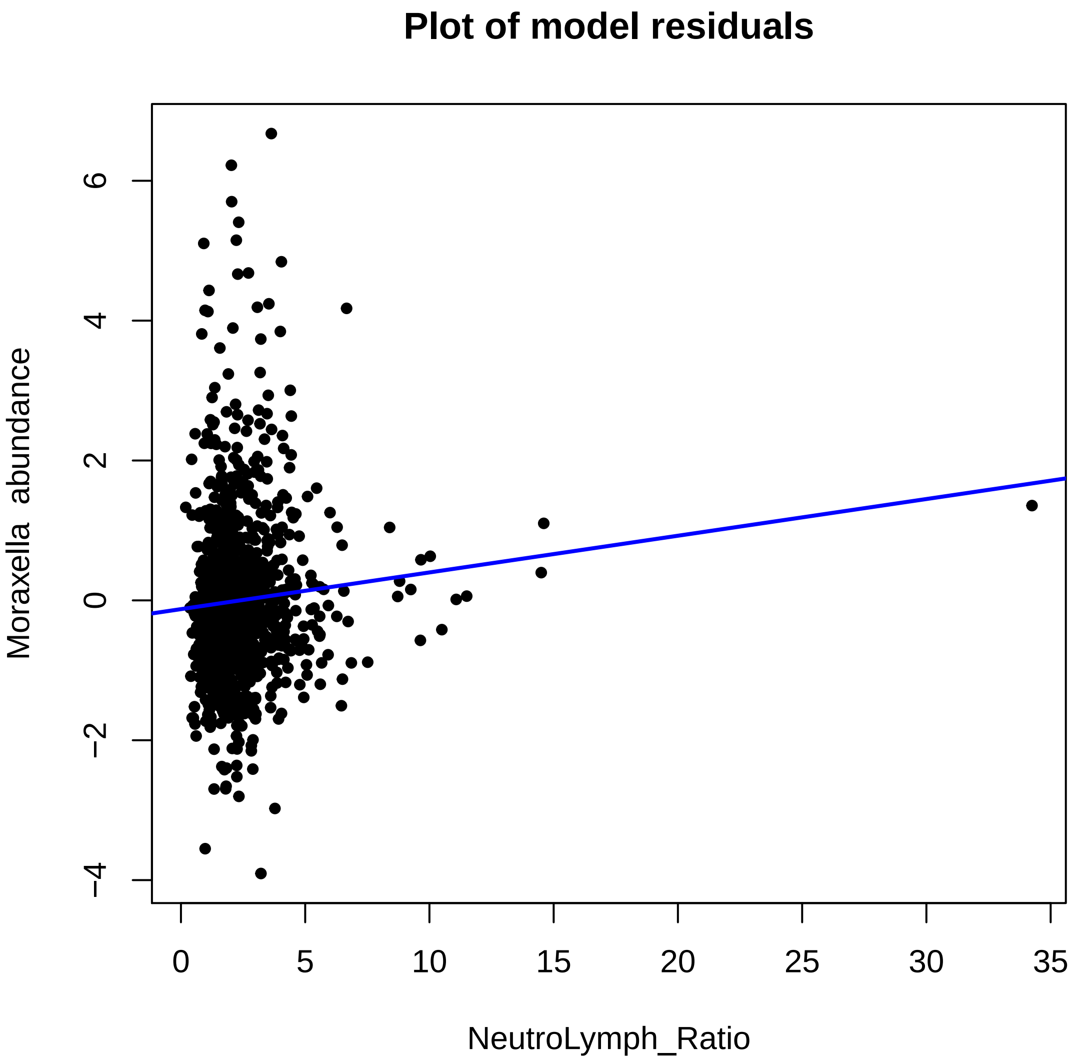

Plot of model residuals
Moraxella abundance
NeutroLymph_Ratio
6
4
2
0
−2
−4
0
5
10
15
20
25
30
35

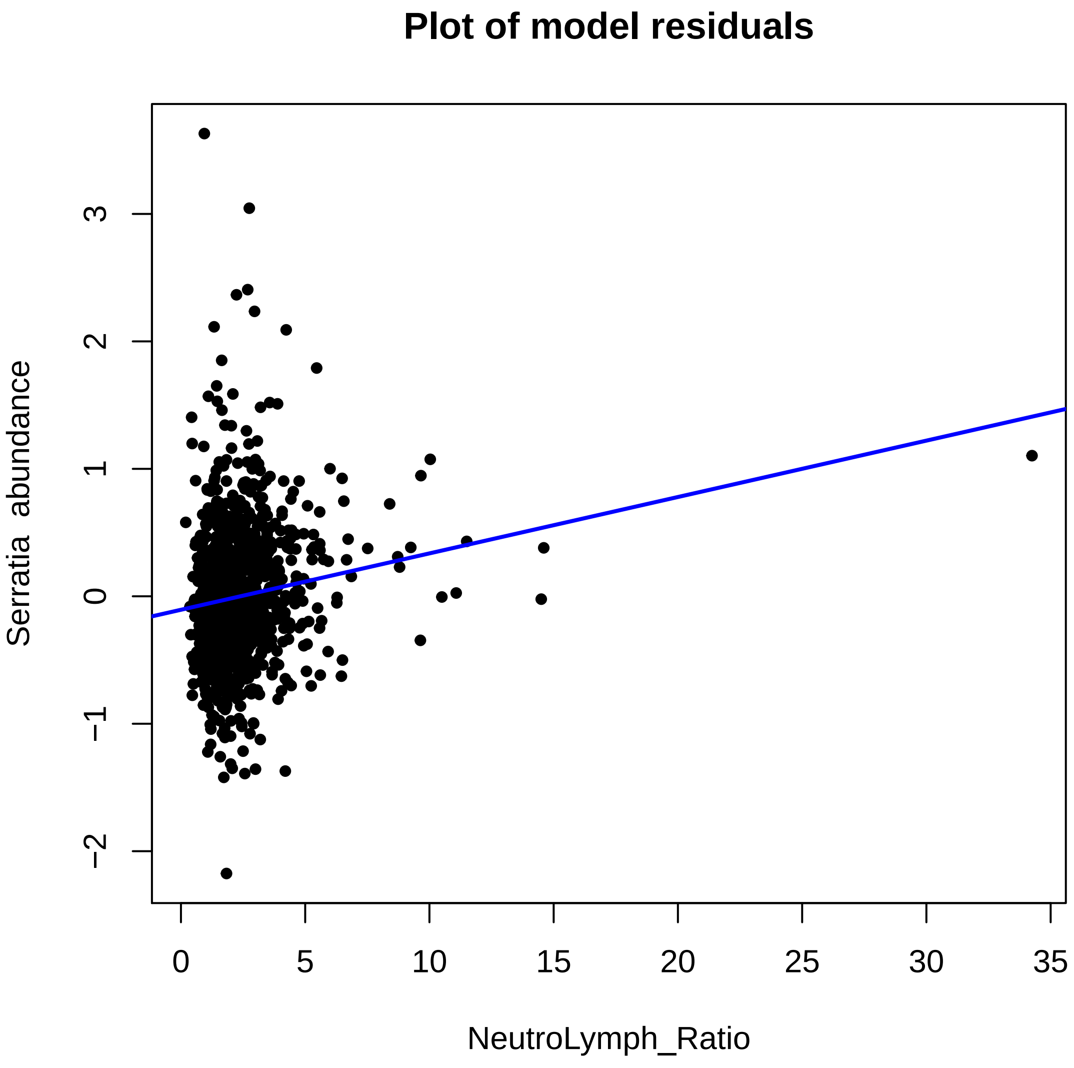

Plot of model residuals
Serratia abundance
NeutroLymph_Ratio
3
2
1
0
−1
−2
0
5
10
15
20
25
30
35

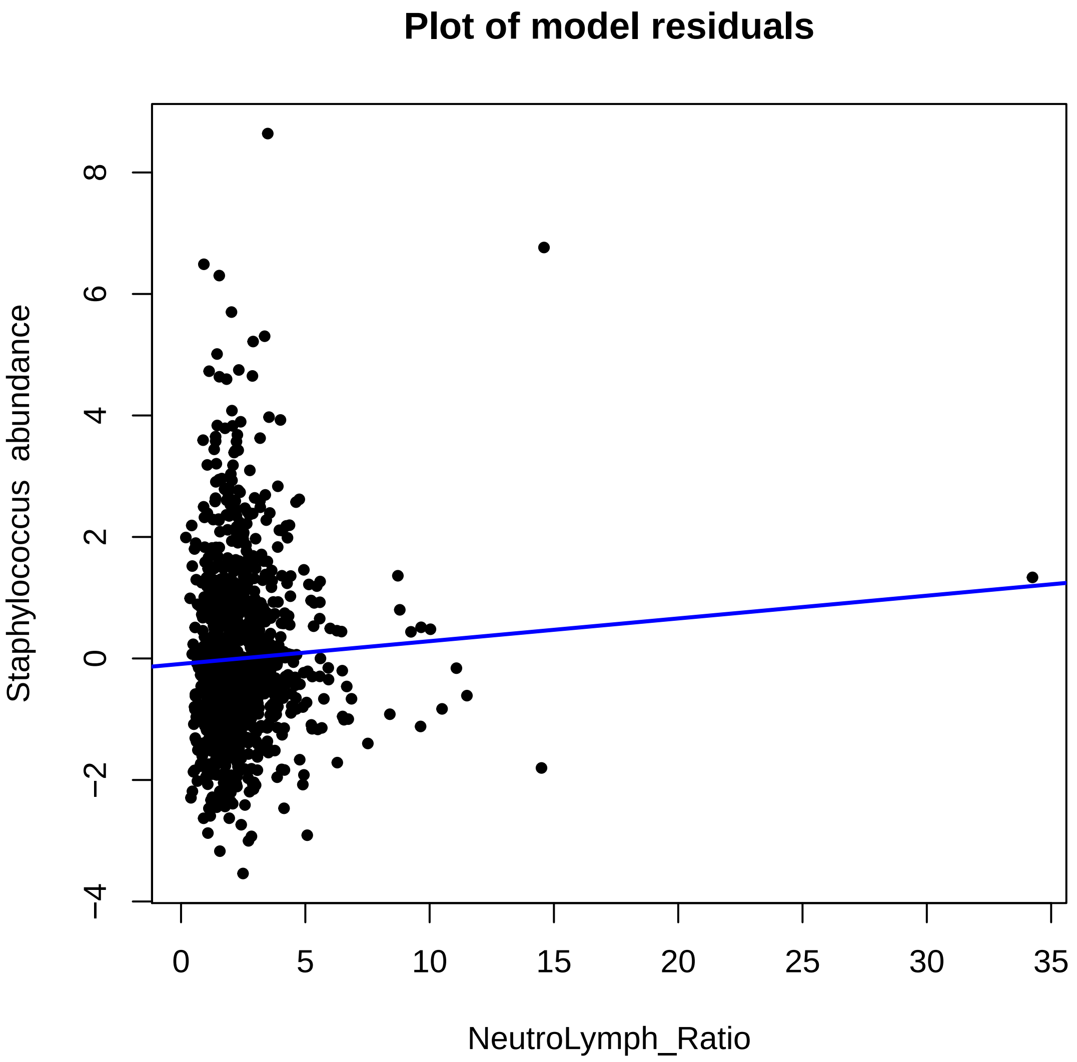
Plot of model residuals
Staphylococcus abundance
NeutroLymph_Ratio
−4
−2
0
2
4
6
8
0
5
10
15
20
25
30
35

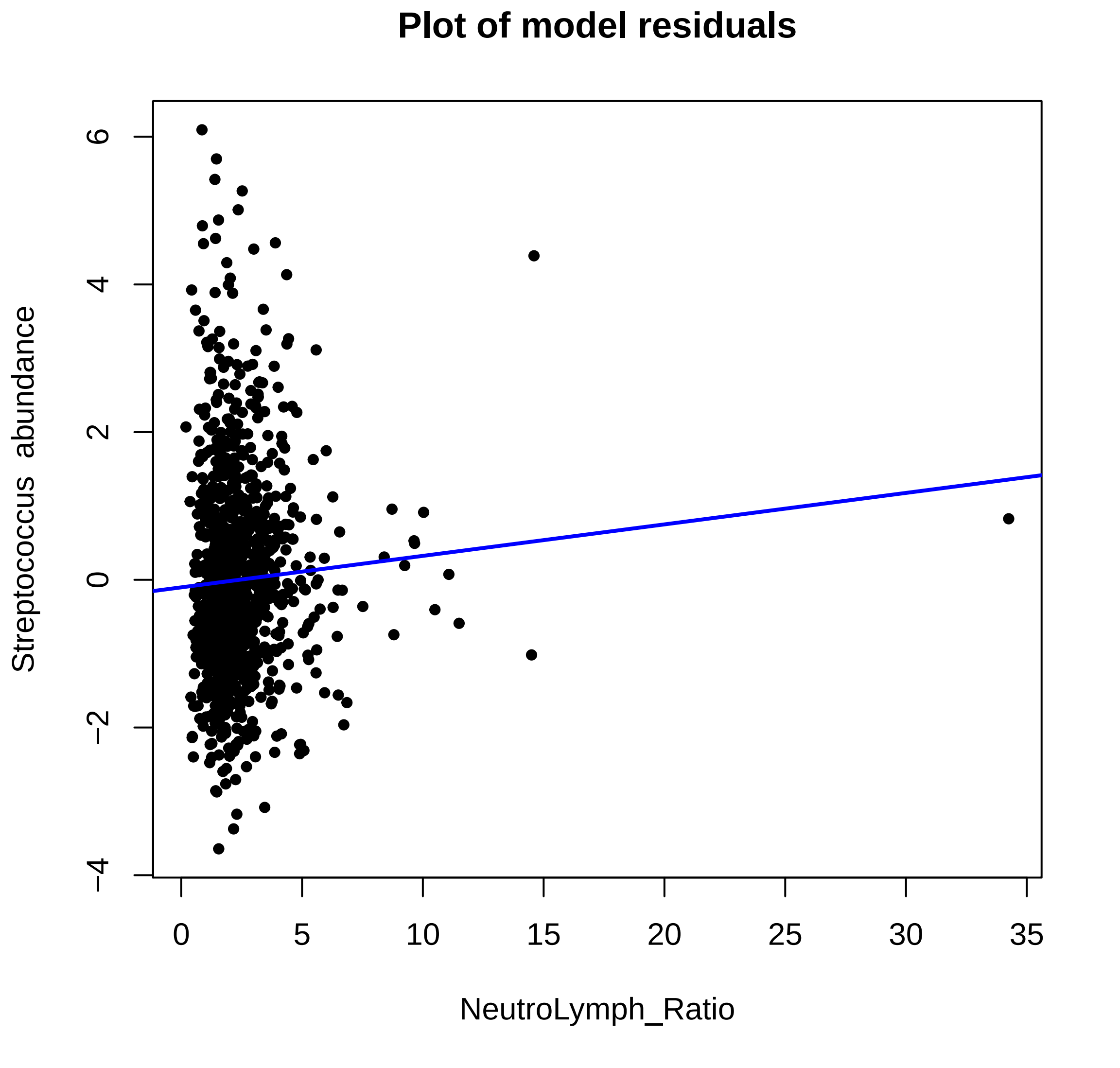

Plot of model residuals
Streptococcus abundance
NeutroLymph_Ratio
6
4
2
0
−2
−4
0
5
10
15
20
25
30
35

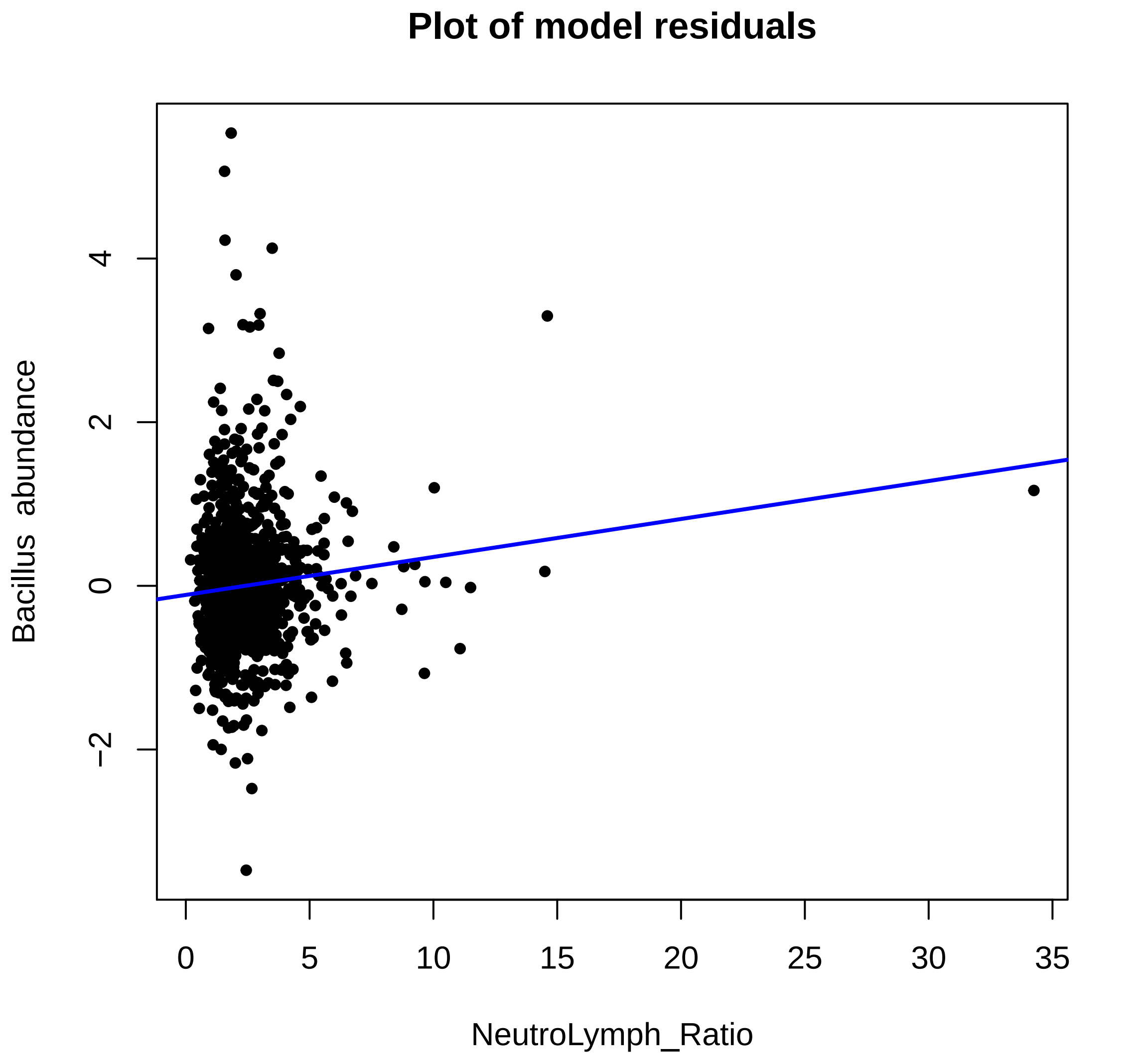

Plot of model residuals
Bacillus abundance
NeutroLymph_Ratio
−2
0
2
4
0
5
10
15
20
25
30
35

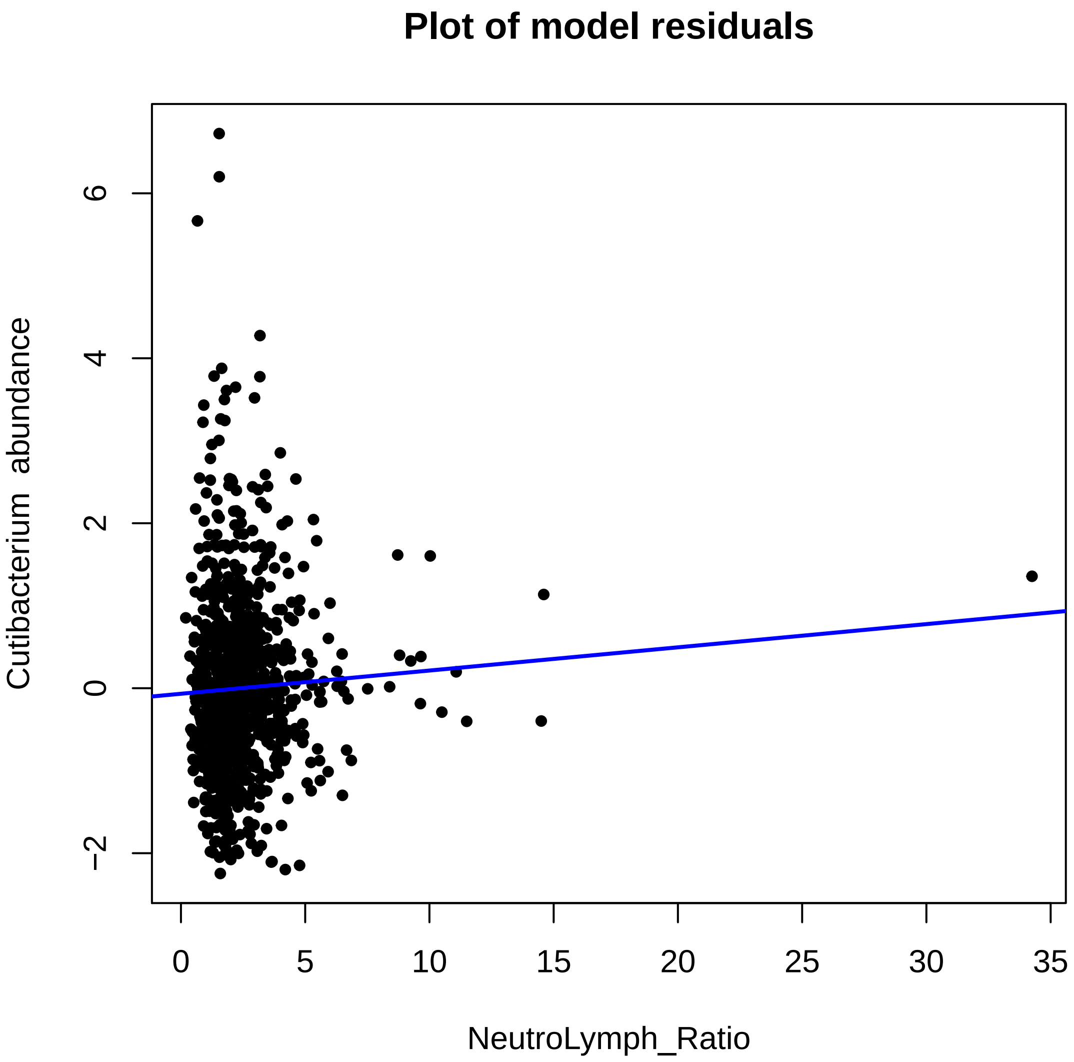
Plot of model residuals
Cutibacterium abundance
−2
0
2
4
6
0
5
10
15
20
25
30
35
NeutroLymph_Ratio

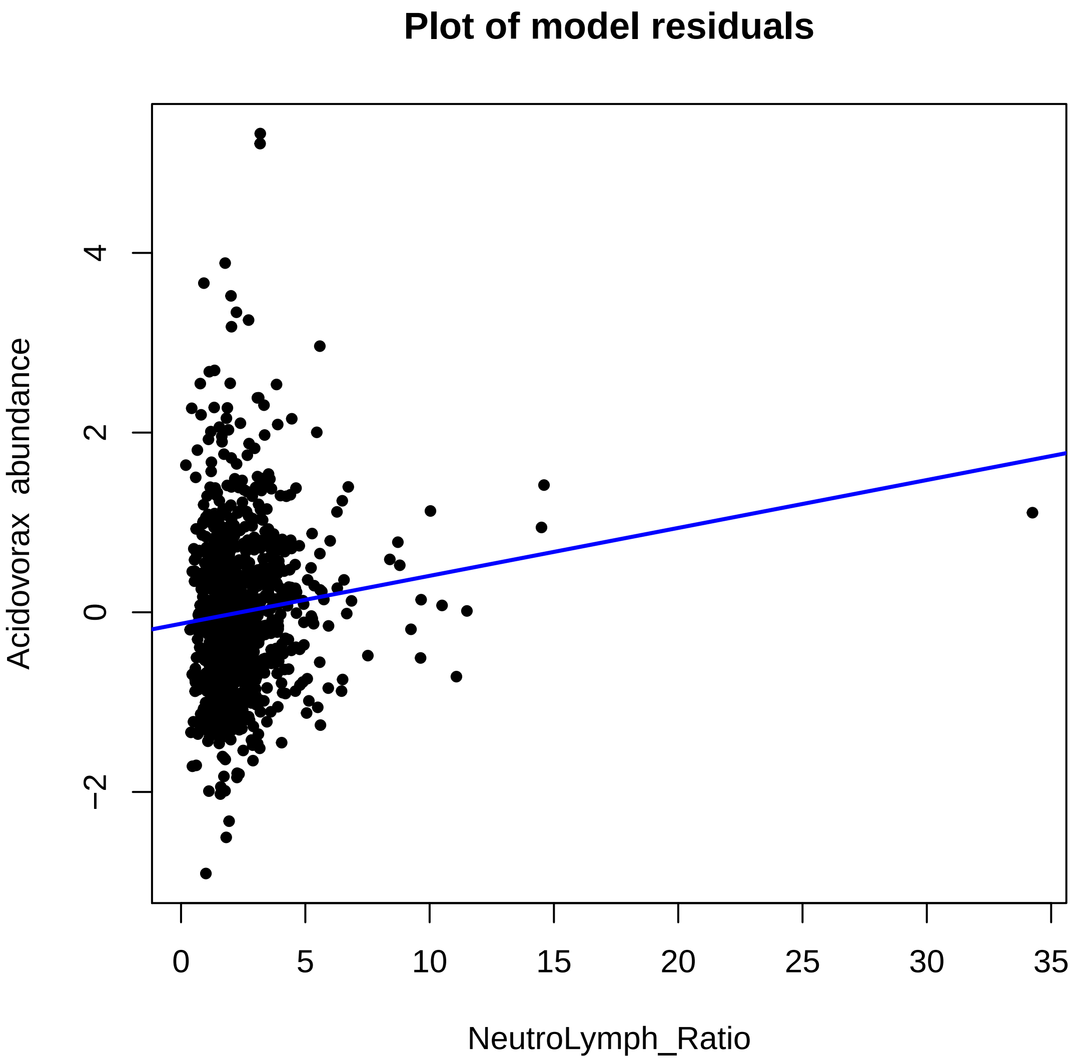

Plot of model residuals
Acidovorax abundance
NeutroLymph_Ratio
4
2
0
−2
0
5
10
15
20
25
30
35

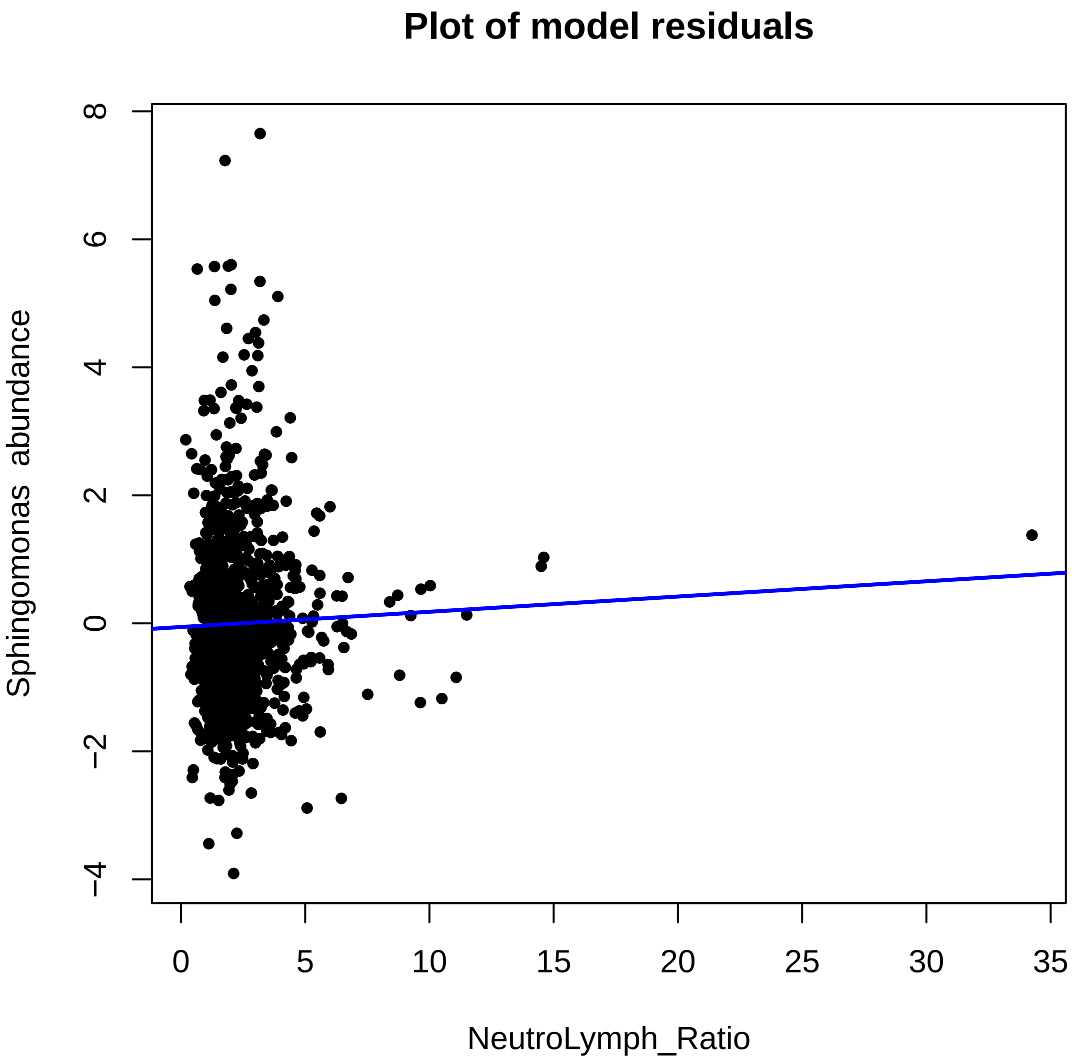

Plot of model residuals
Sphingomonas abundance
NeutroLymph_Ratio
8
6
4
2
0
−2
−4
0
5
10
15
20
25
30
35

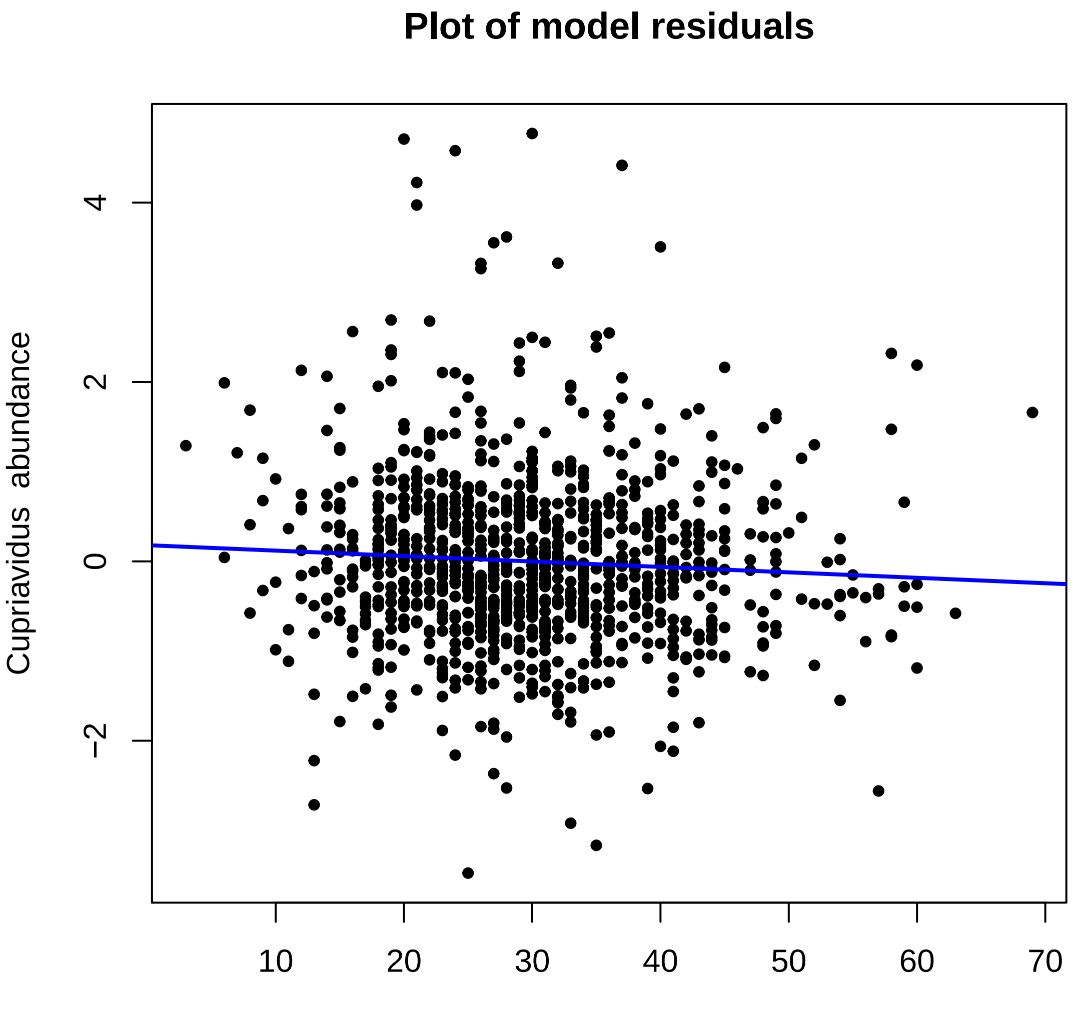
Plot of model residuals
Cupriavidus_abundance
Lymphocyte_pct
4
2
0
−2
10
20
30
40
50
60
70

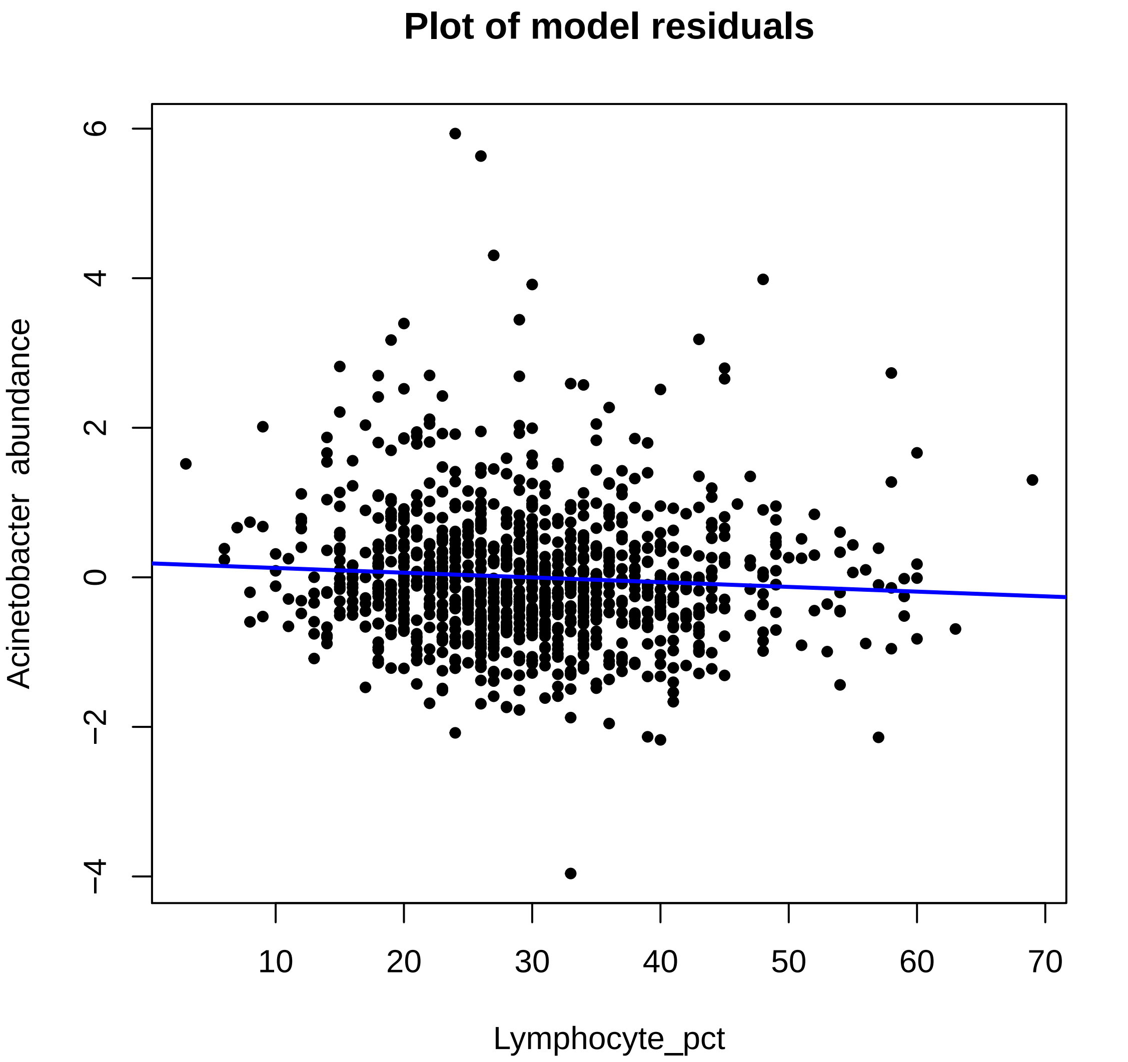

Plot of model residuals
Acinetobacter abundance
Lymphocyte_pct
−4
−2
0
2
4
6
10
20
30
40
50
60
70

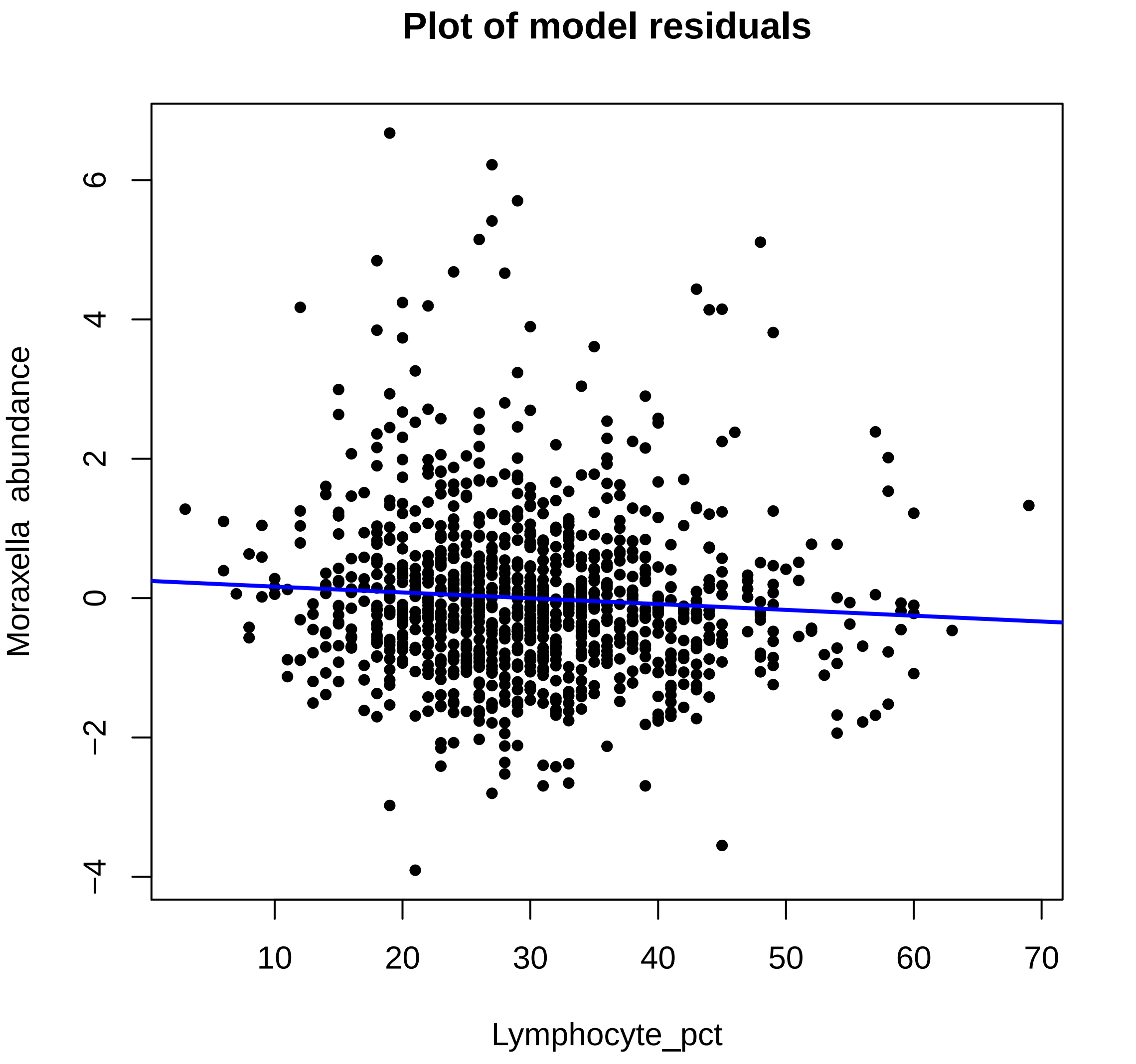
Plot of model residuals
Moraxella abundance
Lymphocyte_pct
6
4
2
0
−2
−4
10
20
30
40
50
60
70

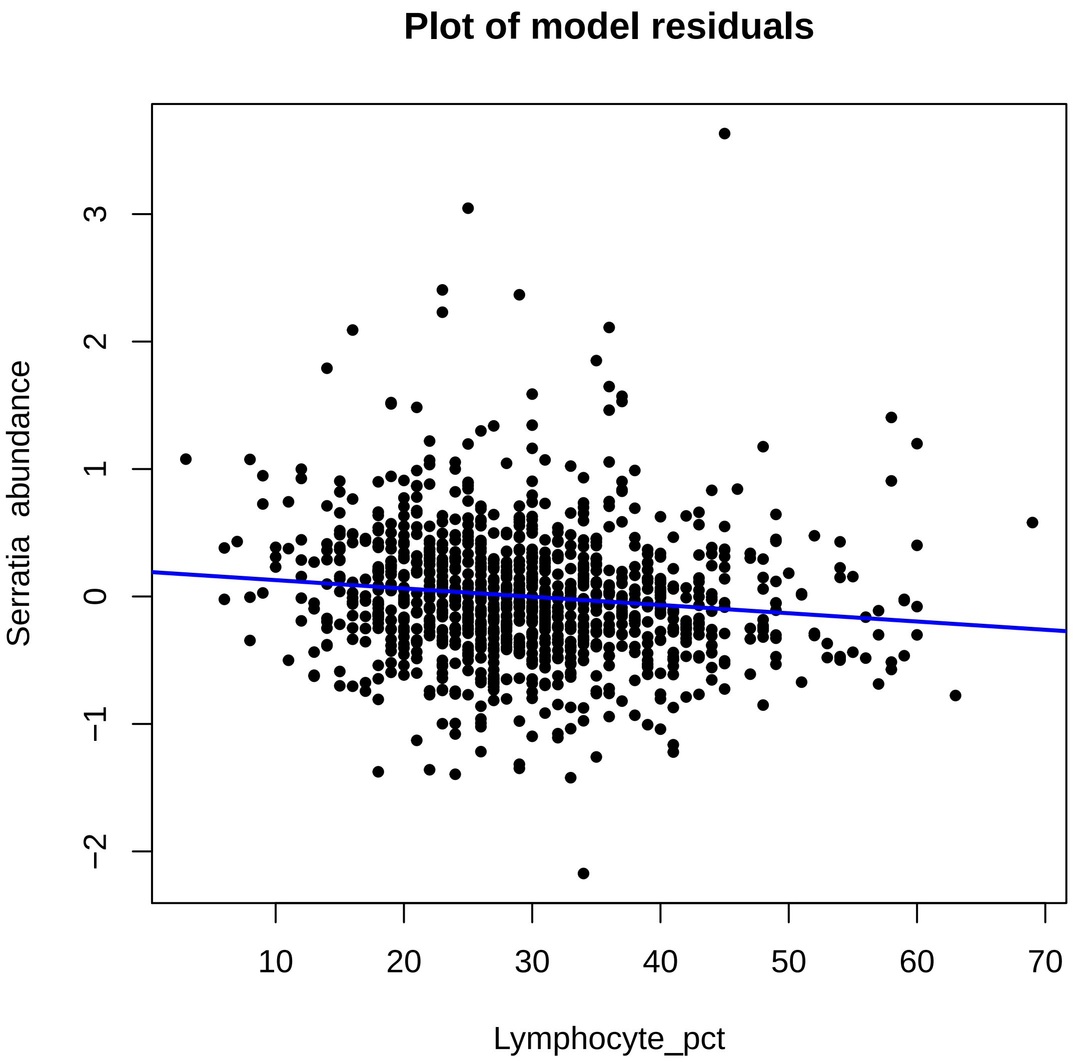

Plot of model residuals
Serratia abundance
Lymphocyte_pct
−2
−1
0
1
2
3
10
20
30
40
50
60
70

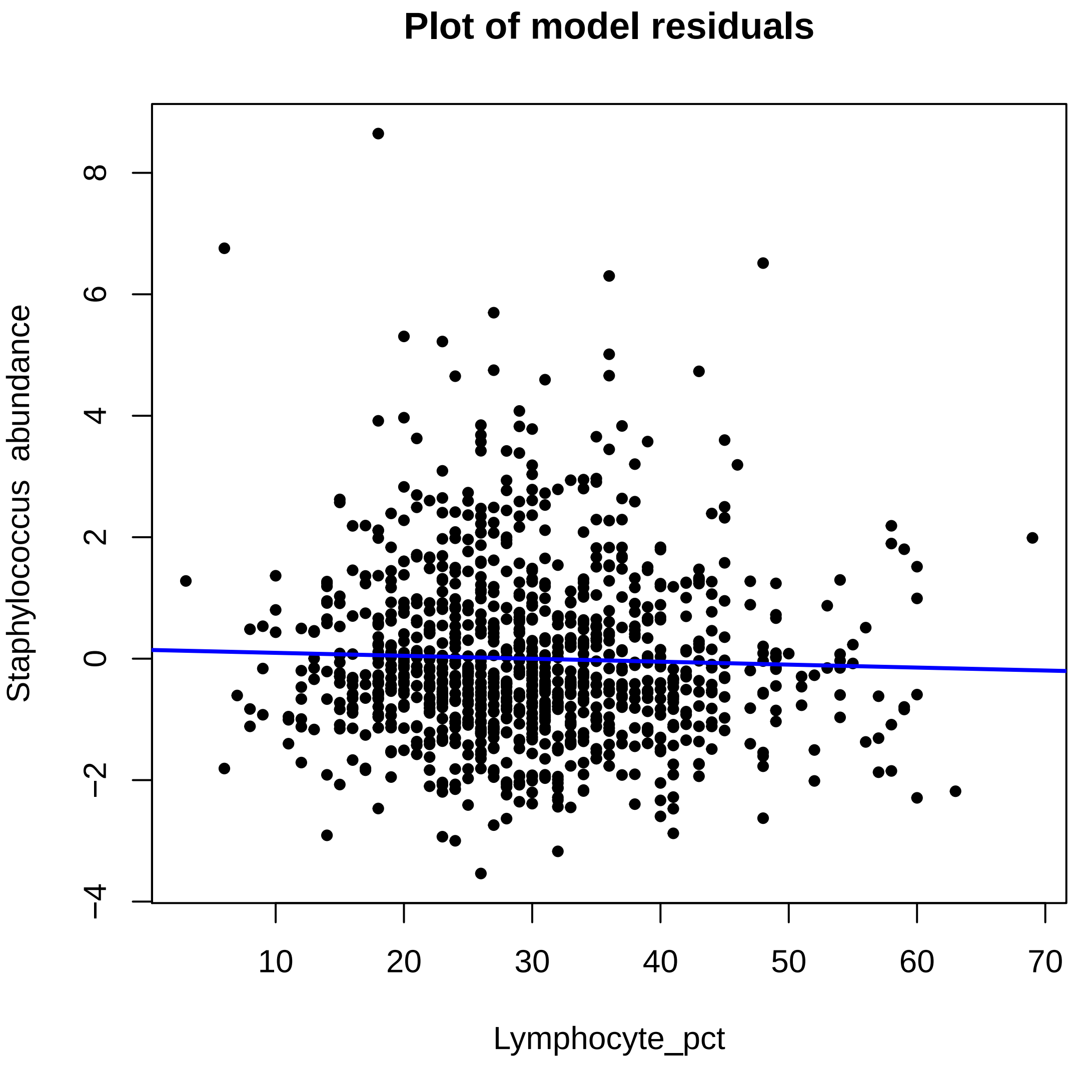

Plot of model residuals
Staphylococcus abundance
Lymphocyte_pct
8
6
4
2
0
−2
−4
10
20
30
40
50
60
70

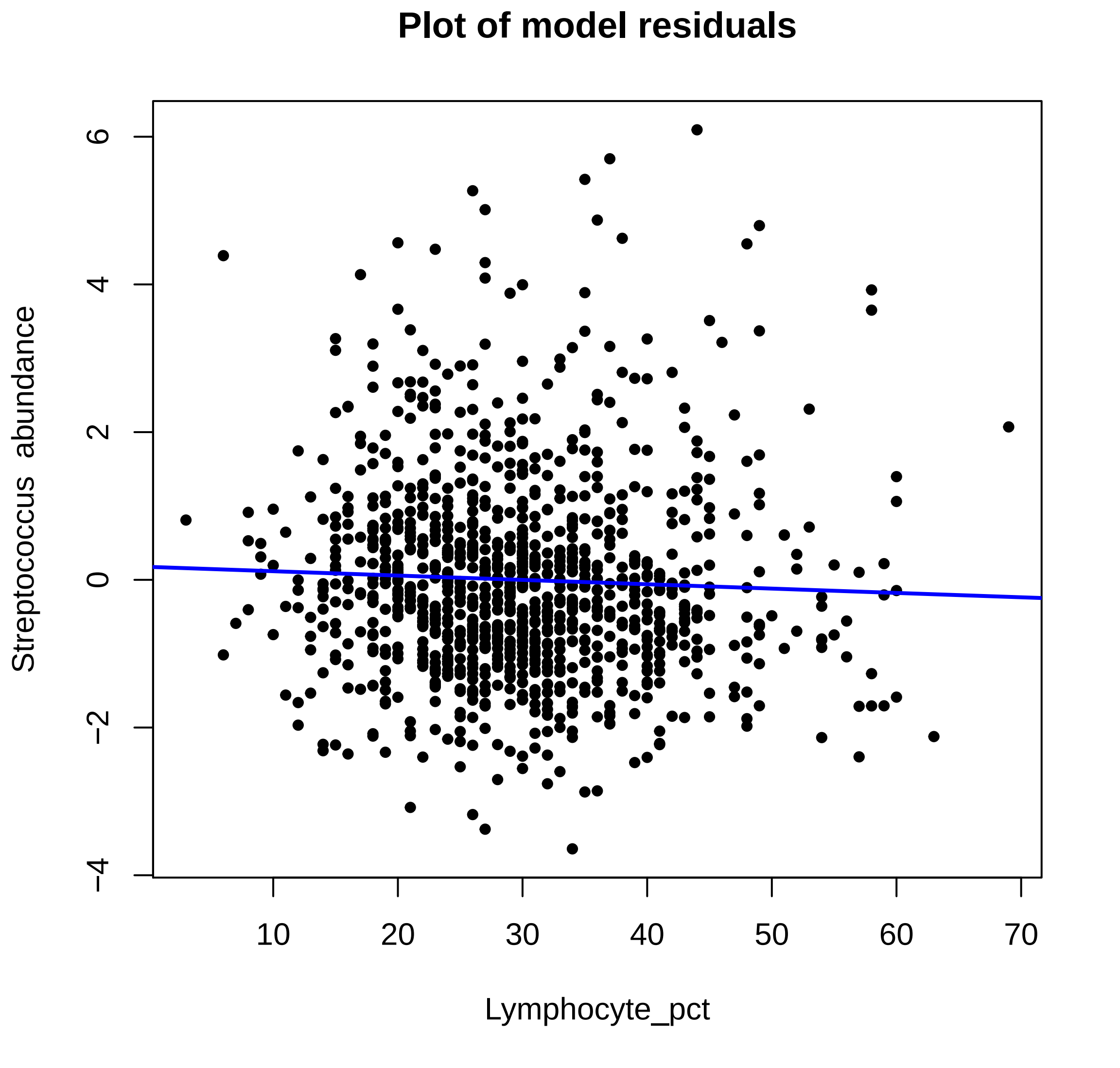

Plot of model residuals
Streptococcus abundance
Lymphocyte_pct
6
4
2
0
−2
−4
10
20
30
40
50
60
70

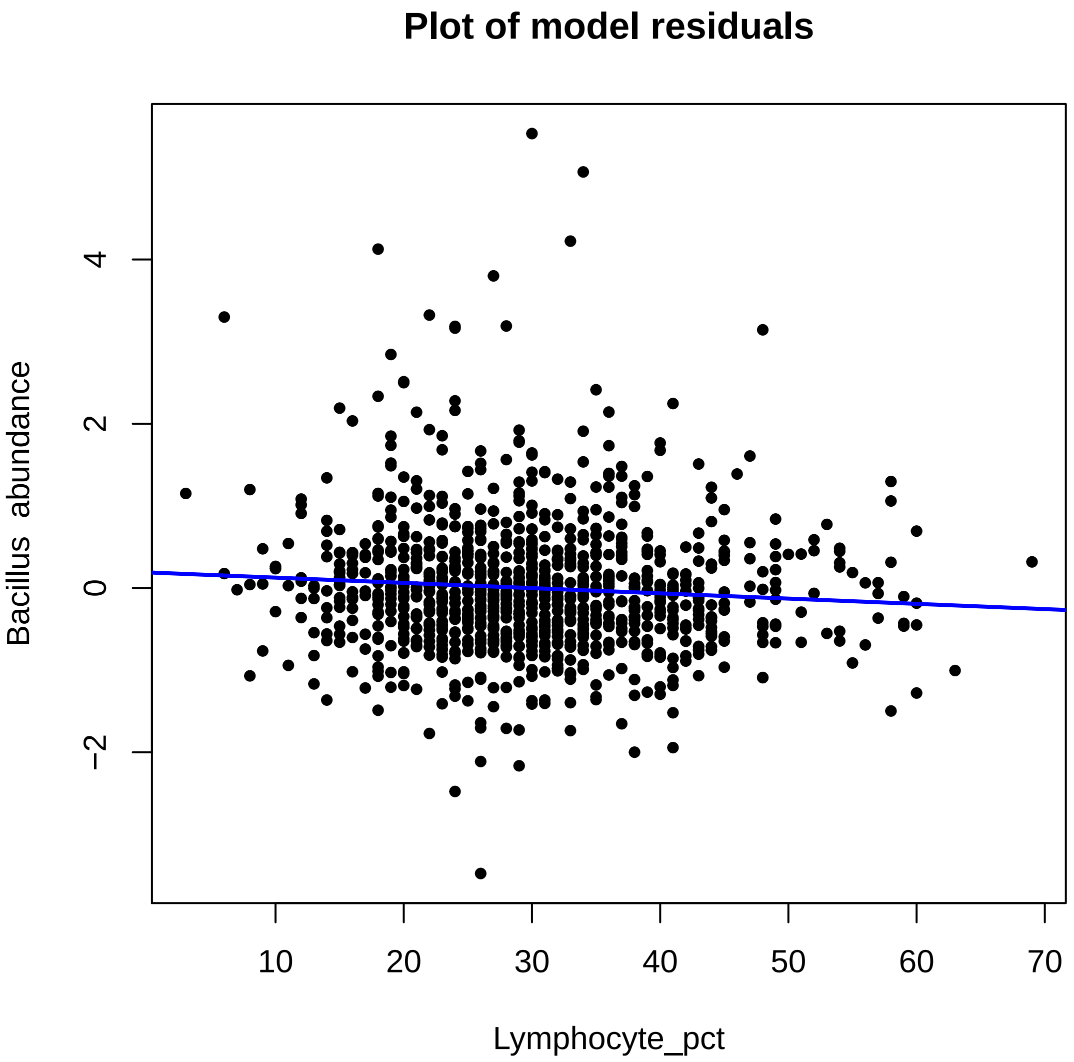

Plot of model residuals
Bacillus abundance
Lymphocyte_pct
4
2
0
−2
10
20
30
40
50
60
70

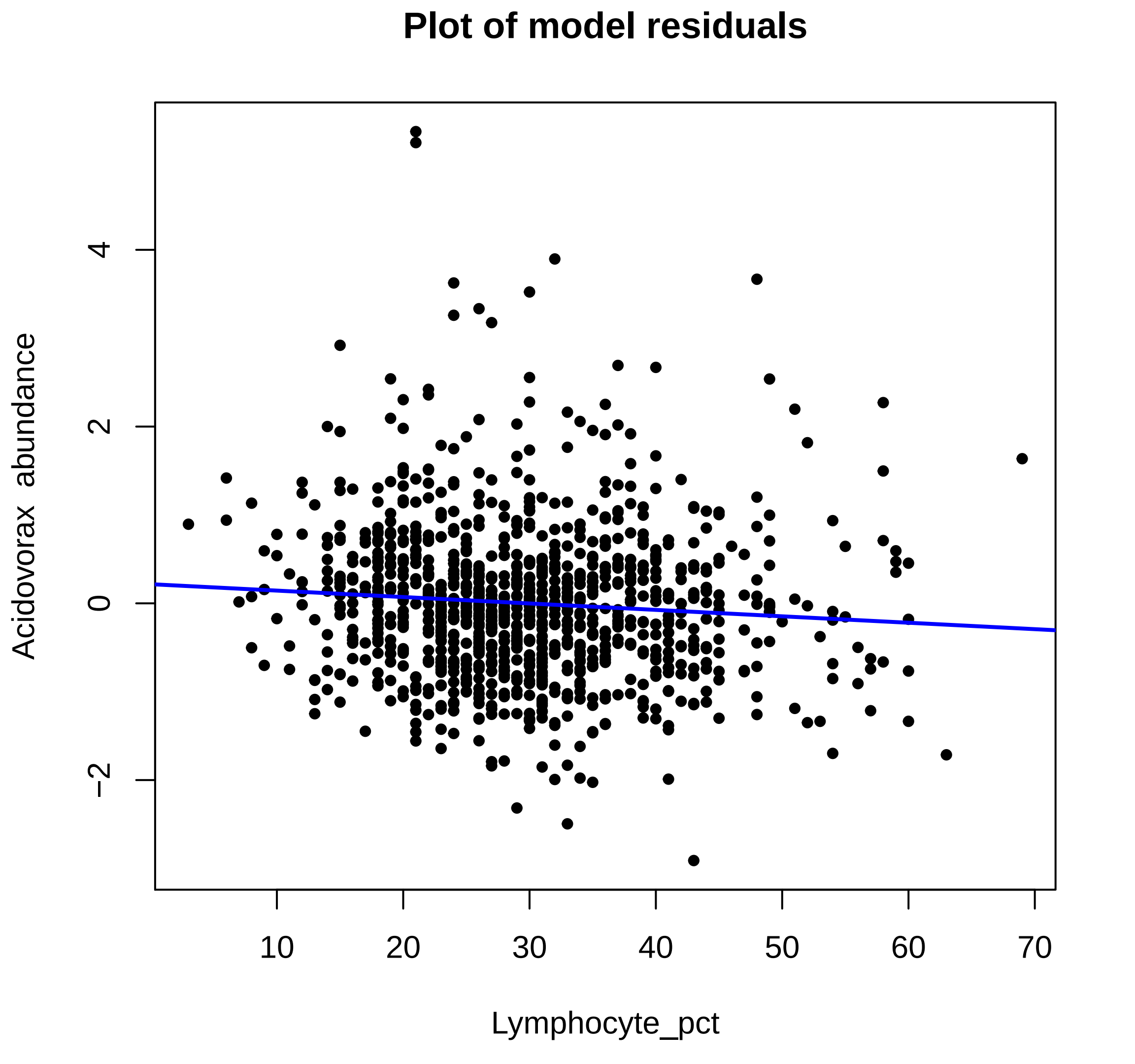
Plot of model residuals
Acidovorax abundance
4
2
0
−2
10
20
30
40
50
60
70
Lymphocyte_pct

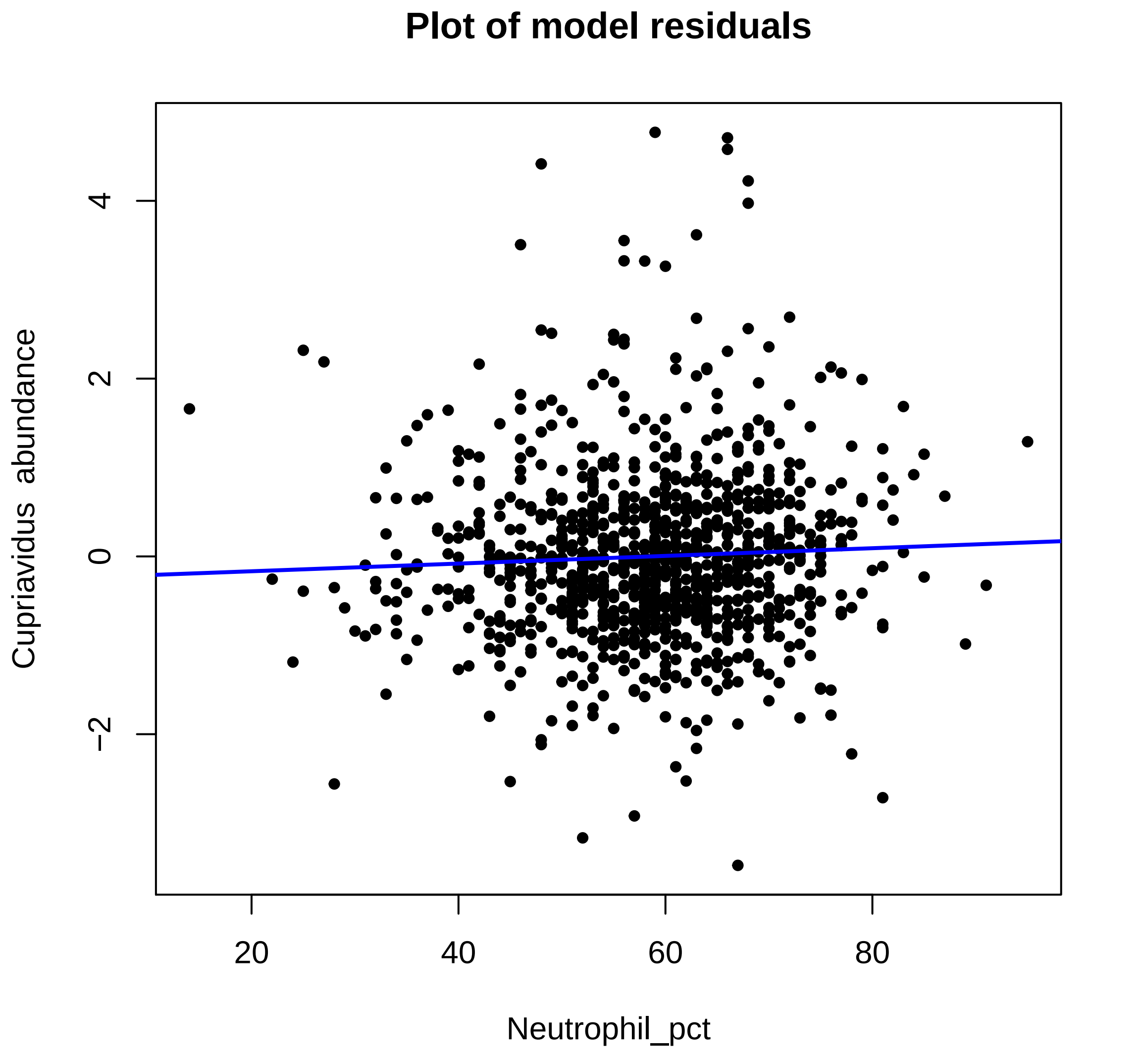
Plot of model residuals
Cupriavidus_abundance
Neutrophil_pct
4
2
0
−2
20
40
60
80

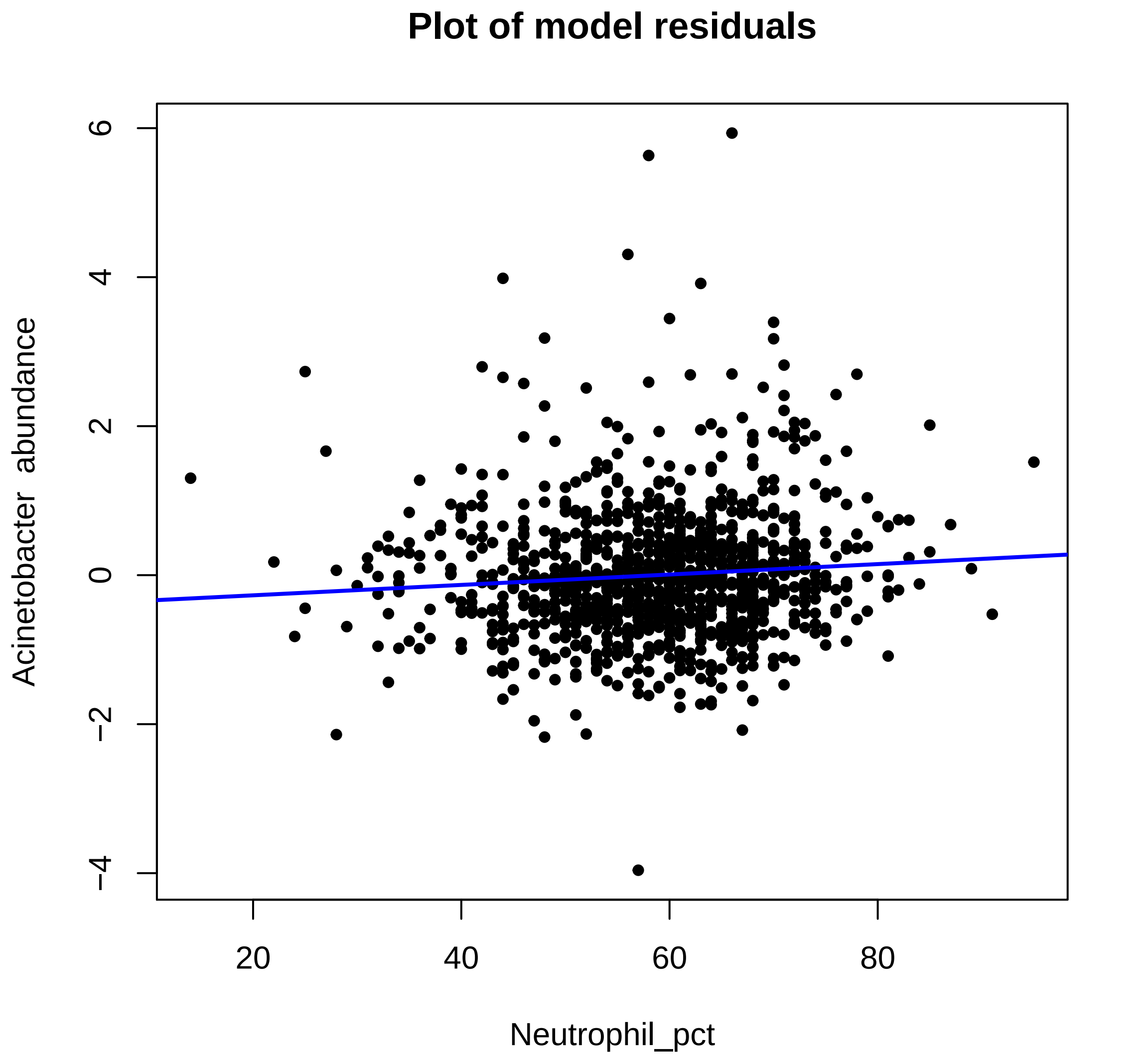
Plot of model residuals
Acinetobacter abundance
Neutrophil_pct
6
4
2
0
−2
−4
20
40
60
80

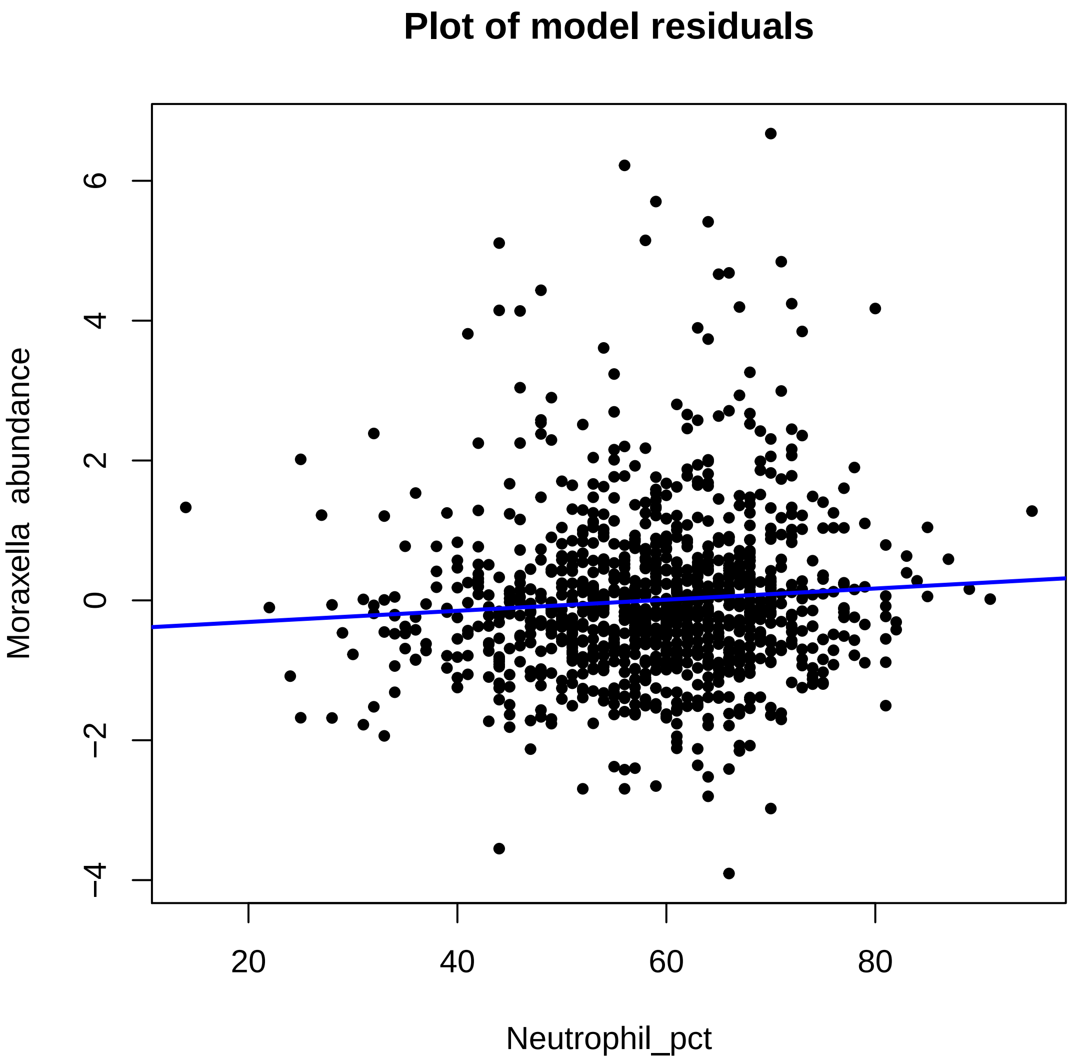

Plot of model residuals
Moraxella abundance
Neutrophil_pct
−4
−2
0
2
4
6
20
40
60
80

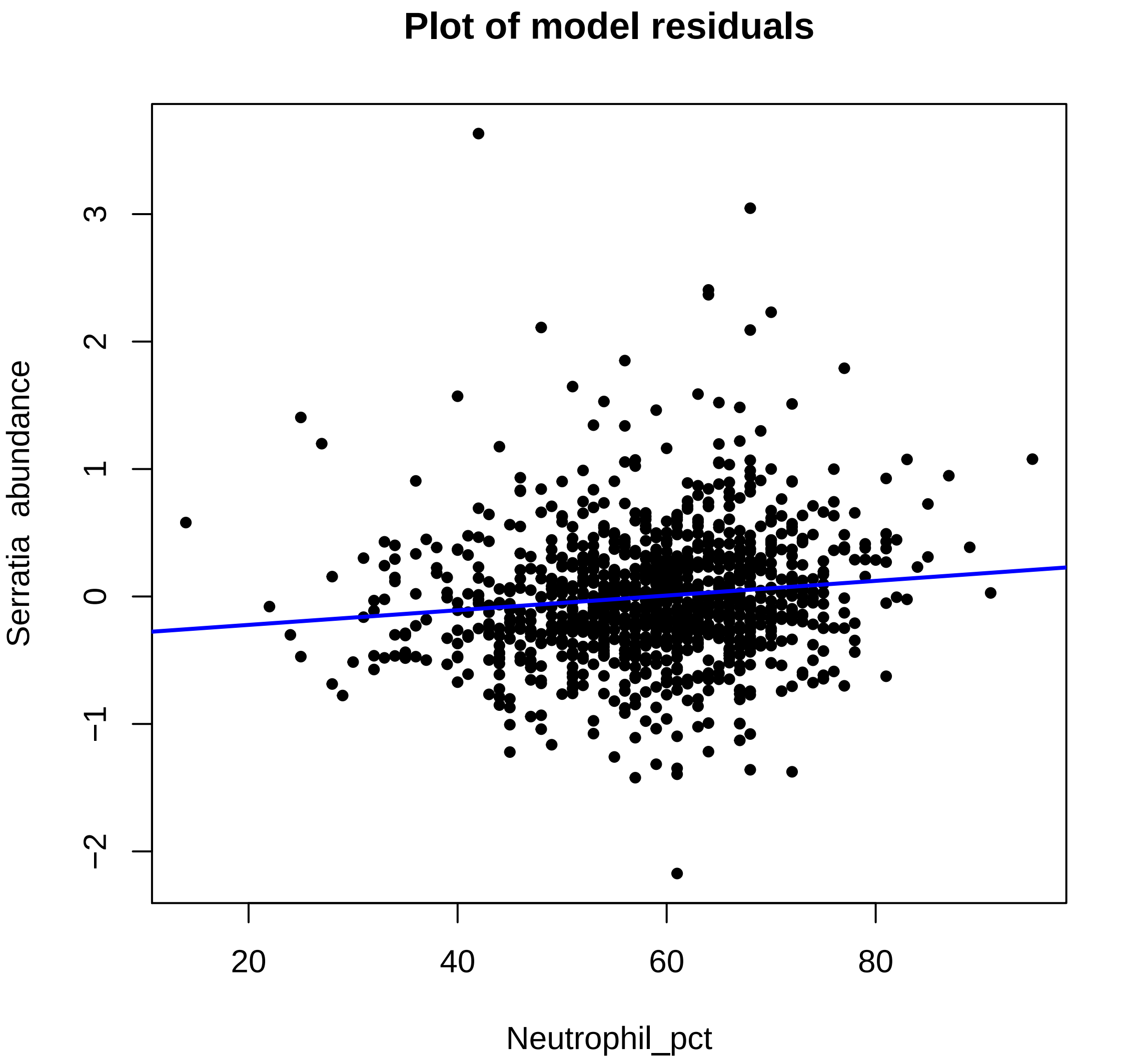

Plot of model residuals
Serratia abundance
Neutrophil_pct
3
2
1
0
−1
−2
20
40
60
80

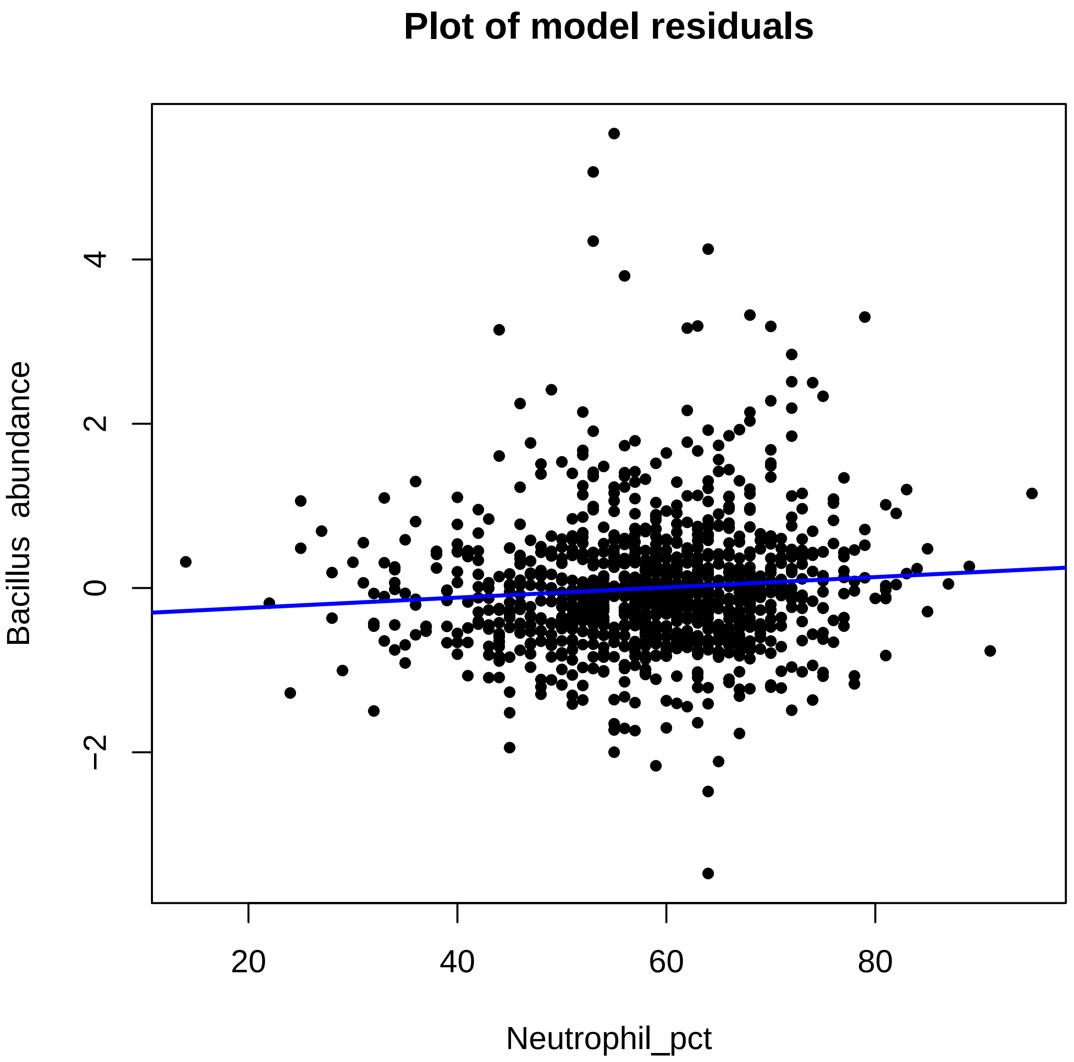
Plot of model residuals
Bacillus abundance
4
2
0
−2
20
40
60
80
Neutrophil_pct

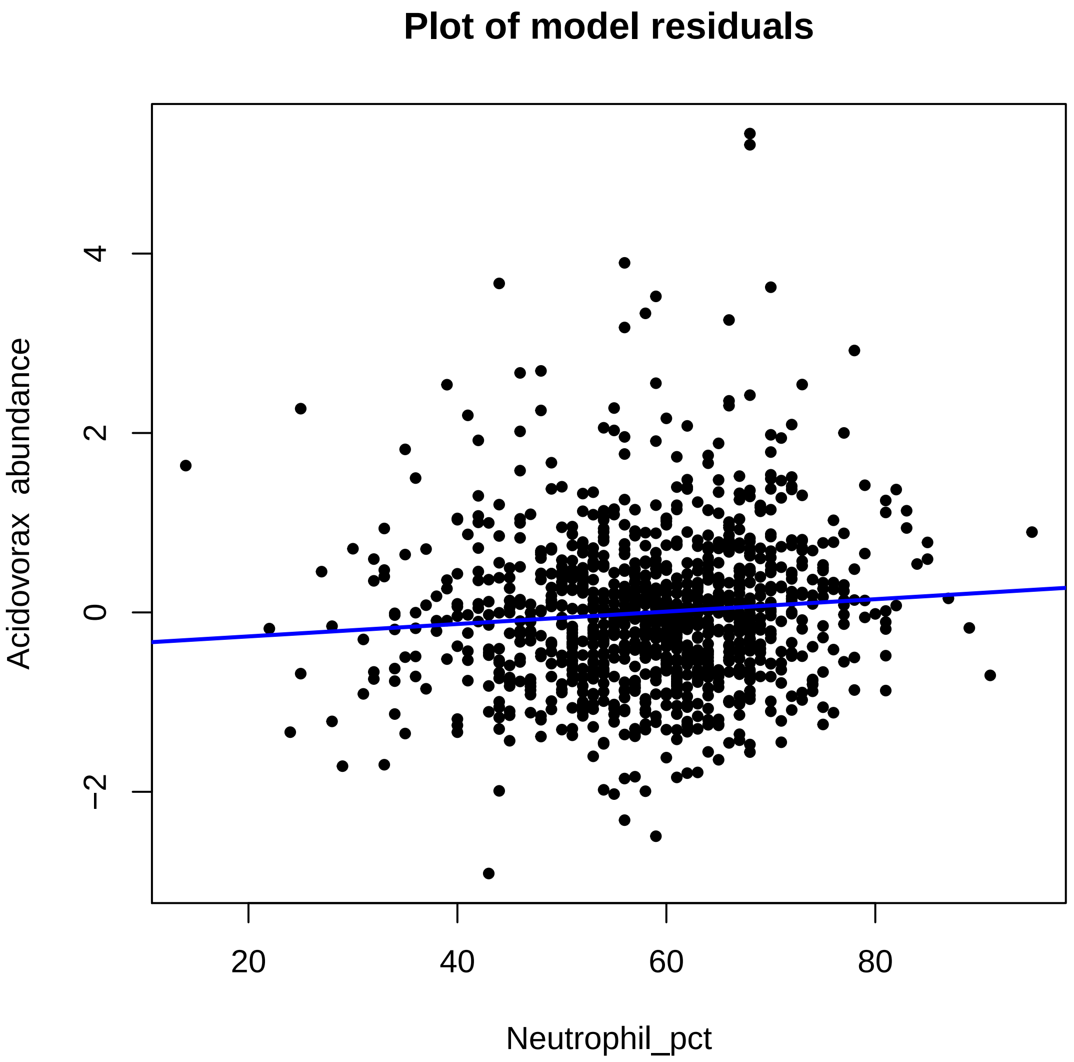

Plot of model residuals
Acidovorax abundance
Neutrophil_pct
−2
0
2
4
20
40
60
80

Plot of model residuals

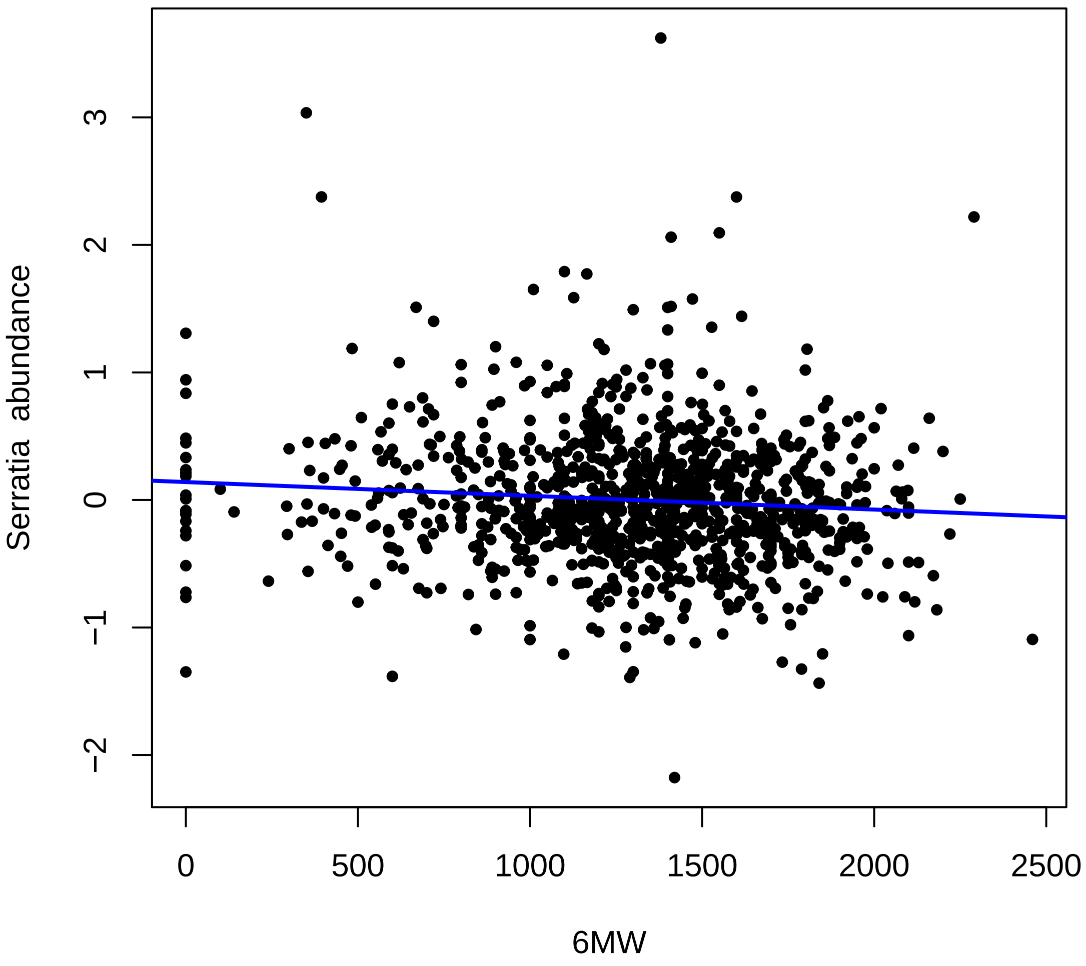

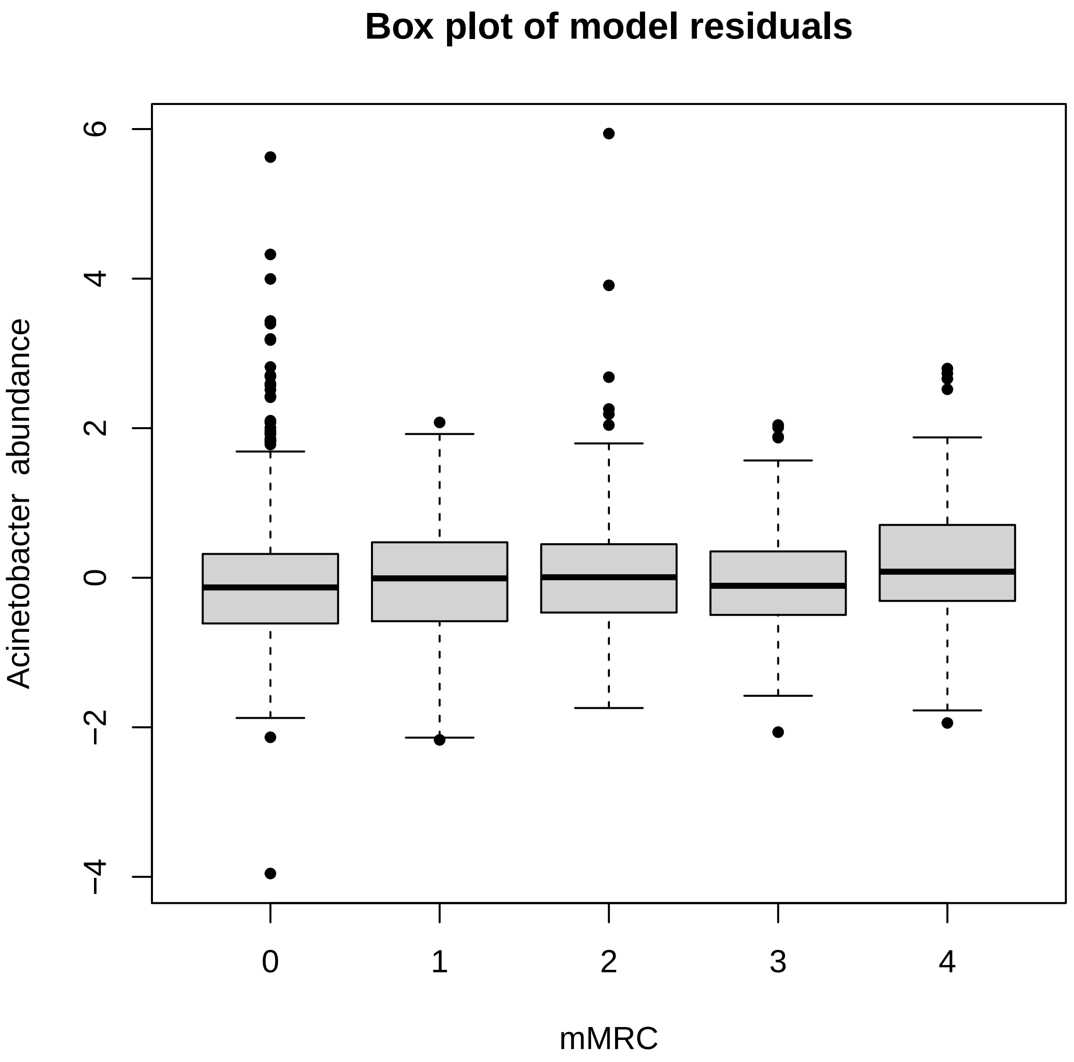
Box plot of model residuals
Acinetobacter abundance
6
4
2
0
−2
−4
0
1
2
3
4
mMRC

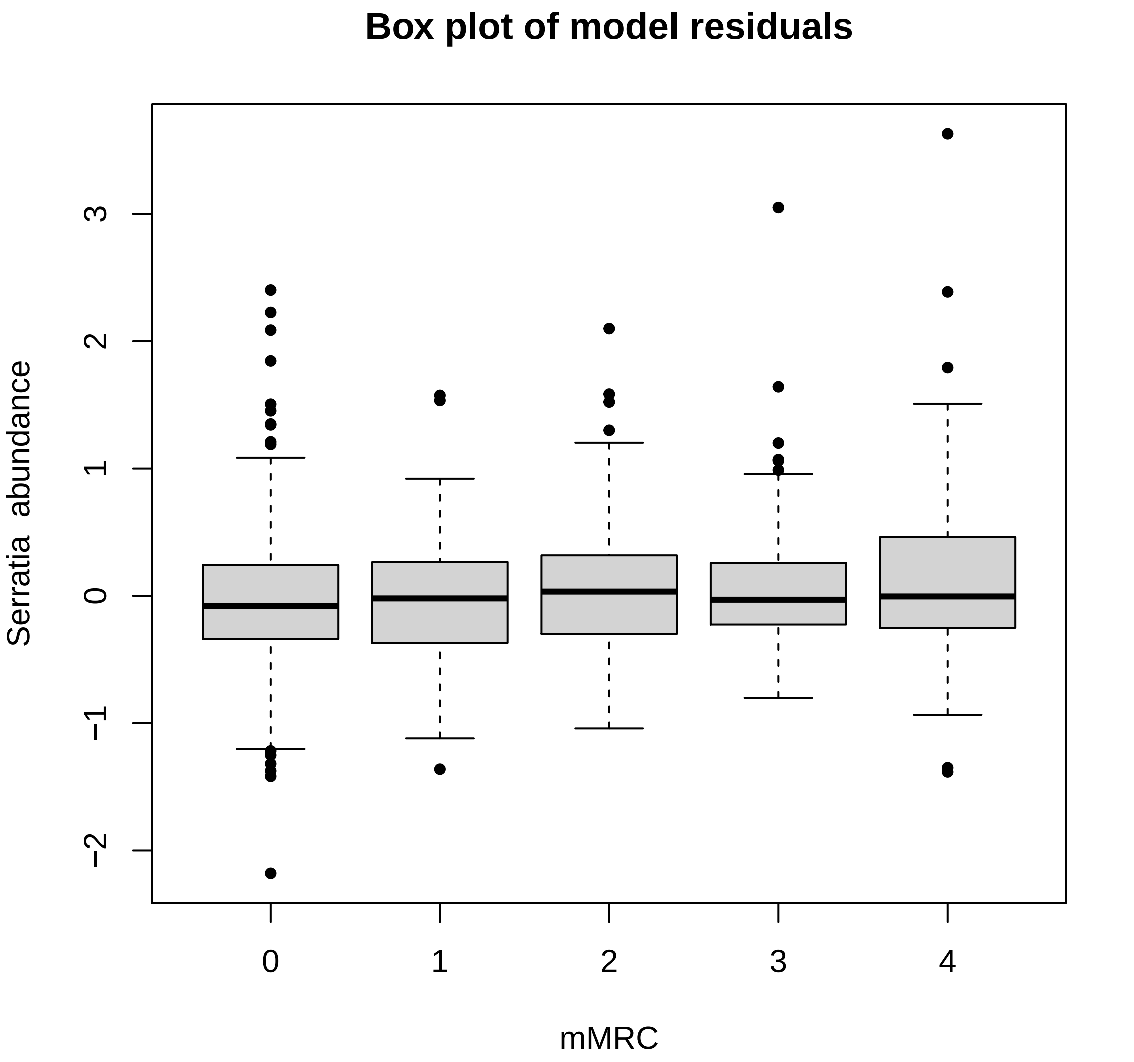
Box plot of model residuals
Serratia abundance
mMRC
3
2
1
0
−1
−2
0
1
2
3
4

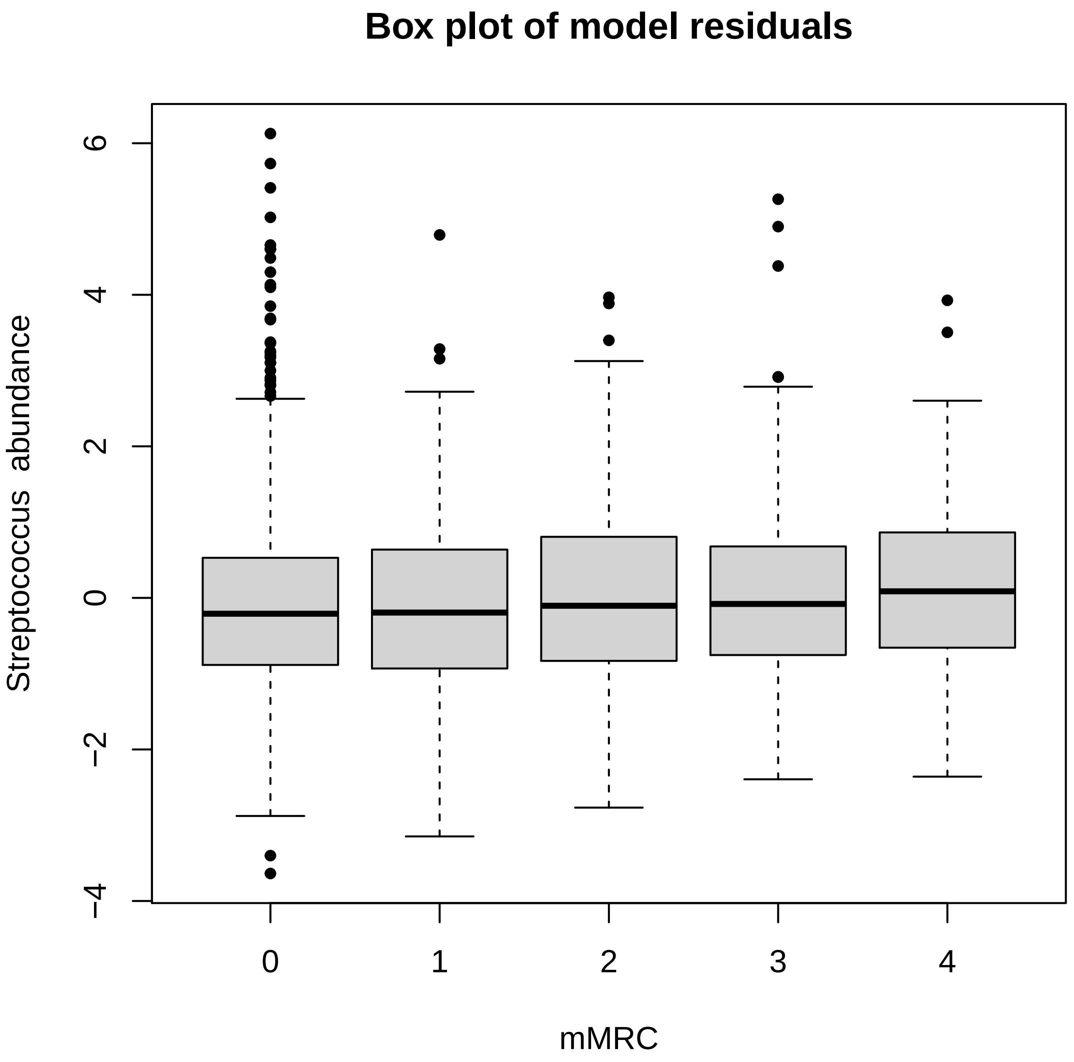

Box plot of model residuals
Streptococcus abundance
mMRC
6
4
2
0
−2
−4
0
1
2
3
4

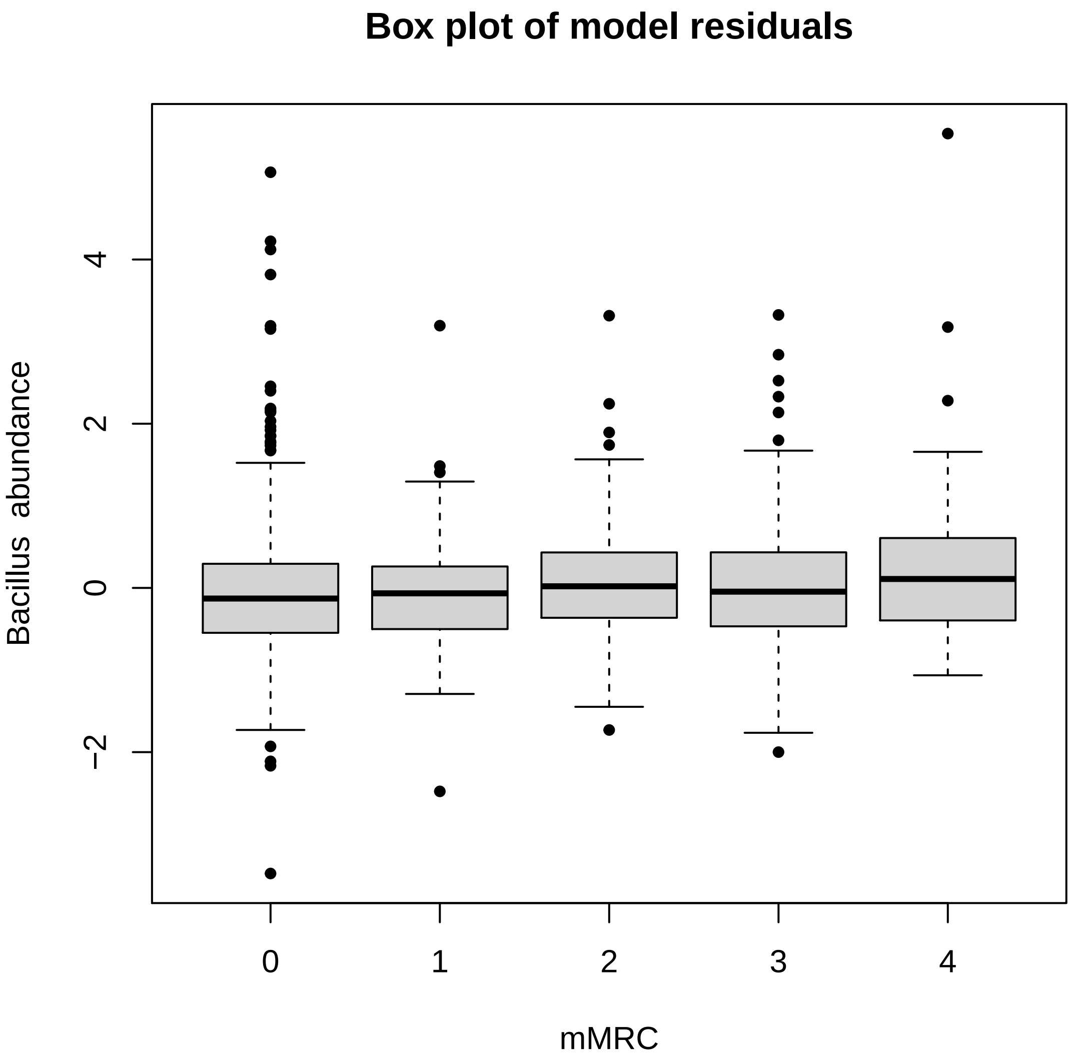
Box plot of model residuals
Bacillus abundance
−2
0
2
4
0
1
2
3
4
mMRC

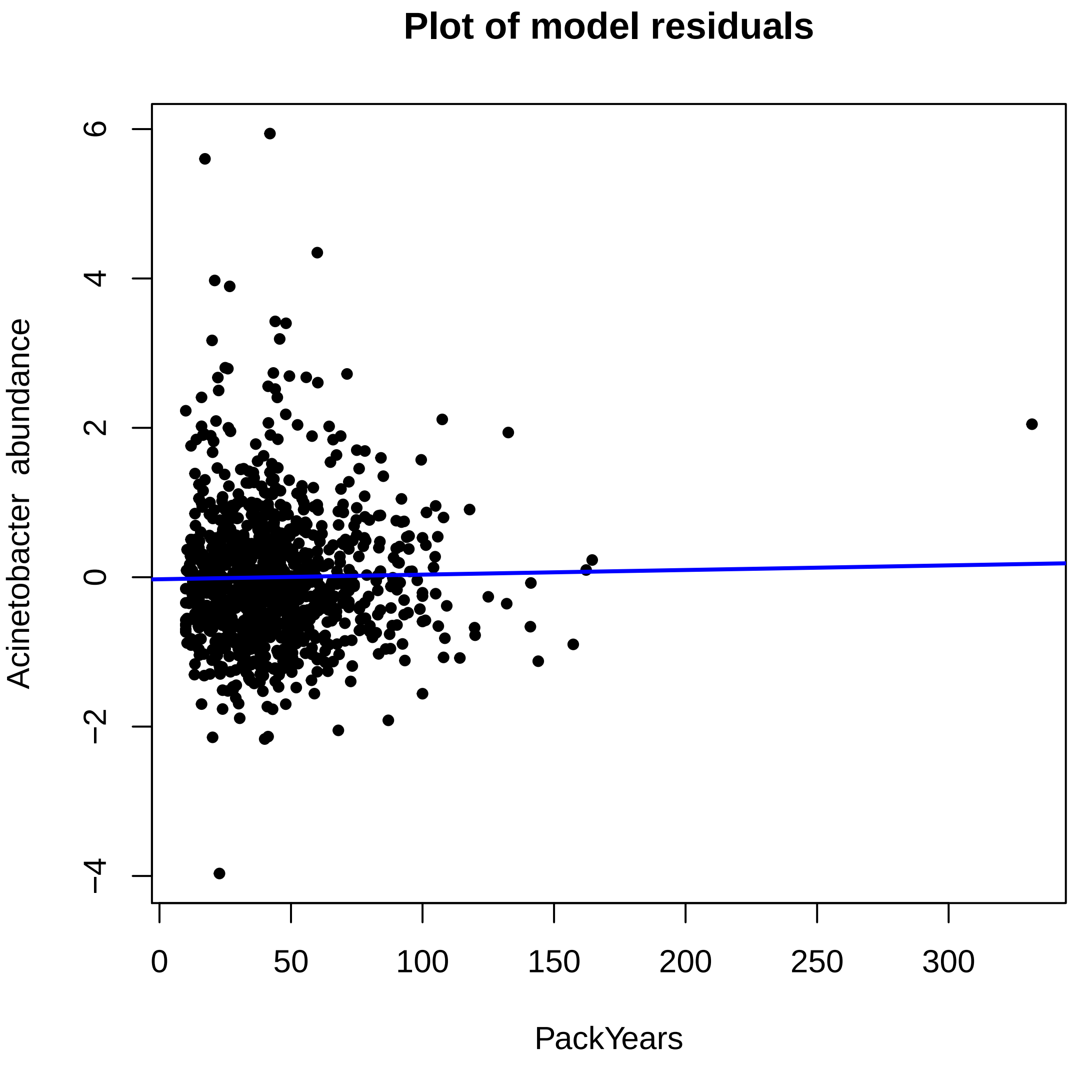

Plot of model residuals
Acinetobacter abundance
PackYears
6
4
2
0
−2
−4
0
50
100
150
200
250
300

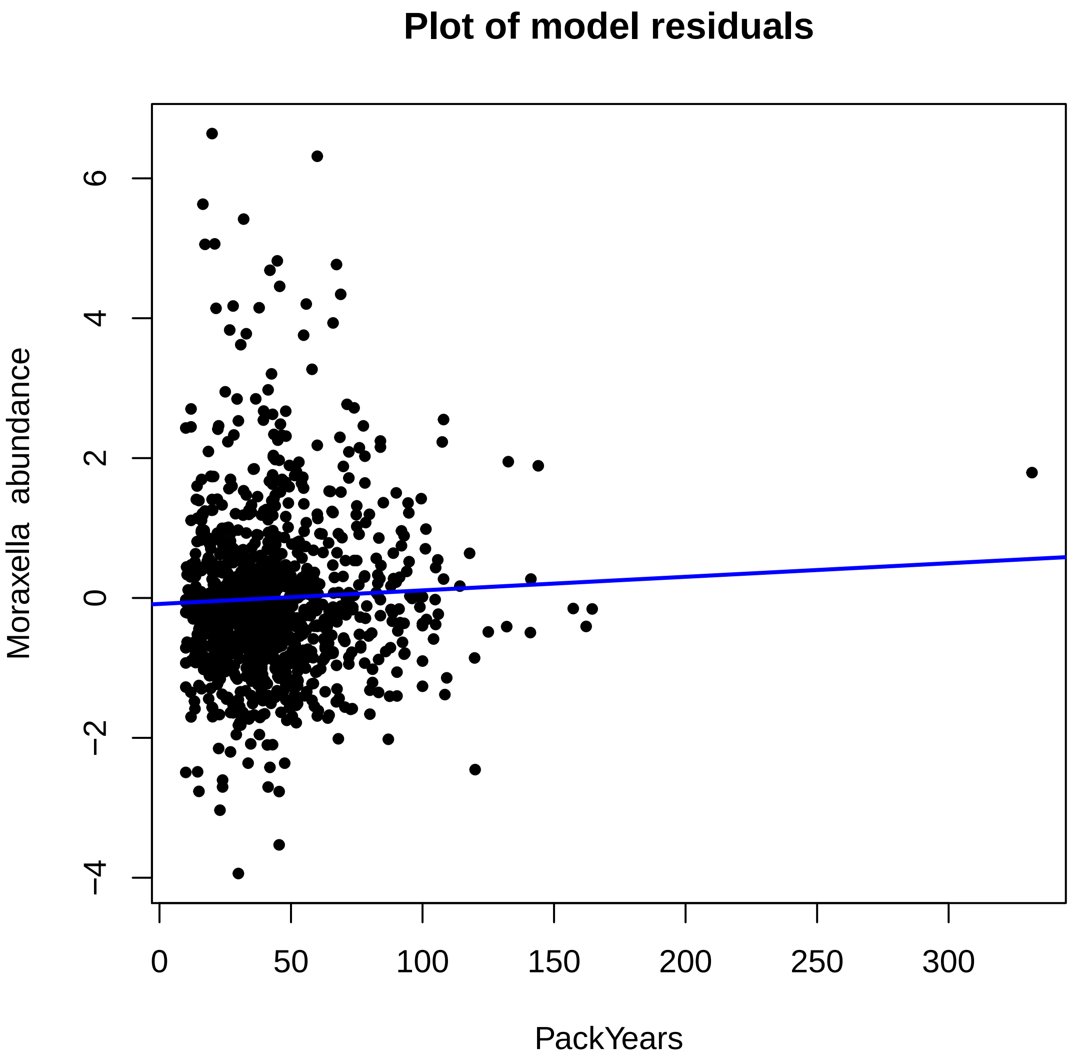

Plot of model residuals
Moraxella abundance
PackYears
6
4
2
0
−2
−4
0
50
100
150
200
250
300

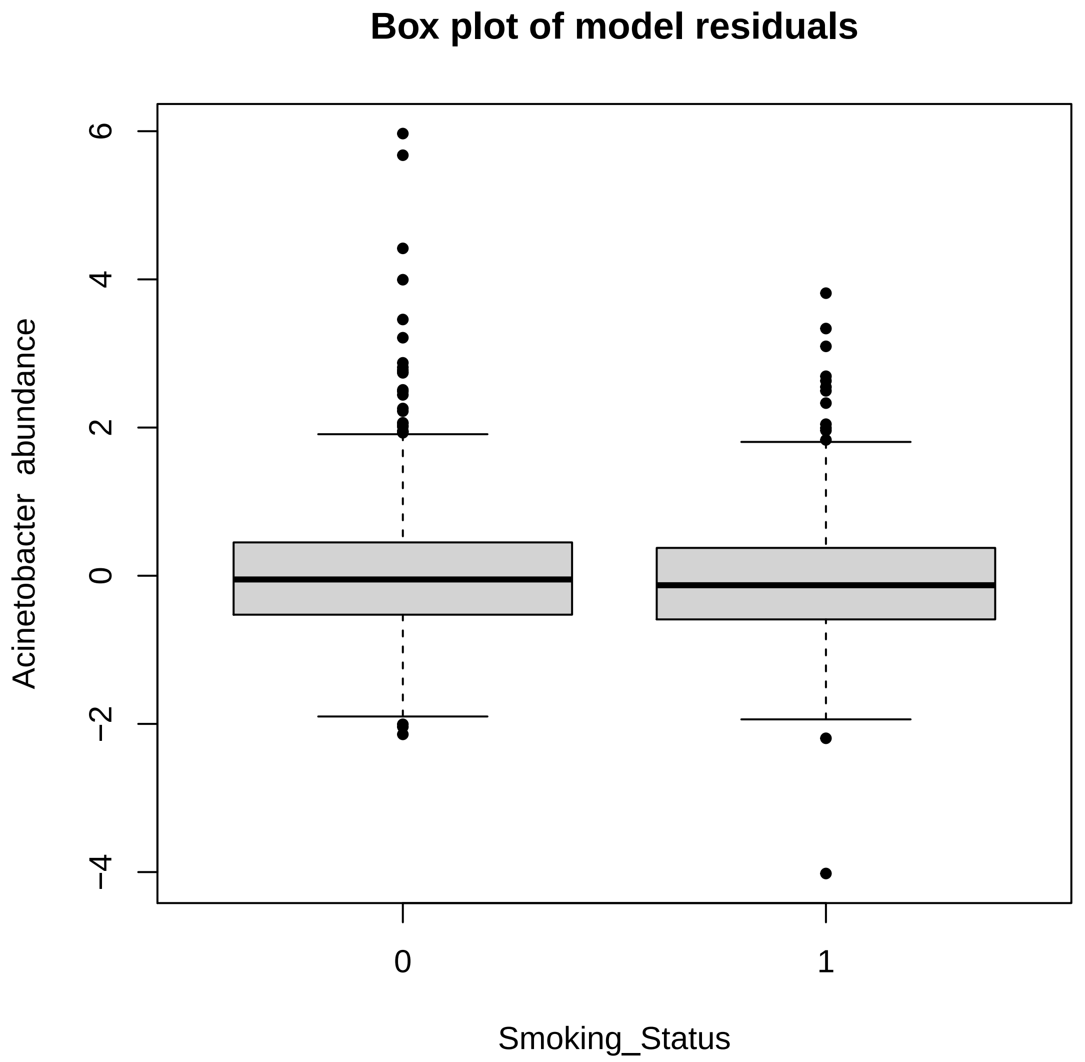

Box plot of model residuals
Acinetobacter abundance
6
4
2
0
−2
−4
0
1
Smoking_Status

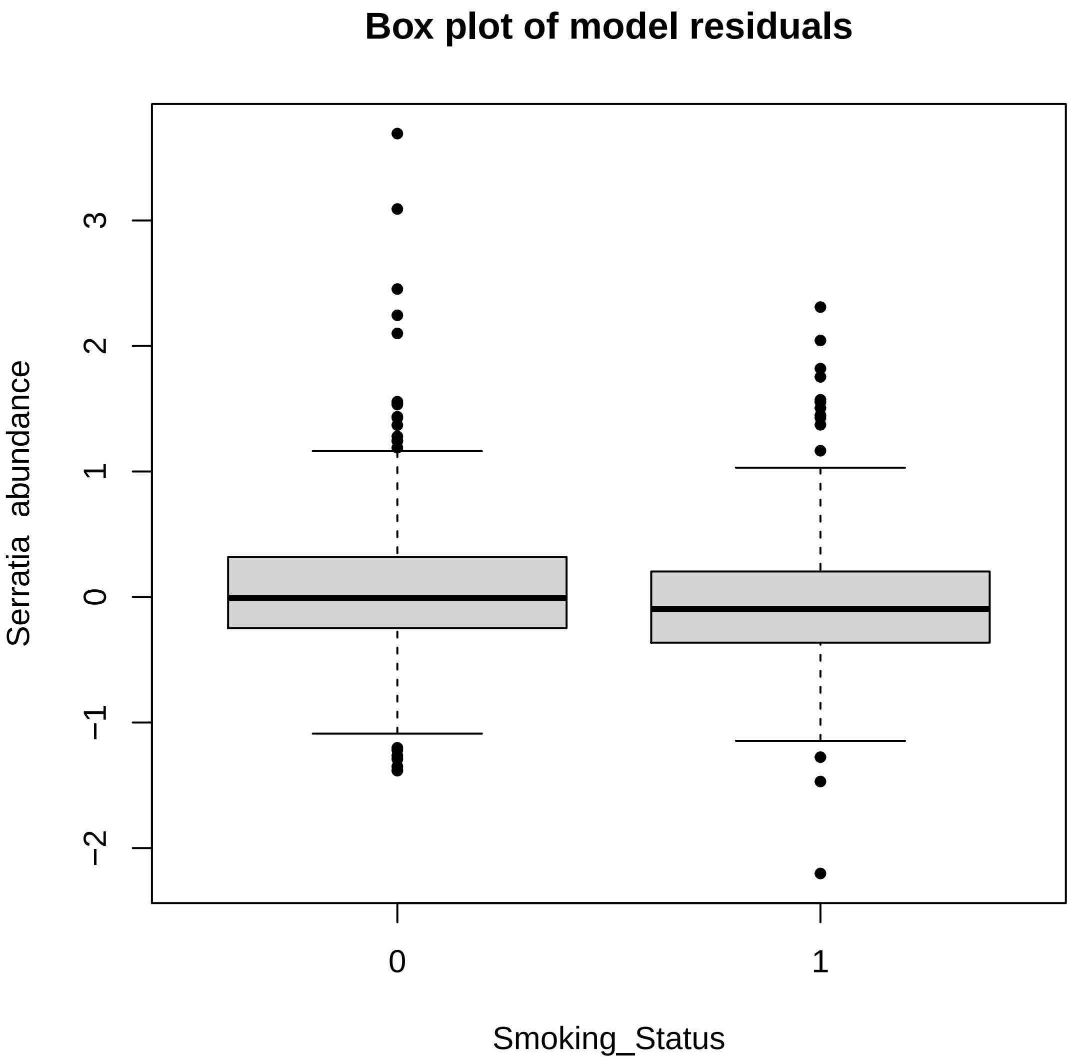
Box plot of model residuals
Serratia abundance
3
2
1
0
−1
−2
0
1
Smoking_Status

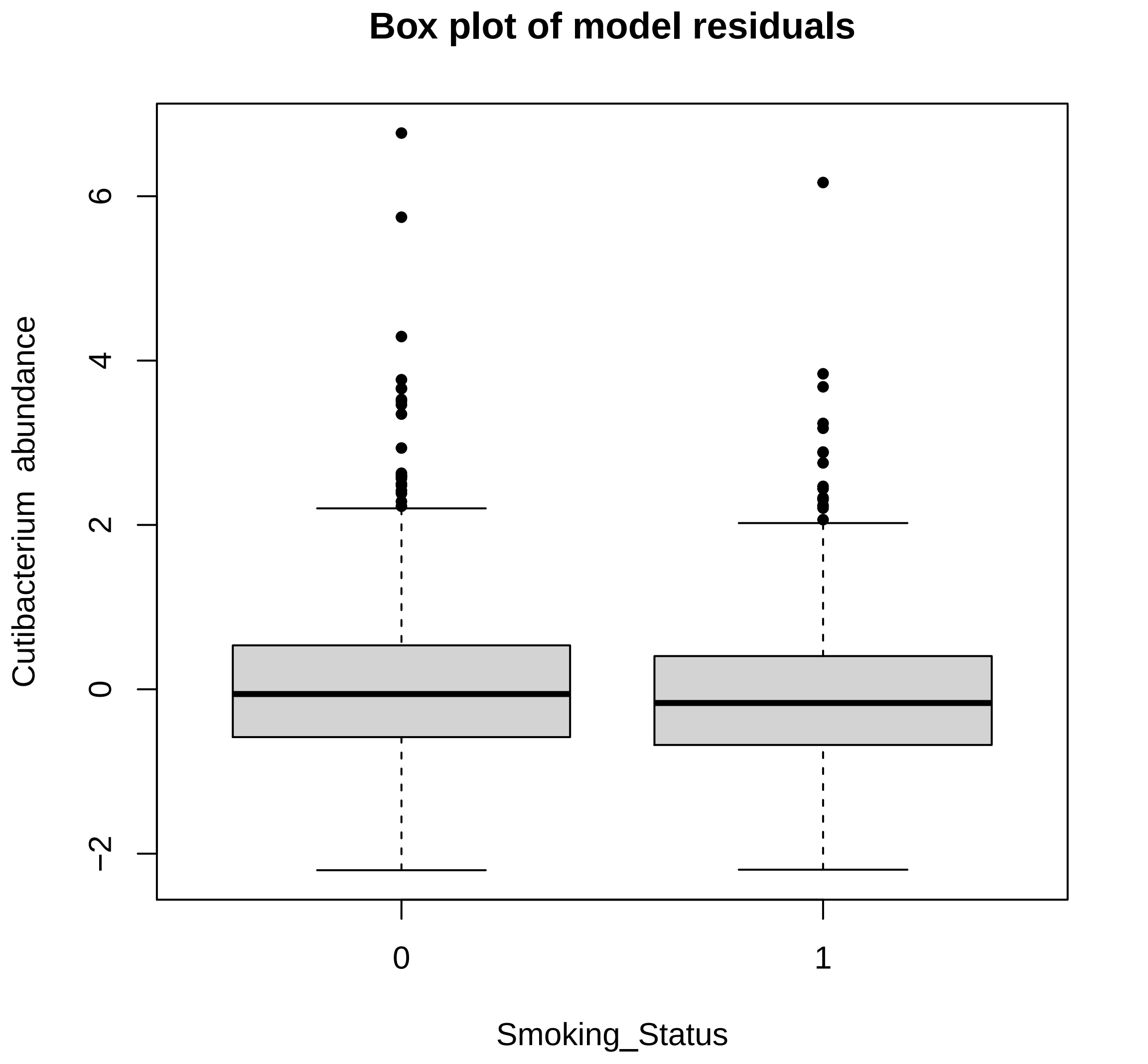

Box plot of model residuals
Cutibacterium abundance
6
4
2
0
−2
0
1
Smoking_Status

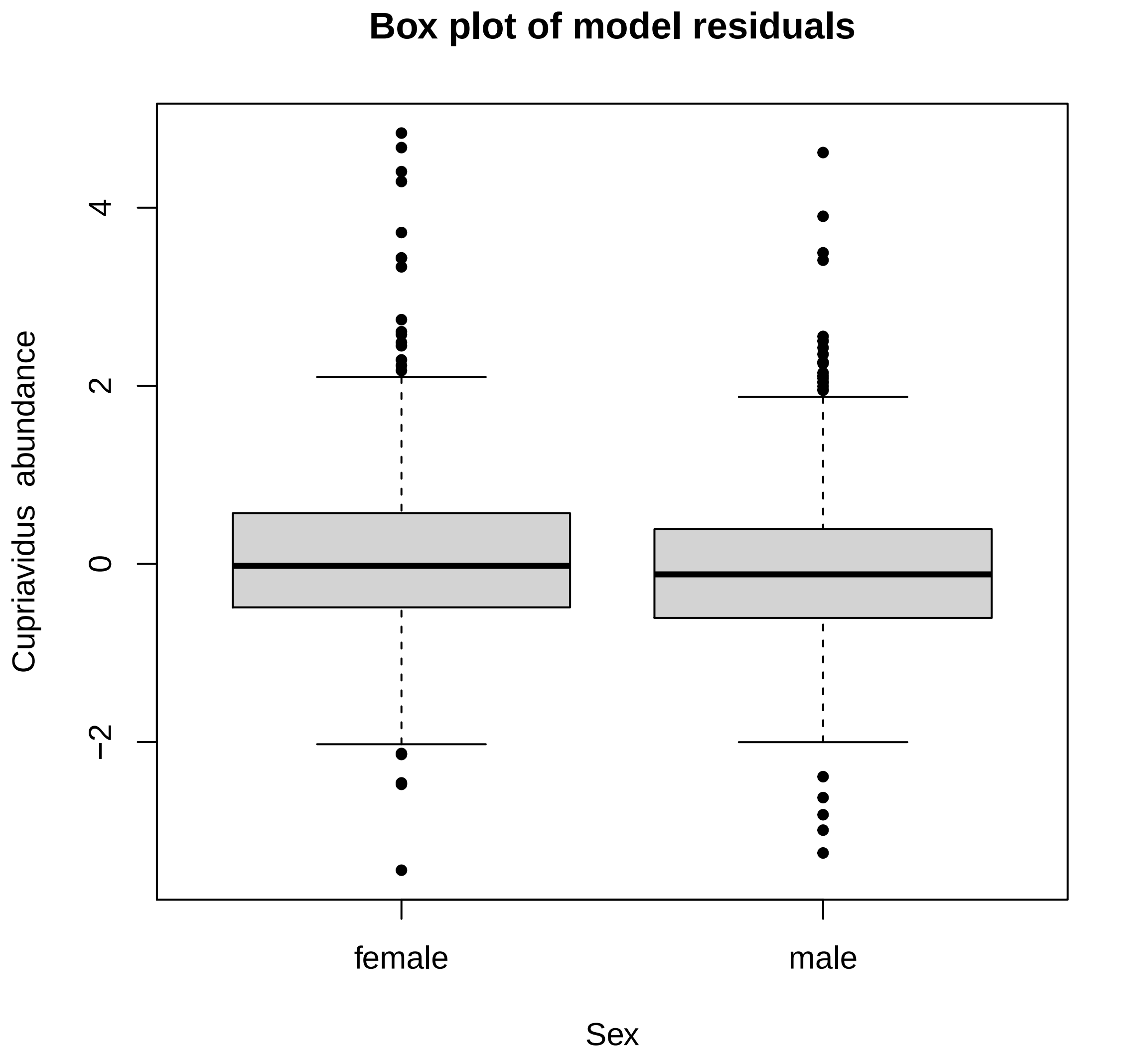

Box plot of model residuals
Cupriavidus abundance
4
2
0
−2
female
male
Sex

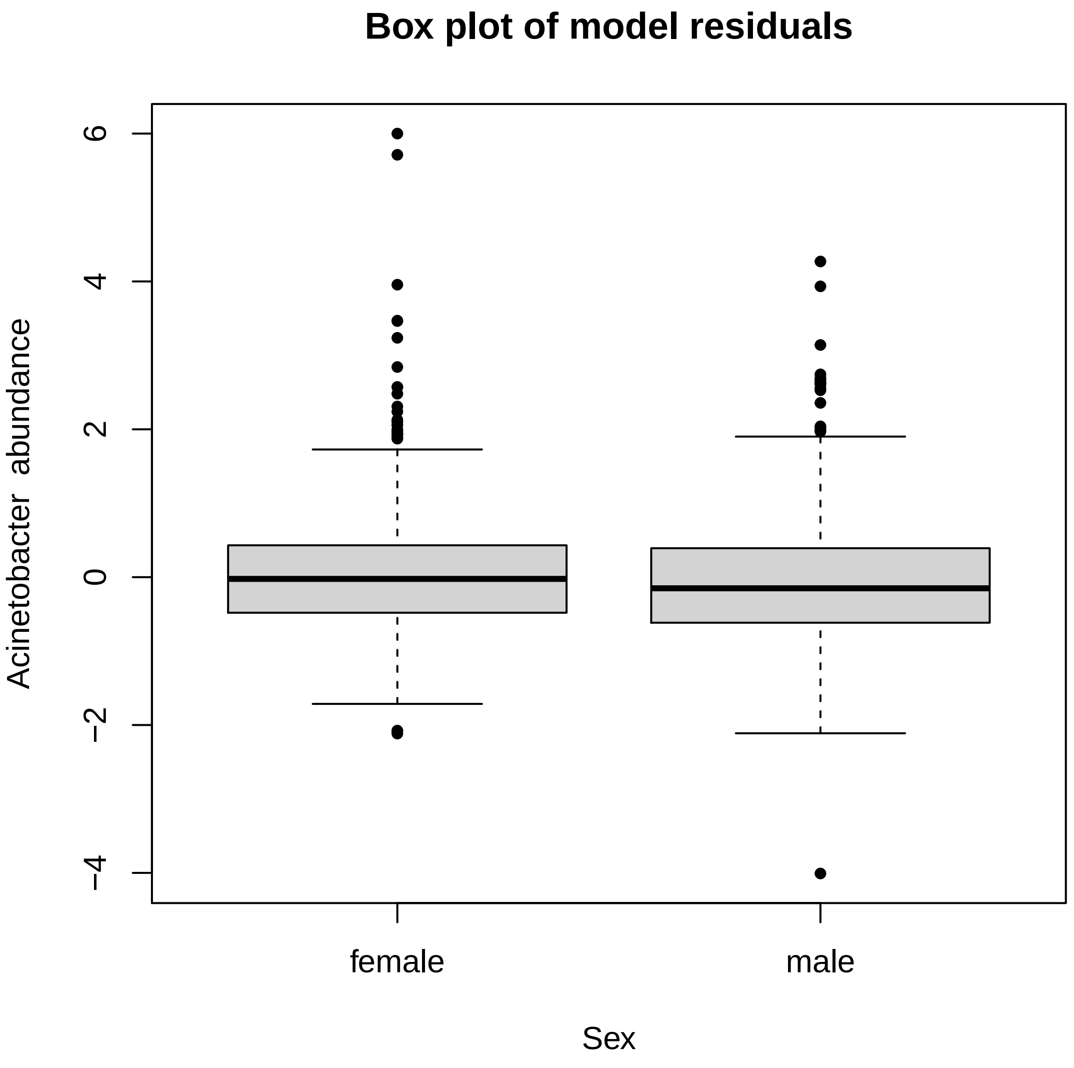
Box plot of model residuals
Acinetobacter abundance
6
4
2
0
−2
−4
female
male
Sex

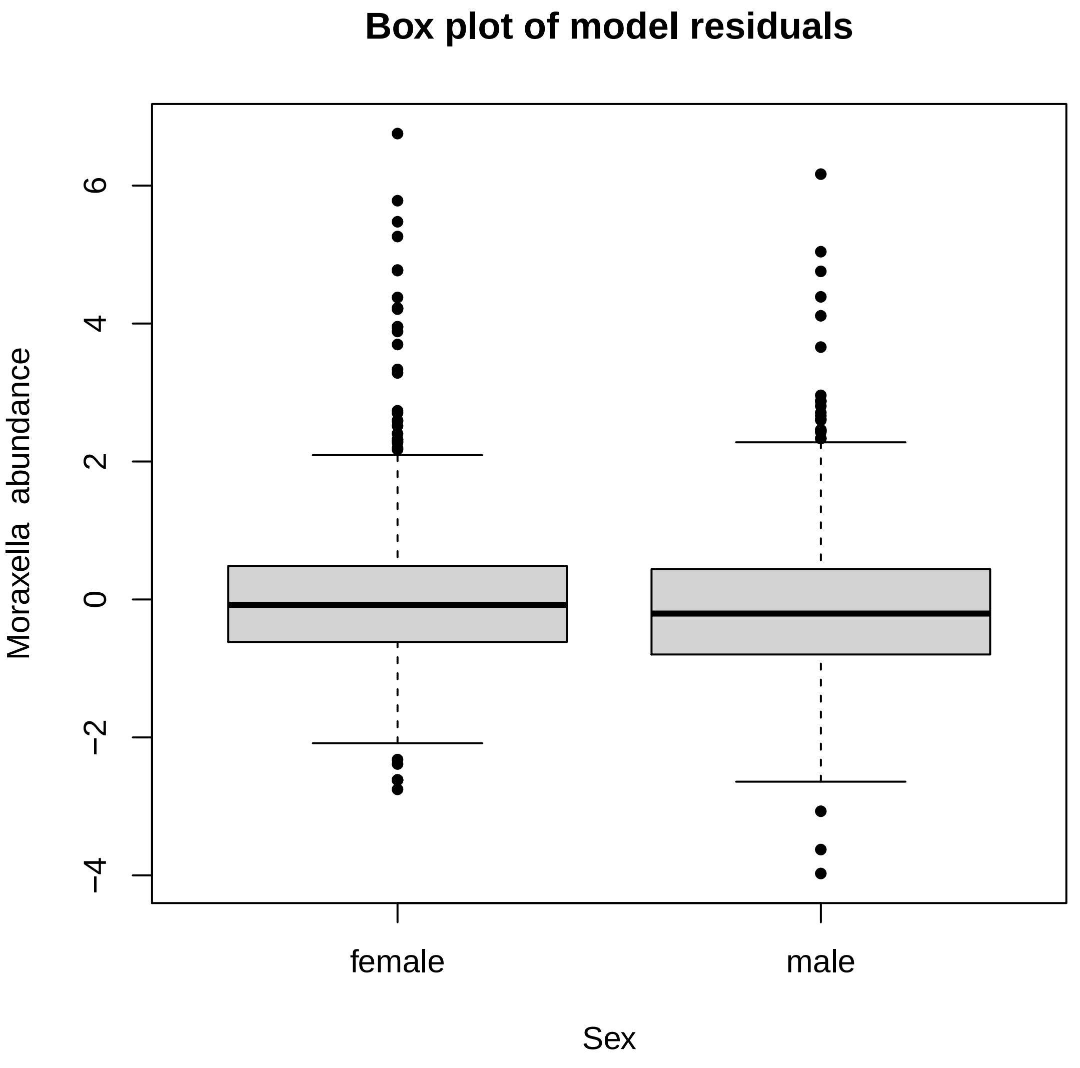

Box plot of model residuals
Moraxella abundance
6
4
2
0
−2
−4
female
male
Sex

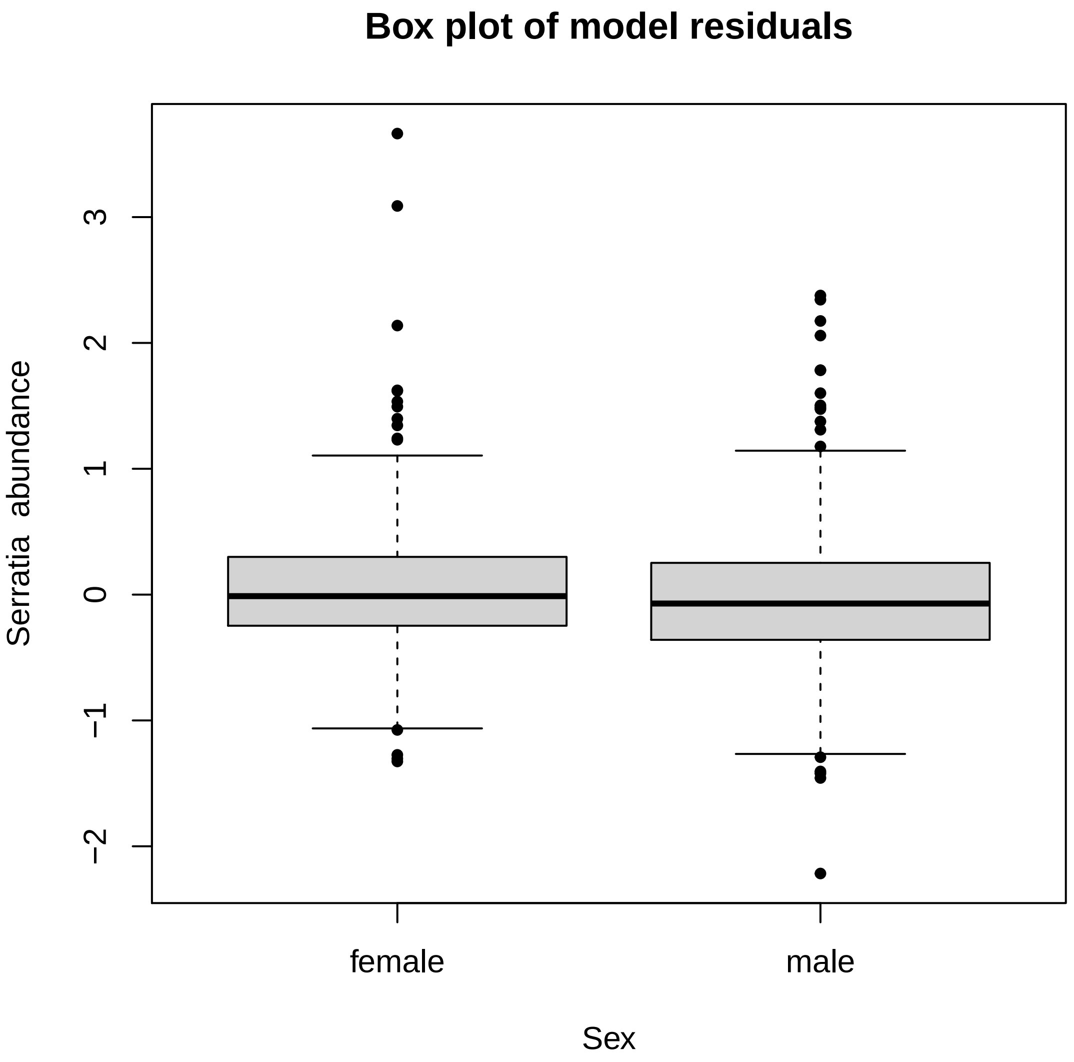
Box plot of model residuals
Serratia abundance
3
2
1
0
−1
−2
female
male
Sex

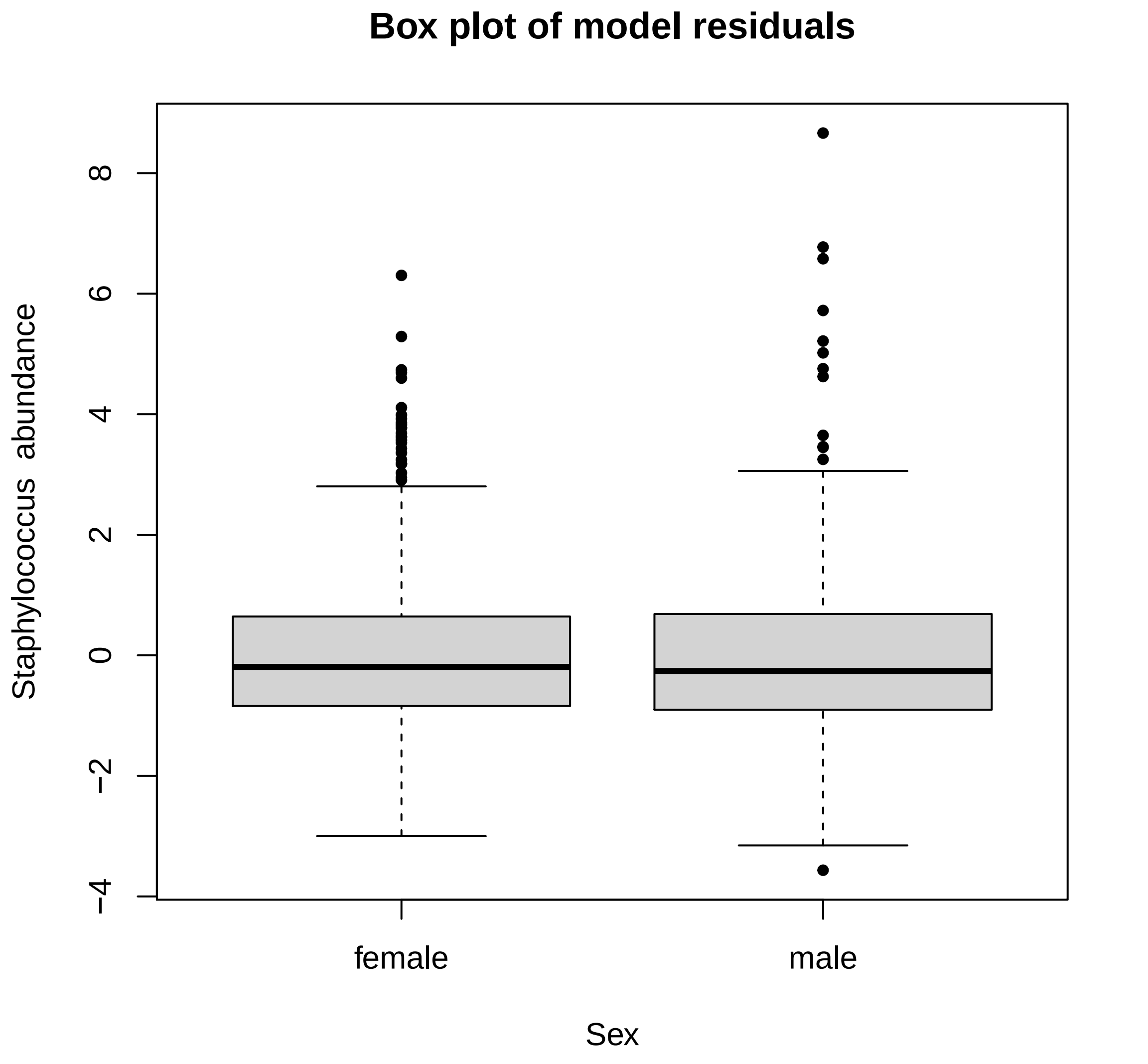
Box plot of model residuals
Staphylococcus abundance
8
6
4
2
0
−2
−4
female
male
Sex

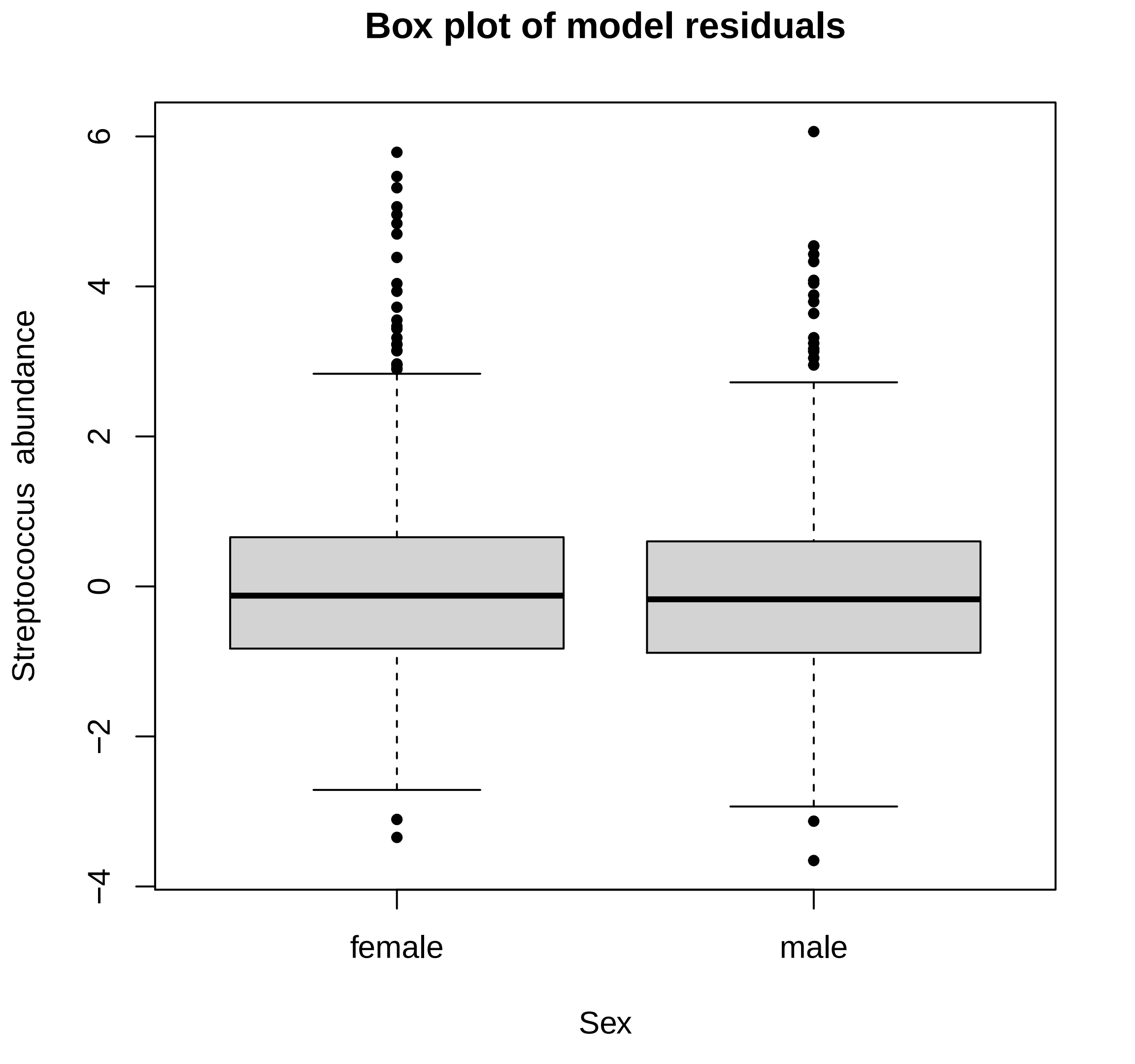

Box plot of model residuals
Streptococcus abundance
6
4
2
0
−2
−4
female
male
Sex

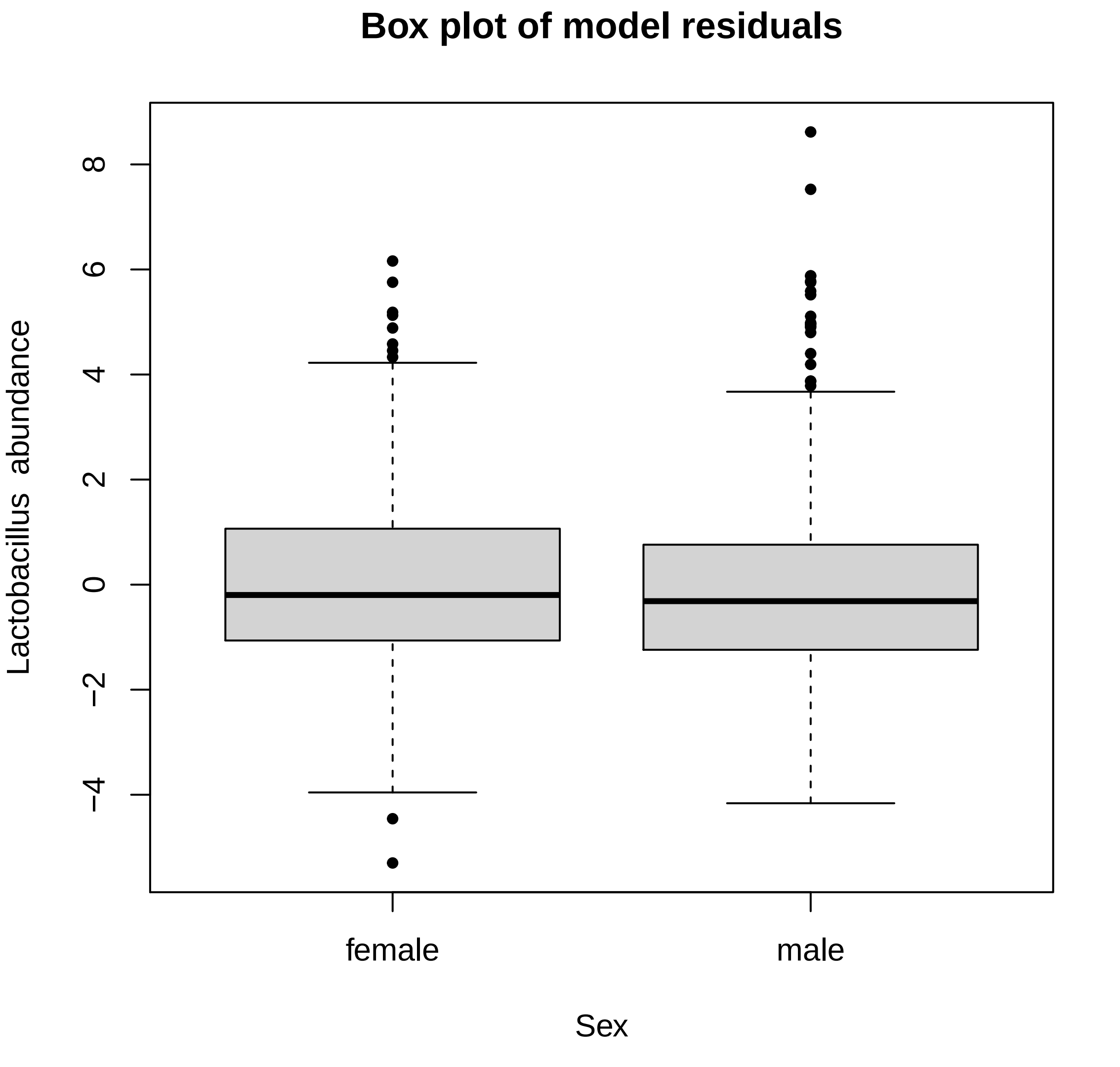
Box plot of model residuals
Lactobacillus abundance
8
6
4
2
0
−2
−4
female
male
Sex

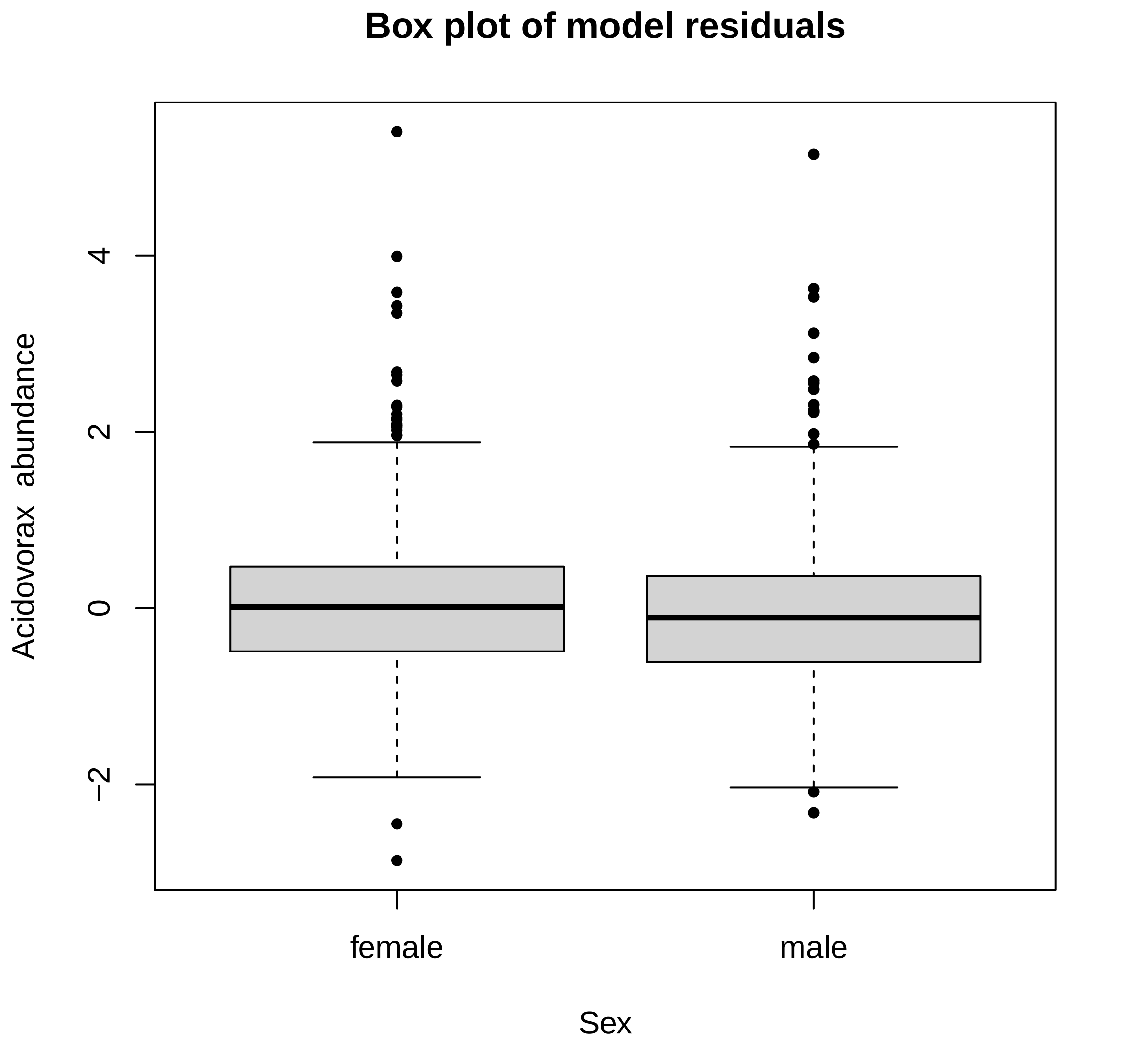
Box plot of model residuals
Acidovorax abundance
4
2
0
−2
female
male
Sex

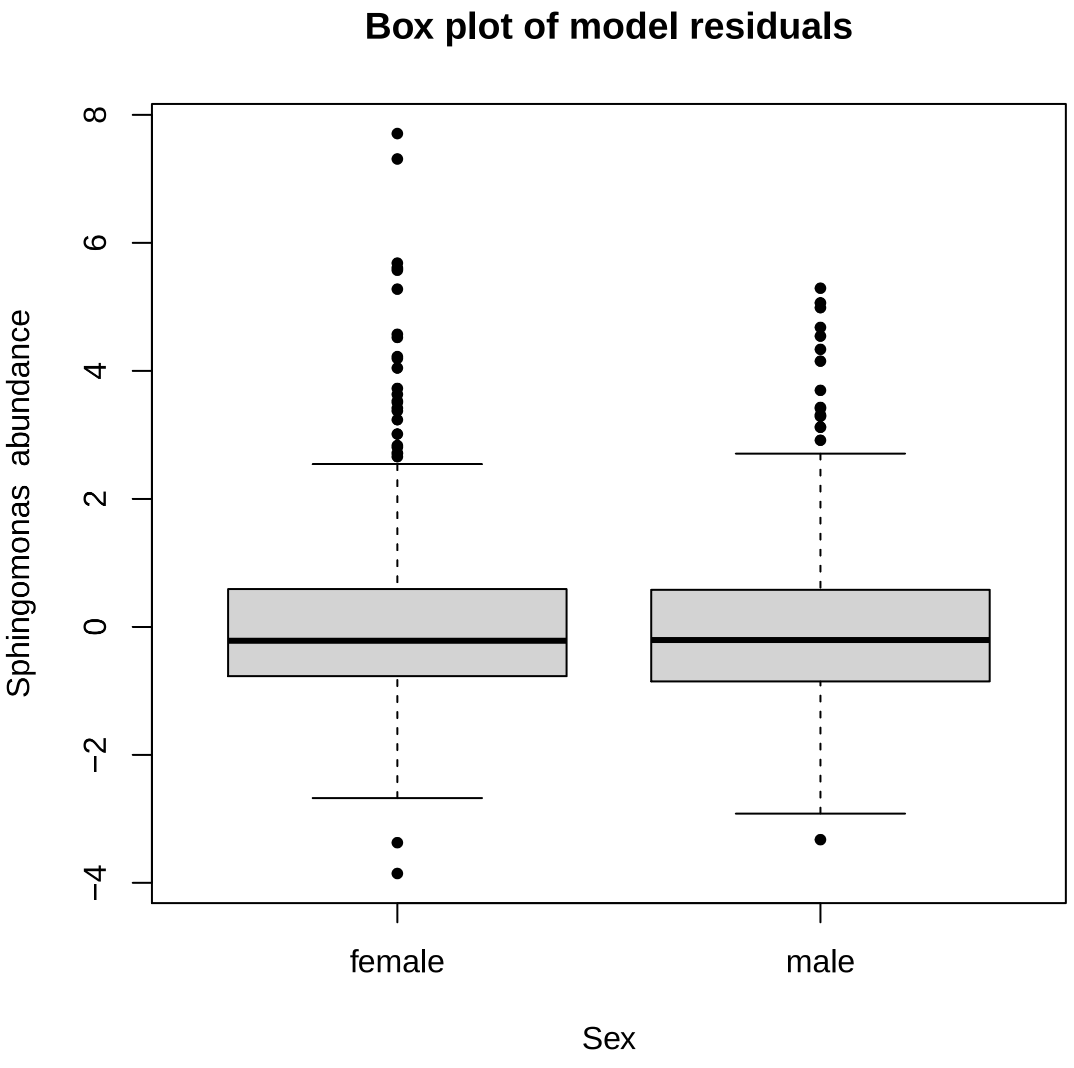

Box plot of model residuals
8
6
4
2
0
−2
−4
Sphingomonas abundance
female
male
Sex

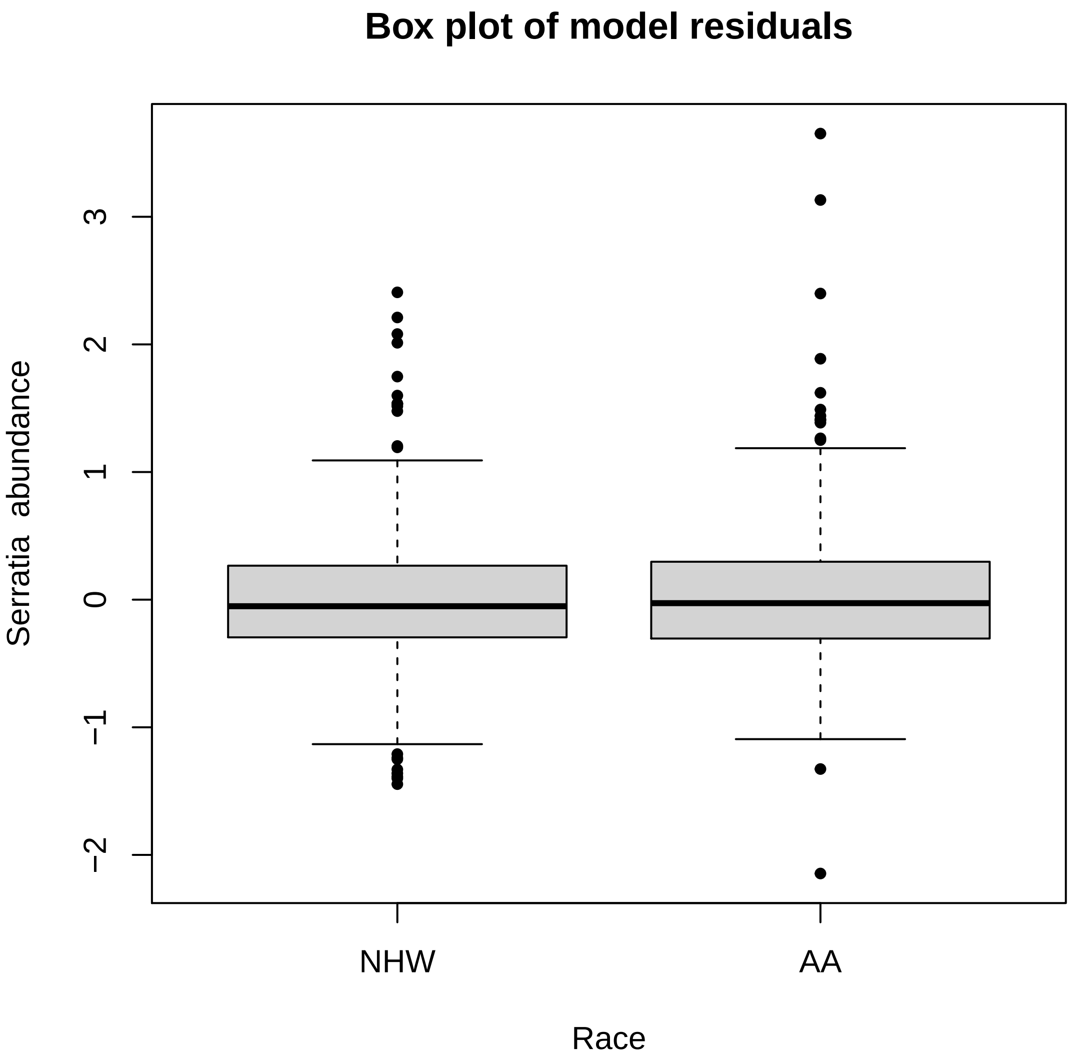

Box plot of model residuals
Serratia abundance
3
2
1
0
−1
−2
NHW
AA
Race

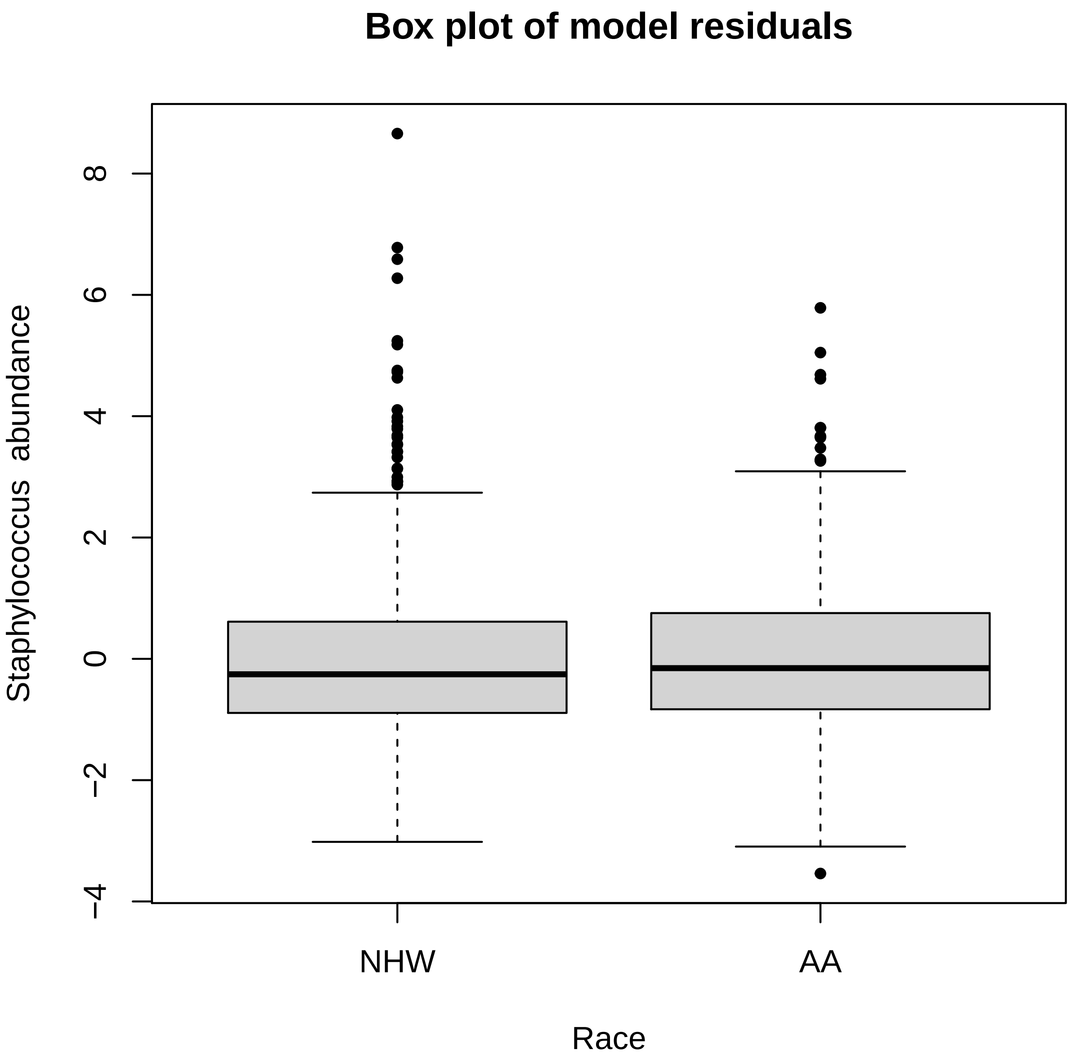

Box plot of model residuals
Staphylococcus abundance
8
6
4
2
0
−2
−4
NHW
AA
Race

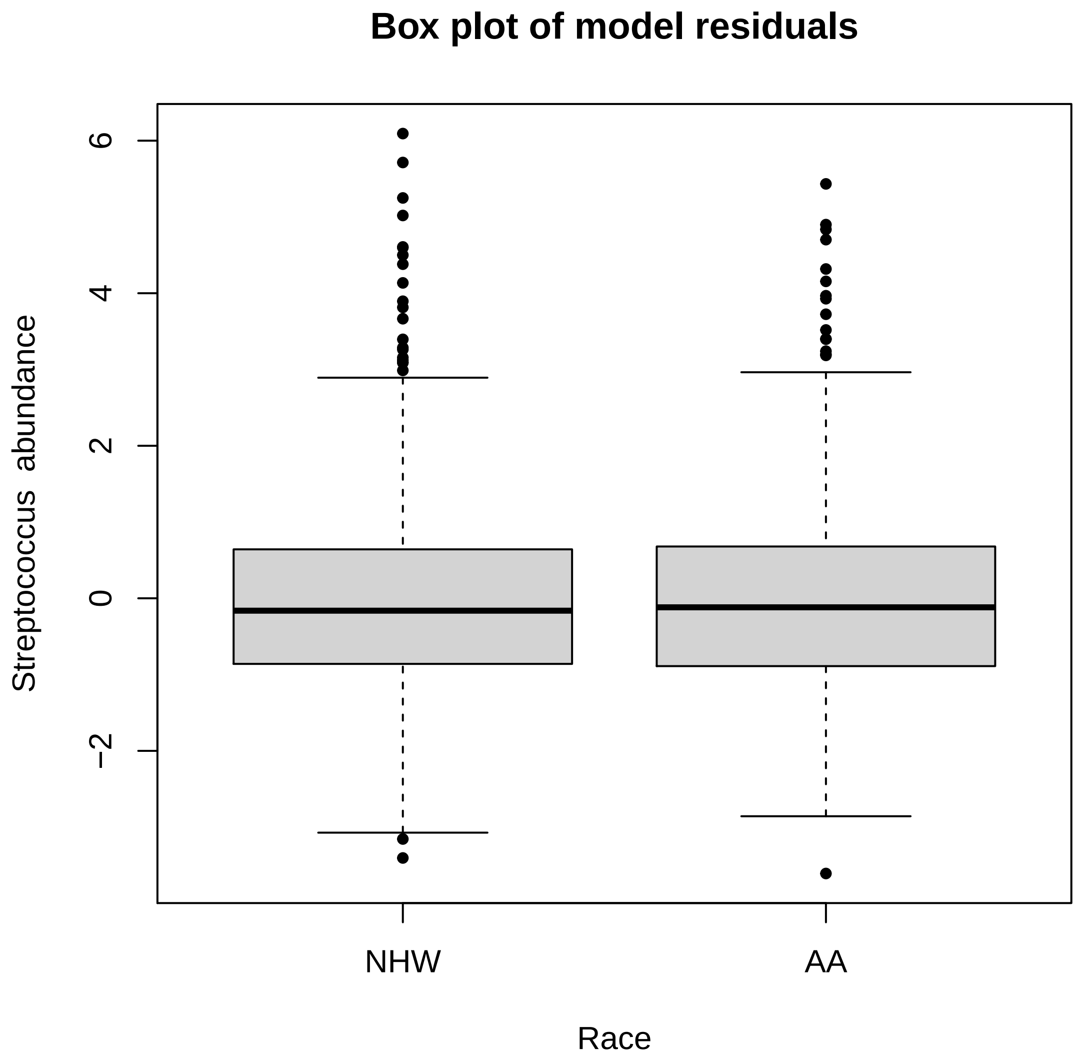

Box plot of model residuals
Streptococcus abundance
6
4
2
0
−2
NHW
AA
Race

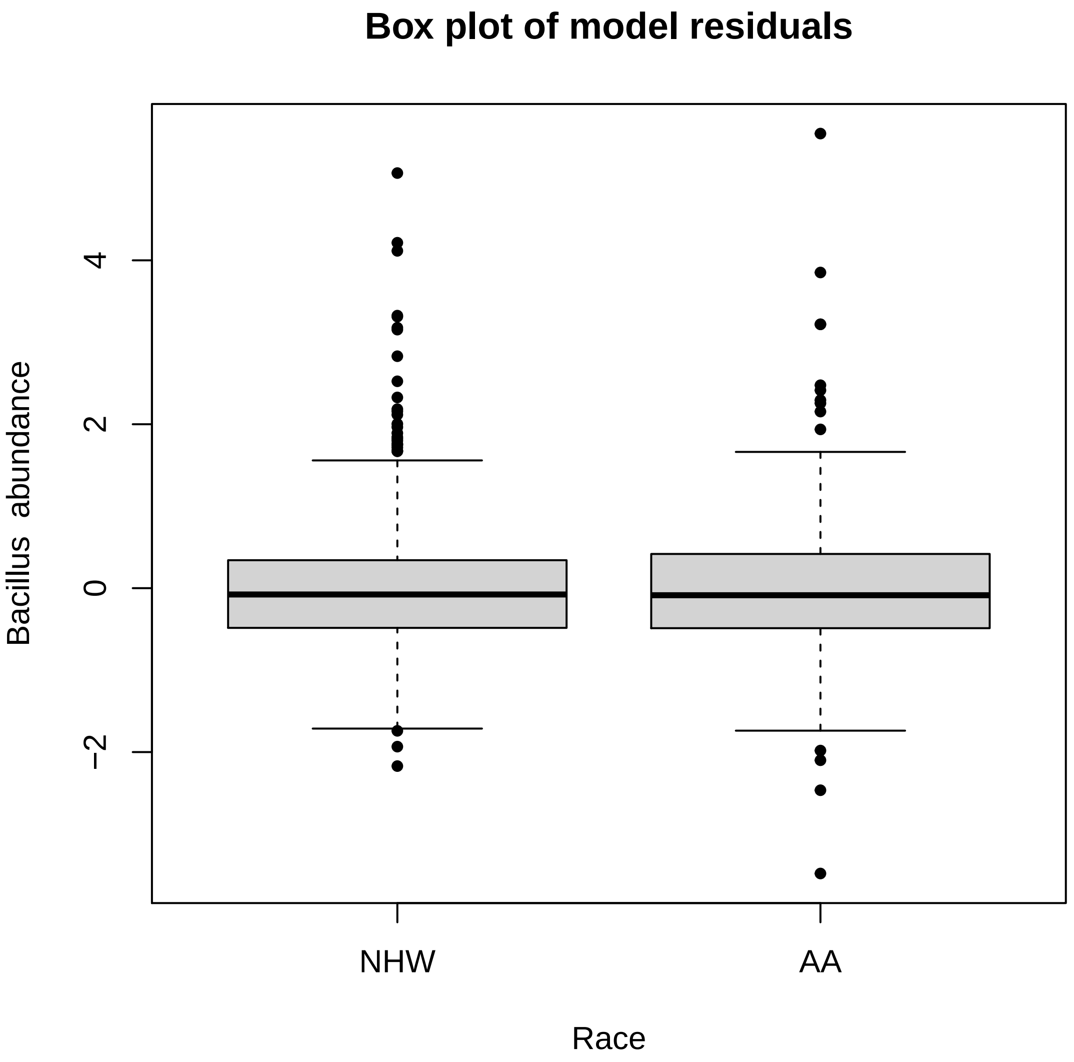
Box plot of model residuals
Bacillus abundance
4
2
0
−2
NHW
AA
Race

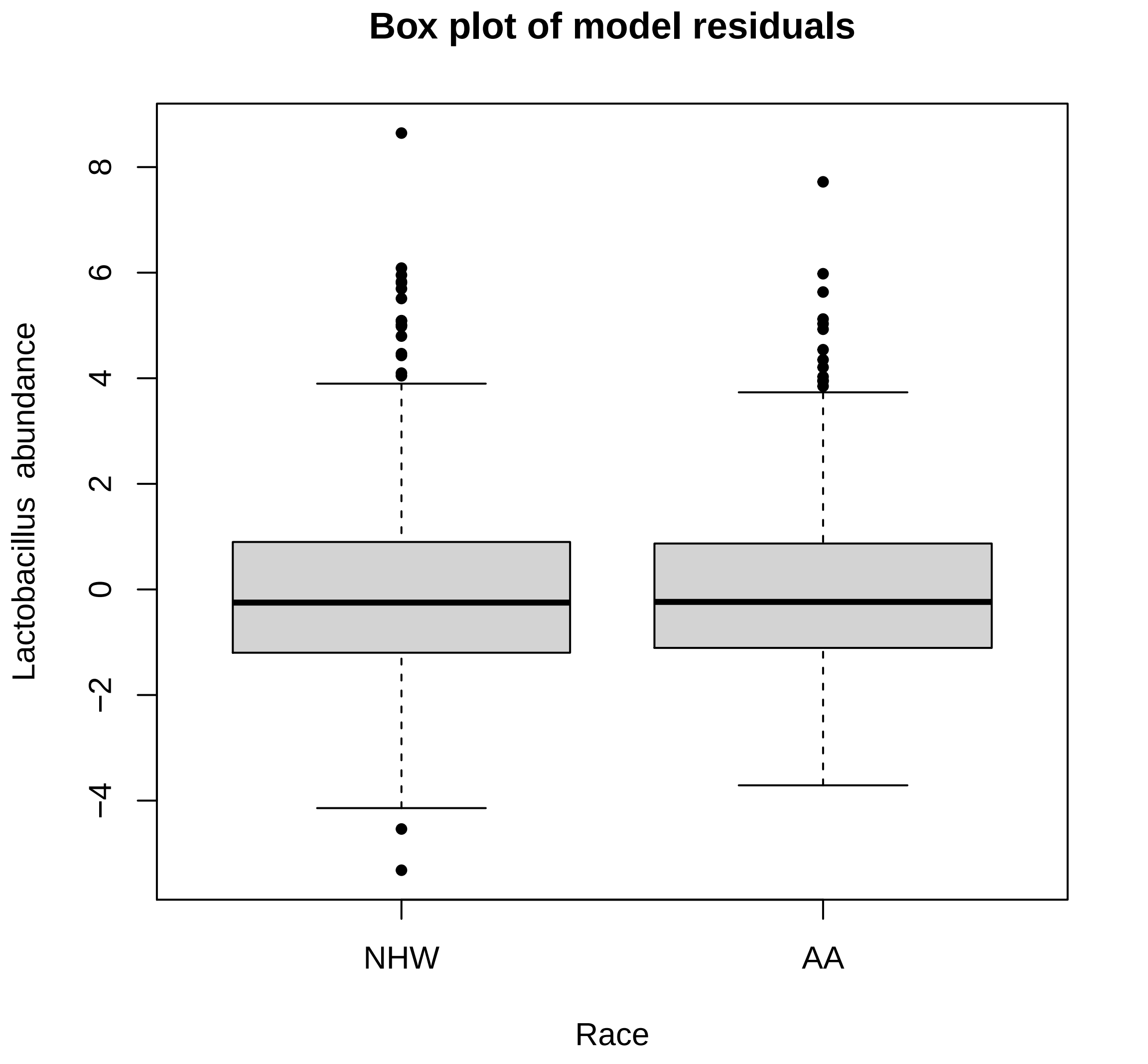
Box plot of model residuals
Lactobacillus abundance
8
6
4
2
0
−2
−4
NHW
AA
Race

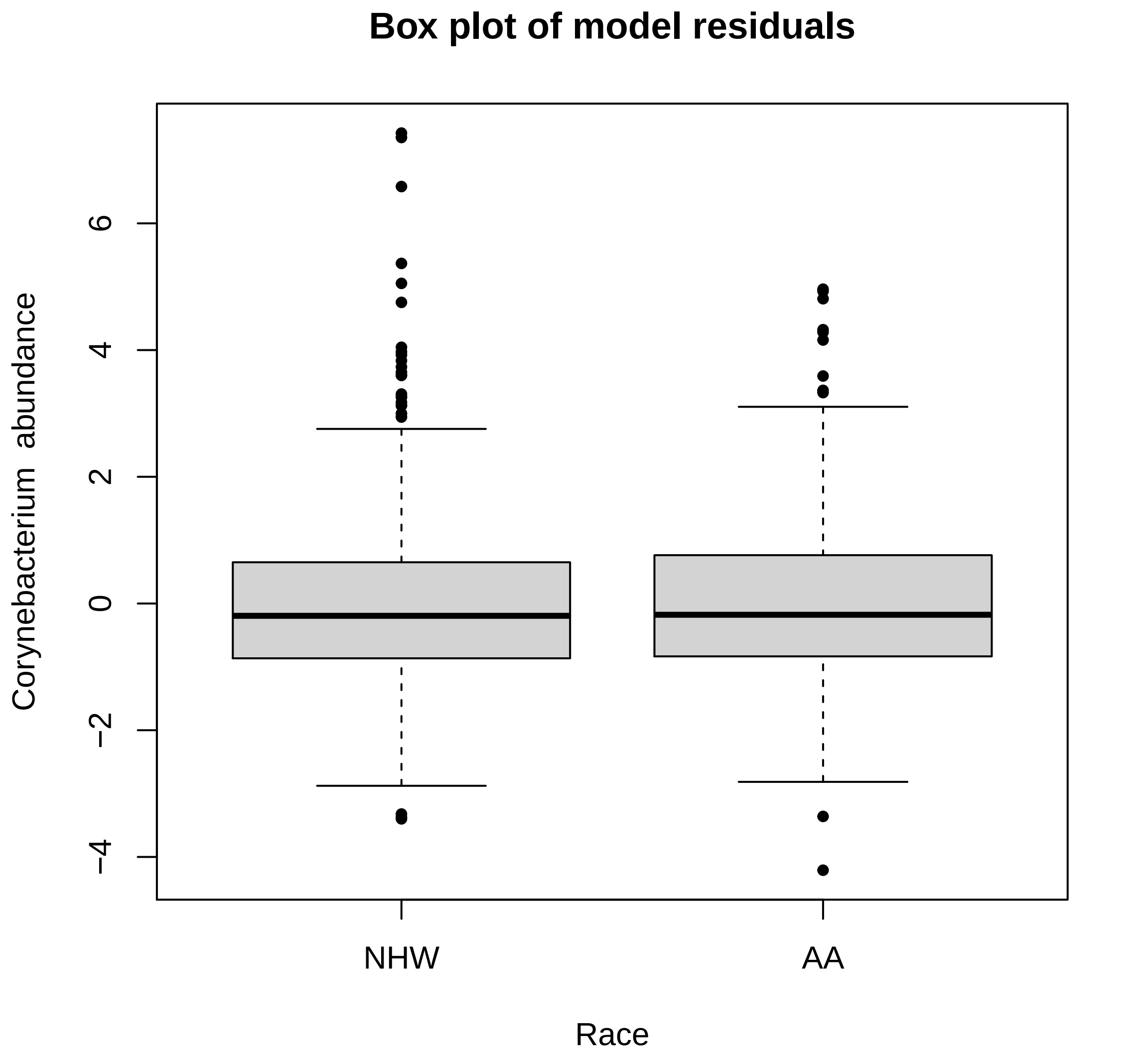
Box plot of model residuals
Corynebacterium abundance
6
4
2
0
−2
−4
NHW
AA
Race

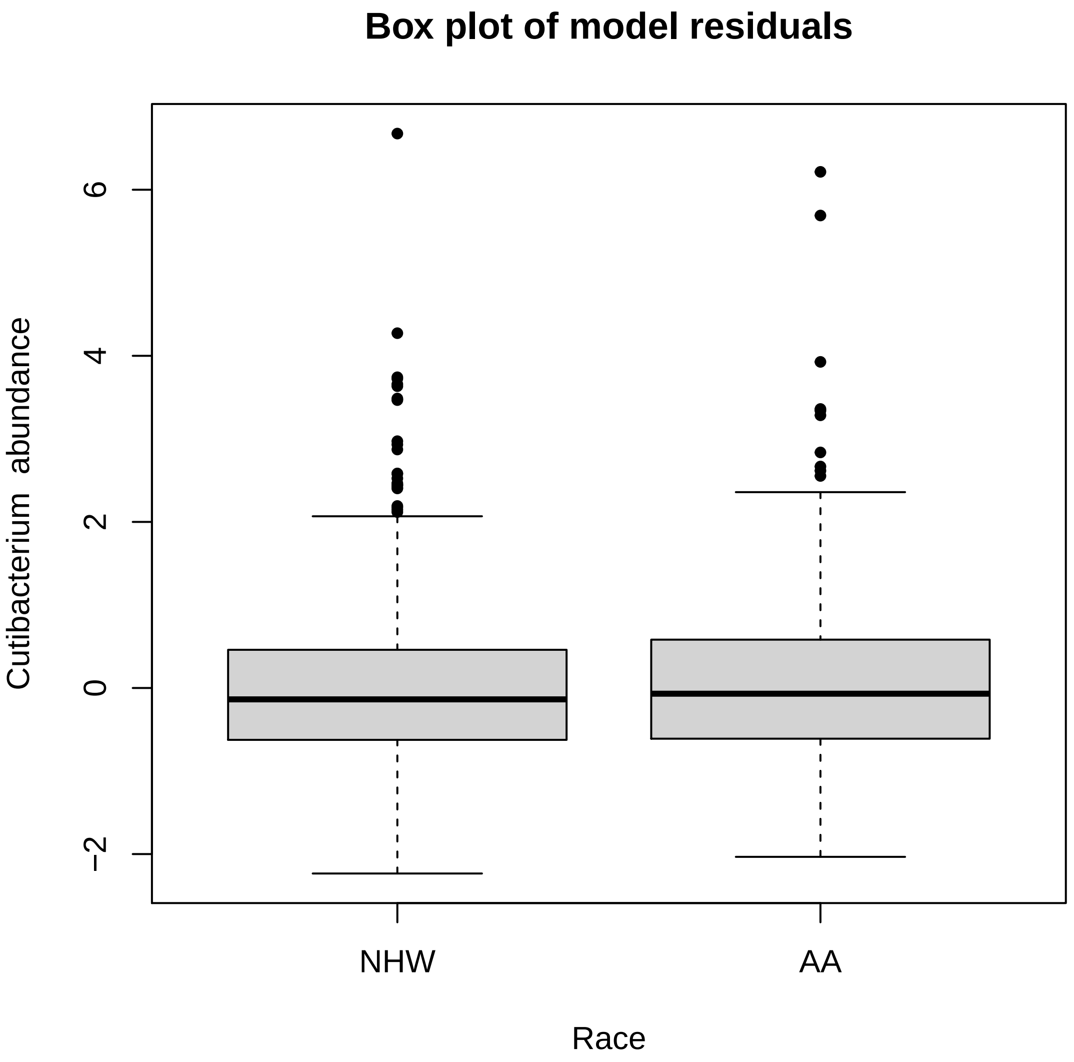
Box plot of model residuals
Cutibacterium abundance
6
4
2
0
−2
NHW
AA
Race
